# A new method for identifying causal genes of schizophrenia and anti-tuberculosis drug-induced hepatotoxicity

**DOI:** 10.1038/srep32571

**Published:** 2016-09-01

**Authors:** Tao Huang, Cheng-Lin Liu, Lin-Lin Li, Mei-Hong Cai, Wen-Zhong Chen, Yi-Feng Xu, Paul F. O’Reilly, Lei Cai, Lin He

**Affiliations:** 1 Bio-X Institutes, Key Laboratory for the Genetics of Developmental and Neuropsychiatric Disorders (Ministry of Education), Shanghai Key Laboratory of Psychotic Disorders (No. 13dz2260500), Shanghai Jiaotong University, Shanghai 200030, China; 2 Institute of Health Sciences, Shanghai Institutes for Biological Sciences, Chinese Academy of Sciences, Shanghai 200031, China; 3 School of Life Sciences and Biotechnology, Shanghai Jiaotong University, Shanghai, 200240, China; 4 Shanghai Mental Health Center, Shanghai Jiaotong University, Shanghai, 200240, China; 5 MRC SGDP Centre, Institute of Psychiatry, Psychology and Neuroscience, King’s College London, London WC2R 2LS, United Kingdom

## Abstract

Schizophrenia (SCZ) may cause tuberculosis, the treatments for which can induce anti-tuberculosis drug-induced hepatotoxicity (ATDH) and SCZ-like disorders. To date, the causal genes of both SCZ and ATDH are unknown. To identify them, we proposed a new network-based method by integrating network random walk with restart algorithm, gene set enrichment analysis, and hypergeometric test; using this method, we identified 500 common causal genes. For gene validation, we created a regularly updated online database ATDH-SCZgenes and conducted a systematic meta-analysis of the association of each gene with either disease. Till now, only *GSTM1* and *GSTT1* have been well studied with respect to both diseases; and a total of 23 high-quality association studies were collected for the current meta-analysis validation. Finally, the *GSTM1* present genotype was confirmed to be significantly associated with both ATDH [Odds Ratio (OR): 0.71, 95% confidence interval (CI): 0.56–0.90, *P* = 0.005] and SCZ (OR: 0.78, 95% CI: 0.66–0.92, *P* = 0.004) according to the random-effect model. Furthermore, these significant results were supported by “moderate” evidence according to the Venice criteria. Our findings indicate that *GSTM1* may be a causal gene of both ATDH and SCZ, although further validation pertaining to other genes, such as *CYP2E1* or *DRD2*, is necessary.

Schizophrenia (SCZ) is a severe, disabling, and chronic mental disorder that affects approximately 1% of the general population^1^. Recent research has identified a potential link between SCZ and an increased risk of contacting tuberculosis (TB)^2^, a devastating infectious disease that remains the major leading cause of death worldwide^3^. The most commonly used anti-TB drugs, although effective^4^, can induce anti-tuberculosis drug-induced hepatotoxicity(ATDH), the most frequent and serious side effect of TB therapy. ATDH, also known as anti-tuberculosis drug-induced liver injury (ATDILI)^5^, is caused by the drugs’ reactive metabolites rather than direct toxicity and can impede scheduled treatment, leading to increased complications morbidity and reduced treatment compliance^6^. Moreover, anti-TB drugs can also induce or cause relapse of psychiatric disorders, including SCZ-like disorders^7^,^8^,^9^.

Few previous studies have addressed the potential relationship between ATDH and SCZ, which belong to two different disease categories. Although we previously proposed that *GST* genes represent a link between ATDILI and SCZ^10^, the molecular basis underlying the connection between ATDH and SCZ is currently unclear. Therefore, identification of common causal genes underlying both diseases would greatly benefit our understanding of the relationship between ATDH and SCZ. In addition, knowledge about this link would also be beneficial to people at risk of developing SCZ and/or TB, particularly in the context of anti-TB drug therapy.

The identification of all genes related to both ATDH and SCZ and subsequent evaluation of their biological roles *in vitro* and *in vivo* comprise one possible strategy for elucidating their molecular mechanisms. However, this approach represents an insurmountable challenge for the large-scale identification of disease-related genes. Therefore, we propose a new network-based pipeline for analyzing the connection between these two diseases and prioritizing the identification of potential common key causal genes that influence the development of these two diseases by integrating network random walk with restart (RWR) algorithm, gene set enrichment analysis (GSEA), and hypergeometric test.

Network-based analyses, which assume that neighboring genes have similar effects, have demonstrated great promise for the identification of causal factors and key driver genes associated with a particular disease^11^,^12^. However, in our proposed novel network-based analysis pipeline to identify candidate key causal genes of both ATDH and SCZ, we first applied RWR to expand the known ATDH- and SCZ-related genes, for which little overlap had previously been detected. RWR simulates a random walker on the network starting from seed genes and moving toward randomly chosen interacting neighbors at each step^13^. After walking many steps, the probability of moving to each node on the network tends to stabilize, and novel candidate disease genes can be identified. A GSEA like method was then adopted to find the peak of a running sum curve, which indicates the stop point for RWR expansion. RWR expansion identified many common genes shared by the expanded ATDH and SCZ gene lists. Subsequently, a hypergeometric test was used to test all genes on the network to determine whether neighboring genes were also significantly enriched among the genes common to both ATDH and SCZ. A positive result indicated a key gene driver that could affect both ATDH- and SCZ- related genes.

Additionally, we conducted a field synopsis or systemic meta-analysis of published studies to assess whether these key gene drivers represent causal genes common to both ATDH and SCZ by validating the associations between gene polymorphisms and ATDH or SCZ. Systemic meta-analysis is a reliable approach that involves pooling both statistically significant and non-significant results from individual studies to generate a more precise conclusion^14^,^15^,^16^. In the end, 500 candidate genes were detected as common causal ones for both ATDH and SCZ using our new method. Among these genes, the glutathione S-transferase Mu-1 gene (*GSTM1)* was validated as a causal factor of these two diseases. Furthermore, we continue to update the validation of other genes in our newly created online database ATDH-SCZgenes (http://www.bio-x.cn/ATDH-SCZgenes.html).

## Results

### Identification of causal genes for both ATDH and SCZ

Five known ATDH/ATDILI-related genes and 1, 305 known SCZ-related genes were collected from GenBank. The human STRING network database was then used to map five and 1, 079 genes were mapped on the network for ATDH and SCZ, respectively. Using these mapped genes, we predicted 3, 045 and 1, 458 possible ATDH- and SCZ-related genes, respectively, with potential effects on known disease-related genes. Among these, we identified 878 overlapping genes between the expanded ATDH and SCZ gene lists, which are shown in Supplementary Table S3. Furthermore, 500 genes with significant false discovery rate (FDR-) corrected hypergeometric test *P* values (<10^−8^) were identified as causal factors for both ATDH and SCZ (Supplementary Table S4).

To validate these 500 genes as common causal factors for both ATDH and SCZ, we created a regularly updated online database ATDH-SCZgenes (www.bio-x.cn/ATDH-SCZgenes.html) and conducted a field synopsis/systemic meta-analysis to analyze associations of polymorphisms in each potential gene with ATDH or SCZ. During validation, GWAS data were firstly collected, and then, candidate gene association studies data were collected. To date, no GWAS for ATDH have been reported yet. Among these genes, only *GSTM1, CYP2E1* and glutathione S-transferase theta-1(*GSTT1)* (*P* = 5.61E-22, 4.54E-18 and 3.87E-09, respectively, Table 1) have been reported to associate with both ATDH and SCZ; all other genes were reported to associate with at most either disease alone. Genes with significant effects only on SCZ, which were obtained from the SzGene database^17^ or a systemic meta-analysis of genome wide association study (GWAS) data provided by Ricopili^18^, are listed in Supplementary Table S5; in the future, association of these genes with ATDH will require testing.

### Characteristics of the included studies

A flow diagram summarizing the study selection process is shown in Supplementary Fig. 1. A total of 33 and 24 potentially relevant studies regarding the association between *CYP2E1*/*GSTM1/GSTT1* polymorphisms and the respective risks of ATDH and SCZ were identified after an initial screening based on the titles and/or abstracts of the candidate articles. After the second screening, 699 cases and 2,546 controls from fifteen studies of *CYP2E1* and ATDH, 679 cases and 2,289 controls from fourteen studies of *GSTM1* and ATDH, 592 cases and 2,569 controls from fourteen studied of *GSTT1* and ATDH, one case-control study of *CYP2E1* and SCZ, 1,469 cases and 1,605 controls from seven studies of *GSTM1* and SCZ, and 936 cases and 971 controls from five studies of *GSTT1* and SCZ were identified. The detailed characteristics of each study were listed in Supplementary Table S6. All studies confirmed the same complete loss of *GSTM1* or *GSTT1* mutation. Because the association studies involving *CYP2E1*did not meet the fifth inclusion criterion (i. e., at least three studies regarding the association of each gene with either disease), studies involving this gene were omitted (detailed information about *CYPE2E1* can be obtained from the online database). The genotype distributions of the cases and controls from all studies involving *GSTM1* or *GSTT1* in the context of ATDH and SCZ are presented in Tables 2 and 3, respectively. The null genotype refers to homozygous gene loss, which indicates a loss of gene function, and the present genotype includes both heterozygous gene loss and homozygous complete gene presence.

### Association of *GST* polymorphisms with ATDH

**Figure 1:**
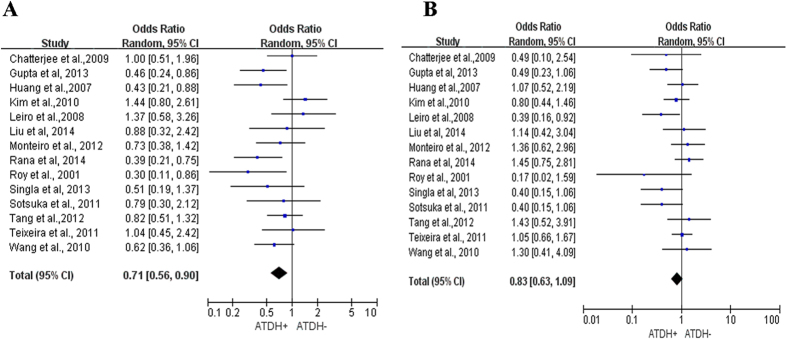
Forest plots from meta-analysis of *GSTM1/GSTT1* polymorphisms and ATDH. (**A**) Summary of the ORs and corresponding 95% CIs for the *GSTM1* present genotype; (**B**) summary of the ORs and 95% CIs for the *GSTT1* present genotype.

Evaluations of the associations between *GSTM1/GSTT1* polymorphisms and the risk of ATDH are summarized in Fig. 1. Significant heterogeneity in the effects of these polymorphisms was observed for *GSTM1*, but not for *GSTT1* [*P* = 0.088 and *I*^2^ = 36%, 95% confidence interval (CI): 0-0.662 for *GSTM1*, *P* = 0.12 and *I*^2^ = 32%, 95% CI: 0-0.61 for *GSTT1*]. Because fewer than 20 studies were included in the meta-analysis, the random-effect model was used for both *GSTM1* and *GSTT1*. The frequencies of *GSTM1/GSTT1* null genotypes among the cases were higher than those among the controls (cases vs. controls: 51.25% vs. 42.85% for *GSTM1*, and 33.61% vs. 31.88% for *GSTT1*). The *GSTM1* present genotype was significantly associated with a decreased risk of ATDH [odd ratio (OR): 0.71, 95% CI: 0.56–0.90, *P* = 0.005] (Fig. 1A). No significant association between the *GSTT1* present genotype and ATDH was observed (OR 0.83, 95% CI: 0.63–1.09, *P* = 0.18; Fig. 1B).

### Association of GST polymorphisms with SCZ

**Figure 2:**
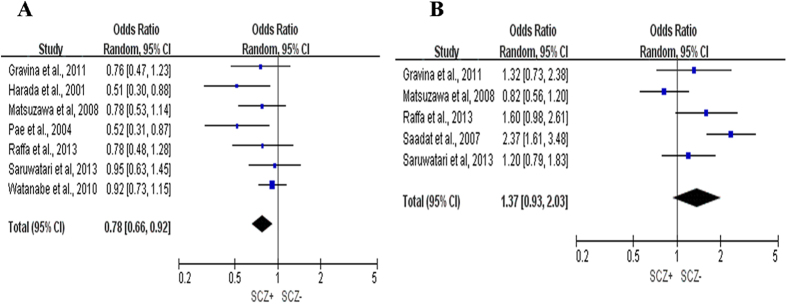
Forest plots from meta-analysis of *GSTM1/GSTT1* polymorphisms and SCZ. (**A**) Summary of the ORs and corresponding 95% CIs for the *GSTM1* present genotype; (**B**) summary of ORs and 95% CIs for the *GSTT1* present genotype.

The combined results regarding the association between *GSTM1/GSTT1* polymorphisms and the risk of SCZ are presented in Fig. 2. Significant effect heterogeneity was observed in relation to *GSTT1*, but not for *GSTM1* (*P* = 0.27 and *I*^2^ = 21%, 95% CI: 0–0.642 for *GSTM1*, *P* = 0.004 and *I*^2^ = 74%, 95% CI: 0.351–0.895 for *GSTT1*). Again, the random-effect model was used for both genes because of the small number of studies. The *GSTM1* null genotype and the *GSTT1* present genotype were more frequent among cases than among controls (cases vs. controls: 56.71% vs. 51.15% for the *GSTM1* null genotype, 31.2% vs. 38.62% for the *GSTT1* null genotype). Statistically significant association were observed between the *GSTM1* present genotype and a decreased risk of SCZ (OR 0.78, 95% CI: 0.66–0.92, *P* = 0.004; Fig. 2A), and between the *GSTT1* present genotype and SCZ (OR 1.37, 95% CI: 0. 93–2.03, *P* = 0.11; Fig. 2B).

### Sensitivity analyses and publication bias

A sensitivity analysis was conducted via sequential analysis after omitting one study at a time to assess the effects of individual studies on the overall meta-analysis estimate. When one study was excluded, the *P* values for overall effects ranged from 0.004 to 3.97E-5 and from 0.17 to 0.68, in the *GSTM1/GSTT1* and ATDH fixed-effect model analyses, respectively; for the fixed-effect model analyses of *GSTM1/GSTT1* and SCZ, the respective *P* values for overall effects ranged from 0.001 to 0.015 and from 0.005 to 0.32. These values indicate the stability of these analytical results.

Furthermore, Harbord’s test indicated no significant publication bias in the overall meta-analysis except for studies of the association between *GSTT1* and ATDH and the association of *GSTM1*and SCZ (*P* = 0.56 for *GSTM1* vs. ATDH; *P* = 0.08 for *GSTT1* vs. ATDH; *P* = 0.0637for *GSTM1* vs.SCZ; *P* = 0.91 for *GSTT1* vs. SCZ).

### Credibility of meta-analysis results

The Power and Sample Size Program^19^ indicated that the total sample size had a power >90% to detect significant associations of the *GSTM1* present genotype with ATDH and SCZ at ORs of 0.71 and 0.78, respectively. Furthermore, the respective false-positive reporting probabilities (FPRP) at a *P* value < 0.05 for the associations of *GSTM1* present genotype with ATDH and SCZ were 0.088 and 0.063, with respective ORs of 0.6 and 0.7 (Supplementary Table S7).

Moreover, the strict inclusion criteria of this meta-analysis had addressed the genotyping quality. For the meta-analysis of ATDH studies, the n_minor_ for the *GSTM1* null genotype was 1,329, and a grade of A was given. The *I*^2^ was 36% and a grade of B was given. After excluding a 2001 study by Roy^20^, a significant association remained between the *GSTM1* present genotype and ATDH (*P* = 0.01), with a Harbord’s test *P* value of 0.868^20^. For the meta-analysis of SCZ studies, the n_minor_ for the *GSTM1* present genotype was 1,654, and a grade of A was given. A grade of A was also given for the *I*^2^ of 21%. After excluding 2001 study by Harada^21^, a significant association remained between the *GSTM1* present genotype and SCZ (*P* = 0.014), with a Harbord’s test *P* value of 0.138. According to the Venice criteria^22^,^23^, “moderate” cumulative evidence supported significant associations of the *GSTM1* present genotype with both ATDH and SCZ.

## Discussion

As noted previously, ATDH can impede TB treatment schedules and thereby increase complications morbidity^6^. Psychiatric disorders has also been reported to represent an additional adverse effect of anti-TB drugs^5^,^7^. SCZ is a severe psychiatric disorder, given that TB and SCZ are frequent co-morbid conditions^2^,^7^, understanding the molecular basis for the relationship between ATDH and SCZ would not only facilitate personalized medicine by allowing physicians to identify potential exacerbation of SCZ and induction of ATDH patients, but could help to elucidate the molecular mechanisms common to both diseases. Previously, however, the relationship between ATDH and SCZ has been unclear, and knowledge about common biological determinants between these conditions has yet to emerge.

Previously, we proposed that *GST* genes might serve as a link between ATDILI and SCZ^10^. To provide a global perspective of the hidden molecular basis for the connection between ATDH and SCZ, we proposed a protein-protein interaction (PPI) network-based analysis pipeline that would prioritize possible key drivers that might affect both ATDH and SCZ by extending ATDH-related or SCZ-related gene sets to neighboring genes and identifying key causal genes that overlap in these extended gene sets. Although a direct overlap of known gene sets is the most intuitive way of exploring a genetic association of two diseases, such a direct overlap would fail to reflect the complexity of the intertwined regulation between these two diseases. Moreover, because the known disease gene sets are incomplete, the lacking genes could lead to a naive comparison. In contrast, in our proposed method, we qualitatively analyzed the potential nature of a gene as a key driver of two diseases using the hypergeometric test. Furthermore, identified candidate key drivers were ranked according to *P* value significance and network microenvironments (i.e., potential interaction neighbors). The ability to reconstruct potential causal signaling will facilitate further molecular biology studies.

In addition, identified key drivers can provide clues about therapeutic interventions that affect genes from both diseases. We note that EnrichNet use similar methods to determine the enrichment of one gene set into a particular pathway or other signatures^24^; these include employing information from the PPI network and extending the seed genes to neighboring gene using the RWR method. However, EnrichNet measures the significance of a relationship between a series of extended genes and a particular pathway according to different RWR distance cutoffs, whereas our method determines the best RWR distance cutoff. Specifically, we adopted the concept of the leading-edge subset used in GSEA, which is a sorted the neighbor gene lists based on RWR distances, and labeled the seed genes as positive and other genes as negative. Using our method, the peak at which the running sum maximally deviates from zero determines the best RWR distance cutoff; subsequently, we can determine the best extended genes for either disease and study the overlaps to identify candidate key causal genes using the hypergeometric test. Therefore, EnrichNet and our analysis pipeline use similar methods in different ways to address different problems.

In the present study, we used our novel pipeline analysis to identify 500 genes with a P < 10^−8^ as possible causal genetic factors shared by ATDH and SCZ. Given the nature of ATDH, however, it is difficult to collect a sufficient number of patients with both ATDH and SCZ in the absence of other comorbidities. Because systemic meta-analysis is considered as a powerful tool for the identification of genes associated with a certain disease, we have created and regularly updated the online database ATDH-SCZgenes (http://www.bio-x.cn/ATDH-SCZgenes.html) to analyze and validate the association between candidate genes and both diseases.

Among the 500 evaluated candidate genes, to date, only *GSTM1, CYP2E1* and *GSTT1* were found to associate with both ATDH and SCZ; all others associated with neither or only one of the diseases. In the analysis of *CYP2E1*and ATDH, a pooled OR of 1.2(95% CI: 0.85–1.68) was determined for the rs2031920 (−1053C > T) polymorphism, whereas a fixed-effect model yielded a pooled OR of 1.3(95% CI: 1.06–1.59). Only one previous study has evaluated and identified a positive association between *CYP2E1* and SCZ. In contrast, the SzGene database^17^ and a systemic meta-analysis of SCZ GWAS data^18^ failed to corroborate that significant association, but consistently supported *DRD2* to be significantly associated with SCZ. *DRD2* has been reported as a prominent genetic risk factor for susceptibility to severe alcoholism^25^, which is associated with liver damage. Further validation of the associations of these genes with ATDH and SCZ is needed in the future.

Through a systemic meta-analysis, we validated a significant association of the *GSTM1* null genotype with increased risks of both ATDH and SCZ, and these significant results were supported by “moderate” evidence according to the Venice criteria^22^, suggesting that *GSTM1* may be a causal factor shared by both ATDH and SCZ.

ATDH has been widely suggested to be a Glutathione S-transferases (*GSTs*) related disease^26^,^27^. Through conjugating glutathione with free radical scavengers and facilitating their elimination from the body to reduce potential toxicities of target substances^28^, GSTs comprise a superfamily of detoxification enzymes that are encoded in two main genes: the *GSTM1* gene on chromosome 1p13.3, which encodes for cytosolic GST class Mu 1 enzyme, and the *GSTT1* gene on chromosome 22q11.2, which encodes for cytosolic GST class theta 1 enzyme^29^,^30^. Both *GSTM1* and *GSTT1* may harbor a null mutation comprising a complete deletion of the respective gene via unequal homologous crossover, and homozygous null mutations can lead to a variable, tissue-specific loss of GSTs activity^31^.

**Figure 3:**
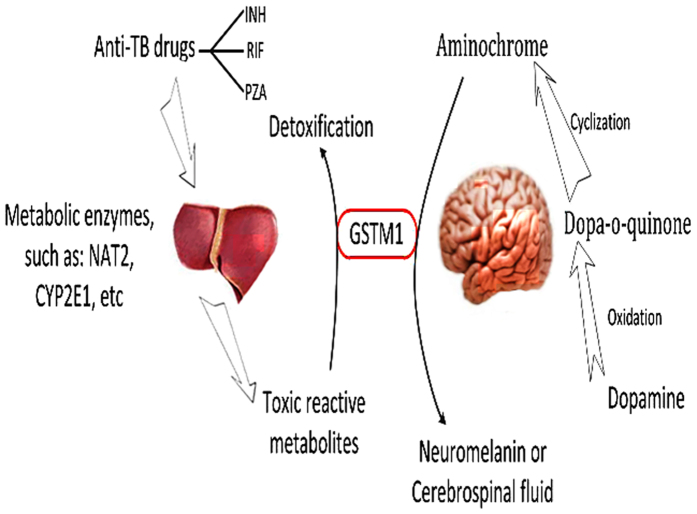
Schematic illustration of the roles of GSTM1 in liver and brain. INH, isoniazid; RIF, rifampin; PZA, pyrazinamide; NAT2, arylamine N-acetyltransferase2; CYP2E1, cytochrome P450 2E1.

*GSTM1* is mainly expressed in the liver and brain^32^, a fact that supports the significant associations observed between this gene and both ATDH and SCZ. Furthermore, *GSTM1* not only detoxifies the toxic metabolites of anti-TB drugs generated by CYP2E1 in the liver, but also catalyzes the conjugation of glutathione with aminochrome and dopa-o-quinone metabolites of oxidized dopamine in the brain^32^. Reactive oxygen species are generated at high rates in the brain, and regulation of the growth and pruning of neurons is partly attributed to the redox mechanism that controls the balance between neuro destructive oxidants and neuro protective antioxidants^33^. Therefore, GSTM1 inactivation due to the *GSTM1* null genotype not only causes liver injury but also promotes the accumulation of neuro destructive oxidants and consequent development of SCZ (Fig. 3).

Compared with our previous study^10^, the present study exhibits the following improvements: (1) the current meta-analysis or field synopsis is more compressive and systematic and involves as many candidate genes as possible including *GST* genes; (2) the current analysis is updated regularly using the online database ATDH-SCZgenes; and (3) more strict statistical methods were used in this analysis, including ORs instead of risk ratios and a threshold P value for publication bias of 0.1 instead of 0.05. However, this study also has some limits, including use of the STRING database, a functional association network without direction. The availability of a disease specific directed network might lead to more comprehensive and concrete conclusions using the current method. Additionally, the significant association of *GSTM1* with SCZ should be interpreted with caution, as the *P* value for the publication bias test was 0.0637; this value represents the evidence of small-study effects with all studies included. However, our sensitivity analysis to assess the effects of individual studies on the overall meta-analysis estimate yielded *P* values for overall effects of 0.001–0.015 in the *GSTM1* and SCZ analysis, indicating the stability of positive results obtained with these analyses. Furthermore, till now, no GWASs on ATDH have been reported and the number of reported ATDH-associated genes is small; therefore, only *GSTM1* and *GSTT1* genes could be validated in the current systemic meta-analysis. Validation of other causal genes must be performed in future studies.

In summary, we provide a list of possible causal genetic factors associated with both ATDH and SCZ, and have identified a shared genetic basis of these two diseases. Furthermore, we have created and will regularly update the ATDH-SCZgenes online database to validate the association of each candidate gene with either disease. Finally, *GSTM1* was validated as a causal factor of both ATDH and SCZ, whereas other genes such as *CYP2E1* and *DRD2* will require further validation.

## Methods

### Ethics statement

The current research was performed in compliance with the Helsinki Declaration and was approved by the Bioethics committee of the Bio-X Institutes of Shanghai Jiaotong University. Informed consent was obtained from all subjects.

### Network-based analysis

**Figure 4:**
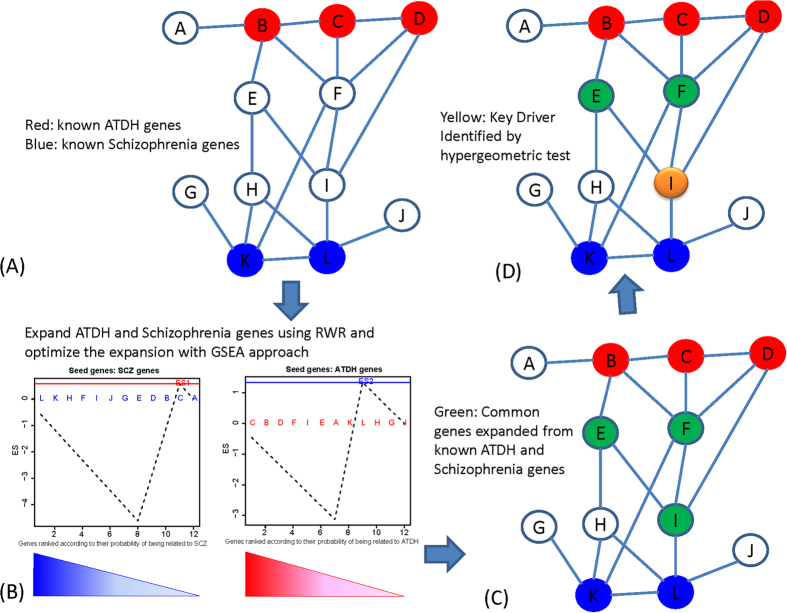
Identification of the key drivers of both ATDH and SCZ. (**A**) Known ATDH and SCZ genes were mapped onto the STRING network. (**B**) ATDH genes were expanded using RWR and the known ADTH genes as seed genes. The stop point was determined using a running sum curve reflective of the overlap between the top expanded ATDH genes and known SCZ genes. SCZ genes were expanded in a similar manner. (**C**) Common genes between ATDH and SCZ were highlighted for further key driver evaluation. (**D**) The neighbor genes of candidate key drivers were tested for overlap significance with common disease genes. These neighbors of key drivers should significantly affect the more common disease genes.

All known ATDH/ATDILI- and SCZ-related genes reported (including GWAS) to have significant effects on the relative disease by at least one study were collected using GenBank (http://www.ncbi.nlm.nih.gov/gene/), and are listed in Supplementary Table S1. These genes were used as seed genes in the subsequent analysis. To explore the causal factors of ATDH and SCZ, a novel network-based analysis pipeline was developed per the workflow shown in Fig. 4. First, established seed genes related to ATDH or SCZ, were mapped onto the highest confidence human STRING network (version 9.1, confidence score > 0.900), which included a total of 8,823 genes^34^. These seed genes were subsequently expanded on the network using the RWR method^35^ as follows: a PPI network G = (V, E) comprised of a set of proteins V and a set of interactions E is represented by an *n* × *n* adjacency matrix *A*, where *n* is the number of proteins. The entries at row *i* and column *j* are set to 1 if protein *i* interacts with protein *j*; otherwise they are set to 0. First, the adjacency matrix *A* was normalized in a column-wise maner as follows


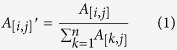


The random walker initiates at a set of seed genes, (e.g., known disease genes). The initial state *P*_0_ can be formulated as a column vector


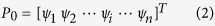


where *ψ*_*i*_ is set to 1/*m* for *m* seed genes and to 0 for the other genes on the network, and *n* is the number of genes on the network. The random walker randomly visits the adjacent genes for every *t* → *t* + 1. The state probabilities *P*_*t*+1_ at time *t* + 1 are calculated as follows


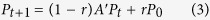


where *P*_*t*_ represents the state probabilities at time *t*, *r* is the restart probability (i.e., starting from the seed genes again), which was set to 0.7 as suggested by multiple previous studies^36^,^37^,^38^,^39^,^40^,^41^. This process was repeated until a steady-state was reached; this was defined as a difference between two steps of <1e-6 according to previous studies^38^,^42^,^43^.

After expansion, all genes on the network were assigned disease gene probability. To determine the boundaries of gene expansion, we developed a new idea from GSEA and calculated the running sum from the top to bottom of the ranked gene list. Specifically, when we expanded ATDH genes from the ATDH seed genes, all genes on the network were ranked according to the likelihood of being an ATDH-related gene. If we encountered a gene that was not an established SCZ seed gene, 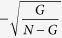 was added to the running sum, where *N* is the number of all network genes and *G* is the number of known established SCZ seed genes; otherwise, 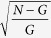 was added^44^,^45^. Based on the running sum, a peak of network expansion from the ATDH seed genes was determined. This cutoff was then used to obtain a list for ATDH seed gene expansion. A list for SCZ seed gene expansion was generated similarly. Common genes between these two lists (i.e., overlapped genes) were studied because these might more robustly reflect the common genetic basis of the two diseases. Finally, we screened all possible causal gene factors by testing their neighbors for overlapped genes using the hypergeometric test *P* value^46^.


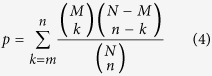


where N is the number of all network genes, M is the number of common disease genes, n is the number of neighbor genes, and m is the number of neighbor genes that are common disease genes. To control the FDR, hypergeometric test *P* values were adjusted according to the method published by Benjamini & Hochberg^47^. The analysis was conducted using R software version 3.1.2 and RWR code was obtained from RWOAG package (https://r-forge.r-project.org/R/?group_id=1126).

### Literature search

The digital medical databases PubMed, Scopus, ISI Web of Science, EMBASE, Chinese National Knowledge Infrastructure(CNKI) and the Chinese BioMedical Literature Database were searched for studies with publication dates up to 03/31/2016 using the following keywords: (“anti-tuberculosis drug-induced liver injury”, “anti-tuberculosis drug-induced hepatotoxicity”, “ATDH” or “ATDILI”) and (“Schizophrenia”), together with the full name or abbreviation of each candidate causal gene, including: “cytochrome P4502E1”, “CYP2E1”, “UDP-glucuronosyltransferase 1A6”, “UGT1A6”, “glutathione S-transferaseM1”, “GSTM1”, and “glutathione S-transferase T1” or “GSTT1”. The references of retrieved articles were also reviewed to identify additional relevant literatures.

### Inclusion and exclusion criteria

Articles included in the meta-analysis complied with the following criteria: (1) original case-control association studies based on randomly selected individuals; (2) Provision of complete genotype distribution data; (3) cases comprising TB patients with ATDH and controls comprising TB patients without ATDH or (4) cases comprising SCZ patients and controls comprising healthy subjects; and (5) at least three available studies regarding the association of each gene with either disease. Other studies, such as case-only studies, duplications, animal studies, comparisons of laboratory methods, editorials, and review articles were excluded. Studies were further evaluated using the Quality-Evaluation Score Sheet^16^ (version 2.0) (Supplementary Table S2); a score of 8–10 indicated a high quality study.

### Data extraction

Data extraction was performed independently by two reviewers using a standardized protocol and reporting form. Discrepancies between the two reviewers were resolved by further discussion with a third party. For overlapping studies, the study with the larger sample size was retained for the meta-analysis. The recorded study characteristics included: (1) the first author’s name, (2) publication year, (3) sample ethnicity, (4) control and case characteristics, (5) methods used for genotyping and (6) target genes.

### Statistical analysis

The strengths of the associations between gene polymorphisms and the risk of ATDH or SCZ were measured using ORs with corresponding 95% CIs. Pooled ORs were calculated for null vs. present genotype camparisons. If the total number of studies was <20, the random-effect model of meta-analysis was used to calculate the pooled ORs according to the DerSimonian–Laird method; if the number was ≥20, the fixed-effect model was applied according to the Mantel-Haenszel method^48^,^49^. Inter-study heterogeneity was assessed using the chi-square-based Q-test (Cochran’s Q statistic), and a strict *P* value < 0.1 was considered statistically significant^14^.

The *I*^2^ values 
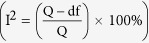
 and corresponding 95% CIs were also calculated to describe the percentages of variability in the effect estimates that were attributable to heterogeneity rather than sampling error, an *I*^2^ > 50% was roughly considered to indicate substantial heterogeneity^50^. This formula use Q as well as degrees of freedom^50^,^51^. A sensitivity analysis, in which one study at a time was removed prior to analysis, was conducted to evaluate whether a single study would significantly affect the results. This analysis used a model other than the model used to calculate the pooled ORs. Harbord’s test was used to test small-study effects among which publication bias might be a contributor, and a *P* value < 0.1 was considered representative of statistically significant publication bias^52^. All statistical analyses were implemented in Review Manger 5.2 (The Nordic Cochrane Centre, Copenhagen, Denmark) and Stata version 11.2 (Stata Corporation, College Station, TX, USA).

### Credibility of meta-analysis results

A power analysis was performed using the Power and Sample Size Program with α = 0.05 as the level of significance; effects sizes were estimated from the meta-analyses^19^. To assess the noteworthiness of an association, the FPRP^34^ was estimated using a FPRP threshold of 0.2 and prior probabilities of 0.05–10^−6^. Cumulative evidence for genetic associations of GSTM1 and GSTT1 present genotypes with ATDH and SCZ, respectively, were assessed according to the Venice interim criteria, which include the amount of evidence, replication of results and protection from bias^22^. Regarding the amount of evidence, grades of A, B, and C were given for a n_minor_ > 1,000, 100–1,000 and <100, respectively, where n_minor_ refers to the total number of cases and controls with the least frequent genotype. Regarding replication, grades A, B, and C were given for *I*^2^ values < 25%, 25–50% and >50%, respectively. Regarding protection from bias, any of the following criteria should be met: (1) combined OR of 0.87–1.15; (2) high genotyping quality with a low genotyping error rate; (3) retained statistical significance after excluding the first published study; or (4) no evidence of small-study effects according to a Harbord regression test (significance: *P* < 0.05^53^).

### Data accessibility

The datasets supporting this article have been uploaded as part of the Supplementary Material.

## Additional Information

**How to cite this article**: Huang, T. *et al*. A new method for identifying causal genes of schizophrenia and anti-tuberculosis drug-induced hepatotoxicity. *Sci. Rep.* **6**, 32571; doi: 10.1038/srep32571 (2016).

## Supplementary Material

Supplementary Information

## Figures and Tables

**Table 1:** The shared key causal genes for ATDH and SCZ.

Genes	Number of neighbors	Number of neighbors that are common disease	FDR corrected *P*
GSTM1	48	33	5.61E-22
CYP2E1	70	36	4.54E-18
GSTT1	13	11	3.87E-09

**Table 2:** Genotype distributions of GSTM1/T1 polymorphisms among ATDH as cases and non-ATDH as controls.

First author, year	Ethnicity	Cases			Controls			P*
		Null	Present	Total	Null	Present	Total	
*GSTM1* polymorphism								
Chatterjee, 2009	Indian	25	26	51	49	51	100	0.998
Gupta, 2013	Indian	21	29	50	61	185	246	0.013
Huang, 2007	Chinese	42	21	63	29	34	63	0.02
Kim, 2010	Korea	26	31	57	104	86	190	0.226
Leiro, 2008	Spanish	12	23	35	25	35	60	0.477
Liu, 2014	Chinese	14	6	20	96	47	143	0.798
Monteiro, 2012	Brazilian	21	38	59	34	84	118	0.358
Rana, 2014	Indian	19	36	55	42	203	245	0.004
Roy, 2001	Indian	17	16	33	8	25	33	0.022
Singla, 2013	Indian	10	7	17	165	226	391	0.175
Sotsuka, 2011	Japanese	12	8	20	50	42	92	0.645
Tang, 2012	Chinese	55	34	89	203	153	356	0.414
Teixeira, 2011	Brazilian	11	15	26	61	80	141	0.928
Wang, 2010	Chinese	63	41	104	54	57	111	0.079
Total		348	331	679	981	1308	2289	
*GSTT1* polymorphism								
Chatterjee, 2009	Indian	3	48	51	3	97	100	0.391
Gupta, 2013	Indian	11	39	50	30	216	246	0.067
Huang, 2007	Chinese	24	39	63	25	38	63	0.855
Kim, 2010	Korea	34	23	57	103	87	190	0.469
Leiro, 2008	Spanish	17	18	35	16	44	60	0.031
Liu, 2014	Chinese	13	7	20	97	46	143	0.8
Monteiro, 2012	Brazilian	11	48	59	28	90	118	0.442
Rana, 2014	Indian	14	41	55	79	164	245	0.308
Roy, 2001	Indian	5	28	33	1	32	33	0.087
Singla, 2013	Indian	8	9	17	102	289	391	0.056
Sotsuka, 2011	Japanese	7	13	20	40	52	92	0.486
Tang, 2012	Chinese	40	49	89	164	192	356	0.849
Teixeira, 2011	Brazilian	4	22	26	27	114	141	0.65
Wang, 2010	Chinese	40	49	89	164	192	356	0.849
Total		231	433	664	879	1653	2534	

The null genotype means homozygous loss of genes, and the present genotype includes heterozygous loss of genes and homozygous complete genes.

^*^P value for chi-square test of genotype distribution.

**Table 3:** Genotype distributions of GSTM1/T1 polymorphisms among SCZ and healthy control.

First author, year	Ethnicity	Cases			Controls			P*
		Null	Present	Total	Null	Present	Total	
*GSTM1* polymorphism								
Gravina, 2011	Italian	82	56	138	70	63	133	0.26
Harada, 2001	Japanese	57	30	87	87	89	176	0.014
Matsuzawa, 2008	Japanese	129	85	214	119	101	220	0.193
Pae, 2004	Korean	70	41	111	61	69	130	0.012
Raffa, 2013	Tunisian	79	59	138	63	60	123	0.329
Saruwatari, 2013	Japanese	77	77	154	99	104	203	0.818
Watanabe, 2010	Japanese	339	288	627	322	298	620	0.451
Total		833	636	1469	821	784	1605	
*GSTT1* polymorphism								
Gravina, 2011	Italian	25	113	138	30	103	133	0.364
Matsuzawa, 2008	Japanese	88	126	214	80	140	220	0.309
Raffa, 2013	Tunisian	59	79	138	67	56	123	0.059
Saadat, 2007	Iranian	52	240	292	99	193	292	9E-06
Saruwatari, 2013	Japanese	68	86	154	99	104	203	0.387
Total		292	644	936	375	596	971	

The null genotype means homozygous loss of genes, and the present genotype includes heterozygous loss of genes and homozygous complete genes.

^*^P value for chi-square test of genotype distribution.
