# Supplementary material for: A new method for identifying causal genes of schizophrenia and anti-tuberculosis drug-induced hepatotoxicity
Source: Sci Rep. 2016 Sep 1;6:32571. doi: 10.1038/srep32571 (PMC5007646; doi:10.1038/srep32571)
Supplement: Supplementary Information [file srep32571-s1.doc]

**A new method for identifying causal genes of Schizophrenia and anti-tuberculosis drug-induced hepatotoxicity**

Tao Huang^1,2^, Cheng-Lin Liu^3^, Lin-Lin Li^1^, Mei-Hong Cai^1^, Wen-Zhong Chen^4^, Yi-Feng Xu^1, 4^, Paul F. O’Reilly^5^, Lei Cai^1,4†^, Lin He^1,4†^

^1^Bio-X Institutes, Key Laboratory for the Genetics of Developmental and Neuropsychiatric Disorders (Ministry of Education), Shanghai Key Laboratory of Psychotic Disorders(No.13dz2260500), Shanghai Jiaotong University, Shanghai 200030, China,

2Institute of Health Sciences, Shanghai Institutes for Biological Sciences, Chinese Academy of Sciences, Shanghai 200031, China,

^3^School of Life Sciences and Biotechnology, Shanghai Jiaotong University, Shanghai, 200240, China,

^4^Shanghai Mental Health Center, Shanghai Jiaotong University, Shanghai, 200240, China,

^5^MRC SGDP Centre, Institute of Psychiatry, Psychology and Neuroscience, King's College London, LondonWC2R 2LS, United Kingdom.

Correspondence and requests for materials should be addressed to L.C .(cailei2010@126.com) or L.H. (email: helinhelin123@yeah.net)

**Supplementary Table S1**  Gene list for known ATDH/ATDILI-related genes and Schizophrenia-related genes

| Org_name | GeneID | Symbol | Aliases | description | map_location | OMIM |
| --- | --- | --- | --- | --- | --- | --- |
| ATDH/ATDILI-related genes | | |  |  |  |  |
| Homo sapiens | 1571 | CYP2E1 | CPE1, CYP2E, P450-J, P450C2E | cytochrome P450, family 2, subfamily E, polypeptide 1 | 10q26.3 | 124040 |
| Homo sapiens | 2944 | GSTM1 | GST1-1, GSTM1a-1a, GSTM1b-1b, GTH4, GTM1, H-B, MU, MU-1, GSTM1 | glutathione S-transferase mu 1 | 1p13.3 | 138350 |
| Homo sapiens | 2952 | GSTT1 |  | glutathione S-transferase theta 1 | 22q11.23 | 600436 |
| Homo sapiens | 10 | NAT2 | AAC2, NAT-2, PNAT | N-acetyltransferase 2 (arylamine N-acetyltransferase) | 8p22 | 612182 |
| Homo sapiens | 54578 | UGT1A6 | GNT1, HLUGP, HLUGP1, UDPGT, UDPGT 1-6, UGT1S, UGT1F, UGT1A6 | UDP glucuronosyltransferase 1 family, polypeptide A6 | 2q37 | 606431 |
| Schizophrenia-related genes | | |  |  |  |  |
| Homo sapiens | 4735 | 2-Sep | DIFF6, NEDD-5, NEDD5, Pnutl3, hNedd5 | septin 2 | 2q37 | 601506 |
| Homo sapiens | 5414 | 4-Sep | ARTS, BRADEION, CE5B3, H5, MART, PNUTL2, SEP4, hCDCREL-2, hucep-7 | septin 4 | 17q22 | 603696 |
| Homo sapiens | 989 | 7-Sep | CDC10, CDC3, NBLA02942A, SEPT7 | septin 7 | 7p14.2 | 603151 |
| Homo sapiens | 19 | ABCA1 | ABC-1, ABC1, CERP, HDLDT1, TGD | ATP-binding cassette, sub-family A (ABC1), member 1 | 9q31.1 | 600046 |
| Homo sapiens | 154664 | ABCA13 |  | ATP-binding cassette, sub-family A (ABC1), member 13 | 7p12.3 | 607807 |
| Homo sapiens | 5243 | ABCB1 | ABC20, CD243, CLCS, GP170, MDR1, P-GP, PGY1 | ATP-binding cassette, sub-family B (MDR/TAP), member 1 | 7q21.12 | 171050 |
| Homo sapiens | 23457 | ABCB9 | EST122234, TAPL | ATP-binding cassette, sub-family B (MDR/TAP), member 9 | 12q24 | 605453 |
| Homo sapiens | 215 | ABCD1 | ABC42, ALD, ALDP, AMN | ATP-binding cassette, sub-family D (ALD), member 1 | Xq28 | 300371 |
| Homo sapiens | 176 | ACAN | AGC1, AGCAN, CSPG1, CSPGCP, MSK16, SEDK | aggrecan | 15q26.1 | 155760 |
| Homo sapiens | 1636 | ACE | ACE1, CD143, DCP, DCP1, ICH, MVCD3 | angiotensin I converting enzyme | 17q23.3 | 106180 |
| Homo sapiens | 50 | ACO2 | ACONM, ICRD, OCA8 | aconitase 2, mitochondrial | 22q13.2 | 100850 |
| Homo sapiens | 23305 | ACSL6 | ACS2, FACL6, LACS 6, LACS2, LACS5 | acyl-CoA synthetase long-chain family member 6 | 5q31.1 | 604443 |
| Homo sapiens | 116285 | ACSM1 | BUCS1, MACS1 | acyl-CoA synthetase medium-chain family member 1 | 16p12.3 | 614357 |
| Homo sapiens | 60 | ACTB | BRWS1, PS1TP5BP1 | actin, beta | 7p22 | 102630 |
| Homo sapiens | 71 | ACTG1 | ACT, ACTG, BRWS2, DFNA20, DFNA26, HEL-176 | actin gamma 1 | 17q25 | 102560 |
| Homo sapiens | 8038 | ADAM12 | ADAM12-OT1, CAR10, MCMP, MCMPMltna, MLTN, MLTNA | ADAM metallopeptidase domain 12 | 10q26 | 602714 |
| Homo sapiens | 8728 | ADAM19 | FKSG34, MADDAM, MLTNB | ADAM metallopeptidase domain 19 | 5q33.3 | 603640 |
| Homo sapiens | 8745 | ADAM23 | MDC-3, MDC3 | ADAM metallopeptidase domain 23 | 2q33 | 603710 |
| Homo sapiens | 81792 | ADAMTS12 | PRO4389 | ADAM metallopeptidase with thrombospondin type 1 motif, 12 | 5q35 | 606184 |
| Homo sapiens | 170690 | ADAMTS16 | ADAMTS16s | ADAM metallopeptidase with thrombospondin type 1 motif, 16 | 5p15 | 607510 |
| Homo sapiens | 11174 | ADAMTS6 | ADAM-TS 6, ADAM-TS6, ADAMTS-6 | ADAM metallopeptidase with thrombospondin type 1 motif, 6 | 5q12 | 605008 |
| Homo sapiens | 57188 | ADAMTSL3 | ADAMTSL-3 | ADAMTS-like 3 | 15q25.2 | 609199 |
| Homo sapiens | 116 | ADCYAP1 | PACAP | adenylate cyclase activating polypeptide 1 (pituitary) | 18p11 | 102980 |
| Homo sapiens | 166647 | ADGRA3 | GPR125, PGR21, TEM5L | adhesion G protein-coupled receptor A3 | 4p15.2 | 612303 |
| Homo sapiens | 23284 | ADGRL3 | CIRL3, LEC3, LPHN3 | adhesion G protein-coupled receptor L3 | 4q13.1 |  |
| Homo sapiens | 124 | ADH1A | ADH1 | alcohol dehydrogenase 1A (class I), alpha polypeptide | 4q23 | 103700 |
| Homo sapiens | 125 | ADH1B | ADH2, HEL-S-117 | alcohol dehydrogenase 1B (class I), beta polypeptide | 4q23 | 103720 |
| Homo sapiens | 126 | ADH1C | ADH3 | alcohol dehydrogenase 1C (class I), gamma polypeptide | 4q23 | 103730 |
| Homo sapiens | 127 | ADH4 | ADH-2, HEL-S-4 | alcohol dehydrogenase 4 (class II), pi polypeptide | 4q22 | 103740 |
| Homo sapiens | 128 | ADH5 | ADH-3, ADHX, FALDH, FDH, GSH-FDH, GSNOR | alcohol dehydrogenase 5 (class III), chi polypeptide | 4q23 | 103710 |
| Homo sapiens | 130 | ADH6 | ADH-5 | alcohol dehydrogenase 6 (class V) | 4q23 | 103735 |
| Homo sapiens | 131 | ADH7 | ADH4 | alcohol dehydrogenase 7 (class IV), mu or sigma polypeptide | 4q23-q24 | 600086 |
| Homo sapiens | 133 | ADM | AM, PAMP | adrenomedullin | 11p15.4 | 103275 |
| Homo sapiens | 23394 | ADNP | ADNP1, HVDAS, MRD28 | activity-dependent neuroprotector homeobox | 20q13.13 | 611386 |
| Homo sapiens | 22850 | ADNP2 | ZNF508 | ADNP homeobox 2 | 18q23 |  |
| Homo sapiens | 134 | ADORA1 | RDC7 | adenosine A1 receptor | 1q32.1 | 102775 |
| Homo sapiens | 135 | ADORA2A | A2aR, ADORA2, RDC8 | adenosine A2a receptor | 22q11.23 | 102776 |
| Homo sapiens | 148 | ADRA1A | ADRA1C, ADRA1L1, ALPHA1AAR | adrenoceptor alpha 1A | 8p21.2 | 104221 |
| Homo sapiens | 150 | ADRA2A | ADRA2, ADRA2R, ADRAR, ALPHA2AAR, ZNF32 | adrenoceptor alpha 2A | 10q25.2 | 104210 |
| Homo sapiens | 155 | ADRB3 | BETA3AR | adrenoceptor beta 3 | 8p12 | 109691 |
| Homo sapiens | 156 | ADRBK1 | BARK1, BETA-ARK1, GRK2 | adrenergic, beta, receptor kinase 1 | 11q13.1 | 109635 |
| Homo sapiens | 157 | ADRBK2 | BARK2, GRK3 | adrenergic, beta, receptor kinase 2 | 22q12.1 | 109636 |
| Homo sapiens | 159 | ADSS | ADEH 2, ADSS | adenylosuccinate synthase | 1q44 | 103060 |
| Homo sapiens | 116987 | AGAP1 | AGAP-1, CENTG2, GGAP1, cnt-g2 | ArfGAP with GTPase domain, ankyrin repeat and PH domain 1 | 2q37 | 608651 |
| Homo sapiens | 123624 | AGBL1 | CCP4, FECD8 | ATP/GTP binding protein-like 1 | 15q25.3 | 615496 |
| Homo sapiens | 177 | AGER | RAGE | advanced glycosylation end product-specific receptor | 6p21.3 | 600214 |
| Homo sapiens | 181 | AGRP | AGRT, ART, ASIP2 | agouti related neuropeptide | 16q22 | 602311 |
| Homo sapiens | 64902 | AGXT2 | AGT2, DAIBAT | alanine--glyoxylate aminotransferase 2 | 5p13 | 612471 |
| Homo sapiens | 54806 | AHI1 | AHI-1, JBTS3, ORF1, dJ71N10.1 | Abelson helper integration site 1 | 6q23.3 | 608894 |
| Homo sapiens | 203 | AK1 | HTL-S-58j | adenylate kinase 1 | 9q34.1 | 103000 |
| Homo sapiens | 207 | AKT1 | AKT, CWS6, PKB, PKB-ALPHA, PRKBA, RAC, RAC-ALPHA | v-akt murine thymoma viral oncogene homolog 1 | 14q32.32 | 164730 |
| Homo sapiens | 10000 | AKT3 | MPPH, MPPH2, PKB-GAMMA, PKBG, PRKBG, RAC-PK-gamma, RAC-gamma, STK-2 | v-akt murine thymoma viral oncogene homolog 3 | 1q44 | 611223 |
| Homo sapiens | 211 | ALAS1 | ALAS, ALAS-H, ALAS3, ALASH, MIG4 | 5'-aminolevulinate synthase 1 | 3p21.1 | 125290 |
| Homo sapiens | 213 | ALB | ANALBA, FDAH, PRO0883, PRO0903, PRO1341 | albumin | 4q13.3 | 103600 |
| Homo sapiens | 8854 | ALDH1A2 | RALDH(II), RALDH2, RALDH2-T | aldehyde dehydrogenase 1 family, member A2 | 15q21.3 | 603687 |
| Homo sapiens | 217 | ALDH2 | ALDH-E2, ALDHI, ALDM | aldehyde dehydrogenase 2 family (mitochondrial) | 12q24.2 | 100650 |
| Homo sapiens | 221 | ALDH3B1 | ALDH4, ALDH7 | aldehyde dehydrogenase 3 family, member B1 | 11q13 | 600466 |
| Homo sapiens | 226 | ALDOA | ALDA, GSD12, HEL-S-87p | aldolase A, fructose-bisphosphate | 16p11.2 | 103850 |
| Homo sapiens | 391538 | ALDOAP1 |  | aldolase A, fructose-bisphosphate pseudogene 1 | 3p21.2 |  |
| Homo sapiens | 230 | ALDOC | ALDC | aldolase C, fructose-bisphosphate | 17cen-q12 | 103870 |
| Homo sapiens | 239 | ALOX12 | 12-LOX, 12S-LOX, LOG12 | arachidonate 12-lipoxygenase | 17p13.1 | 152391 |
| Homo sapiens | 259173 | ALS2CL | RN49018 | ALS2 C-terminal like | 3p21.31 | 612402 |
| Homo sapiens | 23600 | AMACR | AMACRD, CBAS4, RACE, RM | alpha-methylacyl-CoA racemase | 5p13 | 604489 |
| Homo sapiens | 55626 | AMBRA1 | DCAF3, WDR94 | autophagy/beclin-1 regulator 1 | 11p11.2 | 611359 |
| Homo sapiens | 262 | AMD1 | ADOMETDC, AMD, SAMDC | adenosylmethionine decarboxylase 1 | 6q21 | 180980 |
| Homo sapiens | 287 | ANK2 | ANK-2, LQT4, brank-2 | ankyrin 2, neuronal | 4q25-q27 | 106410 |
| Homo sapiens | 288 | ANK3 | ANKYRIN-G, MRT37 | ankyrin 3, node of Ranvier (ankyrin G) | 10q21 | 600465 |
| Homo sapiens | 51479 | ANKFY1 | ANKHZN, BTBD23, ZFYVE14 | ankyrin repeat and FYVE domain containing 1 | 17p13.3 | 607927 |
| Homo sapiens | 255239 | ANKK1 | PKK2 | ankyrin repeat and kinase domain containing 1 | 11q23.2 | 608774 |
| Homo sapiens | 341405 | ANKRD33 | C12orf7, PANKY | ankyrin repeat domain 33 | 12q13.13 |  |
| Homo sapiens | 56899 | ANKS1B | AIDA, AIDA-1, ANKS2, EB-1, EB1, cajalin-2 | ankyrin repeat and sterile alpha motif domain containing 1B | 12q23.1 | 607815 |
| Homo sapiens | 301 | ANXA1 | ANX1, LPC1 | annexin A1 | 9q21.13 | 151690 |
| Homo sapiens | 302 | ANXA2 | ANX2, ANX2L4, CAL1H, HEL-S-270, LIP2, LPC2, LPC2D, P36, PAP-IV | annexin A2 | 15q22.2 | 151740 |
| Homo sapiens | 308 | ANXA5 | ANX5, ENX2, HEL-S-7, PP4, RPRGL3 | annexin A5 | 4q27 | 131230 |
| Homo sapiens | 310 | ANXA7 | ANX7, SNX, SYNEXIN | annexin A7 | 10q22.2 | 186360 |
| Homo sapiens | 26985 | AP3M1 |  | adaptor-related protein complex 3, mu 1 subunit | 10q22.2 | 610366 |
| Homo sapiens | 321 | APBA2 | D15S1518E, HsT16821, LIN-10, MGC:14091, MINT2, X11-BETA, X11L | amyloid beta (A4) precursor protein-binding, family A, member 2 | 15q11-q12 | 602712 |
| Homo sapiens | 324 | APC | BTPS2, DP2, DP2.5, DP3, GS, PPP1R46 | adenomatous polyposis coli | 5q21-q22 | 611731 |
| Homo sapiens | 27301 | APEX2 | APE2, APEXL2, XTH2, ZGRF2 | APEX nuclease (apurinic/apyrimidinic endonuclease) 2 | Xp11.21 | 300773 |
| Homo sapiens | 335 | APOA1 |  | apolipoprotein A-I | 11q23-q24 | 107680 |
| Homo sapiens | 347 | APOD |  | apolipoprotein D | 3q29 | 107740 |
| Homo sapiens | 348 | APOE | AD2, APO-E, LDLCQ5, LPG | apolipoprotein E | 19q13.2 | 107741 |
| Homo sapiens | 8542 | APOL1 | APO-L, APOL, APOL-I, FSGS4 | apolipoprotein L, 1 | 22q13.1 | 603743 |
| Homo sapiens | 23780 | APOL2 | APOL-II, APOL3 | apolipoprotein L, 2 | 22q12 | 607252 |
| Homo sapiens | 80832 | APOL4 | APOL-IV, APOLIV | apolipoprotein L, 4 | 22q11.2-q13.2 | 607254 |
| Homo sapiens | 367 | AR | AIS, DHTR, HUMARA, HYSP1, KD, NR3C4, SBMA, SMAX1, TFM | androgen receptor | Xq12 | 313700 |
| Homo sapiens | 93663 | ARHGAP18 | MacGAP, SENEX, bA307O14.2 | Rho GTPase activating protein 18 | 6q22.33 | 613351 |
| Homo sapiens | 57514 | ARHGAP31 | AOS1, CDGAP | Rho GTPase activating protein 31 | 3q13.33 | 610911 |
| Homo sapiens | 9743 | ARHGAP32 | GC-GAP, GRIT, PX-RICS, RICS, p200RhoGAP, p250GAP | Rho GTPase activating protein 32 | 11q24.3 | 608541 |
| Homo sapiens | 393 | ARHGAP4 | C1, RGC1, RhoGAP4, SrGAP4, p115 | Rho GTPase activating protein 4 | Xq28 | 300023 |
| Homo sapiens | 396 | ARHGDIA | GDIA1, HEL-S-47e, NPHS8, RHOGDI, RHOGDI-1 | Rho GDP dissociation inhibitor (GDI) alpha | 17q25.3 | 601925 |
| Homo sapiens | 9826 | ARHGEF11 | GTRAP48, PDZ-RHOGEF | Rho guanine nucleotide exchange factor (GEF) 11 | 1q21 | 605708 |
| Homo sapiens | 51329 | ARL6IP4 | SFRS20, SR-25, SRp25, SRrp37 | ADP-ribosylation factor-like 6 interacting protein 4 | 12q24.31 | 607668 |
| Homo sapiens | 406 | ARNTL | BMAL1, BMAL1c, JAP3, MOP3, PASD3, TIC, bHLHe5 | aryl hydrocarbon receptor nuclear translocator-like | 11p15 | 602550 |
| Homo sapiens | 421 | ARVCF |  | armadillo repeat gene deleted in velocardiofacial syndrome | 22q11.21 | 602269 |
| Homo sapiens | 57412 | AS3MT | CYT19 | arsenite methyltransferase | 10q24.32 | 611806 |
| Homo sapiens | 427 | ASAH1 | AC, ACDase, ASAH, PHP, PHP32, SMAPME | N-acylsphingosine amidohydrolase (acid ceramidase) 1 | 8p22 | 613468 |
| Homo sapiens | 23245 | ASTN2 | bA67K19.1 | astrotactin 2 | 9q33.1 | 612856 |
| Homo sapiens | 85300 | ATCAY | BNIP-H, CLAC | ataxia, cerebellar, Cayman type | 19p13.3 | 608179 |
| Homo sapiens | 468 | ATF4 | CREB-2, CREB2, TAXREB67, TXREB | activating transcription factor 4 | 22q13.1 | 604064 |
| Homo sapiens | 472 | ATM | AT1, ATA, ATC, ATD, ATDC, ATE, TEL1, TELO1 | ATM serine/threonine kinase | 11q22-q23 | 607585 |
| Homo sapiens | 488 | ATP2A2 | ATP2B, DAR, DD, SERCA2 | ATPase, Ca++ transporting, cardiac muscle, slow twitch 2 | 12q24.11 | 108740 |
| Homo sapiens | 493 | ATP2B4 | ATP2B2, MXRA1, PMCA4, PMCA4b, PMCA4x | ATPase, Ca++ transporting, plasma membrane 4 | 1q32.1 | 108732 |
| Homo sapiens | 498 | ATP5A1 | ATP5A, ATP5AL2, ATPM, COXPD22, HEL-S-123m, MC5DN4, MOM2, OMR, ORM, hATP1 | ATP synthase, H+ transporting, mitochondrial F1 complex, alpha subunit 1, cardiac muscle | 18q21 | 164360 |
| Homo sapiens | 10476 | ATP5H | ATPQ | ATP synthase, H+ transporting, mitochondrial Fo complex, subunit d | 17q25 |  |
| Homo sapiens | 55101 | ATP5SL |  | ATP5S-like | 19q13.2 |  |
| Homo sapiens | 9114 | ATP6V0D1 | ATP6D, ATP6DV, P39, VATX, VMA6, VPATPD | ATPase, H+ transporting, lysosomal 38kDa, V0 subunit d1 | 16q22.1 | 607028 |
| Homo sapiens | 523 | ATP6V1A | ATP6A11, HO68, VA68, VPP2, Vma1, ATP6V1A | ATPase, H+ transporting, lysosomal 70kDa, V1 subunit A | 3q13.31 | 607027 |
| Homo sapiens | 526 | ATP6V1B2 | ATP6B1B2, ATP6B2, HO57, VATB, VPP3, Vma2 | ATPase, H+ transporting, lysosomal 56/58kDa, V1 subunit B2 | 8p21.3 | 606939 |
| Homo sapiens | 51761 | ATP8A2 | ATP, ATPIB, CAMRQ4, IB, ML-1 | ATPase, aminophospholipid transporter, class I, type 8A, member 2 | 13q12 | 605870 |
| Homo sapiens | 6311 | ATXN2 | ASL13, ATX2, SCA2, TNRC13 | ataxin 2 | 12q24.1 | 601517 |
| Homo sapiens | 551 | AVP | ADH, ARVP-NPII, AVRP, VP, AVP | arginine vasopressin | 20p13 | 192340 |
| Homo sapiens | 567 | B2M |  | beta-2-microglobulin | 15q21.1 | 109700 |
| Homo sapiens | 27087 | B3GAT1 | CD57, GLCATP, GLCUATP, HNK1, LEU7, NK-1, NK1 | beta-1,3-glucuronyltransferase 1 | 11q25 | 151290 |
| Homo sapiens | 23621 | BACE1 | ASP2, BACE, HSPC104 | beta-site APP-cleaving enzyme 1 | 11q23.2-q23.3 | 604252 |
| Homo sapiens | 7917 | BAG6 | BAG-6, BAT3, D6S52E, G3 | BCL2-associated athanogene 6 | 6p21.3 | 142590 |
| Homo sapiens | 8314 | BAP1 | HUCEP-13, UCHL2, hucep-6 | BRCA1 associated protein-1 (ubiquitin carboxy-terminal hydrolase) | 3p21.1 | 603089 |
| Homo sapiens | 10409 | BASP1 | CAP-23, CAP23, NAP-22, NAP22 | brain abundant, membrane attached signal protein 1 | 5p15.1 | 605940 |
| Homo sapiens | 8537 | BCAS1 | AIBC1, NABC1 | breast carcinoma amplified sequence 1 | 20q13.2 | 602968 |
| Homo sapiens | 607 | BCL9 | LGS | B-cell CLL/lymphoma 9 | 1q21 | 602597 |
| Homo sapiens | 627 | BDNF | ANON2, BULN2 | brain-derived neurotrophic factor | 11p13 | 113505 |
| Homo sapiens | 8678 | BECN1 | ATG6, VPS30, beclin1 | beclin 1, autophagy related | 17q21 | 604378 |
| Homo sapiens | 23299 | BICD2 | SMALED2, bA526D8.1 | bicaudal D homolog 2 (Drosophila) | 9q22.31 | 609797 |
| Homo sapiens | 638 | BIK | BIP1, BP4, NBK | BCL2-interacting killer (apoptosis-inducing) | 22q13.31 | 603392 |
| Homo sapiens | 329 | BIRC2 | API1, HIAP2, Hiap-2, MIHB, RNF48, c-IAP1, cIAP1 | baculoviral IAP repeat containing 2 | 11q22 | 601712 |
| Homo sapiens | 2647 | BLOC1S1 | BLOS1, GCN5L1, MICoA, RT14 | biogenesis of lysosomal organelles complex-1, subunit 1 | 12q13-q14 | 601444 |
| Homo sapiens | 671 | BPI | BPIFD1, rBPI | bactericidal/permeability-increasing protein | 20q11.23 | 109195 |
| Homo sapiens | 8315 | BRAP | BRAP2, IMP, RNF52 | BRCA1 associated protein | 12q24 | 604986 |
| Homo sapiens | 23774 | BRD1 | BRL, BRPF1, BRPF2 | bromodomain containing 1 | 22q13.33 | 604589 |
| Homo sapiens | 25855 | BRMS1 |  | breast cancer metastasis suppressor 1 | 11q13.2 | 606259 |
| Homo sapiens | 7862 | BRPF1 | BR140 | bromodomain and PHD finger containing, 1 | 3p26-p25 | 602410 |
| Homo sapiens | 27154 | BRPF3 |  | bromodomain and PHD finger containing, 3 | 6p21 |  |
| Homo sapiens | 8927 | BSN | ZNF231 | bassoon presynaptic cytomatrix protein | 3p21.31 | 604020 |
| Homo sapiens | 114781 | BTBD9 | dJ322I12.1 | BTB (POZ) domain containing 9 | 6p21 | 611237 |
| Homo sapiens | 10385 | BTN2A2 | BT2.2, BTF2, BTN2.2 | butyrophilin, subfamily 2, member A2 | 6p22.1 | 613591 |
| Homo sapiens | 11119 | BTN3A1 | BT3.1, BTF5, BTN3.1, CD277 | butyrophilin, subfamily 3, member A1 | 6p22.1 | 613593 |
| Homo sapiens | 11118 | BTN3A2 | BT3.2, BTF4, BTN3.2, CD277 | butyrophilin, subfamily 3, member A2 | 6p22.1 | 613594 |
| Homo sapiens | 119032 | C10orf32 |  | chromosome 10 open reading frame 32 | 10q24.32 |  |
| Homo sapiens | 100528007 | C10orf32-ASMT | C10orf32-AS3MT | C10orf32-ASMT readthrough (NMD candidate) | 10q |  |
| Homo sapiens | 122525 | C14orf28 | DRIP-1, DRIP1, c14_5270 | chromosome 14 open reading frame 28 | 14q21.2 |  |
| Homo sapiens | 712 | C1QA |  | complement component 1, q subcomponent, A chain | 1p36.12 | 120550 |
| Homo sapiens | 713 | C1QB |  | complement component 1, q subcomponent, B chain | 1p36.12 | 120570 |
| Homo sapiens | 114899 | C1QTNF3 | C1ATNF3, CORCS, CORS, CORS-26, CORS26, CTRP3 | C1q and tumor necrosis factor related protein 3 | 5p13 | 612045 |
| Homo sapiens | 79568 | C2orf47 |  | chromosome 2 open reading frame 47 | 2q33.1 |  |
| Homo sapiens | 205327 | C2orf69 |  | chromosome 2 open reading frame 69 | 2q33.1 |  |
| Homo sapiens | 389084 | C2orf82 | UNQ830 | chromosome 2 open reading frame 82 | 2q37.1 |  |
| Homo sapiens | 389676 | C8orf87 |  | chromosome 8 open reading frame 87 | 8q22.1 |  |
| Homo sapiens | 203228 | C9orf72 | ALSFTD, FTDALS | chromosome 9 open reading frame 72 | 9p21.2 | 614260 |
| Homo sapiens | 760 | CA2 | CA-II, CAC, CAII, Car2, HEL-76 | carbonic anhydrase II | 8q22 | 611492 |
| Homo sapiens | 23523 | CABIN1 | CAIN, PPP3IN | calcineurin binding protein 1 | 22q11.23 | 604251 |
| Homo sapiens | 775 | CACNA1C | CACH2, CACN2, CACNL1A1, CCHL1A1, CaV1.2, LQT8, TS | calcium channel, voltage-dependent, L type, alpha 1C subunit | 12p13.3 | 114205 |
| Homo sapiens | 8911 | CACNA1I | Cav3.3, ca(v)3.3 | calcium channel, voltage-dependent, T type, alpha 1I subunit | 22q13.1 | 608230 |
| Homo sapiens | 781 | CACNA2D1 | CACNA2, CACNL2A, CCHL2A, LINC01112, lncRNA-N3 | calcium channel, voltage-dependent, alpha 2/delta subunit 1 | 7q21-q22 | 114204 |
| Homo sapiens | 55799 | CACNA2D3 | HSA272268 | calcium channel, voltage-dependent, alpha 2/delta subunit 3 | 3p21.1 | 606399 |
| Homo sapiens | 783 | CACNB2 | CACNLB2, CAVB2, MYSB | calcium channel, voltage-dependent, beta 2 subunit | 10p12 | 600003 |
| Homo sapiens | 10369 | CACNG2 | MRD10 | calcium channel, voltage-dependent, gamma subunit 2 | 22q13.1 | 602911 |
| Homo sapiens | 27091 | CACNG5 |  | calcium channel, voltage-dependent, gamma subunit 5 | 17q24 | 606405 |
| Homo sapiens | 57863 | CADM3 | BIgR, IGSF4B, NECL1, Necl-1, TSLL1, synCAM3 | cell adhesion molecule 3 | 1q21.2-q22 | 609743 |
| Homo sapiens | 255022 | CALHM1 | FAM26C | calcium homeostasis modulator 1 | 10q24.33 | 612234 |
| Homo sapiens | 51063 | CALHM2 | FAM26B | calcium homeostasis modulator 2 | 10q24.33 | 612235 |
| Homo sapiens | 119395 | CALHM3 | FAM26A, bA225H22.7 | calcium homeostasis modulator 3 | 10q24.33 |  |
| Homo sapiens | 801 | CALM1 | CALML2, CAMI, CPVT4, DD132, LQT14, PHKD, caM | calmodulin 1 (phosphorylase kinase, delta) | 14q32.11 | 114180 |
| Homo sapiens | 83698 | CALN1 | CABP8 | calneuron 1 | 7q11 | 607176 |
| Homo sapiens | 50632 | CALY | DRD1IP, NSG3 | calcyon neuron-specific vesicular protein | 10q26.3 | 604647 |
| Homo sapiens | 815 | CAMK2A | CAMKA | calcium/calmodulin-dependent protein kinase II alpha | 5q32 | 114078 |
| Homo sapiens | 816 | CAMK2B | CAM2, CAMK2, CAMKB | calcium/calmodulin-dependent protein kinase II beta | 7p14.3-p14.1 | 607707 |
| Homo sapiens | 10645 | CAMKK2 | CAMKK, CAMKKB | calcium/calmodulin-dependent protein kinase kinase 2, beta | 12q24.2 | 615002 |
| Homo sapiens | 100130460 | CAND1.11 |  | uncharacterized LOC100130460 | 11p15.4 |  |
| Homo sapiens | 10487 | CAP1 | CAP-PEN, CAP1 | CAP, adenylate cyclase-associated protein 1 (yeast) | 1p34.2 |  |
| Homo sapiens | 9607 | CARTPT | CART | CART prepropeptide | 5q13.2 | 602606 |
| Homo sapiens | 836 | CASP3 | CPP32, CPP32B, SCA-1 | caspase 3, apoptosis-related cysteine peptidase | 4q34 | 600636 |
| Homo sapiens | 873 | CBR1 | CBR, SDR21C1, hCBR1 | carbonyl reductase 1 | 21q22.13 | 114830 |
| Homo sapiens | 875 | CBS | HIP4 | cystathionine-beta-synthase | 21q22.3 | 613381 |
| Homo sapiens | 883 | CCBL1 | GTK, KAT1, KATI | cysteine conjugate-beta lyase, cytoplasmic | 9q34.11 | 600547 |
| Homo sapiens | 56267 | CCBL2 | KAT3, KATIII | cysteine conjugate-beta lyase 2 | 1p22.2 | 610656 |
| Homo sapiens | 160777 | CCDC60 |  | coiled-coil domain containing 60 | 12q24.23 |  |
| Homo sapiens | 80323 | CCDC68 | SE57-1 | coiled-coil domain containing 68 | 18q21 |  |
| Homo sapiens | 79080 | CCDC86 |  | coiled-coil domain containing 86 | 11q12.2 | 611293 |
| Homo sapiens | 885 | CCK |  | cholecystokinin | 3p22.1 | 118440 |
| Homo sapiens | 886 | CCKAR | CCK-A, CCK1-R, CCK1R, CCKRA | cholecystokinin A receptor | 4p15.2 | 118444 |
| Homo sapiens | 887 | CCKBR | CCK-B, CCK2R, GASR | cholecystokinin B receptor | 11p15.4 | 118445 |
| Homo sapiens | 6347 | CCL2 | GDCF-2, HC11, HSMCR30, MCAF, MCP-1, MCP1, SCYA2, SMC-CF | chemokine (C-C motif) ligand 2 | 17q11.2-q12 | 158105 |
| Homo sapiens | 1234 | CCR5 | CC-CKR-5, CCCKR5, CCR-5, CD195, CKR-5, CKR5, CMKBR5, IDDM22 | chemokine (C-C motif) receptor 5 (gene/pseudogene) | 3p21.31 | 601373 |
| Homo sapiens | 7203 | CCT3 | CCT-gamma, CCTG, PIG48, TCP-1-gamma, TRIC5 | chaperonin containing TCP1, subunit 3 (gamma) | 1q23 | 600114 |
| Homo sapiens | 940 | CD28 | Tp44 | CD28 molecule | 2q33 | 186760 |
| Homo sapiens | 962 | CD48 | BCM1, BLAST, BLAST1, MEM-102, SLAMF2, hCD48, mCD48 | CD48 molecule | 1q21.3-q22 | 109530 |
| Homo sapiens | 998 | CDC42 | CDC42Hs, G25K | cell division cycle 42 | 1p36.1 | 116952 |
| Homo sapiens | 10602 | CDC42EP3 | BORG2, CEP3, UB1 | CDC42 effector protein (Rho GTPase binding) 3 | 2p21 | 606133 |
| Homo sapiens | 56990 | CDC42SE2 | SPEC2 | CDC42 small effector 2 | 5q31.1 |  |
| Homo sapiens | 1012 | CDH13 | CDHH, P105 | cadherin 13 | 16q23.3 | 601364 |
| Homo sapiens | 8099 | CDK2AP1 | DOC1, DORC1, ST19, doc-1, p12DOC-1 | cyclin-dependent kinase 2 associated protein 1 | 12q24.31 | 602198 |
| Homo sapiens | 1020 | CDK5 | PSSALRE | cyclin-dependent kinase 5 | 7q36 | 123831 |
| Homo sapiens | 8851 | CDK5R1 | CDK5P35, CDK5R, NCK5A, p23, p25, p35, p35nck5a | cyclin-dependent kinase 5, regulatory subunit 1 (p35) | 17q11.2 | 603460 |
| Homo sapiens | 1033 | CDKN3 | CDI1, CIP2, KAP, KAP1 | cyclin-dependent kinase inhibitor 3 | 14q22 | 123832 |
| Homo sapiens | 90273 | CEACAM21 | CEACAM3, R29124_1 | carcinoembryonic antigen-related cell adhesion molecule 21 | 19q13.2 |  |
| Homo sapiens | 10153 | CEBPZ | CBF, CBF2, HSP-CBF, NOC1 | CCAAT/enhancer binding protein (C/EBP), zeta | 2p22.2 | 612828 |
| Homo sapiens | 56853 | CELF4 | BRUNOL-4, BRUNOL4 | CUGBP, Elav-like family member 4 | 18q12 | 612679 |
| Homo sapiens | 60680 | CELF5 | BRUNOL-5, BRUNOL5, CELF-5 | CUGBP, Elav-like family member 5 | 19p13 | 612680 |
| Homo sapiens | 9620 | CELSR1 | ADGRC1, CDHF9, FMI2, HFMI2, ME2 | cadherin, EGF LAG seven-pass G-type receptor 1 | 22q13.3 | 604523 |
| Homo sapiens | 9859 | CEP170 | FAM68A, KAB, KIAA0470 | centrosomal protein 170kDa | 1q44 | 613023 |
| Homo sapiens | 375298 | CERKL | RP26 | ceramide kinase-like | 2q31.3 | 608381 |
| Homo sapiens | 1072 | CFL1 | CFL, HEL-S-15 | cofilin 1 (non-muscle) | 11q13 | 601442 |
| Homo sapiens | 84952 | CGNL1 | JACOP | cingulin-like 1 | 15q21.3 | 607856 |
| Homo sapiens | 1113 | CHGA | CGA | chromogranin A | 14q32 | 118910 |
| Homo sapiens | 1114 | CHGB | SCG1 | chromogranin B | 20p12.3 | 118920 |
| Homo sapiens | 1116 | CHI3L1 | ASRT7, CGP-39, GP-39, GP39, HC-gp39, HCGP-3P, YKL-40, YKL40, YYL-40, hCGP-39 | chitinase 3-like 1 (cartilage glycoprotein-39) | 1q32.1 | 601525 |
| Homo sapiens | 10752 | CHL1 | CALL, L1CAM2 | cell adhesion molecule L1-like | 3p26.1 | 607416 |
| Homo sapiens | 1124 | CHN2 | ARHGAP3, BCH-3, RHOGAP3, CHN2 | chimerin 2 | 7p15.3 | 602857 |
| Homo sapiens | 11261 | CHP1 | CHP, SLC9A1BP, Sid470p, p22, p24 | calcineurin-like EF-hand protein 1 | 15q13.3 | 606988 |
| Homo sapiens | 89832 | CHRFAM7A | CHRNA7, CHRNA7-DR1, D-10 | CHRNA7 (cholinergic receptor, nicotinic, alpha 7, exons 5-10) and FAM7A (family with sequence similarity 7A, exons A-E) fusion | 15q13.1 | 609756 |
| Homo sapiens | 1128 | CHRM1 | HM1, M1, M1R | cholinergic receptor, muscarinic 1 | 11q13 | 118510 |
| Homo sapiens | 1132 | CHRM4 | HM4, M4R | cholinergic receptor, muscarinic 4 | 11p12-p11.2 | 118495 |
| Homo sapiens | 1133 | CHRM5 | HM5 | cholinergic receptor, muscarinic 5 | 15q26 | 118496 |
| Homo sapiens | 1136 | CHRNA3 | LNCR2, NACHRA3, PAOD2 | cholinergic receptor, nicotinic, alpha 3 (neuronal) | 15q24 | 118503 |
| Homo sapiens | 1137 | CHRNA4 | BFNC, EBN, EBN1, NACHR, NACHRA4, NACRA4 | cholinergic receptor, nicotinic, alpha 4 (neuronal) | 20q13.33 | 118504 |
| Homo sapiens | 1138 | CHRNA5 | LNCR2 | cholinergic receptor, nicotinic, alpha 5 (neuronal) | 15q24 | 118505 |
| Homo sapiens | 1139 | CHRNA7 | CHRNA7-2, NACHRA7 | cholinergic receptor, nicotinic, alpha 7 (neuronal) | 15q14 | 118511 |
| Homo sapiens | 1141 | CHRNB2 | EFNL3, nAChRB2 | cholinergic receptor, nicotinic, beta 2 (neuronal) | 1q21.3 | 118507 |
| Homo sapiens | 64377 | CHST8 | GALNAC4ST1, GalNAc4ST, PSS3 | carbohydrate (N-acetylgalactosamine 4-0) sulfotransferase 8 | 19q13.1 | 610190 |
| Homo sapiens | 148113 | CILP2 | CLIP-2 | cartilage intermediate layer protein 2 | 19p13.11 | 612419 |
| Homo sapiens | 11113 | CIT | CRIK, STK21 | citron rho-interacting serine/threonine kinase | 12q24 | 605629 |
| Homo sapiens | 1152 | CKB | B-CK, BCKB, HEL-211, HEL-S-29, CKB | creatine kinase, brain | 14q32 | 123280 |
| Homo sapiens | 1178 | CLC | GAL10, Gal-10, LGALS10, LGALS10A, LPPL_HUMAN | Charcot-Leyden crystal galectin | 19q13.1 | 153310 |
| Homo sapiens | 9076 | CLDN1 | CLD1, ILVASC, SEMP1 | claudin 1 | 3q28-q29 | 603718 |
| Homo sapiens | 7122 | CLDN5 | AWAL, BEC1, CPETRL1, TMVCF | claudin 5 | 22q11.21 | 602101 |
| Homo sapiens | 9685 | CLINT1 | CLINT, ENTH, EPN4, EPNR | clathrin interactor 1 | 5q33.3 | 607265 |
| Homo sapiens | 9575 | CLOCK | KAT13D, bHLHe8 | clock circadian regulator | 4q12 | 601851 |
| Homo sapiens | 1212 | CLTB | LCB | clathrin, light chain B | 5q35 | 118970 |
| Homo sapiens | 1191 | CLU | AAG4, APO-J, APOJ, CLI1, CLU2, KUB1, NA1/NA2, SGP-2, SGP2, SP-40, TRPM-2, TRPM2, CLU | clusterin | 8p21-p12 | 185430 |
| Homo sapiens | 1240 | CMKLR1 | CHEMERINR, ChemR23, DEZ, RVER1 | chemerin chemokine-like receptor 1 | 12q24.1 | 602351 |
| Homo sapiens | 202333 | CMYA5 | C5orf10, SPRYD2, TRIM76 | cardiomyopathy associated 5 | 5q14.1 | 612193 |
| Homo sapiens | 10175 | CNIH1 | CNIH, CNIH-1, CNIL, TGAM77 | cornichon family AMPA receptor auxiliary protein 1 | 14q22.2 | 611287 |
| Homo sapiens | 254263 | CNIH2 | CNIH-2, Cnil | cornichon family AMPA receptor auxiliary protein 2 | 11q13.2 | 611288 |
| Homo sapiens | 149111 | CNIH3 | CNIH-3 | cornichon family AMPA receptor auxiliary protein 3 | 1q42.12 |  |
| Homo sapiens | 54805 | CNNM2 | ACDP2 | cyclin and CBS domain divalent metal cation transport mediator 2 | 10q24.32 | 607803 |
| Homo sapiens | 1267 | CNP | CNP1 | 2',3'-cyclic nucleotide 3' phosphodiesterase | 17q21 | 123830 |
| Homo sapiens | 1268 | CNR1 | CANN6, CB-R, CB1, CB1A, CB1K5, CB1R, CNR | cannabinoid receptor 1 (brain) | 6q14-q15 | 114610 |
| Homo sapiens | 1269 | CNR2 | CB-2, CB2, CX5 | cannabinoid receptor 2 (macrophage) | 1p36.11 | 605051 |
| Homo sapiens | 1270 | CNTF | HCNTF | ciliary neurotrophic factor | 11q12.2 | 118945 |
| Homo sapiens | 53942 | CNTN5 | HNB-2s, NB-2 | contactin 5 | 11q22.1 | 607219 |
| Homo sapiens | 26047 | CNTNAP2 | AUTS15, CASPR2, CDFE, NRXN4, PTHSL1 | contactin associated protein-like 2 | 7q35 | 604569 |
| Homo sapiens | 129684 | CNTNAP5 | caspr5 | contactin associated protein-like 5 | 2q14.3 | 610519 |
| Homo sapiens | 340267 | COL28A1 | COL28 | collagen, type XXVIII, alpha 1 | 7p21.3 | 609996 |
| Homo sapiens | 1284 | COL4A2 | ICH, POREN2 | collagen, type IV, alpha 2 | 13q34 | 120090 |
| Homo sapiens | 51397 | COMMD10 | PTD002 | COMM domain containing 10 | 5q23.1 |  |
| Homo sapiens | 1312 | COMT | HEL-S-98n | catechol-O-methyltransferase | 22q11.21 | 116790 |
| Homo sapiens | 10814 | CPLX2 | 921-L, CPX-2, CPX2, Hfb1 | complexin 2 | 5q35.2 | 605033 |
| Homo sapiens | 221184 | CPNE2 | COPN2, CPN2 | copine II | 16q13 | 604206 |
| Homo sapiens | 8895 | CPNE3 | CPN3, PRO1071 | copine III | 8q21.3 | 604207 |
| Homo sapiens | 1385 | CREB1 | CREB | cAMP responsive element binding protein 1 | 2q34 | 123810 |
| Homo sapiens | 1387 | CREBBP | CBP, KAT3A, RSTS | CREB binding protein | 16p13.3 | 600140 |
| Homo sapiens | 1390 | CREM | CREM-2, ICER, hCREM-2 | cAMP responsive element modulator | 10p11.21 | 123812 |
| Homo sapiens | 1400 | CRMP1 | CRMP-1, DPYSL1, DRP-1, DRP1, ULIP-3 | collapsin response mediator protein 1 | 4p16.1 | 602462 |
| Homo sapiens | 1401 | CRP | PTX1 | C-reactive protein, pentraxin-related | 1q23.2 | 123260 |
| Homo sapiens | 1407 | CRY1 | PHLL1 | cryptochrome circadian clock 1 | 12q23-q24.1 | 601933 |
| Homo sapiens | 1410 | CRYAB | CMD1II, CRYA2, CTPP2, CTRCT16, HEL-S-101, HSPB5, MFM2 | crystallin, alpha B | 11q22.3-q23.1 | 123590 |
| Homo sapiens | 1413 | CRYBA4 | CTRCT23, MCOPCT4 | crystallin, beta A4 | 22q12.1 | 123631 |
| Homo sapiens | 1414 | CRYBB1 | CATCN3, CTRCT17 | crystallin, beta B1 | 22q12.1 | 600929 |
| Homo sapiens | 1428 | CRYM | DFNA40, THBP | crystallin, mu | 16p12.2 | 123740 |
| Homo sapiens | 1438 | CSF2RA | CD116, CDw116, CSF2RX, CSF2RAY, CSF2RX, CSF2RY, GM-CSF-R-alpha, GMCSFR, GMR, SMDP4, CSF2RA | colony stimulating factor 2 receptor, alpha, low-affinity (granulocyte-macrophage) | Xp22.32 and Yp11.3 |  |
| Homo sapiens | 1439 | CSF2RB | CD131, CDw131, IL3RB, IL5RB, SMDP5 | colony stimulating factor 2 receptor, beta, low-affinity (granulocyte-macrophage) | 22q13.1 | 138981 |
| Homo sapiens | 64478 | CSMD1 | PPP1R24 | CUB and Sushi multiple domains 1 | 8p23.2 | 608397 |
| Homo sapiens | 114784 | CSMD2 | dJ1007G16.1, dJ1007G16.2, dJ947L8.1 | CUB and Sushi multiple domains 2 | 1p34.3 | 608398 |
| Homo sapiens | 1454 | CSNK1E | CKIepsilon, HCKIE | casein kinase 1, epsilon | 22q13.1 | 600863 |
| Homo sapiens | 1457 | CSNK2A1 | CK2A1, CKII, CSNK2A3 | casein kinase 2, alpha 1 polypeptide | 20p13 | 115440 |
| Homo sapiens | 10675 | CSPG5 | NGC | chondroitin sulfate proteoglycan 5 (neuroglycan C) | 3p21.3 | 606775 |
| Homo sapiens | 101928630 | CTC-436P18.1 |  | uncharacterized LOC101928630 | 5q12.1 |  |
| Homo sapiens | 9150 | CTDP1 | CCFDN, FCP1 | CTD (carboxy-terminal domain, RNA polymerase II, polypeptide A) phosphatase, subunit 1 | 18q23 | 604927 |
| Homo sapiens | 1493 | CTLA4 | ALPS5, CD, CD152, CELIAC3, CTLA-4, GRD4, GSE, IDDM12 | cytotoxic T-lymphocyte-associated protein 4 | 2q33 | 123890 |
| Homo sapiens | 1496 | CTNNA2 | CAP-R, CAPR, CT114, CTNR | catenin (cadherin-associated protein), alpha 2 | 2p12-p11.1 | 114025 |
| Homo sapiens | 29119 | CTNNA3 | ARVD13, VR22 | catenin (cadherin-associated protein), alpha 3 | 10q22.2 | 607667 |
| Homo sapiens | 1499 | CTNNB1 | CTNNB, MRD19, armadillo | catenin (cadherin-associated protein), beta 1, 88kDa | 3p21 | 116806 |
| Homo sapiens | 1501 | CTNND2 | GT24, NPRAP | catenin (cadherin-associated protein), delta 2 | 5p15.2 | 604275 |
| Homo sapiens | 613212 | CTXN3 | KABE | cortexin 3 | 5q23.2 |  |
| Homo sapiens | 6387 | CXCL12 | IRH, PBSF, SCYB12, SDF1, TLSF, TPAR1 | chemokine (C-X-C motif) ligand 12 | 10q11.1 | 600835 |
| Homo sapiens | 3576 | CXCL8 | GCP-1, GCP1, IL8, LECT, LUCT, LYNAP, MDNCF, MONAP, NAF, NAP-1, NAP1 | chemokine (C-X-C motif) ligand 8 | 4q13-q21 | 146930 |
| Homo sapiens | 124936 | CYB5D2 |  | cytochrome b5 domain containing 2 | 17p13.2 |  |
| Homo sapiens | 1536 | CYBB | AMCBX2, CGD, GP91-1, GP91-PHOX, GP91PHOX, IMD34, NOX2, p91-PHOX | cytochrome b-245, beta polypeptide | Xp21.1 | 300481 |
| Homo sapiens | 349391 | CYCSP44 | HCP44 | cytochrome c, somatic pseudogene 44 | Xq27.3 |  |
| Homo sapiens | 1586 | CYP17A1 | CPT7, CYP17, P450C17, S17AH | cytochrome P450, family 17, subfamily A, polypeptide 1 | 10q24.3 | 609300 |
| Homo sapiens | 1544 | CYP1A2 | CP12, P3-450, P450(PA) | cytochrome P450, family 1, subfamily A, polypeptide 2 | 15q24.1 | 124060 |
| Homo sapiens | 1565 | CYP2D6 | CPD6, CYP2D, CYP2D7AP, CYP2D7BP, CYP2D7P2, CYP2D8P2, CYP2DL1, CYPIID6, P450-DB1, P450C2D, P450DB1 | cytochrome P450, family 2, subfamily D, polypeptide 6 | 22q13.1 | 124030 |
| Homo sapiens | 1571 | CYP2E1 | CPE1, CYP2E, P450-J, P450C2E | cytochrome P450, family 2, subfamily E, polypeptide 1 | 10q26.3 | 124040 |
| Homo sapiens | 1576 | CYP3A4 | CP33, CP34, CYP3A, CYP3A3, CYPIIIA3, CYPIIIA4, HLP, NF-25, P450C3, P450PCN1 | cytochrome P450, family 3, subfamily A, polypeptide 4 | 7q21.1 | 124010 |
| Homo sapiens | 1577 | CYP3A5 | CP35, CYPIIIA5, P450PCN3, PCN3 | cytochrome P450, family 3, subfamily A, polypeptide 5 | 7q21.1 | 605325 |
| Homo sapiens | 1610 | DAO | DAAO, DAMOX, OXDA | D-amino-acid oxidase | 12q24 | 124050 |
| Homo sapiens | 267012 | DAOA | LG72, SG72 | D-amino acid oxidase activator | 13q34 | 607408 |
| Homo sapiens | 282706 | DAOA-AS1 | DAOA-AS, DAOAAS, G30 | DAOA antisense RNA 1 | 13q34 | 607415 |
| Homo sapiens | 1621 | DBH | DBM | dopamine beta-hydroxylase (dopamine beta-monooxygenase) | 9q34 | 609312 |
| Homo sapiens | 1622 | DBI | ACBD1, ACBP, CCK-RP, EP | diazepam binding inhibitor (GABA receptor modulator, acyl-CoA binding protein) | 2q12-q21 | 125950 |
| Homo sapiens | 55827 | DCAF6 | 1200006M05Rik, ARCAP, IQWD1, MSTP055, NRIP, PC326 | DDB1 and CUL4 associated factor 6 | 1q24.2 | 610494 |
| Homo sapiens | 1630 | DCC | CRC18, CRCR1, IGDCC1, MRMV1, NTN1R1 | DCC netrin 1 receptor | 18q21.3 | 120470 |
| Homo sapiens | 51473 | DCDC2 | DCDC2A, NPHP19, RU2, RU2S | doublecortin domain containing 2 | 6p22.1 | 605755 |
| Homo sapiens | 1641 | DCX | DBCN, DC, LISX, SCLH, XLIS | doublecortin | Xq22.3-q23 | 300121 |
| Homo sapiens | 23576 | DDAH1 | DDAH, HEL-S-16 | dimethylarginine dimethylaminohydrolase 1 | 1p22 | 604743 |
| Homo sapiens | 1644 | DDC | AADC | dopa decarboxylase (aromatic L-amino acid decarboxylase) | 7p12.2 | 107930 |
| Homo sapiens | 8528 | DDO | DASOX-1, DDO-2, DDO | D-aspartate oxidase | 6q21 | 124450 |
| Homo sapiens | 780 | DDR1 | CAK, CD167, DDR, EDDR1, HGK2, MCK10, NEP, NTRK4, PTK3, PTK3A, RTK6, TRKE | discoidin domain receptor tyrosine kinase 1 | 6p21.3 | 600408 |
| Homo sapiens | 100188856 | DEL22Q11.2 | C22DDELSC22DELq11.2 | Chromosome 22q11.2 deletion syndrome, distal | 22q11.2 | 611867 |
| Homo sapiens | 1674 | DES | CSM1, CSM2, LGMD2R | desmin | 2q35 | 125660 |
| Homo sapiens | 9993 | DGCR2 | DGS-C, IDD, LAN, SEZ-12 | DiGeorge syndrome critical region gene 2 | 22q11.21 | 600594 |
| Homo sapiens | 8214 | DGCR6 |  | DiGeorge syndrome critical region gene 6 | 22q11 | 601279 |
| Homo sapiens | 160851 | DGKH | DGKeta | diacylglycerol kinase, eta | 13q14.11 | 604071 |
| Homo sapiens | 27185 | DISC1 | C1orf136, SCZD9 | disrupted in schizophrenia 1 | 1q42.1 | 605210 |
| Homo sapiens | 27184 | DISC2 | DISC1-AS1, DISC1OS, NCRNA00015 | disrupted in schizophrenia 2 (non-protein coding) | 1q42.1 | 606271 |
| Homo sapiens | 85458 | DIXDC1 | CCD1 | DIX domain containing 1 | 11q23.1 | 610493 |
| Homo sapiens | 27121 | DKK4 | DKK-4 | dickkopf WNT signaling pathway inhibitor 4 | 8p11.2-p11.1 | 605417 |
| Homo sapiens | 10395 | DLC1 | ARHGAP7, HP, STARD12, p122-RhoGAP | DLC1 Rho GTPase activating protein | 8p22 | 604258 |
| Homo sapiens | 1738 | DLD | DLDDH, E3, GCSL, LAD, PHE3, DLD | dihydrolipoamide dehydrogenase | 7q31-q32 | 238331 |
| Homo sapiens | 1739 | DLG1 | DLGH1, SAP-97, SAP97, dJ1061C18.1.1, hdlg | discs, large homolog 1 (Drosophila) | 3q29 | 601014 |
| Homo sapiens | 1741 | DLG3 | MRX, MRX90, NEDLG, PPP1R82, SAP102, XLMR | discs, large homolog 3 (Drosophila) | Xq13.1 | 300189 |
| Homo sapiens | 1742 | DLG4 | PSD95, SAP-90, SAP90 | discs, large homolog 4 (Drosophila) | 17p13.1 | 602887 |
| Homo sapiens | 9229 | DLGAP1 | DAP-1, DAP-1-ALPHA, DAP-1-BETA, DAP1A, DLGAP1B, GKAP, SAPAP1, hGKAP, DLGAP1 | discs, large (Drosophila) homolog-associated protein 1 | 18p11.31 | 605445 |
| Homo sapiens | 9228 | DLGAP2 | DAP2, SAPAP2 | discs, large (Drosophila) homolog-associated protein 2 | 8p23 | 605438 |
| Homo sapiens | 58512 | DLGAP3 | DAP3, SAPAP3 | discs, large (Drosophila) homolog-associated protein 3 | 1p35.3-p34.1 | 611413 |
| Homo sapiens | 1756 | DMD | BMD, CMD3B, DXS142, DXS164, DXS206, DXS230, DXS239, DXS268, DXS269, DXS270, DXS272, MRX85 | dystrophin | Xp21.2 | 300377 |
| Homo sapiens | 25981 | DNAH1 | DNAHC1, HDHC7, HL-11, HL11, HSRF-1, XLHSRF-1 | dynein, axonemal, heavy chain 1 | 3p21.1 | 603332 |
| Homo sapiens | 3337 | DNAJB1 | HSPF1, Hdj1, Hsp40, RSPH16B, Sis1 | DnaJ (Hsp40) homolog, subfamily B, member 1 | 19p13.2 | 604572 |
| Homo sapiens | 9829 | DNAJC6 | DJC6, PARK19 | DnaJ (Hsp40) homolog, subfamily C, member 6 | 1p31.3 | 608375 |
| Homo sapiens | 23234 | DNAJC9 | HDJC9, JDD1, SB73 | DnaJ (Hsp40) homolog, subfamily C, member 9 | 10q22.2 | 611206 |
| Homo sapiens | 1759 | DNM1 | DNM | dynamin 1 | 9q34 | 602377 |
| Homo sapiens | 1786 | DNMT1 | ADCADN, AIM, CXXC9, DNMT, HSN1E, MCMT | DNA (cytosine-5-)-methyltransferase 1 | 19p13.2 | 126375 |
| Homo sapiens | 1789 | DNMT3B | ICF, ICF1, M.HsaIIIB | DNA (cytosine-5-)-methyltransferase 3 beta | 20q11.2 | 602900 |
| Homo sapiens | 9732 | DOCK4 |  | dedicator of cytokinesis 4 | 7q31.1 | 607679 |
| Homo sapiens | 57572 | DOCK6 | AOS2, ZIR1 | dedicator of cytokinesis 6 | 19p13.2 | 614194 |
| Homo sapiens | 1806 | DPYD | DHP, DHPDHASE, DPD | dihydropyrimidine dehydrogenase | 1p22 | 612779 |
| Homo sapiens | 1808 | DPYSL2 | CRMP-2, CRMP2, DHPRP2, DRP-2, DRP2, N2A3, ULIP-2, ULIP2 | dihydropyrimidinase-like 2 | 8p22-p21 | 602463 |
| Homo sapiens | 1812 | DRD1 | DADRA, DRD1 | dopamine receptor D1 | 5q35.1 | 126449 |
| Homo sapiens | 1813 | DRD2 | D2DR, D2R | dopamine receptor D2 | 11q23 | 126450 |
| Homo sapiens | 1814 | DRD3 | D3DR, ETM1, FET1 | dopamine receptor D3 | 3q13.3 | 126451 |
| Homo sapiens | 1815 | DRD4 | D4DR | dopamine receptor D4 | 11p15.5 | 126452 |
| Homo sapiens | 1816 | DRD5 | DBDR, DRD1B, DRD1L2 | dopamine receptor D5 | 4p16.1 | 126453 |
| Homo sapiens | 84062 | DTNBP1 | BLOC1S8, DBND, HPS7, My031, SDY | dystrobrevin binding protein 1 | 6p22.3 | 607145 |
| Homo sapiens | 1848 | DUSP6 | HH19, MKP3, PYST1 | dual specificity phosphatase 6 | 12q22-q23 | 602748 |
| Homo sapiens | 54808 | DYM | DMC, SMC | dymeclin | 18q21.1 | 607461 |
| Homo sapiens | 79180 | EFHD2 | SWS1 | EF-hand domain family, member D2 | 1p36.21 |  |
| Homo sapiens | 1946 | EFNA5 | AF1, EFL5, EPLG7, GLC1M, LERK7, RAGS | ephrin-A5 | 5q21 | 601535 |
| Homo sapiens | 1948 | EFNB2 | EPLG5, HTKL, Htk-L, LERK5 | ephrin-B2 | 13q33 | 600527 |
| Homo sapiens | 1950 | EGF | HOMG4, URG | epidermal growth factor | 4q25 | 131530 |
| Homo sapiens | 1956 | EGFR | ERBB, ERBB1, HER1, NISBD2, PIG61, mENA | epidermal growth factor receptor | 7p12 | 131550 |
| Homo sapiens | 1959 | EGR2 | AT591, CMT1D, CMT4E, KROX20 | early growth response 2 | 10q21.1 | 129010 |
| Homo sapiens | 1960 | EGR3 | EGR-3, PILOT | early growth response 3 | 8p23-p21 | 602419 |
| Homo sapiens | 1961 | EGR4 | NGFI-C, NGFIC, PAT133 | early growth response 4 | 2p13 | 128992 |
| Homo sapiens | 26298 | EHF | ESE3, ESE3B, ESEJ | ets homologous factor | 11p12 | 605439 |
| Homo sapiens | 1974 | EIF4A2 | BM-010, DDX2B, EIF4A, EIF4F, eIF-4A-II, eIF4A-II | eukaryotic translation initiation factor 4A2 | 3q28 | 601102 |
| Homo sapiens | 54898 | ELOVL2 | SSC2 | ELOVL fatty acid elongase 2 | 6p24.2 | 611814 |
| Homo sapiens | 2019 | EN1 |  | engrailed homeobox 1 | 2q14.2 | 131290 |
| Homo sapiens | 55740 | ENAH | ENA, MENA, NDPP1 | enabled homolog (Drosophila) | 1q42.12 | 609061 |
| Homo sapiens | 2026 | ENO2 | HEL-S-279, NSE | enolase 2 (gamma, neuronal) | 12p13 | 131360 |
| Homo sapiens | 9583 | ENTPD4 | LALP70, LAP70, LYSAL1, NTPDase-4, UDPase | ectonucleoside triphosphate diphosphohydrolase 4 | 8p21.3 | 607577 |
| Homo sapiens | 2036 | EPB41L1 | 4.1N, MRD11 | erythrocyte membrane protein band 4.1-like 1 | 20q11.2-q12 | 602879 |
| Homo sapiens | 23136 | EPB41L3 | 4.1B, DAL-1, DAL1 | erythrocyte membrane protein band 4.1-like 3 | 18p11.32 | 605331 |
| Homo sapiens | 2047 | EPHB1 | ELK, EPHT2, Hek6, NET | EPH receptor B1 | 3q21-q23 | 600600 |
| Homo sapiens | 2065 | ERBB3 | ErbB-3, HER3, LCCS2, MDA-BF-1, c-erbB-3, c-erbB3, erbB3-S, p180-ErbB3, p45-sErbB3, p85-sErbB3 | erb-b2 receptor tyrosine kinase 3 | 12q13 | 190151 |
| Homo sapiens | 2066 | ERBB4 | ALS19, HER4, p180erbB4 | erb-b2 receptor tyrosine kinase 4 | 2q33.3-q34 | 600543 |
| Homo sapiens | 26059 | ERC2 | CAST, CAST1, ELKSL, SPBC110, Spc110 | ELKS/RAB6-interacting/CAST family member 2 | 3p14.3 |  |
| Homo sapiens | 10613 | ERLIN1 | C10orf69, Erlin-1, KE04, KEO4, SPFH1, SPG62 | ER lipid raft associated 1 | 10q24.31 | 611604 |
| Homo sapiens | 57471 | ERMN | JN, KIAA1189, ermin | ermin, ERM-like protein | 2q24.1 | 610072 |
| Homo sapiens | 30816 | ERVW-1 | ENV, ENVW, ERVWE1, HERV-7q, HERV-W-ENV, HERV7Q, HERVW, HERVWENV | endogenous retrovirus group W, member 1 | 7q21.2 | 604659 |
| Homo sapiens | 2099 | ESR1 | ER, ESR, ESRA, ESTRR, Era, NR3A1 | estrogen receptor 1 | 6q25.1 | 133430 |
| Homo sapiens | 2100 | ESR2 | ER-BETA, ESR-BETA, ESRB, ESTRB, Erb, NR3A2 | estrogen receptor 2 (ER beta) | 14q23.2 | 601663 |
| Homo sapiens | 55770 | EXOC2 | SEC5, SEC5L1, Sec5p | exocyst complex component 2 | 6p25.3 | 615329 |
| Homo sapiens | 2170 | FABP3 | FABP11, H-FABP, M-FABP, MDGI, O-FABP | fatty acid binding protein 3, muscle and heart | 1p33-p32 | 134651 |
| Homo sapiens | 2171 | FABP5 | E-FABP, EFABP, KFABP, PA-FABP, PAFABP | fatty acid binding protein 5 (psoriasis-associated) | 8q21.13 | 605168 |
| Homo sapiens | 2173 | FABP7 | B-FABP, BLBP, FABPB, LTR2-FABP7, MRG | fatty acid binding protein 7, brain | 6q22-q23 | 602965 |
| Homo sapiens | 3992 | FADS1 | D5D, FADS6, FADSD5, LLCDL1, TU12 | fatty acid desaturase 1 | 11q12.2-q13.1 | 606148 |
| Homo sapiens | 9415 | FADS2 | D6D, DES6, FADSD6, LLCDL2, SLL0262, TU13 | fatty acid desaturase 2 | 11q12.2 | 606149 |
| Homo sapiens | 90362 | FAM110B | C8orf72 | family with sequence similarity 110, member B | 8q12.1 | 611394 |
| Homo sapiens | 388650 | FAM69A |  | family with sequence similarity 69, member A | 1p22.1 | 614542 |
| Homo sapiens | 145773 | FAM81A |  | family with sequence similarity 81, member A | 15q22.2 |  |
| Homo sapiens | 22909 | FAN1 | KIAA1018, KMIN, MTMR15, hFAN1 | FANCD2/FANCI-associated nuclease 1 | 15q13.2-q13.3 | 613534 |
| Homo sapiens | 2193 | FARSA | CML33, FARSL, FARSLA, FRSA, PheHA | phenylalanyl-tRNA synthetase, alpha subunit | 19p13.2 | 602918 |
| Homo sapiens | 355 | FAS | ALPS1A, APO-1, APT1, CD951, FASTM, TNFRSF6, FAS | Fas cell surface death receptor | 10q24.1 | 134637 |
| Homo sapiens | 26223 | FBXL21 | FBL3B, FBXL3B, FBXL3P, Fbl21 | F-box and leucine-rich repeat protein 21 (gene/pseudogene) | 5q31 | 609087 |
| Homo sapiens | 80204 | FBXO11 | FBX11, PRMT9, UBR6, UG063H01, VIT1 | F-box protein 11 | 2p16.3 | 607871 |
| Homo sapiens | 200933 | FBXO45 | Fbx45 | F-box protein 45 | 3q29 | 609112 |
| Homo sapiens | 2212 | FCGR2A | CD32, CD32A, CDw32, FCG2, FCGR21, FcGR, IGFR2, FCGR2A | Fc fragment of IgG, low affinity IIa, receptor (CD32) | 1q23 | 146790 |
| Homo sapiens | 2214 | FCGR3A | CD16, CD16A, FCG3, FCGR3, FCGRIII, FCR-10, FCRIII, FCRIIIA, IGFR3, IMD20 | Fc fragment of IgG, low affinity IIIa, receptor (CD16a) | 1q23 | 146740 |
| Homo sapiens | 9638 | FEZ1 |  | fasciculation and elongation protein zeta 1 (zygin I) | 11q24.2 | 604825 |
| Homo sapiens | 2263 | FGFR2 | BBDS, BEK, BFR-1, CD332, CEK3, CFD1, ECT1, JWS, K-SAM, KGFR, TK14, TK25 | fibroblast growth factor receptor 2 | 10q26 | 176943 |
| Homo sapiens | 9457 | FHL5 | ACT, dJ393D12.2 | four and a half LIM domains 5 | 6q16.1-q16.3 | 605126 |
| Homo sapiens | 55137 | FIGN |  | fidgetin | 2q24.3 | 605295 |
| Homo sapiens | 2332 | FMR1 | FMRP, FRAXA, POF, POF1 | fragile X mental retardation 1 | Xq27.3 | 309550 |
| Homo sapiens | 2346 | FOLH1 | FGCP, FOLH, GCP2, GCPII, NAALAD1, NAALAdase, PSM, PSMA, mGCP | folate hydrolase (prostate-specific membrane antigen) 1 | 11p11.2 | 600934 |
| Homo sapiens | 93986 | FOXP2 | CAGH44, SPCH1, TNRC10 | forkhead box P2 | 7q31 | 605317 |
| Homo sapiens | 348751 | FTCDNL1 | FONG | formiminotransferase cyclodeaminase N-terminal like | 2q33.1 | 614308 |
| Homo sapiens | 2512 | FTL | LFTD, NBIA3 | ferritin, light polypeptide | 19q13.33 | 134790 |
| Homo sapiens | 79068 | FTO | ALKBH9 | fat mass and obesity associated | 16q12.2 | 610966 |
| Homo sapiens | 29960 | FTSJ2 | FJH1, HEL97, MRM2, RRMJ2 | FtsJ RNA methyltransferase homolog 2 (E. coli) | 7p22 | 606906 |
| Homo sapiens | 8880 | FUBP1 | FBP, FUBP, hDH V | far upstream element (FUSE) binding protein 1 | 1p31.1 | 603444 |
| Homo sapiens | 8087 | FXR1 | FXR1P | fragile X mental retardation, autosomal homolog 1 | 3q28 | 600819 |
| Homo sapiens | 53826 | FXYD6 |  | FXYD domain containing ion transport regulator 6 | 11q23.3 | 606683 |
| Homo sapiens | 2534 | FYN | SLK, SYN, p59-FYN | FYN proto-oncogene, Src family tyrosine kinase | 6q21 | 137025 |
| Homo sapiens | 7976 | FZD3 | Fz-3 | frizzled class receptor 3 | 8p21 | 606143 |
| Homo sapiens | 2550 | GABBR1 | GABABR1-3, GB1, GPRC3A, dJ271M21.1.1, dJ271M21.1.2, GABBR1 | gamma-aminobutyric acid (GABA) B receptor, 1 | 6p21.31 | 603540 |
| Homo sapiens | 9568 | GABBR2 | GABABR2, GPR51, GPRC3B, HG20, HRIHFB2099 | gamma-aminobutyric acid (GABA) B receptor, 2 | 9q22.1-q22.3 | 607340 |
| Homo sapiens | 2554 | GABRA1 | ECA4, EIEE19, EJM, EJM5 | gamma-aminobutyric acid (GABA) A receptor, alpha 1 | 5q34 | 137160 |
| Homo sapiens | 2555 | GABRA2 |  | gamma-aminobutyric acid (GABA) A receptor, alpha 2 | 4p12 | 137140 |
| Homo sapiens | 2556 | GABRA3 |  | gamma-aminobutyric acid (GABA) A receptor, alpha 3 | Xq28 | 305660 |
| Homo sapiens | 2557 | GABRA4 |  | gamma-aminobutyric acid (GABA) A receptor, alpha 4 | 4p12 | 137141 |
| Homo sapiens | 2558 | GABRA5 |  | gamma-aminobutyric acid (GABA) A receptor, alpha 5 | 15q12 | 137142 |
| Homo sapiens | 2559 | GABRA6 |  | gamma-aminobutyric acid (GABA) A receptor, alpha 6 | 5q34 | 137143 |
| Homo sapiens | 2560 | GABRB1 |  | gamma-aminobutyric acid (GABA) A receptor, beta 1 | 4p12 | 137190 |
| Homo sapiens | 2561 | GABRB2 |  | gamma-aminobutyric acid (GABA) A receptor, beta 2 | 5q34 | 600232 |
| Homo sapiens | 2562 | GABRB3 | ECA5 | gamma-aminobutyric acid (GABA) A receptor, beta 3 | 15q12 | 137192 |
| Homo sapiens | 2563 | GABRD | EIG10, EJM7, GEFSP5 | gamma-aminobutyric acid (GABA) A receptor, delta | 1p36.3 | 137163 |
| Homo sapiens | 2564 | GABRE |  | gamma-aminobutyric acid (GABA) A receptor, epsilon | Xq28 | 300093 |
| Homo sapiens | 2566 | GABRG2 | CAE2, ECA2, GEFSP3 | gamma-aminobutyric acid (GABA) A receptor, gamma 2 | 5q34 | 137164 |
| Homo sapiens | 2569 | GABRR1 |  | gamma-aminobutyric acid (GABA) A receptor, rho 1 | 6q15 | 137161 |
| Homo sapiens | 2571 | GAD1 | CPSQ1, GAD, SCP | glutamate decarboxylase 1 (brain, 67kDa) | 2q31 | 605363 |
| Homo sapiens | 2572 | GAD2 | GAD65 | glutamate decarboxylase 2 (pancreatic islets and brain, 65kDa) | 10p11.23 | 138275 |
| Homo sapiens | 2596 | GAP43 | B-50, PP46 | growth associated protein 43 | 3q13.31 | 162060 |
| Homo sapiens | 2597 | GAPDH | G3PD, GAPD, HEL-S-162eP | glyceraldehyde-3-phosphate dehydrogenase | 12p13 | 138400 |
| Homo sapiens | 54815 | GATAD2A | p66alpha | GATA zinc finger domain containing 2A | 19p13.11 | 614997 |
| Homo sapiens | 2643 | GCH1 | DYT14, DYT5, DYT5a, GCH, GTP-CH-1, GTPCH1, HPABH4B | GTP cyclohydrolase 1 | 14q22.1-q22.2 | 600225 |
| Homo sapiens | 2729 | GCLC | GCL, GCS, GLCL, GLCLC | glutamate-cysteine ligase, catalytic subunit | 6p12 | 606857 |
| Homo sapiens | 2730 | GCLM | GLCLR | glutamate-cysteine ligase, modifier subunit | 1p22.1 | 601176 |
| Homo sapiens | 9615 | GDA | CYPIN, GUANASE, NEDASIN | guanine deaminase | 9q21.13 | 139260 |
| Homo sapiens | 2664 | GDI1 | 1A, GDIL, MRX41, MRX48, OPHN2, RABGD1A, RABGDIA, XAP-4 | GDP dissociation inhibitor 1 | Xq28 | 300104 |
| Homo sapiens | 2668 | GDNF | ATF1, ATF2, HFB1-GDNF, HSCR3 | glial cell derived neurotrophic factor | 5p13.1-p12 | 600837 |
| Homo sapiens | 2670 | GFAP | ALXDRD | glial fibrillary acidic protein | 17q21 | 137780 |
| Homo sapiens | 2674 | GFRA1 | GDNFR, GDNFRA, GFR-ALPHA-1, RET1L, RETL1, TRNR1 | GDNF family receptor alpha 1 | 10q26.11 | 601496 |
| Homo sapiens | 2675 | GFRA2 | GDNFRB, NRTNR-ALPHA, NTNRA, RETL2, TRNR2 | GDNF family receptor alpha 2 | 8p21.3 | 601956 |
| Homo sapiens | 2676 | GFRA3 | GDNFR3 | GDNF family receptor alpha 3 | 5q31.1-q31.3 | 605710 |
| Homo sapiens | 51738 | GHRL | MTLRP | ghrelin/obestatin prepropeptide | 3p26-p25 | 605353 |
| Homo sapiens | 26058 | GIGYF2 | GYF2, PARK11, PERQ2, PERQ3, TNRC15 | GRB10 interacting GYF protein 2 | 2q37.1 | 612003 |
| Homo sapiens | 2701 | GJA4 | CX37 | gap junction protein, alpha 4, 37kDa | 1p35.1 | 121012 |
| Homo sapiens | 2703 | GJA8 | CAE, CAE1, CTRCT1, CX50, CZP1, MP70 | gap junction protein, alpha 8, 50kDa | 1q21.1 | 600897 |
| Homo sapiens | 57369 | GJD2 | CX36, GJA9 | gap junction protein, delta 2, 36kDa | 15q14 | 607058 |
| Homo sapiens | 2739 | GLO1 | GLOD1, GLYI, HEL-S-74 | glyoxalase I | 6p21.3-p21.1 | 138750 |
| Homo sapiens | 55830 | GLT8D1 | AD-017, MSTP139 | glycosyltransferase 8 domain containing 1 | 3p21.1 |  |
| Homo sapiens | 2746 | GLUD1 | GDH, GDH1, GLUD | glutamate dehydrogenase 1 | 10q23.3 | 138130 |
| Homo sapiens | 2752 | GLUL | GLNS, GS, PIG43, PIG59 | glutamate-ammonia ligase | 1q31 | 138290 |
| Homo sapiens | 132158 | GLYCTK | HBEBP2, HBEBP4, HBeAgBP4A | glycerate kinase | 3p21.1 | 610516 |
| Homo sapiens | 51291 | GMIP | ARHGAP46 | GEM interacting protein | 19p13.11 | 609694 |
| Homo sapiens | 2774 | GNAL | DYT25 | guanine nucleotide binding protein (G protein), alpha activating activity polypeptide, olfactory type | 18p11.22-p11.21 | 139312 |
| Homo sapiens | 2778 | GNAS | AHO, C20orf451, GPSA, GSA, GSP, NESP, PHP1A, PHP1B, PHP1C, POH, SgVI, GNAS | GNAS complex locus | 20q13.3 | 139320 |
| Homo sapiens | 2782 | GNB1 |  | guanine nucleotide binding protein (G protein), beta polypeptide 1 | 1p36.33 | 139380 |
| Homo sapiens | 54584 | GNB1L | DGCRK3, FKSG1, GY2, WDR14, WDVCF | guanine nucleotide binding protein (G protein), beta polypeptide 1-like | 22q11.2 | 610778 |
| Homo sapiens | 2783 | GNB2 |  | guanine nucleotide binding protein (G protein), beta polypeptide 2 | 7q22 | 139390 |
| Homo sapiens | 10399 | GNB2L1 | Gnb2-rs1, H12.3, HLC-7, PIG21, RACK1 | guanine nucleotide binding protein (G protein), beta polypeptide 2-like 1 | 5q35.3 | 176981 |
| Homo sapiens | 2784 | GNB3 |  | guanine nucleotide binding protein (G protein), beta polypeptide 3 | 12p13 | 139130 |
| Homo sapiens | 26354 | GNL3 | C77032, E2IG3, NNP47, NS | guanine nucleotide binding protein-like 3 (nucleolar) | 3p21.1 | 608011 |
| Homo sapiens | 8443 | GNPAT | DAP-AT, DAPAT, DHAPAT | glyceronephosphate O-acyltransferase | 1q42 | 602744 |
| Homo sapiens | 64083 | GOLPH3 | GOPP1, GPP34, MIDAS, Vps74 | golgi phosphoprotein 3 (coat-protein) | 5p13.3 | 612207 |
| Homo sapiens | 2817 | GPC1 | glypican | glypican 1 | 2q35-q37 | 600395 |
| Homo sapiens | 10082 | GPC6 | OMIMD1 | glypican 6 | 13q32 | 604404 |
| Homo sapiens | 10243 | GPHN | GEPH, GPH, GPHRYN, HKPX1, MOCODC | gephyrin | 14q23.3 | 603930 |
| Homo sapiens | 2823 | GPM6A | GPM6, M6A | glycoprotein M6A | 4q34 | 601275 |
| Homo sapiens | 2825 | GPR1 |  | G protein-coupled receptor 1 | 2q33.3 | 600239 |
| Homo sapiens | 7107 | GPR137B | TM7SF1 | G protein-coupled receptor 137B | 1q42-q43 | 604658 |
| Homo sapiens | 101669764 | GPR1-AS | GPR1-AS1, GPR1AS | GPR1 antisense RNA | 2q33.3 |  |
| Homo sapiens | 9248 | GPR50 | H9, Mel1c | G protein-coupled receptor 50 | Xq28 | 300207 |
| Homo sapiens | 27201 | GPR78 |  | G protein-coupled receptor 78 | 4p16.1 | 606921 |
| Homo sapiens | 54329 | GPR85 | SREB, SREB2 | G protein-coupled receptor 85 | 7q31 | 605188 |
| Homo sapiens | 2876 | GPX1 | GPXD, GSHPX1 | glutathione peroxidase 1 | 3p21.3 | 138320 |
| Homo sapiens | 2885 | GRB2 | ASH, EGFRBP-GRB2, Grb3-3, MST084, MSTP084, NCKAP2 | growth factor receptor-bound protein 2 | 17q24-q25 | 108355 |
| Homo sapiens | 2890 | GRIA1 | GLUH1, GLUR1, GLURA, GluA1, HBGR1 | glutamate receptor, ionotropic, AMPA 1 | 5q31.1 | 138248 |
| Homo sapiens | 2891 | GRIA2 | GLUR2, GLURB, GluA2, GluR-K2, HBGR2 | glutamate receptor, ionotropic, AMPA 2 | 4q32.1 | 138247 |
| Homo sapiens | 2892 | GRIA3 | GLUR-C, GLUR-K3, GLUR3, GLURC, GluA3, MRX94 | glutamate receptor, ionotropic, AMPA 3 | Xq25 | 305915 |
| Homo sapiens | 2893 | GRIA4 | GLUR4, GLUR4C, GLURD, GluA4 | glutamate receptor, ionotropic, AMPA 4 | 11q22 | 138246 |
| Homo sapiens | 2894 | GRID1 | GluD1 | glutamate receptor, ionotropic, delta 1 | 10q22 | 610659 |
| Homo sapiens | 2897 | GRIK1 | EAA3, EEA3, GLR5, GLUR5, GluK1 | glutamate receptor, ionotropic, kainate 1 | 21q22.11 | 138245 |
| Homo sapiens | 2898 | GRIK2 | EAA4, GLR6, GLUK6, GLUR6, GluK2, MRT6 | glutamate receptor, ionotropic, kainate 2 | 6q16.3 | 138244 |
| Homo sapiens | 2899 | GRIK3 | EAA5, GLR7, GLUR7, GluK3, GluR7a | glutamate receptor, ionotropic, kainate 3 | 1p34.3 | 138243 |
| Homo sapiens | 2900 | GRIK4 | EAA1, GRIK, GluK4, KA1 | glutamate receptor, ionotropic, kainate 4 | 11q22.3 | 600282 |
| Homo sapiens | 2902 | GRIN1 | GluN1, MRD8, NMD-R1, NMDA1, NMDAR1, NR1 | glutamate receptor, ionotropic, N-methyl D-aspartate 1 | 9q34.3 | 138249 |
| Homo sapiens | 2903 | GRIN2A | EPND, FESD, GluN2A, LKS, NMDAR2A, NR2A | glutamate receptor, ionotropic, N-methyl D-aspartate 2A | 16p13.2 | 138253 |
| Homo sapiens | 2904 | GRIN2B | EIEE27, GluN2B, MRD6, NMDAR2B, NR2B, hNR3 | glutamate receptor, ionotropic, N-methyl D-aspartate 2B | 12p12 | 138252 |
| Homo sapiens | 2905 | GRIN2C | GluN2C, NMDAR2C, NR2C | glutamate receptor, ionotropic, N-methyl D-aspartate 2C | 17q25.1 | 138254 |
| Homo sapiens | 2906 | GRIN2D | EB11, GluN2D, NMDAR2D, NR2D | glutamate receptor, ionotropic, N-methyl D-aspartate 2D | 19q13.33 | 602717 |
| Homo sapiens | 116443 | GRIN3A | GluN3A, NMDAR-L, NR3A | glutamate receptor, ionotropic, N-methyl-D-aspartate 3A | 9q31.1 | 606650 |
| Homo sapiens | 23426 | GRIP1 | GRIP | glutamate receptor interacting protein 1 | 12q14.3 | 604597 |
| Homo sapiens | 2869 | GRK5 | GPRK5 | G protein-coupled receptor kinase 5 | 10q26.11 | 600870 |
| Homo sapiens | 2870 | GRK6 | GPRK6 | G protein-coupled receptor kinase 6 | 5q35 | 600869 |
| Homo sapiens | 2911 | GRM1 | GPRC1A, MGLU1, MGLUR1, PPP1R85, SCAR13 | glutamate receptor, metabotropic 1 | 6q24 | 604473 |
| Homo sapiens | 2912 | GRM2 | GLUR2, GPRC1B, MGLUR2, mGlu2 | glutamate receptor, metabotropic 2 | 3p21.2 | 604099 |
| Homo sapiens | 2913 | GRM3 | GLUR3, GPRC1C, MGLUR3, mGlu3 | glutamate receptor, metabotropic 3 | 7q21.1-q21.2 | 601115 |
| Homo sapiens | 2915 | GRM5 | GPRC1E, MGLUR5, PPP1R86, mGlu5 | glutamate receptor, metabotropic 5 | 11q14.3 | 604102 |
| Homo sapiens | 2917 | GRM7 | GLUR7, GPRC1G, MGLU7, MGLUR7, PPP1R87 | glutamate receptor, metabotropic 7 | 3p26.1-p25.1 | 604101 |
| Homo sapiens | 2896 | GRN | CLN11, GEP, GP88, PCDGF, PEPI, PGRN | granulin | 17q21.32 | 138945 |
| Homo sapiens | 2932 | GSK3B |  | glycogen synthase kinase 3 beta | 3q13.3 | 605004 |
| Homo sapiens | 2935 | GSPT1 | 551G9.2, ETF3A, GST1, eRF3a | G1 to S phase transition 1 | 16p13.1 | 139259 |
| Homo sapiens | 2937 | GSS | GSHS, HEL-S-64p, HEL-S-88n | glutathione synthetase | 20q11.2 | 601002 |
| Homo sapiens | 2938 | GSTA1 | GST2-1, GTH1, GSTA1 | glutathione S-transferase alpha 1 | 6p12.1 | 138359 |
| Homo sapiens | 2944 | GSTM1 | GST1-1, GSTM1a-1a, GSTM1b-1b, GTH4, GTM1, H-B, MU, MU-1, GSTM1 | glutathione S-transferase mu 1 | 1p13.3 | 138350 |
| Homo sapiens | 2950 | GSTP1 | DFN7, FAEES3, GST3, GSTP, HEL-S-22, PI | glutathione S-transferase pi 1 | 11q13 | 134660 |
| Homo sapiens | 2952 | GSTT1 |  | glutathione S-transferase theta 1 | 22q11.23 | 600436 |
| Homo sapiens | 2953 | GSTT2 |  | glutathione S-transferase theta 2 (gene/pseudogene) | 22q11.23 | 600437 |
| Homo sapiens | 2954 | GSTZ1 | GSTZ1-1, MAAI, MAI | glutathione S-transferase zeta 1 | 14q24.3 | 603758 |
| Homo sapiens | 2977 | GUCY1A2 | GC-SA2, GUC1A2 | guanylate cyclase 1, soluble, alpha 2 | 11q21-q22 | 601244 |
| Homo sapiens | 51454 | GULP1 | CED-6, CED6, GULP | GULP, engulfment adaptor PTB domain containing 1 | 2q32.3-q33 | 608165 |
| Homo sapiens | 60484 | HAPLN2 | BRAL1 | hyaluronan and proteoglycan link protein 2 | 1q23.1 |  |
| Homo sapiens | 404037 | HAPLN4 | BRAL2 | hyaluronan and proteoglycan link protein 4 | 19p13.1 |  |
| Homo sapiens | 768096 | HAR1A | HAR1F, LINC00064, NCRNA00064 | highly accelerated region 1A (non-protein coding) | 20q13.33 | 610556 |
| Homo sapiens | 23438 | HARS2 | HARSL, HARSR, HO3, PRLTS2 | histidyl-tRNA synthetase 2, mitochondrial | 5q31.3 | 600783 |
| Homo sapiens | 338442 | HCAR2 | GPR109A, HCA2, HM74a, HM74b, NIACR1, PUMAG, Puma-g | hydroxycarboxylic acid receptor 2 | 12q24.31 | 609163 |
| Homo sapiens | 8843 | HCAR3 | GPR109B, HCA3, HM74, PUMAG, Puma-g | hydroxycarboxylic acid receptor 3 | 12q24.31 | 606039 |
| Homo sapiens | 414764 | HCG23 | dJ1077I5.3 | HLA complex group 23 (non-protein coding) | 6p21 |  |
| Homo sapiens | 3061 | HCRTR1 | OX1R | hypocretin (orexin) receptor 1 | 1p33 | 602392 |
| Homo sapiens | 3062 | HCRTR2 | OX2R | hypocretin (orexin) receptor 2 | 6p12 | 602393 |
| Homo sapiens | 3065 | HDAC1 | GON-10, HD1, RPD3, RPD3L1 | histone deacetylase 1 | 1p34 | 601241 |
| Homo sapiens | 3066 | HDAC2 | HD2, RPD3, YAF1 | histone deacetylase 2 | 6q21 | 605164 |
| Homo sapiens | 8841 | HDAC3 | HD3, RPD3, RPD3-2 | histone deacetylase 3 | 5q31 | 605166 |
| Homo sapiens | 9759 | HDAC4 | AHO3, BDMR, HA6116, HD4, HDAC-4, HDAC-A, HDACA | histone deacetylase 4 | 2q37.3 | 605314 |
| Homo sapiens | 3077 | HFE | HFE1, HH, HLA-H, MVCD7, TFQTL2 | hemochromatosis | 6p21.3 | 613609 |
| Homo sapiens | 55733 | HHAT | MART2, SKI1, Skn | hedgehog acyltransferase | 1q32 | 605743 |
| Homo sapiens | 3094 | HINT1 | HINT, NMAN, PKCI-1, PRKCNH1 | histidine triad nucleotide binding protein 1 | 5q31.2 | 601314 |
| Homo sapiens | 8969 | HIST1H2AG | H2A.1b, H2A/p, H2AFP, H2AG, pH2A/f | histone cluster 1, H2ag | 6p22.1 | 615012 |
| Homo sapiens | 8970 | HIST1H2BJ | H2B/r, H2BFR, H2BJ | histone cluster 1, H2bj | 6p22.1 | 615044 |
| Homo sapiens | 8353 | HIST1H3E | H3.1, H3/d, H3FD | histone cluster 1, H3e | 6p22.2 | 602813 |
| Homo sapiens | 3098 | HK1 | HK1-ta-tb, HK1-tc, HKD, HKI, HMSNR, HXK1, HK1 | hexokinase 1 | 10q22 | 142600 |
| Homo sapiens | 3105 | HLA-A | HLAA | major histocompatibility complex, class I, A | 6p21.3 | 142800 |
| Homo sapiens | 3106 | HLA-B | AS, HLAB, SPDA1 | major histocompatibility complex, class I, B | 6p21.3 | 142830 |
| Homo sapiens | 3107 | HLA-C | D6S204, HLA-JY3, HLC-C, PSORS1 | major histocompatibility complex, class I, C | 6p21.3 | 142840 |
| Homo sapiens | 3117 | HLA-DQA1 | CD, CELIAC1, DQ-A1, GSE, HLA-DQA | major histocompatibility complex, class II, DQ alpha 1 | 6p21.3 | 146880 |
| Homo sapiens | 3119 | HLA-DQB1 | CELIAC1, HLA-DQB, IDDM1 | major histocompatibility complex, class II, DQ beta 1 | 6p21.3 | 604305 |
| Homo sapiens | 3123 | HLA-DRB1 | DRB1, DRw10, HLA-DR1B, HLA-DRB, SS1 | major histocompatibility complex, class II, DR beta 1 | 6p21.3 | 142857 |
| Homo sapiens | 3125 | HLA-DRB3 | HLA-DR3B | major histocompatibility complex, class II, DR beta 3 | 6p21.3 | 612735 |
| Homo sapiens | 3132 | HLA-DRB9 | D6S206, D6S206E, HLA-DR1BL, HLA-DRB1L | major histocompatibility complex, class II, DR beta 9 (pseudogene) | 6p21.3 |  |
| Homo sapiens | 3133 | HLA-E | EA1.2, EA2.1, HLA-6.2, MHC, QA1 | major histocompatibility complex, class I, E | 6p21.3 | 143010 |
| Homo sapiens | 3159 | HMGA1 | HMG-RA, HMGIY, HMGA1 | high mobility group AT-hook 1 | 6p21 | 600701 |
| Homo sapiens | 3162 | HMOX1 | HMOX1D, HO-1, HSP32, bK286B10 | heme oxygenase 1 | 22q13.1 | 141250 |
| Homo sapiens | 10949 | HNRNPA0 | HNRPA0 | heterogeneous nuclear ribonucleoprotein A0 | 5q31 | 609409 |
| Homo sapiens | 3181 | HNRNPA2B1 | HNRNPA2, HNRNPB1, HNRPA2, HNRPA2B1, HNRPB1, IBMPFD2, RNPA2, SNRPB1 | heterogeneous nuclear ribonucleoprotein A2/B1 | 7p15 | 600124 |
| Homo sapiens | 3183 | HNRNPC | C1, C2, HNRNP, HNRPC, SNRPC | heterogeneous nuclear ribonucleoprotein C (C1/C2) | 14q11.2 | 164020 |
| Homo sapiens | 3190 | HNRNPK | CSBP, HNRPK, TUNP | heterogeneous nuclear ribonucleoprotein K | 9q21.32-q21.33 | 600712 |
| Homo sapiens | 3192 | HNRNPU | HNRPU, SAF-A, SAFA, U21.1, hnRNP U | heterogeneous nuclear ribonucleoprotein U (scaffold attachment factor A) | 1q44 | 602869 |
| Homo sapiens | 9456 | HOMER1 | HOMERA, HOMER1B, HOMER1C, SYN47, Ves-1, HOMER1 | homer scaffolding protein 1 | 5q14.2 | 604798 |
| Homo sapiens | 9455 | HOMER2 | ACPD, CPD, HOMER-2, VESL-2 | homer scaffolding protein 2 | 15q24.3 | 604799 |
| Homo sapiens | 50809 | HP1BP3 | HP1-BP74, HP1BP74 | heterochromatin protein 1, binding protein 3 | 1p36.12 | 616072 |
| Homo sapiens | 89781 | HPS4 | LE | Hermansky-Pudlak syndrome 4 | 22cen-q12.3 | 606682 |
| Homo sapiens | 3269 | HRH1 | H1-R, H1R, HH1R, hisH1 | histamine receptor H1 | 3p25 | 600167 |
| Homo sapiens | 222537 | HS3ST5 | 3-OST-5, 3OST5, HS3OST5, NBLA04021 | heparan sulfate (glucosamine) 3-O-sulfotransferase 5 | 6q21 | 609407 |
| Homo sapiens | 3028 | HSD17B10 | 17b-HSD10, ABAD, CAMR, DUPXp11.22, ERAB, HADH2, HCD2, MHBD, MRPP2, MRX17, MRX31, MRXS10, SCHAD, SDR5C1 | hydroxysteroid (17-beta) dehydrogenase 10 | Xp11.2 | 300256 |
| Homo sapiens | 3320 | HSP90AA1 | EL52, HSP86, HSP89A, HSP90A, HSP90N, HSPC1, HSPCA, HSPCAL1, HSPCAL4, HSPN, Hsp89, Hsp90, LAP-2, LAP2 | heat shock protein 90kDa alpha (cytosolic), class A member 1 | 14q32.33 | 140571 |
| Homo sapiens | 259217 | HSPA12A |  | heat shock 70kDa protein 12A | 10q26.12 | 610701 |
| Homo sapiens | 3303 | HSPA1A | HEL-S-103, HSP70-1, HSP70-1A, HSP70I, HSP72, HSPA1 | heat shock 70kDa protein 1A | 6p21.3 | 140550 |
| Homo sapiens | 3304 | HSPA1B | HSP70-1B, HSP70-2 | heat shock 70kDa protein 1B | 6p21.3 | 603012 |
| Homo sapiens | 3305 | HSPA1L | HSP70-1L, HSP70-HOM, HSP70T, hum70t | heat shock 70kDa protein 1-like | 6p21.3 | 140559 |
| Homo sapiens | 3309 | HSPA5 | BIP, GRP78, HEL-S-89n, MIF2 | heat shock 70kDa protein 5 (glucose-regulated protein, 78kDa) | 9q33.3 | 138120 |
| Homo sapiens | 3310 | HSPA6 |  | heat shock 70kDa protein 6 (HSP70B') | 1q23 | 140555 |
| Homo sapiens | 3312 | HSPA8 | HEL-33, HEL-S-72p, HSC54, HSC70, HSC71, HSP71, HSP73, HSPA10, LAP-1, LAP1, NIP71 | heat shock 70kDa protein 8 | 11q24.1 | 600816 |
| Homo sapiens | 3313 | HSPA9 | CRP40, CSA, GRP-75, GRP75, HEL-S-124mB, MOT, MOT2, MTHSP75, PBP74, HSPA9 | heat shock 70kDa protein 9 (mortalin) | 5q31.1 | 600548 |
| Homo sapiens | 3329 | HSPD1 | CPN60, GROEL, HLD4, HSP-60, HSP60, HSP65, HuCHA60, SPG13 | heat shock 60kDa protein 1 (chaperonin) | 2q33.1 | 118190 |
| Homo sapiens | 3350 | HTR1A | 5-HT-1A, 5-HT1A, 5HT1a, ADRB2RL1, ADRBRL1, G-21, PFMCD | 5-hydroxytryptamine (serotonin) receptor 1A, G protein-coupled | 5q11.2-q13 | 109760 |
| Homo sapiens | 3351 | HTR1B | 5-HT1B, 5-HT1DB, HTR1D2, HTR1DB, S12 | 5-hydroxytryptamine (serotonin) receptor 1B, G protein-coupled | 6q13 | 182131 |
| Homo sapiens | 3356 | HTR2A | 5-HT2A, HTR2 | 5-hydroxytryptamine (serotonin) receptor 2A, G protein-coupled | 13q14-q21 | 182135 |
| Homo sapiens | 3358 | HTR2C | 5-HT1C, 5-HT2C, 5-HTR2C, 5HTR2C, HTR1C | 5-hydroxytryptamine (serotonin) receptor 2C, G protein-coupled | Xq24 | 312861 |
| Homo sapiens | 3359 | HTR3A | 5-HT-3, 5-HT3A, 5-HT3R, 5HT3R, HTR3 | 5-hydroxytryptamine (serotonin) receptor 3A, ionotropic | 11q23.1 | 182139 |
| Homo sapiens | 9177 | HTR3B | 5-HT3B | 5-hydroxytryptamine (serotonin) receptor 3B, ionotropic | 11q23.1 | 604654 |
| Homo sapiens | 285242 | HTR3E | 5-HT3-E, 5-HT3E, 5-HT3c1 | 5-hydroxytryptamine (serotonin) receptor 3E, ionotropic | 3q27.1 | 610123 |
| Homo sapiens | 3360 | HTR4 | 5-HT4, 5-HT4R | 5-hydroxytryptamine (serotonin) receptor 4, G protein-coupled | 5q31-q33 | 602164 |
| Homo sapiens | 3362 | HTR6 | 5-HT6, 5-HT6R | 5-hydroxytryptamine (serotonin) receptor 6, G protein-coupled | 1p36-p35 | 601109 |
| Homo sapiens | 3363 | HTR7 | 5-HT7 | 5-hydroxytryptamine (serotonin) receptor 7, adenylate cyclase-coupled | 10q21-q24 | 182137 |
| Homo sapiens | 30811 | HUNK |  | hormonally up-regulated Neu-associated kinase | 21q22.1 | 606532 |
| Homo sapiens | 3383 | ICAM1 | BB2, CD54, P3.58 | intercellular adhesion molecule 1 | 19p13.3-p13.2 | 147840 |
| Homo sapiens | 3620 | IDO1 | IDO, IDO-1, INDO | indoleamine 2,3-dioxygenase 1 | 8p12-p11 | 147435 |
| Homo sapiens | 10964 | IFI44L | C1orf29, GS3686 | interferon-induced protein 44-like | 1p31.1 | 613975 |
| Homo sapiens | 8519 | IFITM1 | 9-27, CD225, DSPA2a, IFI17, LEU13 | interferon induced transmembrane protein 1 | 11p15.5 | 604456 |
| Homo sapiens | 3458 | IFNG | IFG, IFI | interferon, gamma | 12q14 | 147570 |
| Homo sapiens | 282617 | IFNL3 | IL-28B, IL28B, IL28C | interferon, lambda 3 | 19q13.13 | 607402 |
| Homo sapiens | 8100 | IFT88 | D13S1056E, DAF19, TG737, TTC10, hTg737 | intraflagellar transport 88 | 13q12.1 | 600595 |
| Homo sapiens | 3479 | IGF1 | IGF-IA, IGFI, IGF1 | insulin-like growth factor 1 (somatomedin C) | 12q23.2 | 147440 |
| Homo sapiens | 10644 | IGF2BP2 | IMP-2, IMP2, VICKZ2 | insulin-like growth factor 2 mRNA binding protein 2 | 3q27.2 | 608289 |
| Homo sapiens | 3492 | IGH | IGD1.1@, IGH@, IGHD@, IGHDY1, IGHJ, IGHJ@, IGHV, IGHV@, IGH | immunoglobulin heavy locus | 14q32.33 |  |
| Homo sapiens | 3586 | IL10 | CSIF, GVHDS, IL-10A, TGIF, IL10 | interleukin 10 | 1q31-q32 | 124092 |
| Homo sapiens | 3587 | IL10RA | CD210, CD210a, CDW210A, HIL-10R, IL-10R1, IL10R | interleukin 10 receptor, alpha | 11q23 | 146933 |
| Homo sapiens | 3605 | IL17A | CTLA8, IL-17, IL-17A, IL17 | interleukin 17A | 6p12 | 603149 |
| Homo sapiens | 3606 | IL18 | IGIF, IL-18, IL-1g, IL1F4 | interleukin 18 | 11q22.2-q22.3 | 600953 |
| Homo sapiens | 10068 | IL18BP | IL18BPa | interleukin 18 binding protein | 11q13 | 604113 |
| Homo sapiens | 29949 | IL19 | IL-10C, MDA1, NG.1, ZMDA1 | interleukin 19 | 1q32.2 | 605687 |
| Homo sapiens | 3552 | IL1A | IL-1A, IL1, IL1-ALPHA, IL1F1 | interleukin 1, alpha | 2q14 | 147760 |
| Homo sapiens | 3553 | IL1B | IL-1, IL1-BETA, IL1F2 | interleukin 1, beta | 2q14 | 147720 |
| Homo sapiens | 3557 | IL1RN | DIRA, ICIL-1RA, IL-1RN, IL-1ra, IL-1ra3, IL1F3, IL1RA, IRAP, MVCD4 | interleukin 1 receptor antagonist | 2q14.2 | 147679 |
| Homo sapiens | 3558 | IL2 | IL-2, TCGF, lymphokine | interleukin 2 | 4q26-q27 | 147680 |
| Homo sapiens | 59067 | IL21 | CVID11, IL-21, Za11 | interleukin 21 | 4q26-q27 | 605384 |
| Homo sapiens | 3561 | IL2RG | CD132, CIDX, IL-2RG, IMD4, P64, SCIDX, SCIDX1 | interleukin 2 receptor, gamma | Xq13.1 | 308380 |
| Homo sapiens | 3563 | IL3RA | CD123, IL3RY, IL3RX, IL3RY, hIL-3Ra, IL3RA | interleukin 3 receptor, alpha (low affinity) | Xp22.3 or Yp11.3 |  |
| Homo sapiens | 3565 | IL4 | BCGF-1, BCGF1, BSF-1, BSF1, IL-4 | interleukin 4 | 5q31.1 | 147780 |
| Homo sapiens | 3569 | IL6 | BSF2, HGF, HSF, IFNB2, IL-6 | interleukin 6 | 7p21 | 147620 |
| Homo sapiens | 3570 | IL6R | CD126, IL-6R-1, IL-6RA, IL6QA, IL6RQ, gp80, IL6R | interleukin 6 receptor | 1q21 | 147880 |
| Homo sapiens | 9118 | INA | NEF5, NF-66, TXBP-1 | internexin neuronal intermediate filament protein, alpha | 10q24.33 | 605338 |
| Homo sapiens | 3635 | INPP5D | SHIP, SHIP-1, SHIP1, SIP-145, hp51CN, p150Ship | inositol polyphosphate-5-phosphatase, 145kDa | 2q37.1 | 601582 |
| Homo sapiens | 3638 | INSIG1 | CL-6, CL6 | insulin induced gene 1 | 7q36 | 602055 |
| Homo sapiens | 51141 | INSIG2 |  | insulin induced gene 2 | 2q14.2 | 608660 |
| Homo sapiens | 3843 | IPO5 | IMB3, KPNB3, Pse1, RANBP5, imp5 | importin 5 | 13q32.2 | 602008 |
| Homo sapiens | 359948 | IRF2BP2 |  | interferon regulatory factor 2 binding protein 2 | 1q42.3 | 615332 |
| Homo sapiens | 8660 | IRS2 | IRS-2 | insulin receptor substrate 2 | 13q34 | 600797 |
| Homo sapiens | 442338 | IRS3P | IRS3L | insulin receptor substrate 3, pseudogene | 7q22.1 |  |
| Homo sapiens | 8471 | IRS4 | IRS-4, PY160 | insulin receptor substrate 4 | Xq22.3 | 300904 |
| Homo sapiens | 3674 | ITGA2B | BDPLT16, BDPLT2, CD41, CD41B, GP2B, GPIIb, GT, GTA, HPA3, PPP1R93 | integrin, alpha 2b (platelet glycoprotein IIb of IIb/IIIa complex, antigen CD41) | 17q21.32 | 607759 |
| Homo sapiens | 8516 | ITGA8 |  | integrin, alpha 8 | 10p13 | 604063 |
| Homo sapiens | 3690 | ITGB3 | BDPLT16, BDPLT2, CD61, GP3A, GPIIIa, GT | integrin, beta 3 (platelet glycoprotein IIIa, antigen CD61) | 17q21.32 | 173470 |
| Homo sapiens | 3697 | ITIH1 | H1P, IATIH, IGHEP1, ITI-HC1, ITIH, SHAP | inter-alpha-trypsin inhibitor heavy chain 1 | 3p21.1 | 147270 |
| Homo sapiens | 3699 | ITIH3 | H3P | inter-alpha-trypsin inhibitor heavy chain 3 | 3p21.1 | 146650 |
| Homo sapiens | 3700 | ITIH4 | GP120, H4P, IHRP, ITI-HC4, ITIHL1, PK-120, PK120 | inter-alpha-trypsin inhibitor heavy chain family, member 4 | 3p21.1 | 600564 |
| Homo sapiens | 55600 | ITLN1 | HL-1, HL1, INTL, ITLN, LFR, hIntL, omentin | intelectin 1 (galactofuranose binding) | 1q21.3 | 609873 |
| Homo sapiens | 50618 | ITSN2 | PRO2015, SH3D1B, SH3P18, SWA, SWAP | intersectin 2 | 2p23.3 | 604464 |
| Homo sapiens | 182 | JAG1 | AGS, AHD, AWS, CD339, HJ1, JAGL1 | jagged 1 | 20p12.1-p11.23 | 601920 |
| Homo sapiens | 3720 | JARID2 | JMJ | jumonji, AT rich interactive domain 2 | 6p24-p23 | 601594 |
| Homo sapiens | 8690 | JRKL | HHMJG | JRK-like | 11q21 | 603211 |
| Homo sapiens | 3725 | JUN | AP-1, AP1, c-Jun | jun proto-oncogene | 1p32-p31 | 165160 |
| Homo sapiens | 8997 | KALRN | ARHGEF24, CHD5, CHDS5, DUET, DUO, HAPIP, TRAD | kalirin, RhoGEF kinase | 3q21.2 | 604605 |
| Homo sapiens | 8850 | KAT2B | CAF, P/CAF, PCAF | K(lysine) acetyltransferase 2B | 3p24 | 602303 |
| Homo sapiens | 3746 | KCNC1 | EPM7, KV3.1, KV4, NGK2 | potassium channel, voltage gated Shaw related subfamily C, member 1 | 11p15 | 176258 |
| Homo sapiens | 3747 | KCNC2 | KV3.2 | potassium channel, voltage gated Shaw related subfamily C, member 2 | 12q14.1 | 176256 |
| Homo sapiens | 3756 | KCNH1 | EAG, EAG1, Kv10.1, TMBTS, h-eag | potassium channel, voltage gated eag related subfamily H, member 1 | 1q32.2 | 603305 |
| Homo sapiens | 3757 | KCNH2 | ERG-1, ERG1, H-ERG, HERG, HERG1, Kv11.1, LQT2, SQT1 | potassium channel, voltage gated eag related subfamily H, member 2 | 7q36.1 | 152427 |
| Homo sapiens | 3766 | KCNJ10 | BIRK-10, KCNJ13-PEN, KIR1.2, KIR4.1, SESAME | potassium channel, inwardly rectifying subfamily J, member 10 | 1q23.2 | 602208 |
| Homo sapiens | 3769 | KCNJ13 | KIR1.4, KIR7.1, LCA16, SVD | potassium channel, inwardly rectifying subfamily J, member 13 | 2q37 | 603208 |
| Homo sapiens | 3759 | KCNJ2 | ATFB9, HHBIRK1, HHIRK1, IRK1, KIR2.1, LQT7, SQT3 | potassium channel, inwardly rectifying subfamily J, member 2 | 17q24.3 | 600681 |
| Homo sapiens | 3762 | KCNJ5 | CIR, GIRK4, KATP1, KIR3.4, LQT13 | potassium channel, inwardly rectifying subfamily J, member 5 | 11q24 | 600734 |
| Homo sapiens | 3765 | KCNJ9 | GIRK3, KIR3.3 | potassium channel, inwardly rectifying subfamily J, member 9 | 1q23.2 | 600932 |
| Homo sapiens | 10242 | KCNMB2 |  | potassium channel subfamily M regulatory beta subunit 2 | 3q26.32 | 605214 |
| Homo sapiens | 3782 | KCNN3 | KCa2.3, SK3, SKCA3, hSK3 | potassium channel, calcium activated intermediate/small conductance subfamily N alpha, member 3 | 1q21.3 | 602983 |
| Homo sapiens | 3785 | KCNQ2 | BFNC, BFNS1, EBN, EBN1, EIEE7, ENB1, HNSPC, KCNA11, KV7.2, KVEBN1 | potassium channel, voltage gated KQT-like subfamily Q, member 2 | 20q13.3 | 602235 |
| Homo sapiens | 3790 | KCNS3 | KV9.3 | potassium voltage-gated channel, modifier subfamily S, member 3 | 2p24 | 603888 |
| Homo sapiens | 23081 | KDM4C | GASC1, JHDM3C, JMJD2C, TDRD14C | lysine (K)-specific demethylase 4C | 9p24.1 | 605469 |
| Homo sapiens | 9764 | KIAA0513 |  | KIAA0513 | 16q24.1 | 611675 |
| Homo sapiens | 57576 | KIF17 | KIF17B, KIF3X, KLP-2, OSM-3 | kinesin family member 17 | 1p36.12 | 605037 |
| Homo sapiens | 55605 | KIF21A | CFEOM1, FEOM1, FEOM3A | kinesin family member 21A | 12q12 | 608283 |
| Homo sapiens | 55083 | KIF26B |  | kinesin family member 26B | 1q44 | 614026 |
| Homo sapiens | 3796 | KIF2A | CDCBM3, HK2, KIF2 | kinesin heavy chain member 2A | 5q12-q13 | 602591 |
| Homo sapiens | 3812 | KIR3DL2 | CD158K, NKAT-4, NKAT4, NKAT4B, p140 | killer cell immunoglobulin-like receptor, three domains, long cytoplasmic tail, 2 | 19q13.4 | 604947 |
| Homo sapiens | 688 | KLF5 | BTEB2, CKLF, IKLF | Kruppel-like factor 5 (intestinal) | 13q22.1 | 602903 |
| Homo sapiens | 64410 | KLHL25 | ENC-2, ENC2 | kelch-like family member 25 | 15q25.3 |  |
| Homo sapiens | 8564 | KMO | dJ317G22.1 | kynurenine 3-monooxygenase (kynurenine 3-hydroxylase) | 1q42-q44 | 603538 |
| Homo sapiens | 4297 | KMT2A | ALL-1, CXXC7, HRX, HTRX1, MLL, MLL/GAS7, MLL1, MLL1A, TET1-MLL, TRX1, WDSTS | lysine (K)-specific methyltransferase 2A | 11q23 | 159555 |
| Homo sapiens | 3836 | KPNA1 | IPOA5, NPI-1, RCH2, SRP1 | karyopherin alpha 1 (importin alpha 5) | 3q21 | 600686 |
| Homo sapiens | 3839 | KPNA3 | IPOA4, SRP1, SRP1gamma, SRP4, hSRP1 | karyopherin alpha 3 (importin alpha 4) | 13q14.3 | 601892 |
| Homo sapiens | 83999 | KREMEN1 | KREMEN, KRM1 | kringle containing transmembrane protein 1 | 22q12.1 | 609898 |
| Homo sapiens | 3897 | L1CAM | CAML1, CD171, HSAS, HSAS1, MASA, MIC5, N-CAM-L1, N-CAML1, NCAM-L1, S10, SPG1 | L1 cell adhesion molecule | Xq28 | 308840 |
| Homo sapiens | 55915 | LANCL2 | GPR69B, TASP | LanC lantibiotic synthetase component C-like 2 (bacterial) | 7q31.1-q31.33 | 612919 |
| Homo sapiens | 23395 | LARS2 | LEURS, PRLTS4 | leucyl-tRNA synthetase 2, mitochondrial | 3p21.3 | 604544 |
| Homo sapiens | 3927 | LASP1 | Lasp-1, MLN50 | LIM and SH3 protein 1 | 17q11-q21.3 | 602920 |
| Homo sapiens | 3952 | LEP | LEPD, OB, OBS | leptin | 7q31.3 | 164160 |
| Homo sapiens | 3953 | LEPR | CD295, LEP-RD, OB-R, OBR, LEPR | leptin receptor | 1p31 | 601007 |
| Homo sapiens | 400696 | LGALS17A |  | Charcot-Leyden crystal protein pseudogene | 19q13.2 |  |
| Homo sapiens | 3977 | LIFR | CD118, LIF-R, SJS2, STWS, SWS | leukemia inhibitory factor receptor alpha | 5p13-p12 | 151443 |
| Homo sapiens | 145978 | LINC00052 | NCRNA00052, TMEM83 | long intergenic non-protein coding RNA 52 | 15q25.3 |  |
| Homo sapiens | 401247 | LINC00243 | C6orf214, NCRNA00243 | long intergenic non-protein coding RNA 243 | 6p21.33 |  |
| Homo sapiens | 100861552 | LINC00558 |  | long intergenic non-protein coding RNA 558 | 13q14.3 |  |
| Homo sapiens | 646982 | LINC00598 | TTL | long intergenic non-protein coding RNA 598 | 13q14.11 |  |
| Homo sapiens | 728586 | LINC01183 |  | long intergenic non-protein coding RNA 1183 | 5q23.2 |  |
| Homo sapiens | 101927433 | LINC01255 |  | long intergenic non-protein coding RNA 1255 | 18p11.21 |  |
| Homo sapiens | 101927134 | LINC01470 |  | long intergenic non-protein coding RNA 1470 | 5q33.1 |  |
| Homo sapiens | 101927921 | LINC01478 |  | long intergenic non-protein coding RNA 1478 | 18q12.3 |  |
| Homo sapiens | 84894 | LINGO1 | LERN1, LRRN6A, UNQ201 | leucine rich repeat and Ig domain containing 1 | 15q24.3 | 609791 |
| Homo sapiens | 84823 | LMNB2 | LAMB2, LMN2 | lamin B2 | 19p13.3 | 150341 |
| Homo sapiens | 4009 | LMX1A | LMX1, LMX1.1 | LIM homeobox transcription factor 1, alpha | 1q24.1 | 600298 |
| Homo sapiens | 4010 | LMX1B | LMX1.2, NPS1 | LIM homeobox transcription factor 1, beta | 9q33.3 | 602575 |
| Homo sapiens | 100128374 | LOC100128374 |  | uncharacterized LOC100128374 | 7p22.3 |  |
| Homo sapiens | 100128714 | LOC100128714 |  | uncharacterized LOC100128714 | 15q12 |  |
| Homo sapiens | 100506457 | LOC100506457 |  | uncharacterized LOC100506457 | 2p24.3 |  |
| Homo sapiens | 101927874 | LOC101927874 |  | uncharacterized LOC101927874 |  |  |
| Homo sapiens | 101928354 | LOC101928354 |  | uncharacterized LOC101928354 |  |  |
| Homo sapiens | 101928882 | LOC101928882 |  | uncharacterized LOC101928882 | 3q26.33 |  |
| Homo sapiens | 393076 | LOC393076 |  | uncharacterized LOC393076 | 7q36.3 |  |
| Homo sapiens | 9170 | LPAR2 | EDG-4, EDG4, LPA-2, LPA2 | lysophosphatidic acid receptor 2 | 19p12 | 605110 |
| Homo sapiens | 4023 | LPL | HDLCQ11, LIPD | lipoprotein lipase | 8p22 | 609708 |
| Homo sapiens | 145581 | LRFN5 | C14orf146, FIGLER8, SALM5 | leucine rich repeat and fibronectin type III domain containing 5 | 14q21.1 | 612811 |
| Homo sapiens | 4035 | LRP1 | A2MR, APOER, APR, CD91, IGFBP3R, LRPA, TGFBR5, LRP1 | low density lipoprotein receptor-related protein 1 | 12q13.3 | 107770 |
| Homo sapiens | 57689 | LRRC4C | NGL-1, NGL1 | leucine rich repeat containing 4C | 11p12 | 608817 |
| Homo sapiens | 127255 | LRRIQ3 | LRRC44 | leucine-rich repeats and IQ motif containing 3 | 1p31.1 |  |
| Homo sapiens | 120892 | LRRK2 | AURA17, DARDARIN, PARK8, RIPK7, ROCO2 | leucine-rich repeat kinase 2 | 12q12 | 609007 |
| Homo sapiens | 347730 | LRRTM1 |  | leucine rich repeat transmembrane neuronal 1 | 2p12 | 610867 |
| Homo sapiens | 4045 | LSAMP | IGLON3, LAMP | limbic system-associated membrane protein | 3q13.2-q21 | 603241 |
| Homo sapiens | 27257 | LSM1 | CASM, YJL124C | LSM1, U6 small nuclear RNA associated | 8p11.2 | 607281 |
| Homo sapiens | 4049 | LTA | LT, TNFB, TNFSF1 | lymphotoxin alpha | 6p21.3 | 153440 |
| Homo sapiens | 57128 | LYRM4 | C6orf149, CGI-203, COXPD19, ISD11 | LYR motif containing 4 | 6p25.1 | 613311 |
| Homo sapiens | 131375 | LYZL4 | LYC4, LYZA | lysozyme-like 4 | 3p22.1 | 612750 |
| Homo sapiens | 23499 | MACF1 | ABP620, ACF7, MACF, OFC4 | microtubule-actin crosslinking factor 1 | 1p32-p31 | 608271 |
| Homo sapiens | 8379 | MAD1L1 | MAD1, PIG9, TP53I9, TXBP181 | MAD1 mitotic arrest deficient-like 1 (yeast) | 7p22 | 602686 |
| Homo sapiens | 4099 | MAG | GMA, S-MAG, SIGLEC-4A, SIGLEC4A | myelin associated glycoprotein | 19q13.1 | 159460 |
| Homo sapiens | 54551 | MAGEL2 | NDNL1, PWLS, SHFYNG, nM15 | melanoma antigen family L2 | 15q11.2 | 605283 |
| Homo sapiens | 9223 | MAGI1 | AIP-3, AIP3, BAIAP1, BAP-1, BAP1, MAGI-1, Magi1d, TNRC19, WWP3 | membrane associated guanylate kinase, WW and PDZ domain containing 1 | 3p14.1 | 602625 |
| Homo sapiens | 9863 | MAGI2 | ACVRIP1, AIP-1, AIP1, ARIP1, MAGI-2, SSCAM | membrane associated guanylate kinase, WW and PDZ domain containing 2 | 7q21 | 606382 |
| Homo sapiens | 4128 | MAOA | MAO-A | monoamine oxidase A | Xp11.3 | 309850 |
| Homo sapiens | 4129 | MAOB |  | monoamine oxidase B | Xp11.23 | 309860 |
| Homo sapiens | 4130 | MAP1A | MAP1L, MTAP1A | microtubule-associated protein 1A | 15q15.3 | 600178 |
| Homo sapiens | 4133 | MAP2 | MAP2AB, MAP2C, MAP2 | microtubule-associated protein 2 | 2q34-q35 | 157130 |
| Homo sapiens | 5609 | MAP2K7 | JNKK2, MAPKK7, MEK, MEK 7, MKK7, PRKMK7, SAPKK-4, SAPKK4 | mitogen-activated protein kinase kinase 7 | 19p13.3-p13.2 | 603014 |
| Homo sapiens | 4293 | MAP3K9 | MEKK9, MLK1, PRKE1 | mitogen-activated protein kinase kinase kinase 9 | 14q24.2 | 600136 |
| Homo sapiens | 4135 | MAP6 | MAP6-N, MTAP6, N-STOP, STOP | microtubule-associated protein 6 | 11q13.5 | 601783 |
| Homo sapiens | 1432 | MAPK14 | CSBP, CSBP1, CSBP2, CSPB1, EXIP, Mxi2, PRKM14, PRKM15, RK, SAPK2A, p38, p38ALPHA | mitogen-activated protein kinase 14 | 6p21.3-p21.2 | 600289 |
| Homo sapiens | 4137 | MAPT | DDPAC, FTDP-17L, MSTD, MTBT1, MTBT2, PPND, PPP1R103, TAU, MAPT | microtubule-associated protein tau | 17q21.1 | 157140 |
| Homo sapiens | 4082 | MARCKS | 80K-L, MACS, PKCSL, PRKCSL | myristoylated alanine-rich protein kinase C substrate | 6q22.2 | 177061 |
| Homo sapiens | 23383 | MAU2 | KIAA0892L, SCC4, mau-2, MAU2 | MAU2 sister chromatid cohesion factor | 19p13.11 | 614560 |
| Homo sapiens | 4155 | MBP |  | myelin basic protein | 18q23 | 159430 |
| Homo sapiens | 4160 | MC4R |  | melanocortin 4 receptor | 18q22 | 155541 |
| Homo sapiens | 4163 | MCC | MCC1 | mutated in colorectal cancers | 5q21 | 159350 |
| Homo sapiens | 2847 | MCHR1 | GPR24, MCH-1R, MCH1R, SLC-1, SLC1 | melanin-concentrating hormone receptor 1 | 22q13.2 | 601751 |
| Homo sapiens | 79648 | MCPH1 | BRIT1, MCT | microcephalin 1 | 8p23.1 | 607117 |
| Homo sapiens | 55784 | MCTP2 |  | multiple C2 domains, transmembrane 2 | 15q26.2 | 616297 |
| Homo sapiens | 266727 | MDGA1 | GPIM, MAMDC3 | MAM domain containing glycosylphosphatidylinositol anchor 1 | 6p21 | 609626 |
| Homo sapiens | 4190 | MDH1 | HEL-S-32, MDH-s, MDHA, MGC:1375, MOR2 | malate dehydrogenase 1, NAD (soluble) | 2p13.3 | 154200 |
| Homo sapiens | 10873 | ME3 | NADP-ME | malic enzyme 3, NADP(+)-dependent, mitochondrial | 11cen-q22.3 | 604626 |
| Homo sapiens | 4204 | MECP2 | AUTSX3, MRX16, MRX79, MRXS13, MRXSL, PPMX, RS, RTS, RTT | methyl CpG binding protein 2 | Xq28 | 300005 |
| Homo sapiens | 9968 | MED12 | ARC240, CAGH45, FGS1, HOPAS, OHDOX, OKS, OPA1, TNRC11, TRAP230, MED12 | mediator complex subunit 12 | Xq13 | 300188 |
| Homo sapiens | 51586 | MED15 | ARC105, CAG7A, CTG7A, PCQAP, TIG-1, TIG1, TNRC7 | mediator complex subunit 15 | 22q11.2 | 607372 |
| Homo sapiens | 84466 | MEGF10 | EMARDD | multiple EGF-like-domains 10 | 5q33 | 612453 |
| Homo sapiens | 728637 | MEIKIN |  | meiotic kinetochore factor | 5q31.1 | 616232 |
| Homo sapiens | 4233 | MET | AUTS9, HGFR, RCCP2, c-Met | MET proto-oncogene, receptor tyrosine kinase | 7q31 | 164860 |
| Homo sapiens | 440823 | MIAT | C22orf35, GOMAFU, LINC00066, NCRNA00066, RNCR2, lncRNA-MIAT | myocardial infarction associated transcript (non-protein coding) | 22q12.1 | 611082 |
| Homo sapiens | 4277 | MICB | PERB11.2 | MHC class I polypeptide-related sequence B | 6p21.3 | 602436 |
| Homo sapiens | 406901 | MIR107 | MIRN107, miR-107 | microRNA 107 | 10q23.31 | 613189 |
| Homo sapiens | 100302174 | MIR1307 | MIRN1307, hsa-mir-1307 | microRNA 1307 |  |  |
| Homo sapiens | 406920 | MIR130B | MIRN130B, mir-130b | microRNA 130b |  | 613682 |
| Homo sapiens | 406925 | MIR135A1 | MIRN135-1, MIRN135A1 | microRNA 135a-1 | 3p21.1 |  |
| Homo sapiens | 442891 | MIR135B | MIRN135B | microRNA 135b | 1q32.1 |  |
| Homo sapiens | 406928 | MIR137 | MIRN137, miR-137 | microRNA 137 | 1p21.3 | 614304 |
| Homo sapiens | 400765 | MIR137HG |  | MIR137 host gene | 1p21.3 |  |
| Homo sapiens | 406930 | MIR138-2 | MIRN138-2 | microRNA 138-2 | 16q13 | 613395 |
| Homo sapiens | 406952 | MIR17 | MIR17-5p, MIR91, MIRN17, MIRN91, hsa-mir-17, miR-17, miR17-3p, miRNA17, miRNA91 | microRNA 17 | 13q31.3 | 609416 |
| Homo sapiens | 406959 | MIR183 | MIRN183, miR-183, miRNA183 | microRNA 183 | 7q32.2 | 611608 |
| Homo sapiens | 100616452 | MIR2682 |  | microRNA 2682 |  |  |
| Homo sapiens | 407034 | MIR30E | MIRN30E | microRNA 30e | 1p34.2 |  |
| Homo sapiens | 494331 | MIR382 | MIRN382, hsa-mir-382 | microRNA 382 | 14q32.31 |  |
| Homo sapiens | 494332 | MIR383 | MIRN383, hsa-mir-383 | microRNA 383 | 8p22 |  |
| Homo sapiens | 100422931 | MIR4304 |  | microRNA 4304 |  |  |
| Homo sapiens | 693225 | MIR640 | MIRN640, hsa-mir-640 | microRNA 640 | 19p13.11 |  |
| Homo sapiens | 102465503 | MIR6836 | hsa-mir-6836 | microRNA 6836 |  |  |
| Homo sapiens | 406890 | MIRLET7G | LET7G, MIRNLET7G, hsa-let-7g | microRNA let-7g | 3p21.1 | 612102 |
| Homo sapiens | 57591 | MKL1 | BSAC, MAL, MRTF-A | megakaryoblastic leukemia (translocation) 1 | 22q13 | 606078 |
| Homo sapiens | 23209 | MLC1 | LVM, MLC, VL | megalencephalic leukoencephalopathy with subcortical cysts 1 | 22q13.33 | 605908 |
| Homo sapiens | 4300 | MLLT3 | AF9, YEATS3 | myeloid/lymphoid or mixed-lineage leukemia (trithorax homolog, Drosophila); translocated to, 3 | 9p22 | 159558 |
| Homo sapiens | 4325 | MMP16 | C8orf57, MMP-X2, MT-MMP2, MT-MMP3, MT3-MMP | matrix metallopeptidase 16 (membrane-inserted) | 8q21.3 | 602262 |
| Homo sapiens | 4314 | MMP3 | CHDS6, MMP-3, SL-1, STMY, STMY1, STR1 | matrix metallopeptidase 3 | 11q22.3 | 185250 |
| Homo sapiens | 4318 | MMP9 | CLG4B, GELB, MANDP2, MMP-9 | matrix metallopeptidase 9 | 20q13.12 | 120361 |
| Homo sapiens | 4340 | MOG | BTN6, BTNL11IG2, NRCLP7, MOG | myelin oligodendrocyte glycoprotein | 6p22.1 | 159465 |
| Homo sapiens | 25874 | MPC2 | BRP44 | mitochondrial pyruvate carrier 2 | 1q24 | 614737 |
| Homo sapiens | 8777 | MPDZ | HYC2, MUPP1 | multiple PDZ domain protein | 9p23 | 603785 |
| Homo sapiens | 10198 | MPHOSPH9 | MPP-9, MPP9 | M-phase phosphoprotein 9 | 12q24.31 | 605501 |
| Homo sapiens | 9019 | MPZL1 | MPZL1b, PZR, PZR1b, PZRa, PZRb | myelin protein zero-like 1 | 1q24.2 | 604376 |
| Homo sapiens | 4482 | MSRA | PMSR | methionine sulfoxide reductase A | 8p23.1 | 601250 |
| Homo sapiens | 51734 | MSRB1 | HSPC270, SELR, SELX, SEPX1, SepR | methionine sulfoxide reductase B1 | 16p13.3 | 606216 |
| Homo sapiens | 118490 | MSS51 | ZMYND17 | MSS51 mitochondrial translational activator | 10q22.2 | 614773 |
| Homo sapiens | 4513 | MT-CO2 | COII, MTCO2, COX2 | mitochondrially encoded cytochrome c oxidase II |  |  |
| Homo sapiens | 4522 | MTHFD1 | MTHFC, MTHFD | methylenetetrahydrofolate dehydrogenase (NADP+ dependent) 1, methenyltetrahydrofolate cyclohydrolase, formyltetrahydrofolate synthetase | 14q24 | 172460 |
| Homo sapiens | 4524 | MTHFR |  | methylenetetrahydrofolate reductase (NAD(P)H) | 1p36.3 | 607093 |
| Homo sapiens | 54545 | MTMR12 | 3-PAP, PIP3AP | myotubularin related protein 12 | 5p13.3 | 606501 |
| Homo sapiens | 4535 | MT-ND1 | MTND1, ND1 | mitochondrially encoded NADH dehydrogenase 1 |  |  |
| Homo sapiens | 4540 | MT-ND5 | MTND5, ND5 | mitochondrially encoded NADH dehydrogenase 5 |  |  |
| Homo sapiens | 4543 | MTNR1A | MEL-1A-R, MT1 | melatonin receptor 1A | 4q35.1 | 600665 |
| Homo sapiens | 2475 | MTOR | FRAP, FRAP1, FRAP2, RAFT1, RAPT1 | mechanistic target of rapamycin (serine/threonine kinase) | 1p36.2 | 601231 |
| Homo sapiens | 4552 | MTRR | MSR, cblE | 5-methyltetrahydrofolate-homocysteine methyltransferase reductase | 5p15.31 | 602568 |
| Homo sapiens | 389125 | MUSTN1 | MUSTANG | musculoskeletal, embryonic nuclear protein 1 | 3p21.1 |  |
| Homo sapiens | 4627 | MYH9 | BDPLT6, DFNA17, EPSTS, FTNS, MHA, NMHC-II-A, NMMHC-IIA, NMMHCA | myosin, heavy chain 9, non-muscle | 22q13.1 | 160775 |
| Homo sapiens | 103910 | MYL12B | MLC-B, MRLC2 | myosin, light chain 12B, regulatory | 18p11.31 | 609211 |
| Homo sapiens | 23026 | MYO16 | MYAP3, MYR8, Myo16b, NYAP3, PPP1R107 | myosin XVI | 13q33.3 | 615479 |
| Homo sapiens | 84700 | MYO18B |  | myosin XVIIIB | 22q12.1 | 607295 |
| Homo sapiens | 4642 | MYO1D | PPP1R108, myr4 | myosin ID | 17q11-q12 | 606539 |
| Homo sapiens | 4645 | MYO5B |  | myosin VB | 18q21 | 606540 |
| Homo sapiens | 4650 | MYO9B | CELIAC4, MYR5 | myosin IXB | 19p13.1 | 602129 |
| Homo sapiens | 9172 | MYOM2 | TTNAP | myomesin 2 | 8p23.3 | 603509 |
| Homo sapiens | 23040 | MYT1L | NZF1, ZC2HC4B | myelin transcription factor 1-like | 2p25.3 | 613084 |
| Homo sapiens | 259232 | NALCN | CLIFAHDD, CanIon, IHPRF, INNFD, VGCNL1, bA430M15.1 | sodium leak channel, non selective | 13q32.3 | 611549 |
| Homo sapiens | 100885778 | NALCN-AS1 |  | NALCN antisense RNA 1 | 13q33.1 |  |
| Homo sapiens | 8774 | NAPG | GAMMASNAP | N-ethylmaleimide-sensitive factor attachment protein, gamma | 18p11.22 | 603216 |
| Homo sapiens | 55672 | NBPF1 | AB13, AB14, AB23, AD2, NBG, NBPF | neuroblastoma breakpoint family, member 1 | 1p36.13 | 610501 |
| Homo sapiens | 100132406 | NBPF10 | AB6, AG1, NBPF9 | neuroblastoma breakpoint family, member 10 | 1q21.1 | 614000 |
| Homo sapiens | 149013 | NBPF12 | COAS1, KIAA1245 | neuroblastoma breakpoint family, member 12 | 1q21 | 608607 |
| Homo sapiens | 644861 | NBPF13P |  | neuroblastoma breakpoint family, member 13, pseudogene | 1q21.1 |  |
| Homo sapiens | 25832 | NBPF14 | DJ328E19.C1.1, NBPF | neuroblastoma breakpoint family, member 14 | 1q21.2 | 614003 |
| Homo sapiens | 284565 | NBPF15 | AB14, AG3, NBPF16 | neuroblastoma breakpoint family, member 15 | 1q21.2 |  |
| Homo sapiens | 401967 | NBPF17P | NBPF23, NBPF23P | neuroblastoma breakpoint family, member 17, pseudogene | 1q21.2 | 612970 |
| Homo sapiens | 441908 | NBPF18P |  | neuroblastoma breakpoint family, member 18, pseudogene | 1q21.3 |  |
| Homo sapiens | 100288142 | NBPF20 |  | neuroblastoma breakpoint family, member 20 | 1q21.2 | 614007 |
| Homo sapiens | 205655 | NBPF21P |  | neuroblastoma breakpoint family, member 21, pseudogene | 3p22.2 |  |
| Homo sapiens | 285622 | NBPF22P |  | neuroblastoma breakpoint family, member 22, pseudogene | 5q14.3 |  |
| Homo sapiens | 101929780 | NBPF25P | WI2-925H4.1 | neuroblastoma breakpoint family, member 25, pseudogene |  |  |
| Homo sapiens | 343381 | NBPF2P |  | neuroblastoma breakpoint family, member 2, pseudogene | 1p36.12 |  |
| Homo sapiens | 84224 | NBPF3 | AE2 | neuroblastoma breakpoint family, member 3 | 1p36.12 | 612992 |
| Homo sapiens | 148545 | NBPF4 |  | neuroblastoma breakpoint family, member 4 | 1p13.3 | 613994 |
| Homo sapiens | 653149 | NBPF6 |  | neuroblastoma breakpoint family, member 6 | 1p13.3 | 613996 |
| Homo sapiens | 343505 | NBPF7 |  | neuroblastoma breakpoint family, member 7 | 1p12 | 613997 |
| Homo sapiens | 728841 | NBPF8 | NBPF8P | neuroblastoma breakpoint family, member 8 | 1q21.1 | 613998 |
| Homo sapiens | 400818 | NBPF9 | AE01 | neuroblastoma breakpoint family, member 9 | 1q21.1 | 613999 |
| Homo sapiens | 4684 | NCAM1 | CD56, MSK39, NCAM | neural cell adhesion molecule 1 | 11q23.1 | 116930 |
| Homo sapiens | 1463 | NCAN | CSPG3 | neurocan | 19p12 | 600826 |
| Homo sapiens | 344148 | NCKAP5 | ERIH1, ERIH2, NAP5 | NCK-associated protein 5 | 2q21.2 | 608789 |
| Homo sapiens | 23413 | NCS1 | FLUP, FREQ | neuronal calcium sensor 1 | 9q34 | 603315 |
| Homo sapiens | 54820 | NDE1 | HOM-TES-87, LIS4, MHAC, NDE, NUDE, NUDE1 | nudE neurodevelopment protein 1 | 16p13.11 | 609449 |
| Homo sapiens | 81565 | NDEL1 | EOPA, MITAP1, NDE1L1, NDE2, NUDEL | nudE neurodevelopment protein 1-like 1 | 17p13.1 | 607538 |
| Homo sapiens | 9348 | NDST3 | HSST3 | N-deacetylase/N-sulfotransferase (heparan glucosaminyl) 3 | 4q26 | 603950 |
| Homo sapiens | 51079 | NDUFA13 | B16.6, CDA016, CGI-39, GRIM-19, GRIM19 | NADH dehydrogenase (ubiquinone) 1 alpha subcomplex, 13 | 19p13.2 | 609435 |
| Homo sapiens | 4711 | NDUFB5 | CISGDH, SGDH | NADH dehydrogenase (ubiquinone) 1 beta subcomplex, 5, 16kDa | 3q26.33 | 603841 |
| Homo sapiens | 4715 | NDUFB9 | B22, CI-B22, LYRM3, UQOR22 | NADH dehydrogenase (ubiquinone) 1 beta subcomplex, 9, 22kDa | 8q13.3 | 601445 |
| Homo sapiens | 4719 | NDUFS1 | CI-75Kd, CI-75k, PRO1304 | NADH dehydrogenase (ubiquinone) Fe-S protein 1, 75kDa (NADH-coenzyme Q reductase) | 2q33-q34 | 157655 |
| Homo sapiens | 4722 | NDUFS3 | CI-30 | NADH dehydrogenase (ubiquinone) Fe-S protein 3, 30kDa (NADH-coenzyme Q reductase) | 11p11.11 | 603846 |
| Homo sapiens | 4726 | NDUFS6 | CI-13kA, CI-13kD-A, CI13KDA | NADH dehydrogenase (ubiquinone) Fe-S protein 6, 13kDa (NADH-coenzyme Q reductase) | 5p15.33 | 603848 |
| Homo sapiens | 374291 | NDUFS7 | CI-20, CI-20KD, MY017, PSST | NADH dehydrogenase (ubiquinone) Fe-S protein 7, 20kDa (NADH-coenzyme Q reductase) | 19p13.3 | 601825 |
| Homo sapiens | 4723 | NDUFV1 | CI-51K, CI51KD, UQOR1 | NADH dehydrogenase (ubiquinone) flavoprotein 1, 51kDa | 11q13 | 161015 |
| Homo sapiens | 4729 | NDUFV2 | CI-24k | NADH dehydrogenase (ubiquinone) flavoprotein 2, 24kDa | 18p11.22 | 600532 |
| Homo sapiens | 4747 | NEFL | CMT1F, CMT2E, NF-L, NF68, NFL, PPP1R110 | neurofilament, light polypeptide | 8p21 | 162280 |
| Homo sapiens | 4741 | NEFM | NEF3, NF-M, NFM | neurofilament, medium polypeptide | 8p21 | 162250 |
| Homo sapiens | 6787 | NEK4 | NRK2, STK2, pp12301 | NIMA-related kinase 4 | 3p21.1 | 601959 |
| Homo sapiens | 81832 | NETO1 | BCTL1, BTCL1 | neuropilin (NRP) and tolloid (TLL)-like 1 | 18q22.2 | 607973 |
| Homo sapiens | 4759 | NEU2 | SIAL2 | sialidase 2 (cytosolic sialidase) | 2q37 | 605528 |
| Homo sapiens | 4762 | NEUROG1 | AKA, Math4C, NEUROD3, bHLHa6, ngn1 | neurogenin 1 | 5q23-q31 | 601726 |
| Homo sapiens | 4790 | NFKB1 | EBP-1, KBF1, NF-kB1, NF-kappa-B, NF-kappaB, NFKB-p105, NFKB-p50, NFkappaB, p105, p50 | nuclear factor of kappa light polypeptide gene enhancer in B-cells 1 | 4q24 | 164011 |
| Homo sapiens | 25791 | NGEF | ARHGEF27, EPHEXIN | neuronal guanine nucleotide exchange factor | 2q37 | 605991 |
| Homo sapiens | 4803 | NGF | Beta-NGF, HSAN5B, NGF | nerve growth factor (beta polypeptide) | 1p13.1 | 162030 |
| Homo sapiens | 4804 | NGFR | CD271, Gp80-LNGFR, TNFRSF16, p75(NTR), p75NTR | nerve growth factor receptor | 17q21-q22 | 162010 |
| Homo sapiens | 11188 | NISCH | I-1, IR1, IRAS, hIRAS | nischarin | 3p21.1 | 615507 |
| Homo sapiens | 154215 | NKAIN2 | FAM77B, NKAIP2, TCBA, TCBA1 | Na+/K+ transporting ATPase interacting 2 | 6q21 | 609758 |
| Homo sapiens | 222698 | NKAPL | C6orf194, bA424I5.1 | NFKB activating protein-like | 6p22.1 |  |
| Homo sapiens | 57555 | NLGN2 |  | neuroligin 2 | 17p13.1 | 606479 |
| Homo sapiens | 84166 | NLRC5 | CLR16.1, NOD27, NOD4 | NLR family, CARD domain containing 5 | 16q13 | 613537 |
| Homo sapiens | 56923 | NMUR2 | FM-4, FM4, NMU-R2, NMU2R, TGR-1, TGR1 | neuromedin U receptor 2 | 5q33.1 | 605108 |
| Homo sapiens | 4837 | NNMT |  | nicotinamide N-methyltransferase | 11q23.1 | 600008 |
| Homo sapiens | 4841 | NONO | NMT55, NRB54, P54, P54NRB, PPP1R114 | non-POU domain containing, octamer-binding | Xq13.1 | 300084 |
| Homo sapiens | 4842 | NOS1 | IHPS1, N-NOS, NC-NOS, NOS, bNOS, nNOS | nitric oxide synthase 1 (neuronal) | 12q24.22 | 163731 |
| Homo sapiens | 9722 | NOS1AP | 6330408P19Rik, CAPON | nitric oxide synthase 1 (neuronal) adaptor protein | 1q23.3 | 605551 |
| Homo sapiens | 4846 | NOS3 | ECNOS, eNOS | nitric oxide synthase 3 (endothelial cell) | 7q36 | 163729 |
| Homo sapiens | 4855 | NOTCH4 | INT3 | notch 4 | 6p21.3 | 164951 |
| Homo sapiens | 64067 | NPAS3 | MOP6, PASD6, bHLHe12 | neuronal PAS domain protein 3 | 14q13.1 | 609430 |
| Homo sapiens | 27020 | NPTN | GP55, GP65, SDFR1, SDR1, np55, np65 | neuroplastin | 15q22 | 612820 |
| Homo sapiens | 4852 | NPY | PYY4 | neuropeptide Y | 7p15.1 | 162640 |
| Homo sapiens | 1728 | NQO1 | DHQU, DIA4, DTD, NMOR1, NMORI, QR1 | NAD(P)H dehydrogenase, quinone 1 | 16q22.1 | 125860 |
| Homo sapiens | 4835 | NQO2 | DHQV, DIA6, NMOR2, QR2 | NAD(P)H dehydrogenase, quinone 2 | 6p25.2 | 160998 |
| Homo sapiens | 2908 | NR3C1 | GCCR, GCR, GCRST, GR, GRL | nuclear receptor subfamily 3, group C, member 1 (glucocorticoid receptor) | 5q31.3 | 138040 |
| Homo sapiens | 4929 | NR4A2 | HZF-3, NOT, NURR1, RNR1, TINUR | nuclear receptor subfamily 4, group A, member 2 | 2q22-q23 | 601828 |
| Homo sapiens | 8013 | NR4A3 | CHN, CSMF, MINOR, NOR1, TEC | nuclear receptor subfamily 4, group A, member 3 | 9q22 | 600542 |
| Homo sapiens | 4898 | NRD1 | hNRD1, hNRD2 | nardilysin (N-arginine dibasic convertase) | 1p32.2-p32.1 | 602651 |
| Homo sapiens | 9315 | NREP | C5orf13, D4S114, P311, PRO1873, PTZ17, SEZ17 | neuronal regeneration related protein | 5q22.1 | 607332 |
| Homo sapiens | 3084 | NRG1 | ARIA, GGF, GGF2, HGL, HRG, HRG1, HRGA, MST131, MSTP131, NDF-IT2, SMDF, NRG1 | neuregulin 1 | 8p12 | 142445 |
| Homo sapiens | 10718 | NRG3 | HRG3, pro-NRG3 | neuregulin 3 | 10q23.1 | 605533 |
| Homo sapiens | 4900 | NRGN | RC3, hng | neurogranin (protein kinase C substrate, RC3) | 11q24 | 602350 |
| Homo sapiens | 51299 | NRN1 | NRN, dJ380B8.2 | neuritin 1 | 6p25.1 | 607409 |
| Homo sapiens | 8829 | NRP1 | BDCA4, CD304, NP1, NRP, VEGF165R | neuropilin 1 | 10p12 | 602069 |
| Homo sapiens | 9378 | NRXN1 | Hs.22998, PTHSL2, SCZD17 | neurexin 1 | 2p16.3 | 600565 |
| Homo sapiens | 9379 | NRXN2 |  | neurexin 2 | 11q13 | 600566 |
| Homo sapiens | 9369 | NRXN3 | C14orf60 | neurexin 3 | 14q31 | 600567 |
| Homo sapiens | 55968 | NSFL1C | P47, UBX1, UBXD10, UBXN2C, dJ776F14.1 | NSFL1 (p97) cofactor (p47) | 20p13 | 606610 |
| Homo sapiens | 221078 | NSUN6 | 4933414E04Rik, ARL5B-AS1, NOPD1 | NOP2/Sun domain family, member 6 | 10p12.31 |  |
| Homo sapiens | 22978 | NT5C2 | GMP, NT5B, PNT5, SPG45, SPG65, cN-II | 5'-nucleotidase, cytosolic II | 10q24.32 | 600417 |
| Homo sapiens | 64943 | NT5DC2 |  | 5'-nucleotidase domain containing 2 | 3p21.1 |  |
| Homo sapiens | 4908 | NTF3 | HDNF, NGF-2, NGF2, NT-3, NT3 | neurotrophin 3 | 12p13 | 162660 |
| Homo sapiens | 50863 | NTM | HNT, IGLON2, NTRI | neurotrimin | 11q25 | 607938 |
| Homo sapiens | 22854 | NTNG1 | Lmnt1 | netrin G1 | 1p13.3 | 608818 |
| Homo sapiens | 84628 | NTNG2 | LHLL9381, Lmnt2, NTNG1, bA479K20.1 | netrin G2 | 9q34 |  |
| Homo sapiens | 4914 | NTRK1 | MTC, TRK, TRK1, TRKA, Trk-A, p140-TrkA | neurotrophic tyrosine kinase, receptor, type 1 | 1q21-q22 | 191315 |
| Homo sapiens | 4915 | NTRK2 | GP145-TrkB, TRKB, trk-B | neurotrophic tyrosine kinase, receptor, type 2 | 9q22.1 | 600456 |
| Homo sapiens | 4916 | NTRK3 | GP145-TrkC, TRKC, gp145(trkC) | neurotrophic tyrosine kinase, receptor, type 3 | 15q25 | 191316 |
| Homo sapiens | 4923 | NTSR1 | NTR | neurotensin receptor 1 (high affinity) | 20q13 | 162651 |
| Homo sapiens | 80224 | NUBPL | C14orf127, IND1, huInd1 | nucleotide binding protein-like | 14q12 | 613621 |
| Homo sapiens | 64710 | NUCKS1 | JC7, NUCKS | nuclear casein kinase and cyclin-dependent kinase substrate 1 | 1q32.1 | 611912 |
| Homo sapiens | 4521 | NUDT1 | MTH1 | nudix (nucleoside diphosphate linked moiety X)-type motif 1 | 7p22 | 600312 |
| Homo sapiens | 79676 | OGFOD2 |  | 2-oxoglutarate and iron-dependent oxygenase domain containing 2 | 12q24.31 |  |
| Homo sapiens | 388906 | OGFRP1 |  | opioid growth factor receptor pseudogene 1 | 22q13.2 |  |
| Homo sapiens | 4968 | OGG1 | HMMH, HOGG1, MUTM, OGH1 | 8-oxoguanine DNA glycosylase | 3p26.2 | 601982 |
| Homo sapiens | 10215 | OLIG2 | BHLHB1, OLIGO2, PRKCBP2, RACK17, bHLHe19 | oligodendrocyte lineage transcription factor 2 | 21q22.11 | 606386 |
| Homo sapiens | 4974 | OMG | OMGP | oligodendrocyte myelin glycoprotein | 17q11.2 | 164345 |
| Homo sapiens | 4976 | OPA1 | MGM1, NPG, NTG, largeG | optic atrophy 1 (autosomal dominant) | 3q29 | 605290 |
| Homo sapiens | 4978 | OPCML | IGLON1, OBCAM, OPCM | opioid binding protein/cell adhesion molecule-like | 11q25 | 600632 |
| Homo sapiens | 4985 | OPRD1 | OPRD | opioid receptor, delta 1 | 1p36.1-p34.3 | 165195 |
| Homo sapiens | 4988 | OPRM1 | LMOR, M-OR-1, MOP, MOR, MOR1, OPRM | opioid receptor, mu 1 | 6q24-q25 | 600018 |
| Homo sapiens | 121364 | OR10A7 | OR12-6 | olfactory receptor, family 10, subfamily A, member 7 | 12q13.2 |  |
| Homo sapiens | 121130 | OR10P1 | OR10P1P, OR10P2P, OR10P3P, OR12-7, OST701 | olfactory receptor, family 10, subfamily P, member 1 | 12q13.2 |  |
| Homo sapiens | 390321 | OR6C1 | OST267 | olfactory receptor, family 6, subfamily C, member 1 | 12q13.2 |  |
| Homo sapiens | 341416 | OR6C2 | OR6C67 | olfactory receptor, family 6, subfamily C, member 2 | 12q13.2 |  |
| Homo sapiens | 254786 | OR6C3 | OST709 | olfactory receptor, family 6, subfamily C, member 3 | 12q13.2 |  |
| Homo sapiens | 341418 | OR6C4 | OR12-10 | olfactory receptor, family 6, subfamily C, member 4 | 12q13.2 |  |
| Homo sapiens | 283365 | OR6C6 |  | olfactory receptor, family 6, subfamily C, member 6 | 12q13.2 |  |
| Homo sapiens | 403281 | OR6C64P |  | olfactory receptor, family 6, subfamily C, member 64 pseudogene | 12q13.2 |  |
| Homo sapiens | 403282 | OR6C65 |  | olfactory receptor, family 6, subfamily C, member 65 | 12q13.2 |  |
| Homo sapiens | 403284 | OR6C68 |  | olfactory receptor, family 6, subfamily C, member 68 | 12q13.2 |  |
| Homo sapiens | 390327 | OR6C70 |  | olfactory receptor, family 6, subfamily C, member 70 | 12q13.2 |  |
| Homo sapiens | 254783 | OR6C74 |  | olfactory receptor, family 6, subfamily C, member 74 | 12q13.2 |  |
| Homo sapiens | 390323 | OR6C75 |  | olfactory receptor, family 6, subfamily C, member 75 | 12q13.2 |  |
| Homo sapiens | 390326 | OR6C76 |  | olfactory receptor, family 6, subfamily C, member 76 | 12q13.2 |  |
| Homo sapiens | 441639 | OR9K2 | OR12-2 | olfactory receptor, family 9, subfamily K, member 2 | 12q13.2 |  |
| Homo sapiens | 5019 | OXCT1 | OXCT, SCOT | 3-oxoacid CoA transferase 1 | 5p13.1 | 601424 |
| Homo sapiens | 9943 | OXSR1 | OSR1 | oxidative stress responsive 1 | 3p22.2 | 604046 |
| Homo sapiens | 5020 | OXT | OT, OT-NPI-NPI, OXT | oxytocin/neurophysin I prepropeptide | 20p13 | 167050 |
| Homo sapiens | 5021 | OXTR | OT-R | oxytocin receptor | 3p25 | 167055 |
| Homo sapiens | 5027 | P2RX7 | P2X7 | purinergic receptor P2X, ligand gated ion channel, 7 | 12q24 | 602566 |
| Homo sapiens | 11240 | PADI2 | PAD-H19, PAD2, PDI2 | peptidyl arginine deiminase, type II | 1p36.13 | 607935 |
| Homo sapiens | 5048 | PAFAH1B1 | LIS1, LIS2, MDCR, MDS, PAFAH | platelet-activating factor acetylhydrolase 1b, regulatory subunit 1 (45kDa) | 17p13.3 | 601545 |
| Homo sapiens | 5053 | PAH | PH, PKU, PKU1 | phenylalanine hydroxylase | 12q22-q24.2 | 612349 |
| Homo sapiens | 23022 | PALLD | CGI-151, CGI151, MYN, PNCA1, SIH002 | palladin, cytoskeletal associated protein | 4q32.3 | 608092 |
| Homo sapiens | 56666 | PANX2 | PX2, hPANX2 | pannexin 2 | 22q13.33 | 608421 |
| Homo sapiens | 54852 | PAQR5 | MPRG | progestin and adipoQ receptor family member V | 15q23 | 607781 |
| Homo sapiens | 56288 | PARD3 | ASIP, Baz, PAR3, PAR3alpha, PARD-3A, PPP1R118, SE2-5L16, SE2-5LT1, SE2-5T2, PARD3 | par-3 family cell polarity regulator | 10p11.21 | 606745 |
| Homo sapiens | 11315 | PARK7 | DJ-1, DJ1, HEL-S-67p | parkinson protein 7 | 1p36.23 | 602533 |
| Homo sapiens | 5074 | PAWR | PAR4, Par-4 | PRKC, apoptosis, WT1, regulator | 12q21 | 601936 |
| Homo sapiens | 55193 | PBRM1 | BAF180, PB1 | polybromo 1 | 3p21 | 606083 |
| Homo sapiens | 80714 | PBX4 |  | pre-B-cell leukemia homeobox 4 | 19p12 | 608127 |
| Homo sapiens | 83259 | PCDH11Y | PCDH-PC, PCDH22, PCDHX, PCDHY | protocadherin 11 Y-linked | Yp11.2 | 400022 |
| Homo sapiens | 64881 | PCDH20 | PCDH13 | protocadherin 20 | 13q21 | 614449 |
| Homo sapiens | 5100 | PCDH8 | ARCADLIN, PAPC | protocadherin 8 | 13q21.1 | 603580 |
| Homo sapiens | 56147 | PCDHA1 | PCDH-ALPHA1 | protocadherin alpha 1 | 5q31 | 606307 |
| Homo sapiens | 64002 | PCGEM1 | LINC00071, NCRNA00071, PCAT9 | PCGEM1, prostate-specific transcript (non-protein coding) | 2q32 | 605443 |
| Homo sapiens | 84108 | PCGF6 | MBLR, RNF134 | polycomb group ring finger 6 | 10q24.33 | 607816 |
| Homo sapiens | 5108 | PCM1 | PTC4 | pericentriolar material 1 | 8p22-p21.3 | 600299 |
| Homo sapiens | 5110 | PCMT1 | PIMT | protein-L-isoaspartate (D-aspartate) O-methyltransferase | 6q25.1 | 176851 |
| Homo sapiens | 5111 | PCNA | ATLD2 | proliferating cell nuclear antigen | 20pter-p12 | 176740 |
| Homo sapiens | 5116 | PCNT | KEN, MOPD2, PCN2, PCNTB, PCTN2, SCKL4, PCNT | pericentrin | 21q22.3 | 605925 |
| Homo sapiens | 22984 | PDCD11 | ALG-4, ALG4, NFBP, RRP5 | programmed cell death 11 | 10q24.33 | 612333 |
| Homo sapiens | 5141 | PDE4A | DPDE2, PDE4, PDE46 | phosphodiesterase 4A, cAMP-specific | 19p13.2 | 600126 |
| Homo sapiens | 5142 | PDE4B | DPDE4, PDEIVB | phosphodiesterase 4B, cAMP-specific | 1p31 | 600127 |
| Homo sapiens | 5144 | PDE4D | ACRDYS2, DPDE3, HSPDE4D, PDE43N2, STRK1, PDE4D | phosphodiesterase 4D, cAMP-specific | 5q12 | 600129 |
| Homo sapiens | 5155 | PDGFB | IBGC5, PDGF-2, PDGF2, SIS, SSV, c-sis | platelet-derived growth factor beta polypeptide | 22q13.1 | 190040 |
| Homo sapiens | 5159 | PDGFRB | CD140B, IBGC4, IMF1, JTK12, PDGFR, PDGFR-1, PDGFR1 | platelet-derived growth factor receptor, beta polypeptide | 5q33.1 | 173410 |
| Homo sapiens | 10611 | PDLIM5 | ENH, ENH1, L9, LIM | PDZ and LIM domain 5 | 4q22 | 605904 |
| Homo sapiens | 5173 | PDYN | ADCA, PENKB, SCA23 | prodynorphin | 20p13 | 131340 |
| Homo sapiens | 23037 | PDZD2 | AIPC, PAPIN, PDZK3, PIN1 | PDZ domain containing 2 | 5p13.3 | 610697 |
| Homo sapiens | 5174 | PDZK1 | CAP70, CLAMP, NHERF-3, NHERF3, PDZD1 | PDZ domain containing 1 | 1q21 | 603831 |
| Homo sapiens | 8682 | PEA15 | HMAT1, HUMMAT1H, MAT1, MAT1H, PEA-15, PED | phosphoprotein enriched in astrocytes 15 | 1q21.1 | 603434 |
| Homo sapiens | 5037 | PEBP1 | HCNP, HCNPpp, HEL-210, HEL-S-34, PBP, PEBP, PEBP-1, RKIP | phosphatidylethanolamine binding protein 1 | 12q24.23 | 604591 |
| Homo sapiens | 10400 | PEMT | PEAMT, PEMPT2, PNMT, PEMT | phosphatidylethanolamine N-methyltransferase | 17p11.2 | 602391 |
| Homo sapiens | 5179 | PENK |  | proenkephalin | 8q23-q24 | 131330 |
| Homo sapiens | 8863 | PER3 | GIG13 | period circadian clock 3 | 1p36.23 | 603427 |
| Homo sapiens | 5213 | PFKM | ATP-PFK, GSD7, PFK-1, PFK1, PFKA, PFKX, PPP1R122 | phosphofructokinase, muscle | 12q13.3 | 610681 |
| Homo sapiens | 5223 | PGAM1 | HEL-S-35, PGAM-B, PGAMA | phosphoglycerate mutase 1 (brain) | 10q25.3 | 172250 |
| Homo sapiens | 84547 | PGBD1 | HUCEP-4, SCAND4, dJ874C20.4 | piggyBac transposable element derived 1 | 6p22.1 |  |
| Homo sapiens | 5226 | PGD | 6PGD | phosphogluconate dehydrogenase | 1p36.22 | 172200 |
| Homo sapiens | 5230 | PGK1 | HEL-S-68p, MIG10, PGKA | phosphoglycerate kinase 1 | Xq13.3 | 311800 |
| Homo sapiens | 54858 | PGPEP1 | PAP-I, PGP, PGP-I, PGPI, Pcp | pyroglutamyl-peptidase I | 19p13.11 | 610694 |
| Homo sapiens | 5245 | PHB | HEL-215, HEL-S-54e1, PHB | prohibitin | 17q21 | 176705 |
| Homo sapiens | 51533 | PHF7 | HSPC045, HSPC226, NYD-SP6 | PHD finger protein 7 | 3p21.1 |  |
| Homo sapiens | 23133 | PHF8 | JHDM1F, MRXSSD, ZNF422 | PHD finger protein 8 | Xp11.22 | 300560 |
| Homo sapiens | 26227 | PHGDH | 3-PGDH, 3PGDH, HEL-S-113, NLS, NLS1, PDG, PGAD, PGD, PGDHD, SERA, PHGDH | phosphoglycerate dehydrogenase | 1p12 | 606879 |
| Homo sapiens | 55300 | PI4K2B | PI4KIIB, PIK42B | phosphatidylinositol 4-kinase type 2 beta | 4p15.2 | 612101 |
| Homo sapiens | 5297 | PI4KA | PI4K-ALPHA, PIK4CA, pi4K230 | phosphatidylinositol 4-kinase, catalytic, alpha | 22q11.21 | 600286 |
| Homo sapiens | 8301 | PICALM | CALM, CLTH, LAP | phosphatidylinositol binding clathrin assembly protein | 11q14 | 603025 |
| Homo sapiens | 9463 | PICK1 | PICK, PRKCABP | protein interacting with PRKCA 1 | 22q13.1 | 605926 |
| Homo sapiens | 55022 | PID1 | HMFN2073, NYGGF4, P-CLI1, PCLI1 | phosphotyrosine interaction domain containing 1 | 2q36.3 | 612930 |
| Homo sapiens | 5286 | PIK3C2A | CPK, PI3-K-C2(ALPHA), PI3-K-C2A | phosphatidylinositol-4-phosphate 3-kinase, catalytic subunit type 2 alpha | 11p15.5-p14 | 603601 |
| Homo sapiens | 5288 | PIK3C2G | PI3K-C2-gamma, PI3K-C2GAMMA | phosphatidylinositol-4-phosphate 3-kinase, catalytic subunit type 2 gamma | 12p12 | 609001 |
| Homo sapiens | 5289 | PIK3C3 | VPS34, Vps34 | phosphatidylinositol 3-kinase, catalytic subunit type 3 | 18q12.3 | 602609 |
| Homo sapiens | 5293 | PIK3CD | APDS, IMD14, P110DELTA, PI3K, p110D | phosphatidylinositol-4,5-bisphosphate 3-kinase, catalytic subunit delta | 1p36.2 | 602839 |
| Homo sapiens | 65018 | PINK1 | BRPK, PARK6 | PTEN induced putative kinase 1 | 1p36 | 608309 |
| Homo sapiens | 5305 | PIP4K2A | PI5P4KA, PIP5K2A, PIP5KII-alpha, PIP5KIIA, PIPK | phosphatidylinositol-5-phosphate 4-kinase, type II, alpha | 10p12.2 | 603140 |
| Homo sapiens | 5306 | PITPNA | HEL-S-36, PI-TPalpha, PITPN, VIB1A | phosphatidylinositol transfer protein, alpha | 17p13.3 | 600174 |
| Homo sapiens | 57605 | PITPNM2 | NIR-3, NIR3, RDGB2, RDGBA2 | phosphatidylinositol transfer protein, membrane-associated 2 | 12q24.31 | 608920 |
| Homo sapiens | 5309 | PITX3 | ASMD, ASOD, CTPP4, CTRCT11, PTX3 | paired-like homeodomain 3 | 10q24.32 | 602669 |
| Homo sapiens | 63876 | PKNOX2 | PREP2 | PBX/knotted 1 homeobox 2 | 11q24.2 | 613066 |
| Homo sapiens | 8502 | PKP4 | p0071 | plakophilin 4 | 2q24.1 | 604276 |
| Homo sapiens | 5320 | PLA2G2A | MOM1, PLA2, PLA2B, PLA2L, PLA2S, PLAS1, sPLA2 | phospholipase A2, group IIA (platelets, synovial fluid) | 1p35 | 172411 |
| Homo sapiens | 5321 | PLA2G4A | PLA2G4, cPLA2-alpha | phospholipase A2, group IVA (cytosolic, calcium-dependent) | 1q25 | 600522 |
| Homo sapiens | 8605 | PLA2G4C | CPLA2-gamma | phospholipase A2, group IVC (cytosolic, calcium-independent) | 19q13.3 | 603602 |
| Homo sapiens | 8398 | PLA2G6 | CaI-PLA2, GVI, INAD1, IPLA2-VIA, NBIA2, NBIA2A, NBIA2B, PARK14, PLA2, PNPLA9, iPLA2, iPLA2beta | phospholipase A2, group VI (cytosolic, calcium-independent) | 22q13.1 | 603604 |
| Homo sapiens | 7941 | PLA2G7 | LDL-PLA2, LP-PLA2, PAFAD, PAFAH | phospholipase A2, group VII (platelet-activating factor acetylhydrolase, plasma) | 6p21.2-p12 | 601690 |
| Homo sapiens | 9373 | PLAA | DOA1, PLA2P, PLAP | phospholipase A2-activating protein | 9p21 | 603873 |
| Homo sapiens | 5327 | PLAT | T-PA, TPA | plasminogen activator, tissue | 8p12 | 173370 |
| Homo sapiens | 23236 | PLCB1 | EIEE12, PI-PLC, PLC-154, PLC-I, PLC154A, PLCB1B, PLCB1 | phospholipase C, beta 1 (phosphoinositide-specific) | 20p12 | 607120 |
| Homo sapiens | 5330 | PLCB2 | PLC-beta-2 | phospholipase C, beta 2 | 15q15 | 604114 |
| Homo sapiens | 5339 | PLEC | EBS1, EBSO, HD1, LGMD2Q, PCN1, PLEC1b, PLTN, PLEC | plectin | 8q24 | 601282 |
| Homo sapiens | 5354 | PLP1 | GPM6C, HLD1, MMPL, PLP, PLP/DM20, PMD, SPG2 | proteolipid protein 1 | Xq22 | 300401 |
| Homo sapiens | 5362 | PLXNA2 | OCT, PLXN2 | plexin A2 | 1q32.2 | 601054 |
| Homo sapiens | 55163 | PNPO | HEL-S-302, PDXPO | pyridoxamine 5'-phosphate oxidase | 17q21.32 | 603287 |
| Homo sapiens | 94026 | POM121L2 | POM121-L, POM121L | POM121 transmembrane nucleoporin-like 2 | 6p22.1 |  |
| Homo sapiens | 5443 | POMC | ACTH, CLIP, LPH, MSH, NPP, POC | proopiomelanocortin | 2p23.3 | 176830 |
| Homo sapiens | 5444 | PON1 | ESA, MVCD5, PON | paraoxonase 1 | 7q21.3 | 168820 |
| Homo sapiens | 5454 | POU3F2 | BRN2, N-Oct3, OCT7, OTF-7, OTF7, POUF3, brn-2, oct-7 | POU class 3 homeobox 2 | 6q16 | 600494 |
| Homo sapiens | 27068 | PPA2 | HSPC124, SID6-306 | pyrophosphatase (inorganic) 2 | 4q25 | 609988 |
| Homo sapiens | 5465 | PPARA | NR1C1, PPAR, PPARalpha, hPPAR | peroxisome proliferator-activated receptor alpha | 22q13.31 | 170998 |
| Homo sapiens | 5468 | PPARG | CIMT1, GLM1, NR1C31, PPARG2, PPARgamma, PPARG | peroxisome proliferator-activated receptor gamma | 3p25 | 601487 |
| Homo sapiens | 8499 | PPFIA2 |  | protein tyrosine phosphatase, receptor type, f polypeptide (PTPRF), interacting protein (liprin), alpha 2 | 12q21.31 | 603143 |
| Homo sapiens | 5478 | PPIA | CYPA, CYPH, HEL-S-69p | peptidylprolyl isomerase A (cyclophilin A) | 7p13 | 123840 |
| Homo sapiens | 132160 | PPM1M | PP2C-eta, PP2CE, PP2Ceta | protein phosphatase, Mg2+/Mn2+ dependent, 1M | 3p21.2 | 608979 |
| Homo sapiens | 84152 | PPP1R1B | DARPP-32, DARPP32 | protein phosphatase 1, regulatory (inhibitor) subunit 1B | 17q12 | 604399 |
| Homo sapiens | 84687 | PPP1R9B | PPP1R6, PPP1R9, SPINO, Spn | protein phosphatase 1, regulatory subunit 9B | 17q21.33 | 603325 |
| Homo sapiens | 5530 | PPP3CA | CALN, CALNA, CALNA1, CCN1, CNA1, PPP2B | protein phosphatase 3, catalytic subunit, alpha isozyme | 4q24 | 114105 |
| Homo sapiens | 5532 | PPP3CB | CALNA2, CALNB, CNA2, PP2Bbeta | protein phosphatase 3, catalytic subunit, beta isozyme | 10q22.2 | 114106 |
| Homo sapiens | 5533 | PPP3CC | CALNA3, CNA3, PP2Bgamma | protein phosphatase 3, catalytic subunit, gamma isozyme | 8p21.3 | 114107 |
| Homo sapiens | 5534 | PPP3R1 | CALNB1, CNB, CNB1 | protein phosphatase 3, regulatory subunit B, alpha | 2p15 | 601302 |
| Homo sapiens | 5538 | PPT1 | CLN1, INCL, PPT | palmitoyl-protein thioesterase 1 | 1p32 | 600722 |
| Homo sapiens | 5052 | PRDX1 | MSP23, NKEF-A, NKEFA, PAG, PAGA, PAGB, PRX1, PRXI, TDPX2 | peroxiredoxin 1 | 1p34.1 | 176763 |
| Homo sapiens | 7001 | PRDX2 | HEL-S-2a, NKEF-B, NKEFB, PRP, PRX2, PRXII, PTX1, TDPX1, TPX1, TSA | peroxiredoxin 2 | 19p13.2 | 600538 |
| Homo sapiens | 9588 | PRDX6 | 1-Cys, AOP2, HEL-S-128m, NSGPx, PRX, aiPLA2, p29 | peroxiredoxin 6 | 1q25.1 | 602316 |
| Homo sapiens | 5563 | PRKAA2 | AMPK, AMPK2, AMPKa2, PRKAA | protein kinase, AMP-activated, alpha 2 catalytic subunit | 1p31 | 600497 |
| Homo sapiens | 5565 | PRKAB2 |  | protein kinase, AMP-activated, beta 2 non-catalytic subunit | 1q21.1 | 602741 |
| Homo sapiens | 5578 | PRKCA | AAG6, PKC-alpha, PKCA, PRKACA | protein kinase C, alpha | 17q22-q23.2 | 176960 |
| Homo sapiens | 5582 | PRKCG | PKC-gamma, PKCC, PKCG, SCA14 | protein kinase C, gamma | 19q13.4 | 176980 |
| Homo sapiens | 5590 | PRKCZ | PKC-ZETA, PKC2 | protein kinase C, zeta | 1p36.33-p36.2 | 176982 |
| Homo sapiens | 23683 | PRKD3 | EPK2, PKC-NU, PKD3, PRKCN, nPKC-NU | protein kinase D3 | 2p21 | 607077 |
| Homo sapiens | 5617 | PRL |  | prolactin | 6p22.3 | 176760 |
| Homo sapiens | 5618 | PRLR | HPRL, MFAB, hPRLrI | prolactin receptor | 5p13.2 | 176761 |
| Homo sapiens | 5621 | PRNP | ASCR, AltPrP, CD230, CJD, GSS, KURU, PRIP, PrP, PrP27-30, PrP33-35C, PrPc, p27-30 | prion protein | 20p13 | 176640 |
| Homo sapiens | 5625 | PRODH | HSPOX2, PIG6, POX1, PRODH2, TP53I6, PRODH | proline dehydrogenase (oxidase) 1 | 22q11.21 | 606810 |
| Homo sapiens | 7916 | PRRC2A | BAT2, D6S51, D6S51E, G2 | proline-rich coiled-coil 2A | 6p21.3 | 142580 |
| Homo sapiens | 10279 | PRSS16 | TSSP | protease, serine, 16 (thymus) | 6p21 | 607169 |
| Homo sapiens | 29968 | PSAT1 | EPIP, NLS2, PSA, PSAT, PSATD | phosphoserine aminotransferase 1 | 9q21.2 | 610936 |
| Homo sapiens | 58155 | PTBP2 | PTBLP, brPTB, nPTB | polypyrimidine tract binding protein 2 | 1p21.3 | 608449 |
| Homo sapiens | 5730 | PTGDS | L-PGDS, LPGDS, PDS, PGD2, PGDS, PGDS2 | prostaglandin D2 synthase 21kDa (brain) | 9q34.2-q34.3 | 176803 |
| Homo sapiens | 5733 | PTGER3 | EP3, EP3-I, EP3-II, EP3-III, EP3-IV, EP3e, PGE2-R | prostaglandin E receptor 3 (subtype EP3) | 1p31.2 | 176806 |
| Homo sapiens | 5742 | PTGS1 | COX1, COX3, PCOX1, PES-1, PGG/HS, PGHS-1, PGHS1, PHS1, PTGHS | prostaglandin-endoperoxide synthase 1 (prostaglandin G/H synthase and cyclooxygenase) | 9q32-q33.3 | 176805 |
| Homo sapiens | 5743 | PTGS2 | COX-2, COX2, GRIPGHS, PGG/HS, PGHS-2, PHS-2, hCox-2 | prostaglandin-endoperoxide synthase 2 (prostaglandin G/H synthase and cyclooxygenase) | 1q25.2-q25.3 | 600262 |
| Homo sapiens | 11099 | PTPN21 | PTPD1, PTPRL10 | protein tyrosine phosphatase, non-receptor type 21 | 14q31.3 | 603271 |
| Homo sapiens | 84867 | PTPN5 | PTPSTEP, STEP | protein tyrosine phosphatase, non-receptor type 5 (striatum-enriched) | 11p15.1 | 176879 |
| Homo sapiens | 5786 | PTPRA | HEPTP, HLPR, HPTPA, HPTPalpha, LRP, PTPA, PTPRL2, R-PTP-alpha, RPTPA | protein tyrosine phosphatase, receptor type, A | 20p13 | 176884 |
| Homo sapiens | 5793 | PTPRG | HPTPG, PTPG, R-PTP-GAMMA, RPTPG | protein tyrosine phosphatase, receptor type, G | 3p21-p14 | 176886 |
| Homo sapiens | 5799 | PTPRN2 | IA-2beta, IAR, ICAAR, PTPRP, R-PTP-N2 | protein tyrosine phosphatase, receptor type, N polypeptide 2 | 7q36 | 601698 |
| Homo sapiens | 10076 | PTPRU | FMI, PCP-2, PTP, PTP-J, PTP-PI, PTP-RO, PTPPSI, PTPRO, PTPU2, R-PTP-PSI, R-PTP-U, hPTP-J | protein tyrosine phosphatase, receptor type, U | 1p35.3 | 602454 |
| Homo sapiens | 5803 | PTPRZ1 | HPTPZ, HPTPzeta, PTP-ZETA, PTP18, PTPRZ, PTPZ, R-PTP-zeta-2, RPTPB, RPTPbeta, phosphacan | protein tyrosine phosphatase, receptor-type, Z polypeptide 1 | 7q31.3 | 176891 |
| Homo sapiens | 5813 | PURA | MRD31, PUR-ALPHA, PUR1LPHA, PURA | purine-rich element binding protein A | 5q31 | 600473 |
| Homo sapiens | 5816 | PVALB | D22S749 | parvalbumin | 22q13.1 | 168890 |
| Homo sapiens | 5834 | PYGB | GPBB | phosphorylase, glycogen; brain | 20p11.21 | 138550 |
| Homo sapiens | 5860 | QDPR | DHPR, PKU2, SDR33C1 | quinoid dihydropteridine reductase | 4p15.31 | 612676 |
| Homo sapiens | 9444 | QKI | Hqk, QK, QK1, QK3, hqkI | QKI, KH domain containing, RNA binding | 6q26 | 609590 |
| Homo sapiens | 25797 | QPCT | GCT, QC, sQC | glutaminyl-peptide cyclotransferase | 2p22.2 | 607065 |
| Homo sapiens | 5902 | RANBP1 | HTF9A | RAN binding protein 1 | 22q11.21 | 601180 |
| Homo sapiens | 5912 | RAP2B |  | RAP2B, member of RAS oncogene family | 3q25.2 | 179541 |
| Homo sapiens | 51735 | RAPGEF6 | KIA001LB, PDZ-GEF2, PDZGEF2, RA-GEF-2, RAGEF2 | Rap guanine nucleotide exchange factor (GEF) 6 | 5q31.1 | 610499 |
| Homo sapiens | 54715 | RBFOX1 | 2BP1, A2BP1, FOX-1, FOX1, HRNBP1 | RNA binding protein, fox-1 homolog (C. elegans) 1 | 16p13.3 | 605104 |
| Homo sapiens | 5967 | REG1A | ICRF, P19, PSP, PSPS, PSPS1, PTP, REG | regenerating islet-derived 1 alpha | 2p12 | 167770 |
| Homo sapiens | 5968 | REG1B | PSPS2, REGH, REGI-BETA, REGL | regenerating islet-derived 1 beta | 2p12 | 167771 |
| Homo sapiens | 130120 | REG3G | LPPM429, PAP IB, PAP-1B, PAP1B, PAPIB, REG III, REG-III, UNQ429 | regenerating islet-derived 3 gamma | 2p12 | 609933 |
| Homo sapiens | 5970 | RELA | NFKB3, p65 | v-rel avian reticuloendotheliosis viral oncogene homolog A | 11q13 | 164014 |
| Homo sapiens | 5649 | RELN | LIS2, PRO1598, RL | reelin | 7q22 | 600514 |
| Homo sapiens | 5973 | RENBP | RBP, RNBP | renin binding protein | Xq28 | 312420 |
| Homo sapiens | 473 | RERE | ARG, ARP, ATN1L, DNB1 | arginine-glutamic acid dipeptide (RE) repeats | 1p36.23 | 605226 |
| Homo sapiens | 91869 | RFT1 | CDG1N | RFT1 homolog (S. cerevisiae) | 3p21.1 | 611908 |
| Homo sapiens | 5990 | RFX2 |  | regulatory factor X, 2 (influences HLA class II expression) | 19p13.3 | 142765 |
| Homo sapiens | 6001 | RGS10 |  | regulator of G-protein signaling 10 | 10q25 | 602856 |
| Homo sapiens | 5997 | RGS2 | G0S8 | regulator of G-protein signaling 2 | 1q31 | 600861 |
| Homo sapiens | 5999 | RGS4 | RGP4, SCZD9 | regulator of G-protein signaling 4 | 1q23.3 | 602516 |
| Homo sapiens | 8490 | RGS5 | MST092, MST106, MST129, MSTP032, MSTP092, MSTP106, MSTP129 | regulator of G-protein signaling 5 | 1q23.1 | 603276 |
| Homo sapiens | 8787 | RGS9 | PERRSL, RGS9 | regulator of G-protein signaling 9 | 17q24 | 604067 |
| Homo sapiens | 6007 | RHD | CD240D, DIIIc, RH, RH30, RHCEDVA(TT), RHDel, RHPII, RHXIII, Rh4, RhDCw, RhII, RhK562-II, RhPI, RHD | Rh blood group, D antigen | 1p36.11 | 111680 |
| Homo sapiens | 196383 | RILPL2 | RLP2 | Rab interacting lysosomal protein-like 2 | 12q24.31 | 614093 |
| Homo sapiens | 9699 | RIMS2 | OBOE, RAB3IP3, RIM2 | regulating synaptic membrane exocytosis 2 | 8q22.3 | 606630 |
| Homo sapiens | 9783 | RIMS3 | NIM3, RIM3 | regulating synaptic membrane exocytosis 3 | 1p34.2 | 611600 |
| Homo sapiens | 54453 | RIN2 | MACS, RASSF4 | Ras and Rab interactor 2 | 20p11.22 | 610222 |
| Homo sapiens | 6017 | RLBP1 | CRALBP | retinaldehyde binding protein 1 | 15q26 | 180090 |
| Homo sapiens | 6048 | RNF5 | RING5, RMA1 | ring finger protein 5, E3 ubiquitin protein ligase | 6p21.3 | 602677 |
| Homo sapiens | 6050 | RNH1 | RAI, RNH | ribonuclease/angiogenin inhibitor 1 | 11p15.5 | 173320 |
| Homo sapiens | 6091 | ROBO1 | DUTT1, SAX3 | roundabout, axon guidance receptor, homolog 1 (Drosophila) | 3p12 | 602430 |
| Homo sapiens | 6092 | ROBO2 | SAX3 | roundabout, axon guidance receptor, homolog 2 (Drosophila) | 3p12.3 | 602431 |
| Homo sapiens | 6093 | ROCK1 | P160ROCK, ROCK-I | Rho-associated, coiled-coil containing protein kinase 1 | 18q11.1 | 601702 |
| Homo sapiens | 4920 | ROR2 | BDB, BDB1, NTRKR2 | receptor tyrosine kinase-like orphan receptor 2 | 9q22 | 602337 |
| Homo sapiens | 6095 | RORA | NR1F1, ROR1, ROR2, ROR3, RZR-ALPHA, RZRA | RAR-related orphan receptor A | 15q22.2 | 600825 |
| Homo sapiens | 6137 | RPL13 | BBC1, D16S444E, D16S44E, L13 | ribosomal protein L13 | 17p11.2 | 113703 |
| Homo sapiens | 6204 | RPS10 | DBA9, S10 | ribosomal protein S10 | 6p21.31 | 603632 |
| Homo sapiens | 51319 | RSRC1 | BM-011, SFRS21, SRrp53 | arginine/serine-rich coiled-coil 1 | 3q25.32 | 613352 |
| Homo sapiens | 388015 | RTL1 | MART1, Mar1, PEG11 | retrotransposon-like 1 | 14q32.2 | 611896 |
| Homo sapiens | 57142 | RTN4 | ASY, NI220/250, NOGO, NOGO-A, NOGOC, NSP, NSP-CL, Nbla00271, Nbla10545, Nogo-B, Nogo-C, RTN-X-A, RTN4-B1, RTN4-B2, RTN4-C, RTN4 | reticulon 4 | 2p16.3 | 604475 |
| Homo sapiens | 65078 | RTN4R | NGR, NOGOR | reticulon 4 receptor | 22q11.21 | 605566 |
| Homo sapiens | 6257 | RXRB | DAUDI6, H-2RIIBP, NR2B2, RCoR-1 | retinoid X receptor, beta | 6p21.3 | 180246 |
| Homo sapiens | 6285 | S100B | NEF, S100, S100-B, S100beta | S100 calcium binding protein B | 21q22.3 | 176990 |
| Homo sapiens | 6289 | SAA2 |  | serum amyloid A2 | 11p15.1-p14 | 104751 |
| Homo sapiens | 100528017 | SAA2-SAA4 |  | SAA2-SAA4 readthrough | 11p15.1 |  |
| Homo sapiens | 6303 | SAT1 | DC21, KFSD, KFSDX, SAT, SSAT, SSAT-1 | spermidine/spermine N1-acetyltransferase 1 | Xp22.1 | 313020 |
| Homo sapiens | 55206 | SBNO1 | MOP3, Sno | strawberry notch homolog 1 (Drosophila) | 12q24.31 | 614274 |
| Homo sapiens | 6309 | SC5D | ERG3, S5DESL, SC5D | sterol-C5-desaturase | 11q23.3 | 602286 |
| Homo sapiens | 6326 | SCN2A | BFIC3, BFIS3, BFNIS, EIEE11, HBA, HBSCI, HBSCII, NAC2, Na(v)1.2, Nav1.21, SCN2A2, SCN2A | sodium channel, voltage gated, type II alpha subunit | 2q24.3 | 182390 |
| Homo sapiens | 9805 | SCRN1 | SES1 | secernin 1 | 7p14.3 | 614965 |
| Homo sapiens | 6377 | SCZD1 |  | schizophrenia disorder 1 | 5q11.2-q13.3 | 181510 |
| Homo sapiens | 63944 | SCZD10 |  | schizophrenia disorder 10 (periodic catatonia) | 15q15 | 605419 |
| Homo sapiens | 404686 | SCZD11 |  | Schizophrenia susceptibility locus, chromosome 10q-related | 10q22.3 | 608078 |
| Homo sapiens | 619488 | SCZD12 |  | schizophrenia 12 | 1p | 608543 |
| Homo sapiens | 100329170 | SCZD13 |  | schizophrenia 13 | 15q13 | 613025 |
| Homo sapiens | 100196913 | SCZD14 |  | schizophrenia, susceptibility to, 14 | 2q32.1 | 612361 |
| Homo sapiens | 6378 | SCZD2 |  | schizophrenia disorder 2 | 11q14-q21 | 603342 |
| Homo sapiens | 6365 | SCZD3 |  | schizophrenia disorder 3 | 6p24-p22 | 600511 |
| Homo sapiens | 8400 | SCZD6 |  | schizophrenia disorder 6 | 8p21 | 603013 |
| Homo sapiens | 8401 | SCZD7 |  | schizophrenia disorder 7 | 13q32 | 603176 |
| Homo sapiens | 8806 | SCZD8 |  | schizophrenia disorder 8 | 18p | 603206 |
| Homo sapiens | 10806 | SDCCAG8 | BBS16, CCCAP, CCCAP SLSN7, HSPC085, NPHP10, NY-CO-8, SLSN7, hCCCAP | serologically defined colon cancer antigen 8 | 1q43 | 613524 |
| Homo sapiens | 89866 | SEC16B | LZTR2, PGPR-p117, RGPR, SEC16S | SEC16 homolog B (S. cerevisiae) | 1q25.2 | 612855 |
| Homo sapiens | 8991 | SELENBP1 | HEL-S-134P, LPSB, SBP56, SP56, hSBP | selenium binding protein 1 | 1q21.3 | 604188 |
| Homo sapiens | 10371 | SEMA3A | COLL1, HH16, Hsema-I, Hsema-III, SEMA1, SEMAD, SEMAIII, SEMAL, SemD, coll-1 | sema domain, immunoglobulin domain (Ig), short basic domain, secreted, (semaphorin) 3A | 7p12.1 | 603961 |
| Homo sapiens | 223117 | SEMA3D | Sema-Z2, coll-2 | sema domain, immunoglobulin domain (Ig), short basic domain, secreted, (semaphorin) 3D | 7q21.11 | 609907 |
| Homo sapiens | 56920 | SEMA3G | sem2 | sema domain, immunoglobulin domain (Ig), short basic domain, secreted, (semaphorin) 3G | 3p21.1 |  |
| Homo sapiens | 12 | SERPINA3 | AACT, ACT, GIG24, GIG25 | serpin peptidase inhibitor, clade A (alpha-1 antiproteinase, antitrypsin), member 3 | 14q32.1 | 107280 |
| Homo sapiens | 9739 | SETD1A | KMT2F, Set1, Set1A | SET domain containing 1A | 16p11.2 | 611052 |
| Homo sapiens | 387893 | SETD8 | KMT5A, PR-Set7, SET07, SET8 | SET domain containing (lysine methyltransferase) 8 | 12q24.31 | 607240 |
| Homo sapiens | 51460 | SFMBT1 | RU1, SFMBT, hSFMBT | Scm-like with four mbt domains 1 | 3p21.1 | 607319 |
| Homo sapiens | 118980 | SFXN2 |  | sideroflexin 2 | 10q24.32 | 615570 |
| Homo sapiens | 137868 | SGCZ | ZSG1 | sarcoglycan, zeta | 8p22 | 608113 |
| Homo sapiens | 6456 | SH3GL2 | CNSA2, EEN-B1, SH3D2A, SH3P4 | SH3-domain GRB2-like 2 | 9p22 | 604465 |
| Homo sapiens | 50944 | SHANK1 | SPANK-1, SSTRIP, synamon | SH3 and multiple ankyrin repeat domains 1 | 19q13.3 | 604999 |
| Homo sapiens | 85358 | SHANK3 | DEL22q13.3, PROSAP2, PSAP2, SCZD15, SPANK-2 | SH3 and multiple ankyrin repeat domains 3 | 22q13.3 | 606230 |
| Homo sapiens | 729993 | SHISA9 | CKAMP44 | shisa family member 9 | 16p13.12 | 613346 |
| Homo sapiens | 10280 | SIGMAR1 | ALS16, OPRS1, SIG-1R, SR-BP, SR-BP1, SRBP, hSigmaR1, sigma1R | sigma non-opioid intracellular receptor 1 | 9p13.3 | 601978 |
| Homo sapiens | 140885 | SIRPA | BIT, CD172A, MFR, MYD-1, P84, PTPNS1, SHPS1, SIRP | signal-regulatory protein alpha | 20p13 | 602461 |
| Homo sapiens | 10326 | SIRPB1 | CD172b, SIRP-BETA-1 | signal-regulatory protein beta 1 | 20p13 | 603889 |
| Homo sapiens | 23411 | SIRT1 | SIR2L1 | sirtuin 1 | 10q21.3 | 604479 |
| Homo sapiens | 23408 | SIRT5 | SIR2L5 | sirtuin 5 | 6p23 | 604483 |
| Homo sapiens | 6504 | SLAMF1 | CD150, CDw150, SLAM | signaling lymphocytic activation molecule family member 1 | 1q23.3 | 603492 |
| Homo sapiens | 6557 | SLC12A1 | BSC1, NKCC2 | solute carrier family 12 (sodium/potassium/chloride transporter), member 1 | 15q15-q21.1 | 600839 |
| Homo sapiens | 6558 | SLC12A2 | BSC, BSC2, NKCC1, PPP1R141 | solute carrier family 12 (sodium/potassium/chloride transporter), member 2 | 5q23.3 | 600840 |
| Homo sapiens | 57468 | SLC12A5 | KCC2 | solute carrier family 12 (potassium/chloride transporter), member 5 | 20q13.12 | 606726 |
| Homo sapiens | 6568 | SLC17A1 | NAPI-1, NPT-1, NPT1 | solute carrier family 17 (organic anion transporter), member 1 | 6p22.2 | 182308 |
| Homo sapiens | 10786 | SLC17A3 | NPT4 | solute carrier family 17 (organic anion transporter), member 3 | 6p21.3 | 611034 |
| Homo sapiens | 57084 | SLC17A6 | DNPI, VGLUT2 | solute carrier family 17 (vesicular glutamate transporter), member 6 | 11p14.3 | 607563 |
| Homo sapiens | 57030 | SLC17A7 | BNPI, VGLUT1 | solute carrier family 17 (vesicular glutamate transporter), member 7 | 19q13 | 605208 |
| Homo sapiens | 6570 | SLC18A1 | CGAT, VAT1, VMAT1 | solute carrier family 18 (vesicular monoamine transporter), member 1 | 8p21.3 | 193002 |
| Homo sapiens | 6571 | SLC18A2 | SVAT, SVMT, VAT2, VMAT2 | solute carrier family 18 (vesicular monoamine transporter), member 2 | 10q25 | 193001 |
| Homo sapiens | 6505 | SLC1A1 | DCBXA, EAAC1, EAAT3, SCZD18 | solute carrier family 1 (neuronal/epithelial high affinity glutamate transporter, system Xag), member 1 | 9p24 | 133550 |
| Homo sapiens | 6506 | SLC1A2 | EAAT2, GLT-1 | solute carrier family 1 (glial high affinity glutamate transporter), member 2 | 11p13-p12 | 600300 |
| Homo sapiens | 6507 | SLC1A3 | EA6, EAAT1, GLAST, GLAST1 | solute carrier family 1 (glial high affinity glutamate transporter), member 3 | 5p13 | 600111 |
| Homo sapiens | 6509 | SLC1A4 | ASCT1, SATT | solute carrier family 1 (glutamate/neutral amino acid transporter), member 4 | 2p15-p13 | 600229 |
| Homo sapiens | 6510 | SLC1A5 | AAAT, ASCT2, ATBO, M7V1, M7VS1, R16, RDRC | solute carrier family 1 (neutral amino acid transporter), member 5 | 19q13.3 | 109190 |
| Homo sapiens | 6511 | SLC1A6 | EAAT4 | solute carrier family 1 (high affinity aspartate/glutamate transporter), member 6 | 19p13.12 | 600637 |
| Homo sapiens | 8604 | SLC25A12 | AGC1, ARALAR | solute carrier family 25 (aspartate/glutamate carrier), member 12 | 2q24 | 603667 |
| Homo sapiens | 9016 | SLC25A14 | BMCP1, UCP5 | solute carrier family 25 (mitochondrial carrier, brain), member 14 | Xq24 | 300242 |
| Homo sapiens | 9481 | SLC25A27 | UCP4 | solute carrier family 25, member 27 | 6p12.3 | 613725 |
| Homo sapiens | 5250 | SLC25A3 | OK/SW-cl.48, PHC, PTP | solute carrier family 25 (mitochondrial carrier; phosphate carrier), member 3 | 12q23 | 600370 |
| Homo sapiens | 115019 | SLC26A9 |  | solute carrier family 26 (anion exchanger), member 9 | 1q32.1 | 608481 |
| Homo sapiens | 2030 | SLC29A1 | ENT1 | solute carrier family 29 (equilibrative nucleoside transporter), member 1 | 6p21.1 | 602193 |
| Homo sapiens | 6513 | SLC2A1 | DYT17, DYT18, DYT9, EIG12, GLUT, GLUT-1, GLUT1, GLUT1DS, HTLVR, PED | solute carrier family 2 (facilitated glucose transporter), member 1 | 1p34.2 |  |
| Homo sapiens | 169026 | SLC30A8 | ZNT8, ZnT-8 | solute carrier family 30 (zinc transporter), member 8 | 8q24.11 | 611145 |
| Homo sapiens | 140679 | SLC32A1 | VGAT, VIAAT | solute carrier family 32 (GABA vesicular transporter), member 1 | 20q11.23 |  |
| Homo sapiens | 341880 | SLC35F4 | C14orf36, c14_5373 | solute carrier family 35, member F4 | 14q22.2 |  |
| Homo sapiens | 6530 | SLC6A2 | NAT1, NET, NET1, SLC6A5 | solute carrier family 6 (neurotransmitter transporter), member 2 | 16q12.2 | 163970 |
| Homo sapiens | 6531 | SLC6A3 | DAT, DAT1, PKDYS | solute carrier family 6 (neurotransmitter transporter), member 3 | 5p15.3 | 126455 |
| Homo sapiens | 6532 | SLC6A4 | 5-HTT, 5-HTTLPR, 5HTT, HTT, OCD1, SERT, SERT1, hSERT | solute carrier family 6 (neurotransmitter transporter), member 4 | 17q11.2 | 182138 |
| Homo sapiens | 9152 | SLC6A5 | GLYT-2, GLYT2, HKPX3, NET1 | solute carrier family 6 (neurotransmitter transporter), member 5 | 11p15.1 | 604159 |
| Homo sapiens | 6536 | SLC6A9 | GLYT1 | solute carrier family 6 (neurotransmitter transporter, glycine), member 9 | 1p33 | 601019 |
| Homo sapiens | 56301 | SLC7A10 | ASC1, HASC-1, asc-1 | solute carrier family 7 (neutral amino acid transporter light chain, asc system), member 10 | 19q13.1 | 607959 |
| Homo sapiens | 9368 | SLC9A3R1 | EBP50, NHERF, NHERF-1, NHERF1, NPHLOP2 | solute carrier family 9, subfamily A (NHE3, cation proton antiporter 3), member 3 regulator 1 | 17q25.1 | 604990 |
| Homo sapiens | 28232 | SLCO3A1 | OATP-D, OATP3A1, OATPD, SLC21A11 | solute carrier organic anion transporter family, member 3A1 | 15q26 | 612435 |
| Homo sapiens | 133482 | SLCO6A1 | CT48, GST, OATP-I, OATP6A1, OATPY | solute carrier organic anion transporter family, member 6A1 | 5q21.1 | 613365 |
| Homo sapiens | 6586 | SLIT3 | MEGF5, SLIL2, SLIT1, Slit-3, slit2 | slit homolog 3 (Drosophila) | 5q35 | 603745 |
| Homo sapiens | 4090 | SMAD5 | DWFC, JV5-1, MADH5 | SMAD family member 5 | 5q31 | 603110 |
| Homo sapiens | 6595 | SMARCA2 | BAF190, BRM, NCBRS, SNF2, SNF2L2, SNF2LA, SWI2, Sth1p, hBRM, hSNF2a | SWI/SNF related, matrix associated, actin dependent regulator of chromatin, subfamily a, member 2 | 9p22.3 | 600014 |
| Homo sapiens | 56916 | SMARCAD1 | ADERM, ETL1, HEL1 | SWI/SNF-related, matrix-associated actin-dependent regulator of chromatin, subfamily a, containing DEAD/H box 1 | 4q22.3 | 612761 |
| Homo sapiens | 23293 | SMG6 | C17orf31, EST1A, SMG-6, hSMG5/7a | SMG6 nonsense mediated mRNA decay factor | 17p13.3 | 610963 |
| Homo sapiens | 6616 | SNAP25 | RIC-4, RIC4, SEC9, SNAP, SNAP-25, bA416N4.2, dJ1068F16.2 | synaptosomal-associated protein, 25kDa | 20p12-p11.2 | 600322 |
| Homo sapiens | 6622 | SNCA | NACP, PARK1, PARK4, PD1 | synuclein, alpha (non A4 component of amyloid precursor) | 4q21 | 163890 |
| Homo sapiens | 692200 | SNORD85 | HBII-251 | small nucleolar RNA, C/D box 85 | 1p35.2 |  |
| Homo sapiens | 6641 | SNTB1 | 59-DAP, A1B, BSYN2, DAPA1B, SNT2, SNT2B1, TIP-43 | syntrophin, beta 1 (dystrophin-associated protein A1, 59kDa, basic component 1) | 8q23-q24 | 600026 |
| Homo sapiens | 399979 | SNX19 | CHET8 | sorting nexin 19 | 11q25 |  |
| Homo sapiens | 92017 | SNX29 | A-388D4.1, RUNDC2A | sorting nexin 29 | 16p13.13-p13.12 |  |
| Homo sapiens | 29886 | SNX8 | Mvp1 | sorting nexin 8 | 7p22.3 | 614905 |
| Homo sapiens | 6647 | SOD1 | ALS, ALS1, HEL-S-44, IPOA, SOD, hSod1, homodimer | superoxide dismutase 1, soluble | 21q22.11 | 147450 |
| Homo sapiens | 6648 | SOD2 | IPOB, MNSOD, MVCD6 | superoxide dismutase 2, mitochondrial | 6q25.3 | 147460 |
| Homo sapiens | 10580 | SORBS1 | CAP, FLAF2, R85FL, SH3D5, SH3P12, SORB1 | sorbin and SH3 domain containing 1 | 10q23.33 | 605264 |
| Homo sapiens | 6663 | SOX10 | DOM, PCWH, WS2E, WS4, WS4C | SRY (sex determining region Y)-box 10 | 22q13.1 | 602229 |
| Homo sapiens | 6667 | SP1 |  | Sp1 transcription factor | 12q13.1 | 189906 |
| Homo sapiens | 6671 | SP4 | HF1B, SPR-1 | Sp4 transcription factor | 7p15.3 | 600540 |
| Homo sapiens | 79582 | SPAG16 | PF20, WDR29 | sperm associated antigen 16 | 2q34 | 612173 |
| Homo sapiens | 26010 | SPATS2L | DNAPTP6, SGNP | spermatogenesis associated, serine-rich 2-like | 2q33.1 | 613817 |
| Homo sapiens | 28972 | SPCS1 | HSPC033, SPC1, SPC12, YJR010C-A | signal peptidase complex subunit 1 homolog (S. cerevisiae) | 3p21.1 | 610358 |
| Homo sapiens | 81848 | SPRY4 | HH17 | sprouty homolog 4 (Drosophila) | 5q31.3 | 607984 |
| Homo sapiens | 6709 | SPTAN1 | EIEE5, NEAS, SPTA2 | spectrin, alpha, non-erythrocytic 1 | 9q34.11 | 182810 |
| Homo sapiens | 10558 | SPTLC1 | HSAN1, HSN1, LBC1, LCB1, SPT1, SPTI | serine palmitoyltransferase, long chain base subunit 1 | 9q22.2 | 605712 |
| Homo sapiens | 6716 | SRD5A2 |  | steroid-5-alpha-reductase, alpha polypeptide 2 (3-oxo-5 alpha-steroid delta 4-dehydrogenase alpha 2) | 2p23 | 607306 |
| Homo sapiens | 6720 | SREBF1 | SREBP-1c, SREBP1, bHLHd1 | sterol regulatory element binding transcription factor 1 | 17p11.2 | 184756 |
| Homo sapiens | 6721 | SREBF2 | SREBP-2, SREBP2, bHLHd2 | sterol regulatory element binding transcription factor 2 | 22q13 | 600481 |
| Homo sapiens | 63826 | SRR | ILV1, ISO1 | serine racemase | 17p13 | 606477 |
| Homo sapiens | 6431 | SRSF6 | B52, HEL-S-91, SFRS6, SRP55 | serine/arginine-rich splicing factor 6 | 20q12-q13.1 | 601944 |
| Homo sapiens | 6432 | SRSF7 | 9G8, AAG3, SFRS7 | serine/arginine-rich splicing factor 7 | 2p22.1 | 600572 |
| Homo sapiens | 6752 | SSTR2 |  | somatostatin receptor 2 | 17q24 | 182452 |
| Homo sapiens | 401648 | ST13P13 |  | suppression of tumorigenicity 13 (colon carcinoma) (Hsp70 interacting protein) pseudogene 13 | 10q24.33 |  |
| Homo sapiens | 6482 | ST3GAL1 | Gal-NAc6S, SIAT4A, SIATFL, ST3GalA, ST3GalA.1, ST3GalIA, ST3GalIA,1, ST3O | ST3 beta-galactoside alpha-2,3-sialyltransferase 1 | 8q24.22 | 607187 |
| Homo sapiens | 8128 | ST8SIA2 | HsT19690, SIAT8B, ST8SIA-II, STX | ST8 alpha-N-acetyl-neuraminide alpha-2,8-sialyltransferase 2 | 15q26 | 602546 |
| Homo sapiens | 23166 | STAB1 | CLEVER-1, FEEL-1, FELE-1, FEX1, STAB-1 | stabilin 1 | 3p21.1 | 608560 |
| Homo sapiens | 246744 | STH | MAPTIT | saitohin | 17q21.1 | 607067 |
| Homo sapiens | 85439 | STON2 | STN2, STNB, STNB2 | stonin 2 | 14q31.1 | 608467 |
| Homo sapiens | 6801 | STRN | SG2NA | striatin, calmodulin binding protein | 2p22.2 | 614765 |
| Homo sapiens | 3703 | STT3A | ITM1, STT3-A, TMC | STT3A, subunit of the oligosaccharyltransferase complex (catalytic) | 11q23.3 | 601134 |
| Homo sapiens | 10273 | STUB1 | CHIP, HSPABP2, NY-CO-7, SCAR16, SDCCAG7, UBOX1 | STIP1 homology and U-box containing protein 1, E3 ubiquitin protein ligase | 16p13.3 | 607207 |
| Homo sapiens | 6804 | STX1A | HPC-1, P35-1, STX1, SYN1A | syntaxin 1A (brain) | 7q11.23 | 186590 |
| Homo sapiens | 6812 | STXBP1 | MUNC18-1, NSEC1, P67, RBSEC1, UNC18 | syntaxin binding protein 1 | 9q34.1 | 602926 |
| Homo sapiens | 57794 | SUGP1 | F23858, RBP, SF4 | SURP and G patch domain containing 1 | 19p13.11 | 607992 |
| Homo sapiens | 25830 | SULT4A1 | BR-STL-1, BRSTL1, DJ388M5.3, NST, SULTX3, hBR-STL-1 | sulfotransferase family 4A, member 1 | 22q13.2 | 608359 |
| Homo sapiens | 391365 | SULT6B1 |  | sulfotransferase family, cytosolic, 6B, member 1 | 2p22.2 |  |
| Homo sapiens | 9900 | SV2A | SV2 | synaptic vesicle glycoprotein 2A | 1q21.2 | 185860 |
| Homo sapiens | 6853 | SYN1 | SYN1ab, SYNI, SYN1 | synapsin I | Xp11.23 | 313440 |
| Homo sapiens | 6854 | SYN2 | SYNII | synapsin II | 3p25 | 600755 |
| Homo sapiens | 8224 | SYN3 |  | synapsin III | 22q12.3 | 602705 |
| Homo sapiens | 8831 | SYNGAP1 | MRD5, RASA1, RASA5, SYNGAP | synaptic Ras GTPase activating protein 1 | 6p21.3 | 603384 |
| Homo sapiens | 9145 | SYNGR1 |  | synaptogyrin 1 | 22q13.1 | 603925 |
| Homo sapiens | 6855 | SYP | MRX96, MRXSYP | synaptophysin | Xp11.23-p11.22 | 313475 |
| Homo sapiens | 23208 | SYT11 | SYT12, sytXI | synaptotagmin XI | 1q21.2 | 608741 |
| Homo sapiens | 6861 | SYT5 |  | synaptotagmin V | 11p | 600782 |
| Homo sapiens | 319100 | TAAR6 | TA4, TAR4, TAR6, TRAR4, taR-4, taR-6 | trace amine associated receptor 6 | 6q23.2 | 608923 |
| Homo sapiens | 6869 | TACR1 | NK1R, NKIR, SPR, TAC1R | tachykinin receptor 1 | 2p12 | 162323 |
| Homo sapiens | 6870 | TACR3 | HH11, NK-3R, NK3R, NKR, TAC3RL | tachykinin receptor 3 | 4q25 | 162332 |
| Homo sapiens | 6877 | TAF5 | TAF2D, TAFII100 | TAF5 RNA polymerase II, TATA box binding protein (TBP)-associated factor, 100kDa | 10q24-q25.2 | 601787 |
| Homo sapiens | 29114 | TAGLN3 | NP22, NP24, NP25 | transgelin 3 | 3q13.2 | 607953 |
| Homo sapiens | 6886 | TAL1 | SCL, TCL5, bHLHa17, tal-1 | T-cell acute lymphocytic leukemia 1 | 1p32 | 187040 |
| Homo sapiens | 445347 | TARP | CD3GTCRG, TCRGC1, TCRGC2 | TCR gamma alternate reading frame protein | 7p15-p14 | 609642 |
| Homo sapiens | 5726 | TAS2R38 | PTC, T2R38, T2R61 | taste receptor, type 2, member 38 | 7q34 | 607751 |
| Homo sapiens | 1155 | TBCB | CG22, CKAP1, CKAPI | tubulin folding cofactor B | 19q13.11-q13.12 | 601303 |
| Homo sapiens | 6908 | TBP | GTF2D, GTF2D1, HDL4, SCA17, TFIID | TATA box binding protein | 6q27 | 600075 |
| Homo sapiens | 6899 | TBX1 | CAFS, CATCH22, CTHM, DGCR, DGS, DORVC, TGA, VCF, VCFS, TBX1 | T-box 1 | 22q11.21 | 602054 |
| Homo sapiens | 6916 | TBXAS1 | BDPLT14, CYP5, CYP5A1, GHOSAL, THAS, TS, TXAS, TXS | thromboxane A synthase 1 (platelet) | 7q34-q35 | 274180 |
| Homo sapiens | 6925 | TCF4 | E2-2, ITF-2, ITF2, PTHS, SEF-2, SEF2, SEF2-1, SEF2-1A, SEF2-1B, SEF2-1D, TCF-4, bHLHb19 | transcription factor 4 | 18q21.1 | 602272 |
| Homo sapiens | 6934 | TCF7L2 | TCF-4, TCF4 | transcription factor 7-like 2 (T-cell specific, HMG-box) | 10q25.3 | 602228 |
| Homo sapiens | 55714 | TENM3 | MCOPCB9, ODZ3, TNM3, Ten-m3 | teneurin transmembrane protein 3 | 4q35.1 | 610083 |
| Homo sapiens | 80312 | TET1 | CXXC6, LCX, bA119F7.1 | tet methylcytosine dioxygenase 1 | 10q21 | 607790 |
| Homo sapiens | 7018 | TF | PRO1557, PRO2086QTL1, TF | transferrin | 3q22.1 | 190000 |
| Homo sapiens | 7021 | TFAP2B | AP-2B, AP2-B | transcription factor AP-2 beta (activating enhancer binding protein 2 beta) | 6p12 | 601601 |
| Homo sapiens | 7040 | TGFB1 | CED, DPD1, LAP, TGFB, TGFbeta | transforming growth factor, beta 1 | 19q13.1 | 190180 |
| Homo sapiens | 7045 | TGFBI | BIGH3, CDB1, CDG2, CDGG1, CSD, CSD1, CSD2, CSD3, EBMD, LCD1 | transforming growth factor, beta-induced, 68kDa | 5q31 | 601692 |
| Homo sapiens | 7048 | TGFBR2 | AAT3, FAA3, LDS1B, LDS2, LDS2B, MFS2, RIIC, TAAD2, TGFR-2, TGFbeta-RII | transforming growth factor, beta receptor II (70/80kDa) | 3p22 | 190182 |
| Homo sapiens | 7052 | TGM2 | G-ALPHA-h, GNAH, HEL-S-45, TG2, TGC | transglutaminase 2 | 20q12 | 190196 |
| Homo sapiens | 7054 | TH | DYT14, DYT5b, TYH | tyrosine hydroxylase | 11p15.5 | 191290 |
| Homo sapiens | 7057 | THBS1 | THBS, THBS-1, TSP, TSP-1, TSP1 | thrombospondin 1 | 15q15 | 188060 |
| Homo sapiens | 10469 | TIMM44 | TIM44 | translocase of inner mitochondrial membrane 44 homolog (yeast) | 19p13.2 | 605058 |
| Homo sapiens | 7079 | TIMP4 |  | TIMP metallopeptidase inhibitor 4 | 3p25 | 601915 |
| Homo sapiens | 7082 | TJP1 | ZO-1 | tight junction protein 1 | 15q13 | 601009 |
| Homo sapiens | 7086 | TKT | HEL107, TK1, TKT | transketolase | 3p14.3 | 606781 |
| Homo sapiens | 7097 | TLR2 | CD282, TIL4 | toll-like receptor 2 | 4q32 | 603028 |
| Homo sapiens | 54106 | TLR9 | CD289 | toll-like receptor 9 | 3p21.3 | 605474 |
| Homo sapiens | 53345 | TM6SF2 |  | transmembrane 6 superfamily member 2 | 19p13.3-p12 | 606563 |
| Homo sapiens | 375346 | TMEM110 |  | transmembrane protein 110 | 3p21.1 |  |
| Homo sapiens | 389177 | TMEM212 |  | transmembrane protein 212 | 3q26.31 |  |
| Homo sapiens | 23731 | TMEM245 | C9orf5, CG-2, CG2 | transmembrane protein 245 | 9q31 |  |
| Homo sapiens | 83857 | TMTC1 | ARG99, OLFA, TMTC1 | transmembrane and tetratricopeptide repeat containing 1 | 12p11.22 | 615855 |
| Homo sapiens | 7124 | TNF | DIF-alpha, TNFA, TNFSF2, TNF | tumor necrosis factor | 6p21.3 | 191160 |
| Homo sapiens | 7132 | TNFRSF1A | CD120a, FPF, MS5, TBP1, TNF-R, TNF-R-I, TNF-R55, TNFAR, TNFR1, TNFR1-d2, TNFR55, TNFR60, p55, p55-R, p60 | tumor necrosis factor receptor superfamily, member 1A | 12p13.2 | 191190 |
| Homo sapiens | 7133 | TNFRSF1B | CD120b, TBPII, TNF-R-II, TNF-R75, TNFBR, TNFR1B, TNFR2, TNFR80, p75, p75TNFR | tumor necrosis factor receptor superfamily, member 1B | 1p36.22 | 191191 |
| Homo sapiens | 23043 | TNIK |  | TRAF2 and NCK interacting kinase | 3q26.31 | 610005 |
| Homo sapiens | 7134 | TNNC1 | CMD1Z, CMH13, TN-C, TNC, TNNC | troponin C type 1 (slow) | 3p21.1 | 191040 |
| Homo sapiens | 7148 | TNXB | EDS3, HXBL, TENX, TN-X, TNX1, TNXB2, TNXBS, VUR8, XB, XBS, TNXB | tenascin XB | 6p21.3 | 600985 |
| Homo sapiens | 7157 | TP53 | BCC7, LFS1, P53, TRP53 | tumor protein p53 | 17p13.1 | 191170 |
| Homo sapiens | 7166 | TPH1 | TPRH, TRPH | tryptophan hydroxylase 1 | 11p15.3-p14 | 191060 |
| Homo sapiens | 121278 | TPH2 | ADHD7, NTPH | tryptophan hydroxylase 2 | 12q21.1 | 607478 |
| Homo sapiens | 7167 | TPI1 | HEL-S-49, TIM, TPI, TPID | triosephosphate isomerase 1 | 12p13 | 190450 |
| Homo sapiens | 7168 | TPM1 | C15orf13, CMD1Y, CMH3, HTM-alpha, LVNC9, TMSA | tropomyosin 1 (alpha) | 15q22.1 | 191010 |
| Homo sapiens | 7169 | TPM2 | AMCD1, DA1, DA2B, HEL-S-273, NEM4, TMSB | tropomyosin 2 (beta) | 9p13 | 190990 |
| Homo sapiens | 7170 | TPM3 | CAPM1, CFTD, HEL-189, HEL-S-82p, NEM1, OK/SW-cl.5, TM-5, TM3, TM30, TM30nm, TM5, TPMsk3, TRK, hscp30 | tropomyosin 3 | 1q21.2 | 191030 |
| Homo sapiens | 7171 | TPM4 | HEL-S-108 | tropomyosin 4 | 19p13.1 | 600317 |
| Homo sapiens | 51673 | TPPP3 | CGI-38, TPPP/p20, p20, p25gamma | tubulin polymerization-promoting protein family member 3 | 16q22.1 |  |
| Homo sapiens | 7187 | TRAF3 | CAP-1, CAP1, CD40bp, CRAF1, IIAE5, LAP1 | TNF receptor-associated factor 3 | 14q32.32 | 601896 |
| Homo sapiens | 9881 | TRANK1 | LBA1 | tetratricopeptide repeat and ankyrin repeat containing 1 | 3p22.2 |  |
| Homo sapiens | 100189401 | TRI-AAT7-2 | TRNAI25 | transfer RNA-Ile (AAT) 7-2 |  |  |
| Homo sapiens | 7726 | TRIM26 | AFP, RNF95, ZNF173 | tripartite motif containing 26 | 6p21.3 | 600830 |
| Homo sapiens | 10612 | TRIM3 | BERP, HAC1, RNF22, RNF97 | tripartite motif containing 3 | 11p15.5 | 605493 |
| Homo sapiens | 27037 | TRMT2A | HTF9C | tRNA methyltransferase 2 homolog A (S. cerevisiae) | 22q11.21 | 611151 |
| Homo sapiens | 203062 | TSNARE1 |  | t-SNARE domain containing 1 | 8q24.3 |  |
| Homo sapiens | 7257 | TSNAX | TRAX | translin-associated factor X | 1q42.1 | 602964 |
| Homo sapiens | 100303453 | TSNAX-DISC1 |  | TSNAX-DISC1 readthrough (NMD candidate) | 1q42.1 |  |
| Homo sapiens | 90139 | TSPAN18 | TSPAN | tetraspanin 18 | 11p11.2 |  |
| Homo sapiens | 83983 | TSSK6 | CT72, FKSG82, SSTK, TSSK4 | testis-specific serine kinase 6 | 19p13.11 | 610712 |
| Homo sapiens | 158219 | TTC39B | C9orf52 | tetratricopeptide repeat domain 39B | 9p22.3 | 613574 |
| Homo sapiens | 283237 | TTC9C |  | tetratricopeptide repeat domain 9C | 11q12.3 |  |
| Homo sapiens | 284076 | TTLL6 | TTL.6 | tubulin tyrosine ligase-like family member 6 | 17q21.32 | 610849 |
| Homo sapiens | 7276 | TTR | CTS, CTS1, HEL111, HsT2651, PALB, TBPA | transthyretin | 18q12.1 | 176300 |
| Homo sapiens | 7846 | TUBA1A | B-ALPHA-1, LIS3, TUBA3 | tubulin, alpha 1a | 12q13.12 | 602529 |
| Homo sapiens | 10376 | TUBA1B | K-ALPHA-1 | tubulin, alpha 1b | 12q13.12 | 602530 |
| Homo sapiens | 203068 | TUBB | CDCBM6, M40, OK/SW-cl.561, TUBB5, TUBB | tubulin, beta class I | 6p21.33 | 191130 |
| Homo sapiens | 7280 | TUBB2A | CDCBM5, TUBB, TUBB2, dJ40E16.7 | tubulin, beta 2A class IIa | 6p25 | 615101 |
| Homo sapiens | 347733 | TUBB2B | PMGYSA, bA506K6.1 | tubulin, beta 2B class IIb | 6p25 | 612850 |
| Homo sapiens | 10381 | TUBB3 | CDCBM, CDCBM1, CFEOM3, CFEOM3A, FEOM3, TUBB4, beta-4 | tubulin, beta 3 class III | 16q24.3 | 602661 |
| Homo sapiens | 10383 | TUBB4B | Beta2, TUBB2, TUBB2C | tubulin, beta 4B class IVb | 9q34 | 602660 |
| Homo sapiens | 7991 | TUSC3 | D8S1992, M33, MRT22, MRT7, N33, OST3A | tumor suppressor candidate 3 | 8p22 | 601385 |
| Homo sapiens | 11344 | TWF2 | A6RP, A6r, MSTP011, PTK9L | twinfilin actin-binding protein 2 | 3p21.1 | 607433 |
| Homo sapiens | 7295 | TXN | TRDX, TRX, TRX1 | thioredoxin | 9q31 | 187700 |
| Homo sapiens | 129450 | TYW5 | C2orf60, hTYW5 | tRNA-yW synthesizing protein 5 | 2q33.1 |  |
| Homo sapiens | 7334 | UBE2N | HEL-S-71, UBC13, UBCHBEN; UBC13, UbcH-ben, UbcH13 | ubiquitin-conjugating enzyme E2N | 12q22 | 603679 |
| Homo sapiens | 7345 | UCHL1 | HEL-117, NDGOA, PARK5, PGP 9.5, PGP9.5, PGP95, Uch-L1 | ubiquitin carboxyl-terminal esterase L1 (ubiquitin thiolesterase) | 4p14 | 191342 |
| Homo sapiens | 7351 | UCP2 | BMIQ4, SLC25A8, UCPH | uncoupling protein 2 (mitochondrial, proton carrier) | 11q13 | 601693 |
| Homo sapiens | 7353 | UFD1L | UFD1 | ubiquitin fusion degradation 1 like (yeast) | 22q11.21 | 601754 |
| Homo sapiens | 54658 | UGT1A1 | BILIQTL1, GNT1, HUG-BR1, UDPGT, UDPGT 1-1, UGT1, UGT1A | UDP glucuronosyltransferase 1 family, polypeptide A1 | 2q37 | 191740 |
| Homo sapiens | 127933 | UHMK1 | KIS, KIST, P-CIP2 | U2AF homology motif (UHM) kinase 1 | 1q23.3 | 608849 |
| Homo sapiens | 54986 | ULK4 | FAM7C1, REC01035 | unc-51 like kinase 4 | 3p22.1 |  |
| Homo sapiens | 7384 | UQCRC1 | D3S3191, QCR1, UQCR1 | ubiquinol-cytochrome c reductase core protein I | 3p21.3 | 191328 |
| Homo sapiens | 84833 | USMG5 | DAPIT, HCVFTP2, bA792D24.4 | up-regulated during skeletal muscle growth 5 homolog (mouse) | 10q24.33 | 615204 |
| Homo sapiens | 219333 | USP12 | UBH1L1, USP12 | ubiquitin specific peptidase 12 | 13q12.13 |  |
| Homo sapiens | 64854 | USP46 |  | ubiquitin specific peptidase 46 | 4q12 | 612849 |
| Homo sapiens | 10911 | UTS2 | PRO1068, U-II, UCN2, UII | urotensin 2 | 1p36 | 604097 |
| Homo sapiens | 6844 | VAMP2 | SYB2, VAMP-2 | vesicle-associated membrane protein 2 (synaptobrevin 2) | 17p13.1 | 185881 |
| Homo sapiens | 9217 | VAPB | ALS8, VAMP-B, VAP-B | VAMP (vesicle-associated membrane protein)-associated protein B and C | 20q13.33 | 605704 |
| Homo sapiens | 57687 | VAT1L |  | vesicle amine transport 1-like | 16q23.1 |  |
| Homo sapiens | 10451 | VAV3 |  | vav 3 guanine nucleotide exchange factor | 1p13.3 | 605541 |
| Homo sapiens | 7422 | VEGFA | MVCD1, VEGF, VPF | vascular endothelial growth factor A | 6p12 | 192240 |
| Homo sapiens | 7431 | VIM | CTRCT30, HEL113 | vimentin | 10p13 | 193060 |
| Homo sapiens | 7434 | VIPR2 | C16DUPq36.3, DUP7q36.3, PACAP-R-3, PACAP-R3, VIP-R-2, VPAC2, VPAC2R, VPCAP2R | vasoactive intestinal peptide receptor 2 | 7q36.3 | 601970 |
| Homo sapiens | 7436 | VLDLR | CAMRQ1, CARMQ1, CHRMQ1CH, VLDLR | very low density lipoprotein receptor | 9p24 | 192977 |
| Homo sapiens | 54832 | VPS13C |  | vacuolar protein sorting 13 homolog C (S. cerevisiae) | 15q22.2 | 608879 |
| Homo sapiens | 7444 | VRK2 |  | vaccinia related kinase 2 | 2p16.1 | 602169 |
| Homo sapiens | 7447 | VSNL1 | HLP3, HPCAL3, HUVISL1, VILIP, VILIP-1 | visinin-like 1 | 2p24.3 | 600817 |
| Homo sapiens | 7450 | VWF | F8VWF, VWD | von Willebrand factor | 12p13.3 | 613160 |
| Homo sapiens | 54838 | WBP1L | C10orf26, OPA1L, OPAL1 | WW domain binding protein 1-like | 10q24.32 | 611129 |
| Homo sapiens | 9948 | WDR1 | AIP1, HEL-S-52, NORI-1 | WD repeat domain 1 | 4p16.1 | 604734 |
| Homo sapiens | 80335 | WDR82 | MST107, MSTP107, PRO2730, PRO34047, SWD2, TMEM113A, WDR82 | WD repeat domain 82 | 3p21.2 | 611059 |
| Homo sapiens | 54904 | WHSC1L1 | NSD3, pp14328 | Wolf-Hirschhorn syndrome candidate 1-like 1 | 8p11.2 | 607083 |
| Homo sapiens | 65267 | WNK3 | PRKWNK3 | WNK lysine deficient protein kinase 3 | Xp11.22 | 300358 |
| Homo sapiens | 7472 | WNT2 | INT1L1, IRP | wingless-type MMTV integration site family member 2 | 7q31.2 | 147870 |
| Homo sapiens | 51741 | WWOX | D16S432E, EIEE28, FOR, FRA16D, HHCMA56, PRO0128, SCAR12, SDR41C1, WOX1 | WW domain containing oxidoreductase | 16q23 | 605131 |
| Homo sapiens | 7494 | XBP1 | TREB-5, TREB5, XBP-1, XBP2 | X-box binding protein 1 | 22q12 | 194355 |
| Homo sapiens | 7504 | XK | KX, MCLDS, NA, NAC, X1kR1, XK | X-linked Kx blood group | Xp21.1 | 314850 |
| Homo sapiens | 7515 | XRCC1 | RCC | X-ray repair complementing defective repair in Chinese hamster cells 1 | 19q13.2 | 194360 |
| Homo sapiens | 7518 | XRCC4 |  | X-ray repair complementing defective repair in Chinese hamster cells 4 | 5q14.2 | 194363 |
| Homo sapiens | 374887 | YJEFN3 |  | YjeF N-terminal domain containing 3 | 19p13.11 |  |
| Homo sapiens | 7529 | YWHAB | GW128, HEL-S-1, HS1, KCIP-1, YWHAA | tyrosine 3-monooxygenase/tryptophan 5-monooxygenase activation protein, beta | 20q13.1 | 601289 |
| Homo sapiens | 7531 | YWHAE | 14-3-3E, HEL2, KCIP-1, MDCR, MDS | tyrosine 3-monooxygenase/tryptophan 5-monooxygenase activation protein, epsilon | 17p13.3 | 605066 |
| Homo sapiens | 7532 | YWHAG | 14-3-3GAMMA, PPP1R170 | tyrosine 3-monooxygenase/tryptophan 5-monooxygenase activation protein, gamma | 7q11.23 | 605356 |
| Homo sapiens | 7533 | YWHAH | YWHA1 | tyrosine 3-monooxygenase/tryptophan 5-monooxygenase activation protein, eta | 22q12.3 | 113508 |
| Homo sapiens | 7534 | YWHAZ | 14-3-3-zeta, HEL-S-3, HEL4, KCIP-1, YWHAD | tyrosine 3-monooxygenase/tryptophan 5-monooxygenase activation protein, zeta | 8q23.1 | 601288 |
| Homo sapiens | 7528 | YY1 | DELTA, INO80S, NF-E1, UCRBP, YIN-YANG-1 | YY1 transcription factor | 14q | 600013 |
| Homo sapiens | 23091 | ZC3H13 | KIAA0853 | zinc finger CCCH-type containing 13 | 13q14.13 |  |
| Homo sapiens | 57683 | ZDBF2 |  | zinc finger, DBF-type containing 2 | 2q33.3 |  |
| Homo sapiens | 29801 | ZDHHC8 | DHHC8, ZDHHCL1, ZNF378 | zinc finger, DHHC-type containing 8 | 22q11.21 | 608784 |
| Homo sapiens | 9839 | ZEB2 | HSPC082, SIP-1, SIP1, SMADIP1, ZFHX1B | zinc finger E-box binding homeobox 2 | 2q22.3 | 605802 |
| Homo sapiens | 146198 | ZFP90 | FIK, NK10, ZNF756, zfp-90 | ZFP90 zinc finger protein | 16q22.1 | 609451 |
| Homo sapiens | 57732 | ZFYVE28 | LST2, LYST2 | zinc finger, FYVE domain containing 28 | 4p16.3 | 614176 |
| Homo sapiens | 387032 | ZKSCAN4 | P1P373C6, ZNF307, ZNF427, ZSCAN36 | zinc finger with KRAB and SCAN domains 4 | 6p21 | 611643 |
| Homo sapiens | 7678 | ZNF124 | HZF-16, HZF16, ZK7 | zinc finger protein 124 | 1q44 | 194631 |
| Homo sapiens | 7738 | ZNF184 |  | zinc finger protein 184 | 6p21.3 | 602277 |
| Homo sapiens | 22891 | ZNF365 | Su48, UAND, ZNF365 | zinc finger protein 365 | 10q21.2 | 607818 |
| Homo sapiens | 147657 | ZNF480 |  | zinc finger protein 480 | 19q13.41 | 613910 |
| Homo sapiens | 51123 | ZNF706 | HSPC038, PNAS-106, PNAS-113 | zinc finger protein 706 | 8q22.3 |  |
| Homo sapiens | 91752 | ZNF804A | C2orf10 | zinc finger protein 804A | 2q32.1 | 612282 |
| Homo sapiens | 57688 | ZSWIM6 | AFND | zinc finger, SWIM-type containing 6 | 5q12.1 | 615951 |
| Homo sapiens | 23140 | ZZEF1 | ZZZ4 | zinc finger, ZZ-type with EF-hand domain 1 | 17p13.2 |  |

**Supplementary Table S2** Quality-Evaluation Score (Q-E Score) (ver2.0)

Please read the following text and try to answer the questions:

| Code | Items | Scores | Guides for scoring instruments |
| --- | --- | --- | --- |
| Q1 | Study design | 2 | One point if the objects in the study were ethnically matched between cases and controls. One additional point if the strict experimental and statistical methods were described. Zero if “No”. |
| Q2 | Gender | 2 | Two points if cases and controls matched; zero if not mentioned or “No”. |
| Q3 | Age | 2 | Two points if cases and controls matched; zero if not mentioned or “No”. |
| Q4 | Diagnostic criteria | 1 | One point if using the acknowledged operational diagnostic criteria; zero if “No”. |
| Q5 | Hardy-Weinberg equilibrium (HWE) | 1 | One point if data were in HWE (p>0.05); zero if p<0.05. |
| Q6 | Publishing journal | 2 | Two points if the journal is a peer review one with an impact factor in the last five years; zero if “No”. |
|  | Total score | 10 |  |

A score of 0-4 indicates poor quality; a score of 5-7 indicates medium quality; a score of 8-10 indicates high quality. This procedure included five items and was produced based on thousands of case-control association studies and polls among dozens of association specialists.

**Supplementary Table S3**  Gene list of common genes between the expanded ATDH and Schizophrenia genes

| Gene symbol |
| --- |
| AANAT |
| ABAT |
| ABCA1 |
| ABCB1 |
| ABCB4 |
| ABCC1 |
| ACACA |
| ACACB |
| ACE |
| ACHE |
| ACSL4 |
| ACTN2 |
| ACVR2A |
| ADA |
| ADAM15 |
| ADCY1 |
| ADCY2 |
| ADCY4 |
| ADCY5 |
| ADCY6 |
| ADCY7 |
| ADCY8 |
| ADCY9 |
| ADH1A |
| ADH1B |
| ADH4 |
| ADH5 |
| ADH6 |
| ADH7 |
| ADIPOQ |
| ADM |
| ADORA1 |
| ADORA2A |
| ADRA2A |
| ADRA2B |
| ADRA2C |
| ADRB1 |
| ADRB2 |
| ADRB3 |
| ADRBK1 |
| ADRBK2 |
| ADSS |
| AGER |
| AGRP |
| AGT |
| AGTR1 |
| AGTR2 |
| AGXT |
| AHCY |
| AKAP5 |
| AKAP9 |
| AKR1C4 |
| AKR1D1 |
| AKT1 |
| AKT2 |
| ALB |
| ALDH1A1 |
| ALDH1A2 |
| ALDH1A3 |
| ALDH2 |
| ALDH3A1 |
| ALDH3A2 |
| ALDH5A1 |
| ALDH9A1 |
| ALDOA |
| ALK |
| ALOX12 |
| ALOX5 |
| AMBP |
| AMT |
| AOX1 |
| AP2A1 |
| AP2M1 |
| APEX1 |
| APOA1 |
| APOA2 |
| APOA4 |
| APOA5 |
| APOB |
| APOC3 |
| APOD |
| APOE |
| APP |
| AR |
| ARHGAP32 |
| ARHGAP4 |
| ARHGDIA |
| ARHGEF11 |
| ARNTL |
| ARRB2 |
| AS3MT |
| ASCL1 |
| ASPM |
| ATF2 |
| ATF4 |
| ATIC |
| ATM |
| ATXN3 |
| AURKB |
| AVPR1A |
| AVPR2 |
| AXIN1 |
| B2M |
| BAD |
| BAG3 |
| BCAN |
| BCAR1 |
| BCHE |
| BCL2 |
| BCL2L1 |
| BDKRB2 |
| BDNF |
| BGN |
| BHLHE41 |
| BIK |
| BMP2 |
| BMPR2 |
| BNIP3L |
| BRCA1 |
| BSG |
| BTG2 |
| BTRC |
| C1QA |
| C1QB |
| C3 |
| C5AR1 |
| CABIN1 |
| CAD |
| CALCA |
| CALCR |
| CALM1 |
| CALM2 |
| CALM3 |
| CAMK2A |
| CAMK2B |
| CAMK2G |
| CANX |
| CAPN10 |
| CARM1 |
| CASK |
| CASP3 |
| CASR |
| CAT |
| CBL |
| CBS |
| CCK |
| CCKAR |
| CCKBR |
| CCL2 |
| CCNB1 |
| CCND1 |
| CCR3 |
| CCR5 |
| CD14 |
| CD4 |
| CD40 |
| CD46 |
| CD47 |
| CDC25A |
| CDC42 |
| CDH1 |
| CDH2 |
| CDK2 |
| CDK5 |
| CDKN1A |
| CDKN2A |
| CEBPB |
| CFTR |
| CHD4 |
| CHEK1 |
| CHRM1 |
| CHRM2 |
| CHRM3 |
| CHRM4 |
| CKAP5 |
| CLOCK |
| CLU |
| CNTF |
| COMT |
| CORO1A |
| CORT |
| CPLX1 |
| CREB1 |
| CREBBP |
| CREM |
| CRH |
| CRK |
| CRP |
| CRY1 |
| CRY2 |
| CSF2 |
| CSF2RA |
| CSF2RB |
| CSNK1D |
| CSNK1E |
| CSNK2A1 |
| CTCF |
| CTH |
| CTLA4 |
| CTNNA1 |
| CTNNB1 |
| CTNND1 |
| CTSD |
| CXCL1 |
| CXCL16 |
| CXCR2 |
| CXCR4 |
| CYBA |
| CYP11B1 |
| CYP17A1 |
| CYP1A1 |
| CYP1A2 |
| CYP26A1 |
| CYP26B1 |
| CYP26C1 |
| CYP2A6 |
| CYP2B6 |
| CYP2C18 |
| CYP2C19 |
| CYP2C8 |
| CYP2C9 |
| CYP2D6 |
| CYP2E1 |
| CYP2J2 |
| CYP3A4 |
| CYP3A43 |
| CYP3A5 |
| CYP3A7 |
| CYP4A11 |
| CYP4A22 |
| DAB2 |
| DAG1 |
| DARC |
| DBH |
| DCC |
| DCN |
| DDC |
| DGCR8 |
| DGKG |
| DGKH |
| DGKI |
| DGKZ |
| DHFR |
| DKK1 |
| DLG3 |
| DMD |
| DNM2 |
| DNMT1 |
| DNMT3A |
| DNMT3B |
| DPYSL2 |
| DRD2 |
| DRD3 |
| DRD4 |
| DROSHA |
| DUSP1 |
| DUSP6 |
| DVL1 |
| DVL2 |
| EDN1 |
| EDNRB |
| EFNB2 |
| EGF |
| EGFR |
| EGR1 |
| EGR2 |
| EGR3 |
| EIF4E |
| ELOVL2 |
| ELOVL5 |
| ELP3 |
| ENPP1 |
| ENTPD4 |
| EP300 |
| EPHA6 |
| EPHB1 |
| EPHB2 |
| EPHX2 |
| EPO |
| EPOR |
| EPS15 |
| ERBB2 |
| ERBB2IP |
| ERBB3 |
| ERBB4 |
| ESR1 |
| ESR2 |
| EZH2 |
| F2R |
| FADD |
| FADS1 |
| FADS2 |
| FAH |
| FAS |
| FASLG |
| FASN |
| FBP1 |
| FCAR |
| FGF1 |
| FGF2 |
| FGF8 |
| FGFR1 |
| FGFR2 |
| FKBP1A |
| FMO3 |
| FN1 |
| FOS |
| FOXA1 |
| FOXO1 |
| FSHR |
| FURIN |
| FYN |
| GAD1 |
| GAD2 |
| GADD45A |
| GAL |
| GALR1 |
| GALR3 |
| GANAB |
| GAP43 |
| GAPDH |
| GAST |
| GATA2 |
| GCG |
| GCGR |
| GCLC |
| GCLM |
| GDNF |
| GFAP |
| GFRA1 |
| GFRA2 |
| GGT1 |
| GH1 |
| GHRH |
| GHRL |
| GIPR |
| GJA1 |
| GLI2 |
| GLO1 |
| GLP1R |
| GLS |
| GLS2 |
| GLUD1 |
| GLUD2 |
| GLUL |
| GMPS |
| GNA13 |
| GNA15 |
| GNAI2 |
| GNAS |
| GNB2L1 |
| GNG2 |
| GNRH1 |
| GOT1 |
| GOT2 |
| GPC1 |
| GPC2 |
| GPC6 |
| GPR17 |
| GPR55 |
| GPX1 |
| GPX3 |
| GRB10 |
| GRB2 |
| GRIN2A |
| GRIP1 |
| GRK6 |
| GRP |
| GRPR |
| GSK3A |
| GSK3B |
| GSS |
| GSTA1 |
| GSTA2 |
| GSTA3 |
| GSTA4 |
| GSTM1 |
| GSTM2 |
| GSTM3 |
| GSTO1 |
| GSTP1 |
| GSTT1 |
| GSTT2B |
| GSTZ1 |
| GTF2I |
| GYS2 |
| H3F3A |
| H3F3B |
| HBEGF |
| HCK |
| HCRT |
| HDAC1 |
| HDAC10 |
| HDAC11 |
| HDAC2 |
| HDAC3 |
| HDAC4 |
| HDAC9 |
| HDC |
| HEXB |
| HFE |
| HIF1A |
| HLA-A |
| HLA-B |
| HLA-C |
| HLA-DRB1 |
| HMGA2 |
| HNF4A |
| HNMT |
| HP |
| HRAS |
| HRH3 |
| HRH4 |
| HSD11B1 |
| HSD11B2 |
| HSD3B1 |
| HSD3B2 |
| HSP90AA1 |
| HSP90B1 |
| HSPA4 |
| HSPA5 |
| HSPA8 |
| HSPG2 |
| HTR1A |
| HTR1B |
| HTR1D |
| HTR1E |
| HTR1F |
| HTR4 |
| HTR5A |
| HTT |
| ICAM1 |
| IDO1 |
| IFNG |
| IGF1 |
| IGF1R |
| IGFBP3 |
| IL10 |
| IL10RA |
| IL12A |
| IL12B |
| IL12RB1 |
| IL13 |
| IL18 |
| IL18RAP |
| IL19 |
| IL1A |
| IL1B |
| IL1R1 |
| IL1RN |
| IL2 |
| IL23A |
| IL2RB |
| IL3 |
| IL3RA |
| IL4 |
| IL4I1 |
| IL4R |
| IL5 |
| IL6 |
| IL6R |
| IL8 |
| IL9 |
| IMPDH2 |
| INPP5D |
| INS-IGF2 |
| INSIG1 |
| INSIG2 |
| INSR |
| IRS1 |
| IRS2 |
| IRS4 |
| ITGA3 |
| ITGA4 |
| ITGA6 |
| ITGAV |
| ITGB1 |
| ITGB2 |
| ITGB4 |
| ITIH1 |
| ITIH3 |
| ITPR1 |
| JAK1 |
| JAK2 |
| JUN |
| JUP |
| KAT2B |
| KCNA5 |
| KCNQ1 |
| KLF5 |
| KNG1 |
| KSR1 |
| L1CAM |
| LCK |
| LEF1 |
| LEP |
| LEPR |
| LHCGR |
| LIF |
| LIFR |
| LPCAT1 |
| LPCAT2 |
| LPL |
| LRP1 |
| LRP6 |
| LRP8 |
| MAOA |
| MAOB |
| MAP1B |
| MAP2K3 |
| MAP2K6 |
| MAP3K3 |
| MAPK1 |
| MAPK11 |
| MAPK14 |
| MAPK3 |
| MAPK8 |
| MAPK8IP1 |
| MAPK8IP2 |
| MAPKAPK3 |
| MAPT |
| MBD3 |
| MBOAT1 |
| MBOAT2 |
| MBP |
| MC2R |
| MC3R |
| MC4R |
| MCHR1 |
| MCHR2 |
| MDM2 |
| MECP2 |
| MED12 |
| MED15 |
| MET |
| MGLL |
| MIS12 |
| MITF |
| MLLT4 |
| MMP2 |
| MMP3 |
| MMP9 |
| MPO |
| MSN |
| MST1R |
| MSX1 |
| MTHFD1 |
| MTNR1A |
| MTNR1B |
| MTOR |
| MTR |
| MTTP |
| MYB |
| MYC |
| MYO6 |
| MYO9B |
| NCAM1 |
| NCAN |
| NCL |
| NCOA2 |
| NDEL1 |
| NEDD4 |
| NEUROD1 |
| NFATC1 |
| NFATC2 |
| NFATC3 |
| NFKB1 |
| NFKBIA |
| NGF |
| NGFR |
| NMU |
| NNMT |
| NOS1 |
| NOS2 |
| NOS3 |
| NOTCH1 |
| NOTCH2 |
| NOTCH3 |
| NPAS2 |
| NPBWR1 |
| NPPA |
| NPSR1 |
| NPY |
| NPY1R |
| NPY2R |
| NQO1 |
| NR0B2 |
| NR1D1 |
| NR1H2 |
| NR2E1 |
| NR2F6 |
| NR3C1 |
| NR4A1 |
| NR4A2 |
| NR5A1 |
| NRAS |
| NRG1 |
| NRG2 |
| NRP1 |
| NT5C2 |
| NT5E |
| NTRK1 |
| NTRK2 |
| NTS |
| NUMB |
| OCLN |
| OLIG2 |
| OLR1 |
| OPRD1 |
| OPRM1 |
| OXT |
| OXTR |
| PADI4 |
| PAFAH1B1 |
| PAG1 |
| PAH |
| PAK2 |
| PAK3 |
| PARD3 |
| PARK2 |
| PARP1 |
| PBK |
| PBRM1 |
| PBX1 |
| PCK1 |
| PCNA |
| PCNT |
| PDE4A |
| PDGFB |
| PDGFRB |
| PDYN |
| PDZK1 |
| PEMT |
| PER1 |
| PER2 |
| PER3 |
| PIK3CA |
| PIK3CB |
| PIK3R1 |
| PIK3R2 |
| PINK1 |
| PIP4K2A |
| PIP5K1B |
| PLA2G1B |
| PLA2G2A |
| PLA2G4A |
| PLA2G4B |
| PLA2G4D |
| PLA2G6 |
| PLA2G7 |
| PLAT |
| PLCB1 |
| PLCB2 |
| PLCB3 |
| PLCB4 |
| PLCD3 |
| PLCG1 |
| PLCG2 |
| PLD1 |
| PLD2 |
| PLG |
| PLK1 |
| PLXNA2 |
| PLXNB1 |
| PMCH |
| PNOC |
| PNP |
| PNPLA8 |
| PNPO |
| POLR2A |
| POMC |
| PPA2 |
| PPARA |
| PPARD |
| PPARG |
| PPARGC1A |
| PPP2R1A |
| PPP2R2A |
| PPP3CA |
| PPP3CB |
| PRKAA1 |
| PRKAA2 |
| PRKAB1 |
| PRKACA |
| PRKACG |
| PRKAR2A |
| PRKAR2B |
| PRKCA |
| PRKCE |
| PRKCZ |
| PRKG1 |
| PRL |
| PRLR |
| PSEN1 |
| PSEN2 |
| PSMD9 |
| PTEN |
| PTGDS |
| PTGER4 |
| PTGS1 |
| PTGS2 |
| PTH |
| PTHLH |
| PTK2 |
| PTK2B |
| PTPN11 |
| PTPN5 |
| PTPN6 |
| PTPRA |
| PYY |
| RAB3A |
| RAC1 |
| RAC3 |
| RAF1 |
| RAN |
| RARA |
| RARB |
| RARG |
| RASGRF2 |
| RBBP4 |
| RDH8 |
| RELA |
| RELN |
| RENBP |
| RET |
| RGS2 |
| RGS4 |
| RHOA |
| RHOU |
| RIMS1 |
| RNF5 |
| ROCK1 |
| RORA |
| RORB |
| RUNX1 |
| RXFP3 |
| RXFP4 |
| RXRA |
| RXRG |
| S100B |
| SAT1 |
| SCGB1A1 |
| SCT |
| SDC1 |
| SDC2 |
| SDC3 |
| SDC4 |
| SDCBP |
| SEBOX |
| SELE |
| SEMA3A |
| SERPINA1 |
| SERPINE1 |
| SETD2 |
| SETDB1 |
| SF3A2 |
| SFN |
| SH3GL2 |
| SHC1 |
| SHH |
| SHMT1 |
| SHMT2 |
| SIN3A |
| SIRT1 |
| SLAMF1 |
| SLC18A2 |
| SLC26A6 |
| SLC26A9 |
| SLC3A2 |
| SLC6A3 |
| SMAD2 |
| SMAD4 |
| SMARCA2 |
| SMARCA4 |
| SMARCC1 |
| SMPD1 |
| SNCA |
| SOCS3 |
| SOD1 |
| SOD2 |
| SOS1 |
| SP1 |
| SPTBN1 |
| SRC |
| SRD5A1 |
| SREBF1 |
| SREBF2 |
| SST |
| SSTR2 |
| SSTR5 |
| STAT1 |
| STAT3 |
| STK11 |
| STX1A |
| SYK |
| SYN1 |
| SYNJ1 |
| SYT1 |
| TAC1 |
| TACR1 |
| TAL1 |
| TBP |
| TBXA2R |
| TBXAS1 |
| TCF7L2 |
| TCP1 |
| TDP2 |
| TF |
| TGFB1 |
| TGFBR1 |
| TGFBR2 |
| TGIF1 |
| TH |
| THBS1 |
| THRA |
| TIMELESS |
| TJP1 |
| TLR2 |
| TLR4 |
| TLR9 |
| TNF |
| TNFRSF1A |
| TOM1 |
| TP53 |
| TPH1 |
| TRAF1 |
| TRAF3 |
| TRH |
| TRIB3 |
| TSC1 |
| TSHR |
| TSPO |
| TTR |
| TYK2 |
| TYROBP |
| UBA52 |
| UBB |
| UBC |
| UBE2A |
| UCP2 |
| UGDH |
| UGT1A1 |
| UGT1A4 |
| UGT1A6 |
| UGT1A7 |
| UGT2B10 |
| UGT2B15 |
| UGT2B17 |
| UNC5C |
| UPP2 |
| VAMP2 |
| VAV1 |
| VAV3 |
| VCAM1 |
| VCAN |
| VCL |
| VCP |
| VDAC1 |
| VDR |
| VEGFA |
| VIP |
| VIPR1 |
| VIPR2 |
| VLDLR |
| VTN |
| VWF |
| WDR77 |
| WNT1 |
| WNT3A |
| XBP1 |
| XRCC1 |
| YES1 |
| YWHAB |
| YWHAE |
| YWHAG |
| YWHAH |
| YWHAQ |
| YWHAZ |

**Supplementary Table S4** The candidate key causal genes for ATDH and SCZ.

|  | GENE | FDR corrected P | P | Number of neighbors | Number of neighbors that are common disease | Neighbors that are common disease |
| --- | --- | --- | --- | --- | --- | --- |
| 1 | AKT1 | 1.26E-75 | 1.43E-79 | 237 | 134 | PTGS2, HMOX1, CREBBP, ESR1, RXRA, IL6, TP53, JUN, UBC, INS-IGF2, YWHAZ, FASLG, UBA52, MYC, EP300, BRCA1, FOS, STAT3, VEGFA, BCL2, CCND1, NOS3, CREB1, CSF2, EGF, CAV1, BIRC5, FGF2, MMP2, RELA, CDH1, HSP90AA1, SRC, HRAS, ARNT, CTNNB1, HIF1A, GSK3B, STAT1, IGF1, AR, TGFB1, NFKB1, ATIC, VIM, LEP, GRB2, RAC1, FOXO1, IL2, FYN, UBB, JAK1, NOTCH1, NFKBIA, IRS1, YWHAG, PTPN11, IL2RA, NGF, MDM2, SOS1, LCK, CASP3, PIK3CA, PTEN, IKBKG, YWHAB, SHC1, BDNF, DNMT1, RAF1, CDKN1B, CDC42, IKBKB, JAK3, PIK3R1, CTLA4, YWHAE, YWHAQ, GNAI2, PPP1CB, INSR, YWHAH, VAV1, RAC3, ICAM1, BAD, HSPA4, IRS2, BCL2L1, CALM1, PTPN1, EDN1, ERBB3, VLDLR, RELN, PPP2CA, MTOR, YES1, CD44, FN1, PIK3CB, PIK3CG, CXCR4, PRKCZ, YAP1, MBP, PIK3CD, IL3, EZR, GAPDH, EPOR, PIK3R2, MAPT, RASA1, CXCL12, PDGFRB, CD86, GRB10, EDNRA, ITGA6, ITGB4, PDGFB, GDNF, GNG2, ARHGDIA, GLI2, VAV2, YBX1, PEA15, EZH2, ITGA2B, FOXP2 |
| 2 | PIK3CA | 1.77E-75 | 4.00E-79 | 193 | 121 | ESR1, INS-IGF2, AKT1, YWHAZ, IL1B, ANXA1, STAT3, VEGFA, CCND1, NOS3, NRAS, KIT, EGFR, CSF2, EGF, ERBB2, KNG1, RELA, SRC, HRAS, IFNG, CTNNB1, IL23A, MAP2K1, SYK, IGF1, JAK2, GNA15, NFKB1, GRB2, RAC1, IL2, FYN, PRKCA, JAK1, NFKBIA, IRS1, PTPN11, IL2RA, NGF, SOS1, LCK, APP, PTEN, IL4, SHC1, CDC42, JAK3, PIK3R1, ITGAV, INSR, VAV1, RAC3, IRS2, BCL2L1, F2R, PTPN1, EDN1, MET, RELN, PRKCD, PLCG1, MTOR, YES1, CBL, PIK3CB, PRKCZ, CRK, CDH2, IL3, LPAR2, RAC2, BCAR1, PLCB2, GCG, PTPN6, PLCB3, IL1R1, PIK3R2, PDGFRB, CD86, GNRH1, CASR, EDNRA, ITGA6, GSN, INPP5D, GAST, PLCG2, PTK2, ITGB4, NGFR, CTNNA1, PDGFB, EDNRB, PLCB1, ERBB4, GDNF, EPB41L1, GNG2, TYROBP, CTNND1, TBXA2R, PLCB4, CCK, TRH, VAV2, RET, CSF2RA, GRP, EPHB2, PTAFR, PLCE1, P2RY2, TAC1, LTB4R, PLCD3, HCRT, AKT3, CHRM3, IRS4 |
| 3 | PIK3R1 | 1.74E-65 | 5.93E-69 | 163 | 104 | ESR1, INS-IGF2, AKT1, YWHAZ, IL1B, STAT3, CCND1, NRAS, KIT, CSF1R, EGFR, CSF2, EGF, ERBB2, KNG1, RELA, SRC, HRAS, IFNG, CTNNB1, MAP2K1, SYK, IGF1, JAK2, NFKB1, GRB2, RAC1, IL2, FYN, PRKCA, JAK1, NFKBIA, IRS1, PTPN11, IL2RA, NGF, SOS1, LCK, APP, PIK3CA, PTEN, IL4, SHC1, CDC42, JAK3, CTLA4, ITGAV, INSR, VAV1, IL12B, IRS2, BCL2L1, PTPN1, EDN1, MET, ERBB3, VLDLR, PLCG1, MTOR, FGFR2, CBL, PIK3CB, PIK3CG, PRKCZ, CRK, CDH2, PIK3CD, IL3, RAC2, BCAR1, GCG, PTPN6, EPOR, IL1R1, PIK3R2, ABL1, GCGR, PDGFRB, CD86, ITGA6, INPP5D, GAST, PLCG2, PTK2, CTNNA1, PDGFB, BDKRB2, ERBB4, GDNF, CSF2RB, CCK, TRH, VAV2, RET, CSF2RA, EPHB2, PLCE1, P2RY2, EFNB2, DNM1, TIAM1, NTRK1, HCRT, IRS4 |
| 4 | SRC | 1.02E-60 | 4.61E-64 | 260 | 126 | MAPK1, ESR1, RXRA, JUN, UBC, INS-IGF2, AKT1, CASP8, FASLG, MAPK14, FOS, STAT3, GPC1, VEGFA, CCND1, MAPK11, NOS3, NRAS, EGFR, EGF, ERBB2, CAV1, FGF2, MMP2, RELA, CDH1, HSP90AA1, HRAS, CARM1, CTNNB1, MAP2K1, SYK, STAT1, IGF1, JAK2, AR, NFKB1, VIM, GRB2, RAC1, ESR2, FYN, PRKCA, NFKBIA, IRS1, SDC2, PTPN11, NGF, SOS1, LCK, CASP3, PIK3CA, PTEN, CDKN1B, CDC42, PIK3R1, GNAI2, ITGAV, RAC3, CALM1, PTPN1, MET, MMP9, NR3C1, GJA1, PRKCD, SOCS3, PLCG1, YES1, CBL, CD44, FN1, PRL, PIK3CB, ADRB2, CALM2, PIK3CG, PRKCZ, CALM3, CRK, CDH2, PIK3CD, VCAM1, EZR, HCK, RAC2, BCAR1, ITGB1, PIK3R2, WWOX, ABL1, RASA1, GRIN2A, PDGFRB, GNB2L1, SDC3, GSN, PLCG2, PTK2, CTNNA1, PDGFB, GDNF, GNG2, HNRNPK, CTNND1, RAB5A, ARHGDIA, VCL, PTK2B, VAV2, JUP, RET, PTPN13, EPHB2, P2RY2, SIRPA, EFNB2, DNM1, CTTN, TIAM1, TJP1, DNM2, DCC, EPHB1, PTPRA, WAS |
| 5 | STAT3 | 1.52E-54 | 8.60E-58 | 149 | 91 | PTGS2, MAPK1, MAPK3, HMOX1, CREBBP, MAPK8, POMC, IL6, TP53, JUN, INS-IGF2, AKT1, MYC, EP300, MAPK14, FOS, VEGFA, CCND1, KIT, CSF1R, IL10, EGFR, SIRT1, CSF2, EGF, ERBB2, BIRC5, FGF2, RELA, SRC, IFNG, IL23A, MAP2K1, SP1, HIF1A, SYK, STAT1, IGF1, JAK2, AR, LEP, GRB2, RAC1, HDAC1, CEBPD, FOXO1, IL2, FYN, JAK1, NOTCH1, HDAC3, SDC2, PTPN11, IL2RA, LCK, IL8, PIK3CA, DNMT1, JAK3, PIK3R1, RAC3, ICAM1, IL12B, BCL2L1, PTPN1, MMP9, NR3C1, PRKCD, SOCS3, MTOR, YES1, CD44, PRL, CCR5, CXCR4, IL6R, HCK, IL6ST, CRP, CXCL12, PDGFRB, IL17A, PDGFB, CNTF, CSF2RB, A2M, RET, CSF2RA, PRLR, LEPR, LIFR |
| 6 | VEGFA | 1.06E-52 | 7.24E-56 | 131 | 84 | PTGS2, MAPK1, HMOX1, CREBBP, ESR1, SOD2, IL6, TP53, JUN, INS-IGF2, AKT1, IL1B, EP300, STAT3, GPC1, CCND1, NOS3, EGFR, PPARG, EGF, ERBB2, BIRC5, FGF2, MMP2, TGFBI, F3, CDH1, SRC, HRAS, ARNT, SP1, HIF1A, IGF1, TGFB1, LEP, RAC1, NOTCH1, PTPN11, NGF, IL8, PIK3CA, PTEN, BDNF, JAK3, ITGAV, ALB, RAC3, ICAM1, EDN1, MET, MMP9, GJA1, MTOR, CBL, CCL2, CD44, FN1, PIK3CB, PIK3CG, CXCR4, PIK3CD, VCAM1, SERPINE1, ADM, ITGB1, MMP3, EPAS1, CRP, ACE, CXCL12, PDGFRB, PLG, PTK2B, RET, IGHG1, SHC2, SIRPA, MMP14, EFNB2, THBS1, TJP1, PLAT, AKT3, NRP1 |
| 7 | HRAS | 3.48E-52 | 2.76E-55 | 139 | 86 | MAPK1, MAPK3, MAPK8, TP53, JUN, INS-IGF2, AKT1, YWHAZ, CAMK2B, FOS, VEGFA, CCND1, NRAS, KIT, EGFR, CSF2, EGF, ERBB2, HBEGF, SRC, MAP2K1, IGF1, JAK2, GRB2, RAC1, IL2, FYN, PRKCA, JAK1, IRS1, SDC2, YWHAG, PTPN11, IL2RA, PCNA, MDM2, SOS1, LCK, PIK3CA, YWHAB, SHC1, RAF1, JAK3, PIK3R1, YWHAE, YWHAQ, GNAI2, INSR, YWHAH, VAV1, IRS2, CALM1, MET, ERBB3, PRKCD, SOCS3, YES1, PIK3CB, PIK3CG, PIK3CD, RASA1, GRIN2A, ACTN2, PDGFRB, SPTAN1, ITGA6, GNB2L1, INPP5D, PTK2, ITGB4, PDGFB, GRIN2D, GDNF, RET, CSF2RA, EPHB2, PLCE1, SHC2, NCAM1, EFNB2, TIAM1, RASGRF1, NTF3, AKAP9, EPHB1, PTPRA |
| 8 | EGFR | 2.51E-51 | 2.27E-54 | 177 | 96 | PTGS2, MAPK1, MAPK3, ESR1, IL6, TP53, JUN, UBC, INS-IGF2, PLD2, YWHAZ, UBA52, STAT3, VEGFA, CCND1, NRAS, EGF, ERBB2, CAV1, BIRC5, FGF2, CDH1, HBEGF, HSP90AA1, SRC, HRAS, CTNNB1, HIF1A, STAT1, IGF1, JAK2, AR, GRB2, RAC1, FYN, UBB, PRKCA, NOTCH1, PTPN11, MDM2, SOS1, LCK, IL8, PIK3CA, PTEN, SHC1, CDC42, PIK3R1, CALM1, PTPN1, EDN1, MET, MMP9, ERBB3, PLCG1, MTOR, YES1, CBL, CD44, FN1, SH3GL2, PIK3CB, CALM2, PIK3CG, L1CAM, CALM3, CRK, PIK3CD, EZR, HCK, LPAR2, PTPN6, ITGB1, DCN, PIK3R2, MMP3, AP2M1, RASA1, GRB10, AP2A1, EDNRA, ITGA6, GSN, PTK2, ITGB4, CTNNA1, NRG1, CTNND1, RAB5A, VAV2, JUP, IGHG1, SLC9A3R1, SHC2, DNM1, SLC9A1 |
| 9 | FOS | 4.04E-51 | 4.12E-54 | 149 | 88 | PTGS2, MAPK1, MAPK3, CREBBP, MAPK8, ESR1, POMC, NCOA2, IL6, TP53, JUN, UBC, INS-IGF2, AKT1, IL1B, FASLG, MYC, EP300, MAPK14, STAT3, CCND1, MAPK11, CD4, NRAS, IL10, CREB1, CSF2, EGF, NFATC2, PTH, NFATC1, NFATC3, FGF2, MMP2, SRC, HRAS, IFNG, CTNNB1, SYK, STAT1, IGF1, JAK2, SMAD4, TGFB1, LEP, IL2, ESR2, NOTCH1, SDC2, IL2RA, NGF, IL8, PTEN, IL4, BDNF, CDKN1B, TH, IL12B, EDN1, TCF7L2, MAPK9, NR3C1, CREM, NPPA, CRH, SRF, SST, IL3, LPAR2, TBP, NPY, ACE, PENK, GNRH1, GAL, GAST, GDNF, CCK, TRH, GRP, NTS, AGRP, HLA-DRB1, TAC1, LMNA, HCRT, GHRL, CCKAR |
| 10 | MAPK1 | 4.31E-51 | 4.89E-54 | 167 | 93 | GSTA2, GSTA4, GSTA1, GSTA3, PLA2G1B, PTGS2, PLA2G4A, MAPK3, ESR1, RXRA, IL6, TP53, JUN, UBC, INS-IGF2, PLD2, CASP8, YWHAZ, IL1B, MYC, MAPK14, FOS, STAT3, CEBPB, VEGFA, BCL2, CCND1, EGFR, CREB1, CSF2, EGF, RELA, SRC, HRAS, IFNG, SMAD3, MAP2K1, SP1, STAT1, IGF1, JAK2, AR, SMAD4, TGFB1, NFKB1, SMAD2, GRB2, CDK2, RAC1, IL2, FYN, PRKCA, JAK1, NFKBIA, IRS1, SDC2, YWHAG, PTPN11, IL2RA, NGF, SOS1, LCK, APP, CASP3, IL8, YWHAB, SHC1, RAF1, JAK3, YWHAE, YWHAQ, GNAI2, ITGAV, INSR, YWHAH, VAV1, RAC3, MYD88, BAD, F2R, MET, SDC4, KSR1, GJA1, PPP2CA, TNF, YES1, SDC1, DUSP6, PTPN5, SH3GL2, L1CAM, MBP |
| 11 | IL6 | 4.55E-50 | 5.67E-53 | 119 | 78 | GPX1, PLA2G1B, PTGS2, MAPK1, HMOX1, ESR1, SOD2, POMC, TP53, JUN, INS-IGF2, AKT1, TNFRSF1A, IL1B, FOS, STAT3, VEGFA, CCND1, NOS3, IL10, EGFR, PPARG, CSF2, EGF, PTH, KNG1, FGF2, RELA, IL1A, F3, IFNG, IL23A, STAT1, IGF1, JAK2, AR, TGFB1, NFKB1, LEP, APOE, GRB2, FOXO1, IL2, JAK1, IRS1, PTPN11, IL2RA, SOS1, IL8, IL4, BDNF, VAV1, ICAM1, MYD88, IL12B, EDN1, MMP9, NR3C1, SOCS3, CCL2, CD44, CRH, FN1, PRL, PIK3CB, CXCR4, IL6R, VCAM1, HCK, SERPINE1, IL18, IL6ST, CRP, TLR2, GFAP, IL17A, CNTF, FTL |
| 12 | JUN | 5.40E-48 | 7.34E-51 | 170 | 91 | PTGS2, MAPK1, MAPK3, HMOX1, CREBBP, MAPK8, NFE2L2, ESR1, PPARA, IL6, TP53, UBC, INS-IGF2, AKT1, TRADD, IL1B, FASLG, MYC, EP300, MAPK14, FOS, STAT3, CEBPB, VEGFA, CCND1, MAPK11, NOS3, NRAS, IL10, EGFR, PPARG, CREB1, SIRT1, CSF2, EGF, NFATC2, NFATC1, NFATC3, FGF2, MMP2, RELA, SRC, HRAS, IFNG, CTNNB1, IL23A, SMAD3, SP1, HIF1A, SYK, GSK3B, RB1, IGF1, AR, SMAD4, TGFB1, SMAD2, IL2, NOTCH1, SDC2, IL2RA, NGF, IL8, PTEN, IL4, CDKN1B, TH, ICAM1, EDN1, TCF7L2, MMP9, MAPK9, NR3C1, CREM, CCL2, CD44, SRF, IL3, LPAR2, ATF4, MSH2, DCN, CSNK2A1, TBP, DKK1, B2M, ABL1, PENK, CD247, DVL3, NTS |
| 13 | JAK2 | 7.15E-47 | 1.05E-49 | 148 | 84 | MAPK1, MAPK3, IL6, UBC, INS-IGF2, TNFRSF1A, YWHAZ, IL1B, FASLG, CAMK2B, FOS, STAT3, CD4, NRAS, KIT, EGFR, CSF2, ERBB2, RELA, HBEGF, SRC, HRAS, IFNG, IL23A, STAT1, NFKB1, LEP, GRB2, RAC1, IL2, FYN, PRKCA, JAK1, NFKBIA, IRS1, PTPN11, IL2RA, SOS1, LCK, PIK3CA, IL4, SHC1, PIK3R1, INSR, VAV1, RAC3, IL12B, IRS2, PTPN1, EDN1, ERBB3, PRKCD, SOCS3, PLCG1, YES1, CBL, CCL2, PRL, PIK3CB, CCR5, PIK3CG, CXCR4, IL6R, SKP1, PIK3CD, IL3, BCAR1, IL6ST, PTPN6, EPOR, IL1R1, CXCL12, PDGFRB, EDNRA, INPP5D, PDGFB, CSF2RB, VAV2, CSF2RA, PRLR, SIRPA, LEPR, IL10RA, LIFR |
| 14 | PTPN11 | 1.61E-45 | 2.55E-48 | 138 | 80 | MAPK1, MAPK3, IL6, UBC, INS-IGF2, AKT1, UBA52, STAT3, VEGFA, CD4, NRAS, KIT, EGFR, CSF2, EGF, ERBB2, SRC, HRAS, IFNG, CTNNB1, MAP2K1, SYK, STAT1, IGF1, JAK2, GRB2, IL2, FYN, JAK1, IRS1, SDC2, IL2RA, SOS1, LCK, PIK3CA, SHC1, JAK3, PIK3R1, CTLA4, INSR, VAV1, IRS2, MET, ERBB3, SOCS3, YES1, CBL, PRL, PIK3CB, PIK3CG, CXCR4, PRKCZ, CRK, IL6R, CDH2, IL3, HCK, IL6ST, PTPN6, PIK3R2, CXCL12, PDGFRB, CD86, CD247, INPP5D, PTK2, PDGFB, GDNF, GNG2, CTNND1, CSF2RB, PTK2B, VAV2, RET, CSF2RA, PRLR, HLA-DRB1, SIRPA, LEPR, DCC |
| 15 | APP | 3.76E-44 | 6.39E-47 | 157 | 84 | MAPK1, MAPK3, POMC, UBC, CASP8, ANXA1, GPC1, EGF, KNG1, TGFBI, RELA, GNA15, TGFB1, NFKB1, APOE, NFKBIA, PIK3CA, IKBKG, SHC1, IKBKB, PIK3R1, APOA1, ALB, F2R, EDN1, MAPK9, ADCY2, NPPA, SNCA, FN1, PRL, MAPK8IP1, CCR5, SST, CALCA, ADCY8, SERPINE1, CXCR2, PLCB2, GCG, IAPP, GAPDH, HSPG2, TTR, NLRP3, PLCB3, NPY, B2M, PENK, GNRH1, EDNRA, GAL, CASP6, GSN, PLG, GAST, ADCY1, NGFR, OPRM1, EDNRB, ADCY5, PLCB1, DRD2, HTR1A, PLCB4, CCK, A2M, TRH, PDYN, PSEN1, DRD3, GRP, NTS, ADRBK1, OPRD1, P2RY2, CLU, TAC1, HCRT, SERPING1, TACR1, CHRM3, S100B, OXTR |
| 16 | EGF | 1.25E-43 | 2.27E-46 | 144 | 80 | PTGS2, MAPK1, MAPK3, IL6, JUN, UBC, AKT1, UBA52, FOS, STAT3, VEGFA, CCND1, NRAS, EGFR, ERBB2, CAV1, KNG1, FGF2, TGFBI, CDH1, HBEGF, SRC, HRAS, CTNNB1, STAT1, IGF1, TGFB1, VIM, GRB2, RAC1, FYN, UBB, PTPN11, PCNA, SOS1, LCK, APP, PIK3CA, SHC1, CDC42, PIK3R1, ALB, PTPN1, EDN1, MET, MMP9, ERBB3, PLCG1, MTOR, YES1, CBL, CD44, FN1, PRL, SH3GL2, HCK, SERPINE1, PTPN6, ITGB1, AP2M1, RASA1, AP2A1, EDNRA, ITGA6, GSN, PLG, GAST, ITGB4, CTNNA1, ERBB4, NRG1, CTNND1, CNTF, RAB5A, ALDOA, A2M, CLU, DNM1, SERPING1, SLC9A1 |
| 17 | GRB2 | 1.99E-41 | 3.83E-44 | 179 | 87 | MAPK1, MAPK3, ESR1, IL6, INS-IGF2, AKT1, FASLG, STAT3, NRAS, KIT, CSF1R, EGFR, CREB1, CSF2, EGF, ERBB2, SRC, HRAS, MAP2K1, SYK, IGF1, JAK2, IL2, FYN, JAK1, IRS1, SDC2, PTPN11, IL2RA, SOS1, LCK, PIK3CA, IL4, SHC1, CDKN1B, JAK3, PIK3R1, INSR, VAV1, IRS2, PTPN1, MET, ERBB3, MAPK9, PRKCD, PLCG1, MTOR, FGFR2, YES1, CBL, PRKCZ, CRK, IL6R, IL3, HCK, HNRNPC, BCAR1, IL6ST, PTPN6, EPOR, PIK3R2, ABL1, RASA1, PDGFRB, ITGA6, GNB2L1, INPP5D, PTK2, ITGB4, PDGFB, GDNF, PTK2B, A2M, VAV2, RET, CSF2RA, VAV3, SHC2, EFNB2, DNM1, CTTN, NTRK1, DNM2, EPHB1, PTPRA, WAS, IRS4 |
| 18 | PRKCA | 5.21E-39 | 1.06E-41 | 75 | 55 | MAPK1, MAPK3, RXRA, PLD2, YWHAZ, CHEK1, KIT, EGFR, RELA, HBEGF, SRC, HRAS, JAK2, NFKB1, RAC1, FYN, NFKBIA, YWHAG, LCK, PIK3CA, IKBKG, YWHAB, RAF1, IKBKB, PIK3R1, YWHAQ, ICAM1, SDC4, GJA1, PRKCD, PLCG1, MTOR, YES1, RHOA, CRK, MBP, VCAM1, EZR, BCAR1, ITGB1, PIK3R2, MMP3, GRIN2A, GRB10, ITGA6, PLCG2, ITGB4, PLCB1, GDNF, ARHGDIA, CABIN1, PTK2B, RET, ADRBK1, PRKACA |
| 19 | INS-IGF2 | 6.55E-39 | 1.41E-41 | 194 | 88 | CYP3A4, MAPK1, PLA2G7, MAPK8, ESR1, SOD2, POMC, IL6, GAD2, JUN, AKT1, UBA52, MYC, FOS, STAT3, VEGFA, CCND1, NOS3, EGFR, PPARG, CREB1, CAV1, F3, SRC, HRAS, HIF1A, IGF1, JAK2, TGFB1, LEP, GRB2, CDK2, FOXO1, IRS1, PTPN11, NGF, SOS1, PIK3CA, PTEN, SHC1, PIK3R1, INSR, ALB, SREBF1, ICAM1, IRS2, CALM1, LPL, SREBF2, PTPN1, EDN1, TCF7L2, MMP9, SOCS3, MTOR, CBL, CCL2, CD44, PRL, CALM2, PRKCZ, CALM3, CDH2, VCAM1, SERPINE1, PKM, GCG, IAPP, GAPDH, TTR, NPY, GRB10, GNRH1, ALAS1, GAL, INPP5D, GFAP, GAST, SLC2A1, PPP1CC, VAMP2, UCP2, LEPR, EXOC2, INSIG2, PTPRA, MYO5A, IRS4 |
| 20 | LCK | 6.66E-39 | 1.51E-41 | 153 | 78 | MAPK1, MAPK3, UBC, AKT1, MYC, MAPK14, STAT3, MAPK11, CD4, NRAS, KIT, EGFR, EGF, RELA, SRC, HRAS, MAP2K1, SYK, STAT1, JAK2, NFKB1, GRB2, RAC1, IL2, FYN, PRKCA, JAK1, NOTCH1, NFKBIA, IRS1, PTPN11, IL2RA, SOS1, PIK3CA, SHC1, CDC42, JAK3, PIK3R1, CTLA4, VAV1, IL12B, IRS2, BCL2L1, MAPK9, PRKCD, SOCS3, PLCG1, YES1, SNCA, CBL, CD44, PIK3CB, CXCR4, PRKCZ, EZR, HCK, BCAR1, PTPN6, PIK3R2, B2M, ABL1, RASA1, CXCL12, PDGFRB, CD86, CD247, INPP5D, PLCG2, PDGFB, TYROBP, PTK2B, VAV2, PTPN13, VAV3, EPHB2, HLA-DRB1, EPHB1, PTPRC |
| 21 | RELA | 1.22E-38 | 3.15E-41 | 117 | 68 | MAPK1, MAPK3, CREBBP, ESR1, RXRA, IL6, JUN, UBC, AKT1, TNFRSF1A, CASP8, TRADD, RIPK1, TRAF1, IL1B, UBA52, MYC, EP300, CHEK1, MAPK14, STAT3, CEBPB, SIRT1, CSF2, SRC, CARM1, IL23A, SP1, SYK, JAK2, NFKB1, MAP3K7, HDAC1, CEBPD, IL2, UBB, PRKCA, NFKBIA, HDAC3, NGF, LCK, APP, IL8, PIK3CA, IKBKG, IKBKB, PIK3R1, ICAM1, IL12B, KAT2B, MMP9, NR3C1, PPP2CA, TNF, PRKCD, MTOR, PRKCZ, EGR2, LPAR2, IL18, HDAC2, CSNK2A1, IL1R1, TBP, BTRC, IL17A, PPP2R1A, NGFR |
| 22 | MAPK3 | 1.22E-38 | 3.15E-41 | 117 | 68 | PLA2G1B, PTGS2, MAPK1, RXRA, JUN, PLD2, CASP8, YWHAZ, MYC, MAPK14, FOS, STAT3, CEBPB, BCL2, EGFR, CREB1, CSF2, EGF, NFATC1, RELA, HRAS, IFNG, MAP2K1, SP1, STAT1, IGF1, JAK2, TGFB1, NFKB1, SMAD2, GRB2, CDK2, IL2, PRKCA, JAK1, NFKBIA, IRS1, SDC2, YWHAG, PTPN11, IL2RA, NGF, SOS1, LCK, APP, YWHAB, SHC1, RAF1, JAK3, YWHAE, YWHAQ, GNAI2, ITGAV, INSR, YWHAH, BAD, IRS2, F2R, MET, SDC4, KSR1, GJA1, PPP2CA, PRKCD, PLCG1, DUSP6, PTPN5, IFIT1 |
| 23 | FYN | 1.22E-38 | 3.19E-41 | 187 | 86 | MAPK1, UBC, PLD2, AKT1, FASLG, MAP2K7, MAPK14, STAT3, MAPK11, CD4, NRAS, KIT, EGFR, EGF, CAV1, CDH1, SRC, HRAS, CTNNB1, SYK, STAT1, JAK2, GRB2, RAC1, IL2, PRKCA, JAK1, IRS1, PTPN11, SOS1, LCK, PIK3CA, SHC1, CDC42, JAK3, PIK3R1, CTLA4, GNAI2, ITGAV, VAV1, VLDLR, RELN, PRKCD, PLCG1, YES1, SNCA, CBL, CD44, PIK3CB, PIK3CG, CRK, HCK, BCAR1, PTPN6, PIK3R2, MAPT, ABL1, RASA1, GRIN2A, PDGFRB, CD86, CD247, GNB2L1, SDC3, INPP5D, PLCG2, PTK2, DPYSL2, PDGFB, TYROBP, CTNND1, VCL, PTK2B, VAV2, JUP, PTPN13, VAV3, EPHB2, CDK5, NCAM1, CTTN, DCC, EPHB1, PTPRA, PTPRC, WAS |
| 24 | IRS1 | 1.41E-37 | 3.83E-40 | 81 | 56 | MAPK1, MAPK3, MAPK8, ESR1, IL6, UBC, INS-IGF2, AKT1, NRAS, CAV1, SRC, HRAS, IGF1, JAK2, LEP, GRB2, IL2, FYN, JAK1, PTPN11, IL2RA, NGF, SOS1, LCK, PIK3CA, IL4, SHC1, IKBKB, JAK3, PIK3R1, INSR, IRS2, PTPN1, MAPK9, PRKCD, SOCS3, MTOR, CBL, FN1, PRL, PIK3CB, PIK3CG, PRKCZ, YAP1, CRK, PIK3CD, BCAR1, PIK3R2, GNB2L1, INPP5D, PTK2, GDNF, RET, PRLR, LEPR, NTRK1 |
| 25 | KNG1 | 3.37E-37 | 9.56E-40 | 198 | 87 | PTGS2, POMC, IL6, ANXA1, NOS3, EGF, GNA15, TGFB1, SDC2, APP, PIK3CA, PIK3R1, ALB, F2R, EDN1, ADCY2, NPPA, FN1, CCR5, SST, ADCY8, LPAR2, SERPINE1, CXCR2, PLCB2, GCG, ADORA1, ADCY6, SSTR5, PLCB3, NPY, ACE, PENK, GCGR, GNRH1, CASR, EDNRA, GAL, PLG, GAST, OPRM1, CHRM2, EDNRB, BDKRB2, ITGB2, PLCB1, DRD2, HTR1A, NMU, TBXA2R, PTGER3, PLCB4, CCK, ALDOA, ADRA2A, A2M, RGS4, TRH, PDYN, RGS2, DRD3, NPY1R, DRD4, PNOC, GRP, NPY2R, NTS, ADRBK1, HRH3, PTAFR, CHRM4, SSTR2, NMS, P2RY2, GPR55, CLU, TAC1, GNRHR, LTB4R, HCAR2, HCRT, SERPING1, GRK5, TACR1, CCKAR, CHRM3, OXTR |
| 26 | SHC1 | 3.75E-37 | 1.11E-39 | 82 | 56 | MAPK1, MAPK3, INS-IGF2, AKT1, YWHAZ, MAPK14, CD4, NRAS, EGFR, CREB1, EGF, ERBB2, HRAS, MAP2K1, SYK, IGF1, JAK2, GRB2, IL2, FYN, JAK1, IRS1, PTPN11, IL2RA, NGF, SOS1, LCK, APP, PIK3CA, JAK3, PIK3R1, INSR, PTPN1, MET, ERBB3, MAPK9, PRKCD, CBL, IL3, EPOR, ABL1, RASA1, PDGFRB, GRB10, CD247, TGFBR2, INPP5D, PTK2, ITGB4, NGFR, GDNF, CSF2RB, RET, SHC2, NTRK1, NTRK2 |
| 27 | IGF1 | 2.24E-36 | 6.85E-39 | 90 | 58 | MAPK1, MAPK3, PLA2G7, ESR1, IL6, TP53, JUN, INS-IGF2, AKT1, IL1B, FOS, STAT3, VEGFA, CCND1, EGFR, EGF, ERBB2, PTH, FGF2, MMP2, TGFBI, SRC, HRAS, CTNNB1, TGFB1, LEP, GRB2, FOXO1, IRS1, PTPN11, SOS1, PIK3CA, SHC1, BDNF, PIK3R1, ITGAV, INSR, IRS2, PTPN1, GJA1, PRKCD, SOCS3, CD44, FN1, PRL, PIK3CB, PIK3CG, CRK, PIK3CD, BCAR1, NPY, GRB10, GNRH1, GNB2L1, PLG, CTNND1, LEPR, GHRL |
| 28 | NFKB1 | 4.45E-36 | 1.41E-38 | 94 | 59 | MAPK1, MAPK3, CREBBP, RXRA, IL6, UBC, AKT1, TNFRSF1A, CASP8, TRADD, RIPK1, TRAF1, IL1B, UBA52, MYC, EP300, MAPK14, CEBPB, CSF2, RELA, SRC, IL23A, MAP2K1, SP1, SYK, JAK2, HDAC1, CEBPD, UBB, PRKCA, NOTCH1, NFKBIA, HDAC3, NGF, LCK, APP, IL8, PIK3CA, IKBKG, IKBKB, PIK3R1, RAC3, ICAM1, IL12B, MMP9, NR3C1, PRKCD, MTOR, PRKCZ, EGR2, SKP1, IL18, EPOR, IL1R1, KLF5, BTRC, IL17A, RAN, ITGB2 |
| 29 | NRAS | 5.28E-36 | 1.74E-38 | 70 | 51 | JUN, YWHAZ, FOS, KIT, EGFR, CSF2, EGF, ERBB2, HBEGF, SRC, HRAS, MAP2K1, JAK2, GRB2, RAC1, IL2, FYN, JAK1, IRS1, YWHAG, PTPN11, IL2RA, SOS1, LCK, PIK3CA, YWHAB, SHC1, RAF1, JAK3, PIK3R1, YWHAE, YWHAQ, YWHAH, IRS2, ERBB3, SOCS3, YES1, PIK3CG, RASA1, PDGFRB, SPTAN1, PTK2, PDGFB, CSF2RA, EPHB2, SHC2, NCAM1, EFNB2, TIAM1, RASGRF1, NTF3 |
| 30 | POMC | 5.76E-36 | 1.96E-38 | 157 | 76 | CYP1B1, IL6, INS-IGF2, CYP17A1, FOS, ANXA1, STAT3, PTH, KNG1, LEP, APP, GNAI2, ADCY2, CRH, ADRB2, CCR5, SST, LHCGR, CALCA, ADCY9, ADCY8, CXCR2, GCG, ADORA1, ADM, IAPP, ADCY6, SSTR5, NPY, ADORA2A, GNAS, PENK, GCGR, DRD5, ADRB1, ADCY7, CASR, GAL, SDC3, AVPR2, ADCY1, HTR7, ADCY4, OPRM1, CHRM2, BDKRB2, ADCY5, MC4R, DRD2, HTR1A, NMU, MCHR1, VIPR2, HTR4, PTGER3, MTNR1A, ADRA2A, RGS4, PDYN, DRD3, NPY1R, ADRB3, DRD4, PNOC, NPY2R, HRH3, AGRP, CHRM4, SSTR2, NMS, OPRD1, GPR55, ADRA2C, HTR1B, NMUR2, HTR6 |
| 31 | EDN1 | 9.73E-36 | 3.42E-38 | 95 | 59 | CREBBP, IL6, JUN, INS-IGF2, AKT1, MAPK14, FOS, ANXA1, VEGFA, NOS3, EGFR, EGF, KNG1, ARNT, HIF1A, JAK2, GNA15, RAC1, NOS2, APP, PIK3CA, PIK3R1, GNAI2, ALB, F2R, GJA1, ADCY2, NPPA, FN1, LPAR2, GCG, ADM, PLCB3, PIK3R2, GNRH1, CASR, EDNRA, GAST, ADCY4, EDNRB, BDKRB2, ADCY5, PLCB1, TBXA2R, PLCB4, CCK, TRH, RGS2, GRP, NTS, ADRBK1, PTAFR, P2RY2, TAC1, GRK5, TACR1, CHRM3, SLC9A1, OXTR |
| 32 | IL2 | 3.87E-35 | 1.40E-37 | 100 | 60 | MAPK1, MAPK3, IL6, JUN, AKT1, IL1B, FASLG, MYC, MAPK14, FOS, STAT3, MAPK11, NRAS, IL10, PPARG, CSF2, NFATC2, NFATC1, NFATC3, RELA, HRAS, IFNG, MAP2K1, SYK, STAT1, JAK2, TGFB1, GRB2, FYN, JAK1, IRS1, PTPN11, IL2RA, SOS1, LCK, IL8, PIK3CA, IL4, SHC1, JAK3, PIK3R1, ICAM1, IRS2, BCL2L1, MAPK9, CREM, SOCS3, MTOR, PRL, CCR5, PRKCZ, EGR2, IL3, IL6ST, GAPDH, B2M, CD247, EGR3, PTK2B, PTPRC |
| 33 | RAC1 | 8.83E-35 | 3.30E-37 | 234 | 92 | MAPK1, UBC, AKT1, MAP2K7, PAFAH1B1, MAPK14, STAT3, GPC1, VEGFA, CCND1, MAPK11, NRAS, KIT, EGFR, EGF, ERBB2, CAV1, CDH1, APC, SRC, HRAS, CTNNB1, SYK, JAK2, NOS2, FYN, PRKCA, NGF, SOS1, LCK, IL8, PIK3CA, RAF1, CDC42, PIK3R1, VAV1, ICAM1, EDN1, MET, SDC4, MMP9, MAPK9, PLCG1, YES1, FN1, PIK3CB, PIK3CG, CXCR4, CRK, CDH2, PIK3CD, VCAM1, RAC2, BCAR1, PLCB2, ITGB1, PIK3R2, ABL1, CXCL12, PDGFRB, CD86, EDNRA, PTK2, NGFR, DVL2, CTNNA1, PDGFB, GNG2, TYROBP, CTNND1, ARHGDIA, DVL3, VAMP2, TBXA2R, DVL1, VAV2, JUP, WNT5A, VAV3, EPHB2, EFNB2, CTTN, TIAM1, DNM2, RASGRF1, EXOC2, DCC, EPHB1, SLIT2, MYO5A, RHOB, CYBB |
| 34 | PTGS2 | 1.20E-34 | 4.63E-37 | 85 | 55 | CYP2E1, CYP2B6, CYP2C19, CYP2C9, CYP2C18, CYP2C8, CYP1B1, PLA2G1B, PLA2G2A, PLA2G4A, PLA2G6, MAPK1, ALOX12, MAPK3, PTGDS, HPGDS, ESR1, PPARA, IL6, TP53, JUN, AKT1, PTGES2, PTGES, PTGIS, TBXAS1, IL1B, MAPK14, FOS, STAT3, VEGFA, MAPK11, NOS3, IL10, EGFR, PPARG, CREB1, CSF2, EGF, ERBB2, NFATC2, PTH, CAV1, NFATC1, NFATC3, KNG1, BIRC5, FGF2, MMP2, TGFBI, IL1A, F3, CDH1, HBEGF, APC |
| 35 | NGF | 1.07E-33 | 4.24E-36 | 94 | 57 | MAPK1, MAPK3, MAPK8, JUN, UBC, INS-IGF2, AKT1, IL1B, UBA52, FOS, VEGFA, E2F1, CREB1, FGF2, RELA, SRC, TGFB1, NFKB1, RAC1, UBB, NFKBIA, IRS1, SOS1, CASP3, PIK3CA, IKBKG, YWHAB, SHC1, IKBKB, TH, PIK3R1, YWHAE, MYD88, BAD, IRS2, MAPK9, PLCG1, SH3GL2, PIK3CB, PRKCZ, PIK3R2, NPY, CASP6, PLG, GFAP, NGFR, EPB41L1, ARHGDIA, A2M, SHC2, TAC1, DNM1, NTRK1, RASGRF1, FURIN, RHOB, NTRK2 |
| 36 | CSF2 | 2.02E-33 | 8.26E-36 | 56 | 44 | PTGS2, MAPK1, MAPK3, CREBBP, IL6, JUN, AKT1, IL1B, EP300, FOS, STAT3, NRAS, CSF1R, IL10, NFATC2, NFATC1, NFATC3, RELA, IL1A, HRAS, SYK, STAT1, JAK2, TGFB1, NFKB1, GRB2, IL2, SDC2, PTPN11, IL2RA, SOS1, PIK3CA, IL4, IKBKB, PIK3R1, ICAM1, CCL2, CCR5, IL3, ITGB1, TLR2, INPP5D, CSF2RB, CSF2RA |
| 37 | TGFB1 | 3.17E-33 | 1.33E-35 | 132 | 67 | MAPK1, MAPK3, CREBBP, RXRA, NCOA2, IL6, TP53, JUN, UBC, INS-IGF2, AKT1, UBA52, EP300, FOS, CEBPB, VEGFA, CCND1, IL10, CSF2, EGF, CAV1, KNG1, FGF2, MMP2, TGFBI, IL1A, CDH1, IFNG, SMAD3, IGF1, SMAD4, LEP, SMAD2, CEBPD, IL2, NOS2, UBB, HDAC3, SDC2, NGF, APP, PPARGC1A, MED12, PPP1CB, ALB, ICAM1, MMP9, SDC1, FN1, PRKCZ, EGR2, SERPINE1, DCN, MED15, ACTN2, KLF5, TGFBR2, PLG, IL17A, PDGFB, PPP1CC, ALDOA, A2M, CLU, THBS1, FURIN, SERPING1 |
| 38 | SOS1 | 1.13E-32 | 4.85E-35 | 104 | 59 | MAPK1, MAPK3, ESR1, IL6, INS-IGF2, AKT1, NRAS, KIT, EGFR, CREB1, CSF2, EGF, ERBB2, HBEGF, SRC, HRAS, MAP2K1, SYK, IGF1, JAK2, GRB2, RAC1, IL2, FYN, JAK1, IRS1, PTPN11, NGF, LCK, PIK3CA, SHC1, JAK3, PIK3R1, INSR, VAV1, IRS2, MET, ERBB3, PRKCD, PLCG1, MTOR, CBL, FN1, CRK, PIK3CD, HCK, RAC2, EPOR, PIK3R2, ABL1, PDGFRB, SPTAN1, PLCG2, PTK2, PDGFB, RET, SHC2, NCAM1, RHOB |
| 39 | CREBBP | 1.41E-32 | 6.22E-35 | 190 | 80 | CYP1A1, NFE2L2, ESR1, RXRA, PPARA, NCOA2, TP53, JUN, AKT1, MYC, EP300, BRCA1, MAPK14, FOS, STAT3, CEBPB, VEGFA, BCL2, CCND1, SIN3A, KIT, CREB1, CSF2, NFATC2, RELA, ARNT, SMARCA4, CARM1, CTNNB1, ABCA1, SMAD3, SP1, HIF1A, RB1, STAT1, AR, SMAD4, TGFB1, NFKB1, LEP, SMAD2, HDAC1, CEBPD, FOXO1, NOTCH1, HDAC3, IL2RA, MDM2, RORA, PPARGC1A, APOA1, SREBF1, MAX, LPL, SREBF2, EDN1, TCF7L2, KAT2B, NR3C1, CLOCK, NPAS2, NCL, CTBP1, YAP1, EGR2, SKP1, CDH2, HDAC2, ATF4, EPOR, ARNTL, EPAS1, MED15, YY1, SLC2A1, CITED2, CABIN1, HIST1H3A, HMGA1, KAT6A |
| 40 | CASR | 2.75E-32 | 1.25E-34 | 88 | 54 | POMC, ANXA1, PTH, KNG1, GNA15, PIK3CA, F2R, EDN1, ADCY2, CCR5, SST, GCG, ADORA1, ADCY6, SSTR5, NPY, GCGR, GNRH1, EDNRA, GAL, GAST, OPRM1, CHRM2, EDNRB, BDKRB2, ADCY5, DRD2, MCHR1, TBXA2R, CCK, RGS4, TRH, PDYN, DRD3, PNOC, GRP, NTS, ADRBK1, HRH3, PTAFR, SSTR2, NMS, OPRD1, P2RY2, TAC1, GNRHR, LTB4R, HCAR2, HCRT, GRK5, TACR1, CCKAR, CHRM3, OXTR |
| 41 | BDKRB2 | 5.73E-32 | 2.66E-34 | 76 | 50 | POMC, TP53, KNG1, GNA15, PIK3R1, GNAI2, F2R, EDN1, ADCY2, CCR5, SST, CXCR2, ADORA1, ADCY6, PLCB3, PIK3R2, NPY, CASR, EDNRA, GAL, GAST, OPRM1, CHRM2, EDNRB, PLCB1, DRD2, TBXA2R, PTGER3, CCK, MTNR1A, ADRA2A, RGS4, TRH, RGS2, DRD3, PNOC, GRP, NTS, ADRBK1, PTAFR, CHRM4, P2RY2, TAC1, GNRHR, HCAR2, GRK5, TACR1, CCKAR, CHRM3, OXTR |
| 42 | IGF1R | 5.55E-31 | 2.64E-33 | 55 | 42 | MAPK1, MAPK3, ESR1, TP53, UBC, INS-IGF2, VEGFA, EGFR, EGF, FGF2, SRC, HRAS, CTNNB1, IGF1, GRB2, IRS1, PTPN11, MDM2, SOS1, PIK3CA, YWHAB, SHC1, PIK3R1, GNAI2, ITGAV, IRS2, PTPN1, PRKCD, MTOR, CBL, FN1, PIK3CB, PIK3CG, CRK, PIK3CD, BCAR1, PIK3R2, GRB10, GNB2L1, PTK2, CTNNA1, CTNND1 |
| 43 | CTNNB1 | 5.95E-31 | 2.90E-33 | 194 | 79 | CREBBP, NCOA2, JUN, UBC, AKT1, YWHAZ, MYC, EP300, MAPK14, FOS, CCND1, LEF1, EGFR, EGF, ERBB2, MMP2, CDH1, APC, SRC, SMARCA4, SMAD3, GSK3B, IGF1, AR, RAC1, HDAC1, FOXO1, FYN, NOTCH1, YWHAG, PTPN11, IL8, PIK3CA, YWHAB, BDNF, CDKN1B, CDC42, PIK3R1, YWHAE, YWHAQ, MED12, YWHAH, PTPN1, MET, TCF7L2, MMP9, MAPK9, MTOR, CTBP1, PRKCZ, SKP1, CDH2, PTPN6, CSNK2A1, LRP6, DKK1, MMP3, ABL1, TGFBR2, BTRC, DVL2, CTNNA1, GDNF, CTNND1, RAB5A, DVL3, CSNK1D, VCL, DVL1, JUP, RET, PSEN1, CSNK1E, TNIK, CTTN, DKK4, TJP1, DNM2, NTRK2 |
| 44 | PRKCD | 2.64E-30 | 1.32E-32 | 46 | 38 | MAPK3, TP53, PLD2, STAT3, RELA, SRC, HRAS, IFNG, MAP2K1, STAT1, IGF1, JAK2, NFKB1, GRB2, FYN, PRKCA, NFKBIA, IRS1, SOS1, LCK, CASP3, PIK3CA, SHC1, RAF1, ICAM1, SDC4, PLCG1, YES1, SNCA, CRK, VCAM1, HCK, GNB2L1, ACTB, PLCG2, PLCB1, PTK2B, ADRBK1 |
| 45 | GCG | 9.25E-30 | 4.72E-32 | 138 | 65 | POMC, INS-IGF2, PTH, KNG1, GNA15, LEP, APP, PIK3CA, PIK3R1, F2R, EDN1, TCF7L2, ADCY2, CRH, ADRB2, SST, LHCGR, CALCA, ADCY9, ADCY8, PLCB2, ADM, IAPP, ADCY6, PIK3R2, ADORA2A, GNAS, GCGR, ADRB1, ADCY7, GNRH1, CASR, EDNRA, GAST, AVPR2, ADCY1, ADCY4, EDNRB, ADCY5, PLCB1, MC4R, NMU, MCHR1, TBXA2R, VIPR2, HTR4, CCK, TRH, RGS2, ADRB3, GRP, NTS, ADRBK1, PTAFR, P2RY2, TAC1, GNRHR, LTB4R, HCRT, GRK5, TACR1, GHRL, CCKAR, CHRM3, OXTR |
| 46 | UBC | 1.20E-29 | 6.27E-32 | 513 | 134 | MAPK1, NFE2L2, ESR1, TP53, JUN, AKT1, RIPK1, TRAF2, STUB1, UBA52, MYC, BRCA1, CHEK1, FOS, BCL2, CCND1, E2F1, CD4, HSPA8, KIT, TRIM28, EGFR, EGF, ERBB2, RELA, CDH1, HSP90AA1, SRC, SOD1, CTNNB1, ABCA1, SMAD3, SP1, HIF1A, SYK, RB1, STAT1, JAK2, AR, SMAD4, TGFB1, NFKB1, SMAD2, MAP3K7, CDK2, RAC1, HDAC1, FOXO1, NOS2, FYN, UBB, NOTCH1, NFKBIA, IRS1, PTPN11, NGF, PCNA, MDM2, LCK, APP, PTEN, IKBKG, CDKN1B, CDC42, IKBKB, BIRC2, PPARGC1A, YWHAE, VAV1, SREBF1, HSPA4, MET, VCP, ERBB3, NR3C1, GJA1, PPP2CA, INSIG1, SNCA, CBL, SH3GL2, MAVS, RHOA, ADRB2, CFTR, CXCR4, L1CAM, PRKCZ, PLK1, TRAF3, SKP1, HSPD1, PKM, ATF4, GGA3, GAPDH, XIAP, EPOR, MAPT, EPAS1, PARK2, GNAS, PRDX1, KLF5, PDGFRB, CD86, TGFBR2, ACTB, BTRC, INPP5D, AURKB, UBE2N, PPP2R1A, NGFR, RAN, NEDD8, ERBB4, HNRNPK, ALDOA, TAL1, YBX1, HDAC6, PHB, NOS1, HSPA5, CLU, TKT, CKB, NTRK1, DDX58, PER2, CRY2, UCHL1, NTRK2 |
| 47 | SYK | 1.31E-29 | 6.97E-32 | 107 | 57 | PLA2G4A, JUN, UBC, PLD2, TNFRSF1A, FASLG, MAPK14, FOS, STAT3, MAPK11, CD4, CSF2, RELA, SRC, NFKB1, GRB2, RAC1, IL2, FYN, JAK1, NFKBIA, PTPN11, IL2RA, SOS1, LCK, PIK3CA, SHC1, JAK3, PIK3R1, ITGAV, VAV1, PLCG1, YES1, SNCA, CBL, FN1, PIK3CB, PIK3CG, PIK3CD, HCK, RAC2, BCAR1, PTPN6, PIK3R2, CD247, PLCG2, ITGB2, TYROBP, CSF2RB, TBXA2R, PTK2B, VAV2, CSF2RA, VAV3, CTTN, ITGA2B, RHOB |
| 48 | GCGR | 1.87E-29 | 1.02E-31 | 86 | 51 | POMC, ANXA1, PTH, KNG1, GNA15, PIK3R1, F2R, ADCY2, CRH, ADRB2, LHCGR, CALCA, ADCY9, ADCY8, GCG, ADM, IAPP, ADCY6, PIK3R2, GNAS, DRD5, ADRB1, ADCY7, GNRH1, CASR, EDNRA, GAST, AVPR2, ADCY1, ADCY4, EDNRB, ADCY5, MC4R, NMU, TBXA2R, VIPR2, CCK, TRH, ADRB3, GRP, NTS, ADRBK1, PTAFR, TAC1, GNRHR, HCRT, GRK5, TACR1, CCKAR, CHRM3, OXTR |
| 49 | ADCY2 | 2.10E-29 | 1.17E-31 | 198 | 78 | POMC, ANXA1, PTH, KNG1, APP, GNAI2, PDE4A, CALM1, EDN1, CRH, ADRB2, CCR5, CALM2, SST, CALM3, LHCGR, CALCA, ADCY9, ADCY8, LPAR2, CXCR2, PKM, GCG, ADORA1, ADM, IAPP, ADCY6, SSTR5, NPY, ADORA2A, PKLR, GNAS, PENK, GCGR, DRD5, ADRB1, ADCY7, CASR, EDNRA, GAL, AVPR2, ADCY1, HTR7, ADCY4, OPRM1, CHRM2, BDKRB2, ADCY5, MC4R, GNG2, DRD2, HTR1A, NMU, MCHR1, VIPR2, HTR4, PTGER3, MTNR1A, ADRA2A, PDYN, DRD3, NPY1R, ADRB3, DRD4, PNOC, NPY2R, HRH3, CHRM4, SSTR2, OPRD1, GPR55, ADRA2C, HTR1B, PRKACA, NMUR2, HTR6, PDE8A, HCAR2 |
| 50 | PIK3R2 | 2.36E-29 | 1.34E-31 | 70 | 46 | AKT1, KIT, CSF1R, EGFR, ERBB2, SRC, SYK, GRB2, RAC1, FYN, PRKCA, IRS1, PTPN11, NGF, SOS1, LCK, PIK3CA, PTEN, PIK3R1, VAV1, IRS2, EDN1, PLCG1, CBL, PIK3CB, PIK3CG, CRK, PIK3CD, RAC2, GCG, GCGR, PDGFRB, INPP5D, GAST, PTK2, BDKRB2, PLCB1, ERBB4, CCK, TRH, VAV2, PLCE1, P2RY2, XBP1, TIAM1, PLCD3 |
| 51 | TP53 | 4.77E-29 | 2.76E-31 | 298 | 97 | GPX1, PTGS2, MAPK1, CREBBP, MAPK8, ESR1, IL6, JUN, UBC, AKT1, CASP8, STUB1, TXN, MYC, EP300, BRCA1, CHEK1, MAPK14, FOS, STAT3, VEGFA, BCL2, CCND1, SIN3A, MAPK11, HSPA8, TRIM28, EGFR, CREB1, SIRT1, ERBB2, CAV1, BIRC5, MMP2, ABCB1, APC, HSP90AA1, HRAS, SMARCA4, CARM1, SP1, HIF1A, GSK3B, RB1, IGF1, AR, TGFB1, SMAD2, CDK2, HDAC1, NOTCH1, PCNA, MDM2, PTEN, ATM, BDNF, DNMT1, IKBKB, NQO1, MAX, HSPA4, BCL2L1, MET, KAT2B, MAPK9, NR3C1, PRKCD, CD44, NCL, RBBP4, PLK1, SERPINE1, HDAC2, MSH2, GAPDH, CSNK2A1, TBP, DKK1, APEX1, WWOX, ABL1, YY1, TGFBR2, CASP6, PTK2, NGFR, BDKRB2, NEDD8, CSNK1D, YBX1, CSNK1E, PHB, CDK5, MSX1, HMGB1, CRYAB, S100B |
| 52 | LEP | 2.26E-28 | 1.33E-30 | 79 | 48 | PLA2G7, CREBBP, ESR1, RXRA, POMC, NCOA2, IL6, INS-IGF2, AKT1, IL1B, EP300, FOS, STAT3, VEGFA, PPARG, CREB1, ARNT, HIF1A, IGF1, JAK2, TGFB1, IRS1, PPARGC1A, MED12, ALB, SREBF1, LPL, PTPN1, SOCS3, MTOR, CRH, PRL, SST, GCG, NPY, CRP, MED15, GAL, MC4R, MCHR1, CCK, A2M, TRH, ADRB3, AGRP, UCP2, LEPR, GHRL |
| 53 | IRS2 | 4.21E-28 | 2.53E-30 | 47 | 37 | MAPK3, MAPK8, INS-IGF2, AKT1, NRAS, SIRT1, HRAS, IGF1, JAK2, GRB2, FOXO1, IL2, JAK1, IRS1, PTPN11, IL2RA, NGF, SOS1, LCK, PIK3CA, IL4, JAK3, PIK3R1, INSR, PTPN1, SOCS3, MTOR, PRL, PIK3CB, PIK3CG, CRK, PIK3CD, EPOR, PIK3R2, INPP5D, PRLR, NTRK1 |
| 54 | CBL | 3.09E-27 | 1.89E-29 | 120 | 58 | UBC, INS-IGF2, YWHAZ, UBA52, VEGFA, CD4, KIT, CSF1R, EGFR, EGF, SRC, IFNG, SYK, JAK2, GRB2, FYN, UBB, JAK1, IRS1, PTPN11, SOS1, LCK, PIK3CA, IL4, YWHAB, SHC1, CDC42, JAK3, PIK3R1, YWHAQ, INSR, VAV1, MET, PLCG1, FGFR2, YES1, SH3GL2, PIK3CB, PIK3CG, CRK, PIK3CD, HCK, BCAR1, EPOR, PIK3R2, B2M, ABL1, AP2M1, PDGFRB, CD247, PLCG2, PDGFB, PTK2B, VAV2, RET, VAV3, NTRK1, DNM2 |
| 55 | IL2RA | 4.08E-27 | 2.54E-29 | 60 | 41 | MAPK1, MAPK3, CREBBP, IL6, JUN, AKT1, MYC, MAPK14, FOS, STAT3, CD4, NRAS, CSF2, NFATC2, NFATC1, NFATC3, HRAS, MAP2K1, SYK, STAT1, JAK2, GRB2, IL2, JAK1, IRS1, PTPN11, LCK, PIK3CA, IL4, SHC1, JAK3, PIK3R1, IRS2, MAPK9, TNF, SOCS3, IL3, B2M, CD86, CD247, PTK2B |
| 56 | PLCG1 | 4.51E-27 | 2.86E-29 | 129 | 60 | MAPK3, CD4, KIT, EGFR, CREB1, EGF, ERBB2, SRC, SYK, STAT1, JAK2, GNA15, GRB2, RAC1, FYN, PRKCA, JAK1, NGF, SOS1, LCK, PIK3CA, PTEN, CDC42, PIK3R1, INSR, VAV1, DGKG, MET, PRKCD, YES1, CBL, PIK3CB, PIK3CG, PIK3CD, HCK, PLCB2, PLCB3, EPOR, PIK3R2, ABL1, PPP3CA, PDGFRB, CAMK2A, GSN, PLCG2, PDGFB, PLCB1, PPP3CB, ERBB4, TYROBP, PLCB4, VAV2, RET, VAV3, PLCE1, NTRK1, PLCD3, WAS, PPP3CC, NTRK2 |
| 57 | STAT1 | 5.19E-27 | 3.35E-29 | 109 | 55 | MAPK1, MAPK3, CREBBP, IL6, UBC, AKT1, TNFRSF1A, CASP8, TRADD, RIPK1, FADD, CAMK2B, EP300, MAPK14, FOS, STAT3, BCL2, KIT, EGFR, CSF2, EGF, SRC, IFNG, SMARCA4, IL23A, JAK2, IL2, FYN, JAK1, SDC2, PTPN11, IL2RA, LCK, JAK3, IL12B, BCL2L1, PRKCD, SOCS3, PLCG1, YES1, PRL, CXCR4, IL6R, IL6ST, EPOR, IFIT1, CXCL12, CAMK2G, PDGFRB, GNB2L1, IL17A, PDGFB, CSF2RB, CSF2RA, KPNA1 |
| 58 | PIK3CB | 1.36E-26 | 8.93E-29 | 74 | 45 | IL6, AKT1, VEGFA, NOS3, EGFR, ERBB2, SRC, HRAS, SYK, IGF1, JAK2, RAC1, FYN, JAK1, IRS1, PTPN11, NGF, LCK, PIK3CA, PTEN, PIK3R1, VAV1, RAC3, IRS2, BCL2L1, PLCG1, MTOR, CBL, PIK3CG, HCK, LPAR2, PLCB2, PLCB3, PIK3R2, INPP5D, PLCG2, PLCB1, ERBB4, TYROBP, PLCB4, VAV2, PLCE1, TIAM1, PLCD3, AKT3 |
| 59 | EP300 | 1.67E-26 | 1.12E-28 | 158 | 66 | CREBBP, ESR1, RXRA, PPARA, NCOA2, TP53, JUN, AKT1, MYC, BRCA1, MAPK14, FOS, STAT3, CEBPB, VEGFA, LEF1, PPARG, CREB1, SIRT1, CSF2, NFATC2, RELA, ARNT, CARM1, CTNNB1, SMAD3, SP1, HIF1A, RB1, STAT1, AR, SMAD4, TGFB1, NFKB1, LEP, SMAD2, HDAC1, CEBPD, FOXO1, NOTCH1, HDAC3, PCNA, MDM2, RORA, PPARGC1A, MED12, SREBF1, MAX, LPL, TCF7L2, KAT2B, CLOCK, NPAS2, NCL, EGR2, SKP1, ATF4, APEX1, ARNTL, EPAS1, MED15, YY1, SLC2A1, CITED2, CABIN1, HIST1H3A |
| 60 | GNA15 | 2.90E-26 | 1.97E-28 | 89 | 49 | PLA2G4A, ESR1, MAPK14, ANXA1, KNG1, APP, IL8, PIK3CA, GNAI2, F2R, EDN1, PLCG1, LPAR2, CXCR2, PLCB2, GCG, PLCB3, GNAS, GCGR, GNRH1, CASR, EDNRA, GAST, CHRM2, EDNRB, BDKRB2, PLCB1, GNG2, NMU, MCHR1, TBXA2R, PLCB4, CCK, TRH, RGS2, GRP, NTS, ADRBK1, PTAFR, P2RY2, TAC1, GNRHR, LTB4R, HCRT, GRK5, TACR1, CCKAR, CHRM3, OXTR |
| 61 | MYC | 3.42E-26 | 2.37E-28 | 188 | 72 | MAPK1, MAPK3, CREBBP, MAPK8, ESR1, TP53, JUN, UBC, INS-IGF2, AKT1, EP300, BRCA1, FOS, STAT3, BCL2, CCND1, SIN3A, E2F1, TRIM28, SIRT1, ERBB2, BIRC5, RELA, SMARCA4, CTNNB1, SP1, GSK3B, RB1, SMAD4, NFKB1, SMAD2, CDK2, HDAC1, CEBPD, CCNT1, IL2, JAK1, NOTCH1, HDAC3, IL2RA, LCK, SHMT1, CDKN1B, JAK3, MAX, HSPA4, TCF7L2, KAT2B, MMP9, CD44, NCL, CTBP1, SKP1, HSPD1, GAPDH, ITGB1, TBP, DKK1, ABL1, YY1, PRDX1, PDGFRB, ITGA6, ACTB, SMARCA2, ITGB4, SLC2A1, DVL3, WNT5A, HMGA1, CLU, PFKM |
| 62 | ERBB2 | 4.61E-26 | 3.24E-28 | 79 | 46 | PTGS2, ESR1, TP53, UBC, STUB1, UBA52, MYC, STAT3, VEGFA, CCND1, NRAS, EGFR, EGF, BIRC5, CDH1, HBEGF, HSP90AA1, SRC, HRAS, CTNNB1, MAP2K1, IGF1, JAK2, GRB2, RAC1, UBB, PTPN11, SOS1, PIK3CA, SHC1, PIK3R1, MAX, HSPA4, ERBB3, PLCG1, CD44, PIK3CB, PIK3CG, PIK3CD, RAC2, PIK3R2, PTK2, ERBB4, NRG1, IGHG1, RHOB |
| 63 | NPY | 8.51E-26 | 6.08E-28 | 102 | 52 | POMC, INS-IGF2, FOS, ANXA1, KNG1, IGF1, LEP, NGF, APP, GNAI2, ADCY2, CRH, CCR5, SST, ADCY9, ADCY8, CXCR2, ADORA1, SSTR5, PENK, GNRH1, CASR, GAL, OPRM1, CHRM2, BDKRB2, MC4R, DRD2, HTR1A, NMU, MCHR1, PTGER3, MTNR1A, ADRA2A, RGS4, PDYN, DRD3, NPY1R, DRD4, PNOC, NPY2R, HRH3, CHRM4, SSTR2, NMS, OPRD1, GPR55, ADRA2C, HTR1B, NMUR2, HCAR2, GHRL |
| 64 | ESR1 | 1.40E-25 | 1.01E-27 | 163 | 66 | CYP1A1, PTGS2, MAPK1, CREBBP, RXRA, PPARA, NCOA2, IL6, TP53, JUN, UBC, INS-IGF2, AKT1, STUB1, MYC, EP300, BRCA1, MAPK14, FOS, CEBPB, VEGFA, CCND1, MAPK11, NOS3, HSPA8, EGFR, PPARG, ERBB2, CAV1, RELA, HSP90AA1, SRC, SOD1, SMARCA4, SP1, RB1, IGF1, GNA15, SMAD4, LEP, GRB2, HDAC1, FOXO1, CCNT1, ESR2, IRS1, PCNA, MDM2, SOS1, PIK3CA, RORA, PIK3R1, GNAI2, HSPA4, NR4A2, NR2F1, PRL, HDAC4, BCAR1, MSH2, TBP, NEDD8, ARHGDIA, GRIP1, XBP1, BLOC1S1 |
| 65 | DRD2 | 4.28E-25 | 3.16E-27 | 75 | 44 | POMC, KNG1, APP, GNAI2, ADCY2, CCR5, SST, ADCY9, CXCR2, ADORA1, ADCY6, SSTR5, NPY, ADORA2A, PENK, CASR, GAL, ADCY1, OPRM1, CHRM2, BDKRB2, ADCY5, GNG2, HTR1A, MCHR1, MTNR1A, ADRA2A, RGS4, PDYN, DRD3, NPY1R, DRD4, PNOC, NPY2R, HRH3, CHRM4, SSTR2, NMS, OPRD1, GPR55, ADRA2C, HTR1B, NMUR2, HCAR2 |
| 66 | SST | 6.60E-25 | 4.94E-27 | 90 | 48 | POMC, FOS, ANXA1, KNG1, LEP, APP, GNAI2, ADCY2, CCR5, ADCY9, ADCY8, CXCR2, GCG, ADORA1, ADCY6, SSTR5, NPY, PENK, CASR, GAL, GAST, ADCY1, OPRM1, CHRM2, BDKRB2, DRD2, HTR1A, NMU, MCHR1, CCK, MTNR1A, ADRA2A, RGS4, PDYN, DRD3, NPY1R, DRD4, PNOC, NPY2R, NTS, HRH3, CHRM4, SSTR2, NMS, OPRD1, TAC1, NMUR2, HCAR2 |
| 67 | IFNG | 8.35E-25 | 6.34E-27 | 83 | 46 | MAPK1, MAPK3, HMOX1, IL6, IDO1, JUN, IL1B, CAMK2B, FOS, STAT3, CD4, NFATC2, NFATC1, NFATC3, IL23A, STAT1, JAK2, TGFB1, IL2, NOS2, JAK1, PTPN11, IL8, PIK3CA, IL4, CDKN1B, PIK3R1, CTLA4, ICAM1, IL12B, PTPN1, MAPK9, PRKCD, SOCS3, CBL, FN1, CCR5, CXCR4, VCAM1, IL3, IL18, IL6ST, PTPN6, CD86, CD247, HLA-DRB1 |
| 68 | YES1 | 9.97E-25 | 7.68E-27 | 66 | 41 | MAPK1, AKT1, MAPK14, STAT3, NRAS, KIT, EGFR, EGF, SRC, HRAS, SYK, STAT1, JAK2, GRB2, RAC1, FYN, PRKCA, PTPN11, LCK, PIK3CA, CDC42, VAV1, PRKCD, PLCG1, CBL, CRK, HCK, BCAR1, PTPN6, ABL1, RASA1, PDGFRB, CD247, PDGFB, CTNND1, VCL, PTK2B, WNT5A, PTPN13, EPHB2, DCC |
| 69 | CREB1 | 1.21E-24 | 9.49E-27 | 63 | 40 | PTGS2, MAPK1, MAPK3, CREBBP, TP53, JUN, INS-IGF2, AKT1, CAMK2B, EP300, MAPK14, FOS, BCL2, CCND1, MAPK11, GSK3B, LEP, GRB2, HDAC1, CEBPD, NGF, SOS1, ATM, SHC1, BDNF, RORA, PPARGC1A, CALM1, NR3C1, PLCG1, CALM2, CALM3, ADCY8, CAMK2G, CAMK2A, ADCY1, GDNF, RET, PRKACA, NTF3 |
| 70 | GNAI2 | 1.50E-24 | 1.20E-26 | 107 | 52 | MAPK1, MAPK3, ESR1, POMC, AKT1, MAPK14, ANXA1, CD4, SRC, HRAS, GNA15, FYN, IL8, CDC42, PDE4A, F2R, EDN1, ADCY2, CCR5, CXCR4, SST, ADCY9, ADCY8, CXCR2, BCAR1, ADORA1, SSTR5, NPY, GNAS, PENK, CXCL12, GAL, ADCY1, ADCY4, OPRM1, EDNRB, BDKRB2, ADCY5, GNG2, DRD2, HTR1A, PTGER3, MTNR1A, ADRA2A, RGS4, PDYN, DRD3, HRH3, SSTR2, OPRD1, HTR1B, PDE8A |
| 71 | JAK1 | 1.50E-24 | 1.20E-26 | 103 | 51 | MAPK1, MAPK3, IL6, AKT1, TNFRSF1A, MYC, MAPK14, STAT3, MAPK11, NRAS, HRAS, IFNG, MAP2K1, SYK, STAT1, JAK2, GRB2, FOXO1, IL2, FYN, IRS1, PTPN11, IL2RA, SOS1, LCK, PIK3CA, IL4, SHC1, JAK3, PIK3R1, VAV1, IRS2, BCL2L1, PTPN1, MAPK9, SOCS3, PLCG1, CBL, PIK3CB, PRKCZ, IL6R, HCK, IL6ST, PTPN6, EPOR, IFIT1, GNB2L1, INPP5D, CSF2RB, PTK2B, IL10RA |
| 72 | MAPK14 | 1.74E-24 | 1.42E-26 | 146 | 61 | PTGS2, PLA2G4A, MAPK1, MAPK3, CREBBP, ESR1, TP53, JUN, RIPK1, YWHAZ, EP300, FOS, STAT3, CEBPB, MAPK11, CREB1, NFATC2, NFATC1, RELA, SRC, CTNNB1, SMAD3, MAP2K1, SYK, STAT1, GNA15, NFKB1, MAP3K7, RAC1, IL2, FYN, JAK1, NFKBIA, IL2RA, LCK, CASP3, IL4, SHC1, CDC42, TH, JAK3, PPARGC1A, GNAI2, RAC3, MAX, EDN1, KAT2B, SOCS3, YES1, DUSP6, SRF, CDH2, HCK, PLCB2, EPOR, ABL1, EDNRB, HNRNPK, GDI1, CYBB, SLC9A1 |
| 73 | EDNRA | 2.19E-24 | 1.81E-26 | 67 | 41 | AKT1, ANXA1, NOS3, EGFR, EGF, KNG1, JAK2, GNA15, RAC1, APP, PIK3CA, F2R, EDN1, ADCY2, GCG, PLCB3, GCGR, GNRH1, CASR, GAST, EDNRB, BDKRB2, NMU, TBXA2R, CCK, TRH, RGS2, GRP, NTS, ADRBK1, PTAFR, NMS, P2RY2, TAC1, GNRHR, GRK5, TACR1, CCKAR, CHRM3, SLC9A1, OXTR |
| 74 | GSTA2 | 4.08E-24 | 3.47E-26 | 55 | 37 | GSTM1, CYP2E1, GSTT1, CYP1A2, CYP1A1, ADH7, GSTA4, GSTA1, GSTA3, GSTP1, ALDH3A1, ALDH1A3, CYP2B6, CYP3A4, CYP3A43, CYP3A5, CYP2C19, CYP2C9, CYP2C18, CYP2C8, GGT7, ADH1B, ADH4, ADH1A, CYP1B1, GSTM2, GSTM3, GSTT2B, GSTO1, GSTZ1, GSTO2, GSS, GPX1, GGT1, ADH5, MAPK1, NFE2L2 |
| 75 | PDGFRB | 4.08E-24 | 3.43E-26 | 52 | 36 | UBC, AKT1, MYC, STAT3, VEGFA, NRAS, SRC, HRAS, STAT1, JAK2, GRB2, RAC1, FYN, PTPN11, SOS1, LCK, PIK3CA, PTEN, SHC1, PIK3R1, ITGAV, PTPN1, PLCG1, YES1, CBL, CRK, HCK, BCAR1, PIK3R2, ABL1, RASA1, GRB10, PTK2, PDGFB, SLC9A3R1, SHC2 |
| 76 | MAP2K1 | 6.94E-24 | 5.97E-26 | 44 | 33 | PLA2G4A, MAPK1, MAPK3, YWHAZ, MAPK14, STAT3, NRAS, ERBB2, SRC, HRAS, NFKB1, GRB2, IL2, JAK1, PTPN11, IL2RA, SOS1, LCK, PIK3CA, YWHAB, SHC1, RAF1, JAK3, PIK3R1, YWHAE, YWHAH, ERBB3, KSR1, PRKCD, PPP2R1A, SHH, DCC, EPHB1 |
| 77 | PTPN1 | 1.07E-23 | 9.35E-26 | 50 | 35 | INS-IGF2, PLD2, AKT1, TXN, STAT3, CSF1R, EGFR, EGF, CAV1, SRC, IFNG, CTNNB1, IGF1, JAK2, LEP, GRB2, JAK1, IRS1, PIK3CA, SHC1, PIK3R1, INSR, IRS2, MET, FN1, CRK, CDH2, BCAR1, CSNK2A1, PDGFRB, PDGFB, YBX1, LEPR, CTTN, ITGA2B |
| 78 | ADORA1 | 1.11E-23 | 9.78E-26 | 53 | 36 | POMC, KNG1, GNAI2, ADCY2, CCR5, SST, ADCY9, ADCY8, CXCR2, ADCY6, SSTR5, NPY, PENK, CASR, GAL, ADCY1, OPRM1, CHRM2, BDKRB2, DRD2, HTR1A, MCHR1, PTGER3, MTNR1A, ADRA2A, RGS4, PDYN, DRD3, DRD4, PNOC, NPY2R, HRH3, CHRM4, SSTR2, HTR1B, HCAR2 |
| 79 | PIK3R3 | 2.29E-23 | 2.05E-25 | 45 | 33 | AKT1, SRC, SYK, RAC1, FYN, IRS1, PTPN11, LCK, PIK3CA, PTEN, PIK3R1, VAV1, IRS2, EDN1, PLCG1, PIK3CB, PIK3CG, CRK, RAC2, PLCB2, GCG, PLCB3, PIK3R2, GCGR, PDGFRB, INPP5D, PTK2, BDKRB2, PLCB1, PLCB4, CCK, TRH, PLCE1 |
| 80 | GSTA4 | 2.96E-23 | 2.69E-25 | 54 | 36 | GSTM1, CYP2E1, GSTT1, CYP1A2, CYP1A1, ADH7, GSTA2, GSTA1, GSTA3, GSTP1, ALDH3A1, CYP2B6, CYP3A4, CYP3A43, CYP3A5, CYP2C19, CYP2C9, CYP2C18, CYP2C8, GGT7, ADH1B, ADH4, ADH1A, CYP1B1, GSTM2, GSTM3, GSTT2B, GSTO1, GSTZ1, GSTO2, ADH6, GSS, GPX1, GGT1, ADH5, MAPK1 |
| 81 | GSTP1 | 2.97E-23 | 2.73E-25 | 51 | 35 | GSTM1, CYP2E1, GSTT1, CYP1A2, CYP1A1, ADH7, GSTA2, GSTA4, GSTA1, GSTA3, ALDH3A1, ALDH1A3, CYP2B6, CYP3A4, CYP3A43, CYP3A5, CYP2C19, CYP2C9, CYP2C18, CYP2C8, ADH1B, ADH4, ADH1A, CYP1B1, GSTM2, GSTM3, GSTT2B, GSTO1, GSTZ1, GSTO2, GSS, GPX1, GGT1, ADH5, MAPK8 |
| 82 | F2R | 1.76E-22 | 1.64E-24 | 80 | 43 | MAPK1, MAPK3, ANXA1, KNG1, GNA15, APP, PIK3CA, GNAI2, EDN1, LPAR2, PLCB2, GCG, PLCB3, GCGR, GNRH1, CASR, EDNRA, GAST, EDNRB, BDKRB2, PLCB1, NMU, TBXA2R, PLCB4, CCK, TRH, RGS2, GRP, NTS, ADRBK1, PTAFR, NMS, P2RY2, TAC1, DNM1, GNRHR, LTB4R, DNM2, GRK5, TACR1, CCKAR, CHRM3, OXTR |
| 83 | NFATC2 | 1.83E-22 | 1.72E-24 | 44 | 32 | PTGS2, CREBBP, MAPK8, JUN, FASLG, EP300, MAPK14, FOS, PPARG, CSF2, NFATC1, NFATC3, IFNG, GSK3B, IL2, IL2RA, CASP3, IL4, CDC42, CTLA4, YWHAQ, MAPK9, CREM, PRKCZ, IL3, PPP3CA, EGR3, PPP3R1, PPP3CB, PPIA, CABIN1, FOXP2 |
| 84 | GSTA1 | 2.14E-22 | 2.06E-24 | 53 | 35 | GSTM1, CYP2E1, GSTT1, CYP1A2, CYP1A1, ADH7, GSTA2, GSTA4, GSTA3, GSTP1, ALDH3A1, CYP2B6, CYP3A4, CYP3A43, CYP3A5, CYP2C19, CYP2C9, CYP2C18, CYP2C8, GGT7, ADH1B, ADH4, ADH1A, CYP1B1, GSTM2, GSTM3, GSTT2B, GSTO1, GSTZ1, GSTO2, GSS, GPX1, GGT1, ADH5, MAPK1 |
| 85 | GSTA3 | 2.14E-22 | 2.06E-24 | 53 | 35 | GSTM1, CYP2E1, GSTT1, CYP1A2, CYP1A1, ADH7, GSTA2, GSTA4, GSTA1, GSTP1, ALDH3A1, CYP2B6, CYP3A4, CYP3A43, CYP3A5, CYP2C19, CYP2C9, CYP2C18, CYP2C8, GGT7, ADH1B, ADH4, ADH1A, CYP1B1, GSTM2, GSTM3, GSTT2B, GSTO1, GSTZ1, GSTO2, GSS, GPX1, GGT1, ADH5, MAPK1 |
| 86 | PRKCZ | 3.71E-22 | 3.62E-24 | 60 | 37 | UBC, INS-IGF2, AKT1, TNFRSF1A, RIPK1, UBA52, NFATC2, NFATC1, RELA, SRC, CTNNB1, GSK3B, TGFB1, NFKB1, GRB2, IL2, UBB, JAK1, NFKBIA, IRS1, PTPN11, NGF, LCK, PIK3CA, RAF1, CDC42, IKBKB, JAK3, PIK3R1, INSR, MMP9, MAPK9, MSH2, NGFR, CTNNA1, VAMP2, TIAM1 |
| 87 | HIF1A | 4.14E-22 | 4.08E-24 | 97 | 47 | HMOX1, CREBBP, RXRA, PPARA, NCOA2, TP53, JUN, UBC, INS-IGF2, AKT1, UBA52, EP300, STAT3, VEGFA, EGFR, ABCB1, HSP90AA1, ARNT, CARM1, SP1, SMAD4, LEP, HDAC1, UBB, NOTCH1, HDAC3, MDM2, RORA, PPARGC1A, HSPA4, EDN1, MTOR, NPAS2, CXCR4, SERPINE1, PKM, ADM, APEX1, EPAS1, CXCL12, GNB2L1, ITGB2, SLC2A1, CITED2, ALDOA, TF, FURIN |
| 88 | IL1B | 4.41E-22 | 4.40E-24 | 67 | 39 | PLA2G1B, PTGS2, MAPK1, IL6, JUN, FOS, VEGFA, CSF2, FGF2, MMP2, RELA, IL1A, IFNG, IL23A, IGF1, JAK2, NFKB1, LEP, IL2, NOS2, NFKBIA, NGF, CASP3, IL8, PIK3CA, IL4, PIK3R1, ICAM1, MYD88, IL12B, MMP9, CCL2, FN1, ADRB2, VCAM1, IL18, NLRP3, IL1R1, MMP3 |
| 89 | IL8 | 4.49E-22 | 4.53E-24 | 57 | 36 | MAPK1, IL6, JUN, IL1B, FOS, STAT3, VEGFA, IL10, EGFR, NFATC1, RELA, IL1A, HBEGF, IFNG, CTNNB1, GNA15, NFKB1, RAC1, IL2, SDC2, GNAI2, ALB, MMP9, CCL2, SDC1, CCR5, CXCR4, HCK, LPAR2, CXCR2, ATF4, CRP, TLR2, RAB5A, ADRBK1, DNM1 |
| 90 | CCND1 | 5.29E-22 | 5.45E-24 | 123 | 53 | MAPK1, CREBBP, ESR1, IL6, TP53, JUN, UBC, INS-IGF2, AKT1, UBA52, MYC, BRCA1, FOS, STAT3, VEGFA, LEF1, E2F1, EGFR, PPARG, CREB1, EGF, ERBB2, PTH, FGF2, CDH1, SRC, HRAS, CTNNB1, GSK3B, RB1, IGF1, AR, TGFB1, CDK2, RAC1, HDAC1, FOXO1, UBB, NOTCH1, HDAC3, PCNA, PIK3CA, PTEN, ATM, CDKN1B, CDC42, PIK3R1, TCF7L2, KAT2B, MTOR, CTBP1, SKP1, GAST |
| 91 | PDGFB | 5.29E-22 | 5.46E-24 | 45 | 32 | AKT1, STAT3, NRAS, SRC, HRAS, STAT1, JAK2, TGFB1, GRB2, RAC1, FYN, PTPN11, SOS1, LCK, PIK3CA, PTEN, PIK3R1, ITGAV, PTPN1, PLCG1, YES1, CBL, CRK, HCK, BCAR1, ABL1, RASA1, PDGFRB, GRB10, THBS1, DNM2, FURIN |
| 92 | FGF2 | 5.60E-22 | 5.90E-24 | 51 | 34 | PTGS2, IL6, JUN, AKT1, IL1B, FOS, STAT3, GPC1, VEGFA, CCND1, NOS3, EGFR, EGF, SRC, IGF1, TGFB1, SDC2, NGF, PCNA, BDNF, SDC4, MMP9, GJA1, FGFR2, SDC1, FN1, CXCR4, MMP3, SDC3, GFAP, GDNF, TF, PLAT, NRP1 |
| 93 | DRD3 | 5.60E-22 | 5.90E-24 | 51 | 34 | POMC, KNG1, APP, GNAI2, ADCY2, CCR5, SST, CXCR2, ADORA1, ADCY6, NPY, PENK, CASR, GAL, OPRM1, CHRM2, BDKRB2, ADCY5, DRD2, HTR1A, MCHR1, ADRA2A, RGS4, PDYN, NPY1R, DRD4, PNOC, NPY2R, HRH3, CHRM4, OPRD1, ADRA2C, HTR1B, NMUR2 |
| 94 | GSTM1 | 5.61E-22 | 5.98E-24 | 48 | 33 | CYP2E1, GSTT1, CYP1A2, CYP1A1, ADH7, GSTA2, GSTA4, GSTA1, GSTA3, GSTP1, ALDH3A1, ALDH1A3, CYP2B6, CYP3A4, CYP3A43, CYP3A5, CYP2C19, CYP2C9, CYP2C18, CYP2C8, ADH1B, ADH4, ADH1A, CYP1B1, GSTM2, GSTM3, GSTT2B, GSTO1, GSTZ1, GSTO2, GSS, GPX1, GGT1 |
| 95 | JAK3 | 7.83E-22 | 8.43E-24 | 61 | 37 | MAPK1, MAPK3, AKT1, MYC, MAPK14, STAT3, VEGFA, MAPK11, NRAS, HRAS, MAP2K1, SYK, STAT1, GRB2, IL2, FYN, JAK1, IRS1, PTPN11, IL2RA, SOS1, LCK, PIK3CA, IL4, SHC1, PIK3R1, IRS2, BCL2L1, MAPK9, SOCS3, CBL, CXCR4, PRKCZ, PTPN6, INPP5D, PTK2B, LEPR |
| 96 | ADH1A | 1.34E-21 | 1.46E-23 | 35 | 28 | GSTM1, CYP2E1, UGT1A6, GSTA2, GSTA4, GSTA1, GSTA3, GSTP1, ALDH3A1, ALDH1A3, CYP2B6, ADH1B, GSTM2, GSTM3, GSTT2B, GSTO1, GSTZ1, GSTO2, ALDH1A1, COMT, ALDH3A2, ALDH2, ALDH1A2, MAOA, ALDH9A1, MAOB, ALDH7A1, ALDH1B1 |
| 97 | NOS3 | 1.49E-21 | 1.64E-23 | 46 | 32 | PTGS2, HMOX1, ESR1, SOD2, IL6, JUN, INS-IGF2, AKT1, VEGFA, SIRT1, CAV1, KNG1, FGF2, HSP90AA1, SRC, NOS2, PIK3CA, BDNF, ICAM1, HSPA4, CALM1, EDN1, MMP9, PIK3CB, CALM2, PIK3CG, CALM3, PIK3CD, CRP, ACE, EDNRA, NOS1 |
| 98 | NFKBIA | 1.77E-21 | 1.99E-23 | 69 | 39 | MAPK1, MAPK3, UBC, AKT1, TNFRSF1A, RIPK1, TRAF1, IL1B, UBA52, MAPK14, RELA, SRC, IL23A, SYK, JAK2, NFKB1, HDAC1, UBB, PRKCA, HDAC3, NGF, LCK, APP, PIK3CA, IKBKG, IKBKB, PIK3R1, ICAM1, IL12B, VCP, PRKCD, PRKCZ, SKP1, CSNK2A1, ABL1, BTRC, IL17A, NGFR, PTPN13 |
| 99 | GAL | 1.77E-21 | 1.99E-23 | 69 | 39 | POMC, INS-IGF2, FOS, KNG1, LEP, APP, TH, GNAI2, ADCY2, CCR5, SST, CXCR2, ADORA1, SSTR5, NPY, PENK, CASR, OPRM1, CHRM2, BDKRB2, DRD2, HTR1A, NMU, MCHR1, MTNR1A, ADRA2A, PDYN, DRD3, NPY1R, DRD4, PNOC, NPY2R, HRH3, SSTR2, NMS, OPRD1, HTR1B, NMUR2, HCAR2 |
| 100 | CCK | 2.03E-21 | 2.30E-23 | 96 | 46 | FOS, ANXA1, KNG1, GNA15, LEP, APP, PIK3CA, PIK3R1, ALB, F2R, EDN1, SST, PLCB2, GCG, PLCB3, PIK3R2, GCGR, GNRH1, CASR, EDNRA, GAST, EDNRB, BDKRB2, PLCB1, NMU, MCHR1, TBXA2R, TRH, RGS2, GRP, NTS, ADRBK1, PTAFR, NMS, P2RY2, TAC1, NMUR2, GNRHR, LTB4R, HCRT, GRK5, TACR1, GHRL, CCKAR, CHRM3, OXTR |
| 101 | YWHAZ | 2.30E-21 | 2.64E-23 | 73 | 40 | MAPK1, MAPK3, AKT1, AANAT, CHEK1, MAPK14, MAPK11, NRAS, EGFR, HRAS, CTNNB1, MAP2K1, JAK2, FOXO1, PRKCA, YWHAG, PIK3CA, YWHAB, SHC1, RAF1, CDKN1B, TH, PIK3R1, YWHAE, YWHAQ, YWHAH, BAD, KSR1, MTOR, CBL, FN1, HDAC4, ITGB1, MAPT, ITGA6, ITGB4, PPP2R1A, RAN, CSF2RB, KPNA1 |
| 102 | CDC42 | 2.34E-21 | 2.71E-23 | 145 | 57 | UBC, AKT1, PAFAH1B1, UBA52, MAPK14, GPC1, CCND1, MAPK11, EGFR, EGF, NFATC2, CDH1, APC, SRC, CTNNB1, ABCA1, RAC1, FYN, UBB, LCK, PIK3CA, BDNF, PIK3R1, GNAI2, VAV1, CALM1, MET, MAPK9, PLCG1, MTOR, YES1, CBL, CALM2, CXCR4, PRKCZ, CALM3, CDH2, RAC2, BCAR1, ABL1, CXCL12, CD86, PTK2, CTNNA1, GNG2, CTNND1, ARHGDIA, VAV2, JUP, VAV3, EPHB2, EFNB2, TIAM1, RASGRF1, DCC, SLIT2, WAS |
| 103 | EDNRB | 3.52E-21 | 4.11E-23 | 70 | 39 | MAPK14, ANXA1, CAV1, KNG1, GNA15, APP, PIK3CA, GNAI2, F2R, EDN1, GCG, GCGR, GNRH1, CASR, EDNRA, GAST, BDKRB2, PLCB1, GDNF, GNG2, NMU, TBXA2R, CCK, TRH, RGS2, GRP, NTS, ADRBK1, PTAFR, P2RY2, TAC1, GNRHR, SOX10, HCRT, GRK5, TACR1, CCKAR, CHRM3, OXTR |
| 104 | P2RY2 | 3.60E-21 | 4.24E-23 | 63 | 37 | ANXA1, KNG1, SRC, GNA15, APP, PIK3CA, PIK3R1, F2R, EDN1, LPAR2, PLCB2, GCG, PLCB3, PIK3R2, GNRH1, CASR, EDNRA, GAST, EDNRB, BDKRB2, PLCB1, TBXA2R, PLCB4, CCK, TRH, RGS2, GRP, ADRBK1, PTAFR, TAC1, GNRHR, LTB4R, HCRT, GRK5, TACR1, CCKAR, CHRM3 |
| 105 | PIK3CG | 5.24E-21 | 6.24E-23 | 67 | 38 | AKT1, VEGFA, NOS3, NRAS, KIT, EGFR, ERBB2, SRC, HRAS, SYK, IGF1, JAK2, RAC1, FYN, IRS1, PTPN11, PTEN, PIK3R1, VAV1, RAC3, IRS2, PLCG1, MTOR, CBL, PIK3CB, PLCB2, PLCB3, PIK3R2, INPP5D, PLCG2, PLCB1, ERBB4, GNG2, PLCB4, VAV2, PLCE1, PLCD3, AKT3 |
| 106 | TRH | 5.83E-21 | 7.00E-23 | 82 | 42 | FOS, ANXA1, KNG1, GNA15, LEP, APP, PIK3CA, PIK3R1, F2R, EDN1, PRL, GCG, PIK3R2, GCGR, GNRH1, CASR, EDNRA, GAST, EDNRB, BDKRB2, MC4R, NMU, MCHR1, PLCB4, CCK, GRP, NTS, ADRBK1, PTAFR, AGRP, NMS, P2RY2, TAC1, NMUR2, GNRHR, LTB4R, HCRT, GRK5, TACR1, CCKAR, CHRM3, OXTR |
| 107 | MMP9 | 6.44E-21 | 7.81E-23 | 57 | 35 | IL6, JUN, INS-IGF2, IL1B, MYC, STAT3, VEGFA, LEF1, NOS3, EGFR, EGF, FGF2, TGFBI, RELA, HBEGF, SRC, CTNNB1, TGFB1, NFKB1, RAC1, IL8, RAC3, MAX, SDC4, CCL2, SDC1, CD44, PRKCZ, CDH2, CXCL12, PLG, ITGB2, THBS1, PLAT, CYBB |
| 108 | FOXO1 | 9.56E-21 | 1.17E-22 | 51 | 33 | CREBBP, ESR1, SOD2, IL6, UBC, INS-IGF2, AKT1, YWHAZ, EP300, STAT3, CEBPB, CCND1, SIRT1, CTNNB1, IGF1, AR, SMAD4, SMAD2, CDK2, JAK1, YWHAG, YWHAB, CDKN1B, PPARGC1A, YWHAE, YWHAQ, YWHAH, IRS2, MTOR, IL6R, IAPP, AGRP, AKT3 |
| 109 | GSTM2 | 1.06E-20 | 1.31E-22 | 45 | 31 | GSTM1, CYP2E1, CYP1A2, CYP1A1, ADH7, GSTA2, GSTA4, GSTA1, GSTA3, GSTP1, ALDH3A1, CYP2B6, CYP3A4, CYP3A43, CYP3A5, CYP2C19, CYP2C9, CYP2C18, CYP2C8, ADH1B, ADH1A, CYP1B1, GSTM3, GSTT2B, GSTO1, GSTZ1, GSTO2, GSS, GPX1, GGT1, ADH5 |
| 110 | AGTR1 | 4.41E-20 | 5.50E-22 | 56 | 34 | NOS3, KNG1, GNA15, APP, PIK3CA, PIK3R1, F2R, EDN1, GCG, PIK3R2, GCGR, GNRH1, CASR, EDNRA, EDNRB, BDKRB2, PLCB1, TBXA2R, CCK, TRH, RGS2, GRP, NTS, ADRBK1, PTAFR, TAC1, GNRHR, LTB4R, HCRT, GRK5, TACR1, CCKAR, CHRM3, OXTR |
| 111 | VAV1 | 4.47E-20 | 5.62E-22 | 102 | 46 | MAPK1, IL6, UBC, AKT1, CD4, KIT, HRAS, SYK, JAK2, GRB2, RAC1, FYN, JAK1, PTPN11, SOS1, LCK, PIK3CA, CDC42, PIK3R1, RAC3, PLCG1, YES1, CBL, PIK3CB, RHOA, PIK3CG, CXCR4, IL6R, PIK3CD, RAC2, IL6ST, PTPN6, PIK3R2, CXCL12, CD86, CD247, PLCG2, ITGB2, HNRNPK, PTK2B, VAV2, VAV3, EZH2, DNM2, RHOB, WAS |
| 112 | ADH1B | 6.19E-20 | 7.86E-22 | 38 | 28 | GSTM1, CYP2E1, UGT1A6, GSTA2, GSTA4, GSTA1, GSTA3, GSTP1, ALDH3A1, CYP2B6, ADH1A, GSTM2, GSTM3, GSTT2B, GSTO1, GSTZ1, GSTO2, ALDH1A1, COMT, UGT1A1, ALDH3A2, ALDH2, ALDH1A2, MAOA, ALDH9A1, MAOB, ALDH7A1, ALDH1B1 |
| 113 | HCK | 6.96E-20 | 8.91E-22 | 60 | 35 | IL6, MAPK14, STAT3, MAPK11, EGFR, EGF, SRC, SYK, GRB2, FYN, JAK1, PTPN11, SOS1, LCK, IL8, PRKCD, PLCG1, YES1, CBL, PIK3CB, IL6R, BCAR1, IL6ST, ABL1, RASA1, PDGFRB, PLCG2, PDGFB, ITGB2, PTK2B, VAV2, PTPN13, EPHB2, EPHB1, WAS |
| 114 | MAOA | 7.50E-20 | 9.69E-22 | 28 | 24 | ADH7, ALDH1A3, CYP3A4, CYP3A43, CYP3A5, CYP2C19, CYP2C9, CYP2C8, ADH1B, ADH4, ADH1A, ADH6, COMT, CYP2D6, ALDH3A2, ALDH2, ALDH9A1, ALDH1B1, AOX1, DDC, DBH, IDO1, AANAT, SAT1 |
| 115 | INSR | 7.57E-20 | 9.87E-22 | 47 | 31 | MAPK1, MAPK3, INS-IGF2, AKT1, CAV1, HRAS, IGF1, JAK2, GRB2, IRS1, PTPN11, SOS1, PIK3CA, SHC1, PIK3R1, IRS2, CALM1, PTPN1, SOCS3, PLCG1, CBL, CALM2, PRKCZ, CALM3, PTPN6, GRB10, INPP5D, PPP1CC, SHC2, PTPRA, IRS4 |
| 116 | IL10 | 9.13E-20 | 1.20E-21 | 33 | 26 | PTGS2, HMOX1, IL6, JUN, FASLG, FOS, STAT3, CD4, CSF2, IL23A, SMAD4, TGFB1, IL2, NOS2, IL8, IL4, CTLA4, ICAM1, IL12B, CCL2, CCR5, IL18, CD86, TLR2, IL10RA, CD209 |
| 117 | HTR1A | 9.66E-20 | 1.28E-21 | 64 | 36 | POMC, KNG1, APP, GNAI2, ADCY2, SST, ADCY9, ADCY8, CXCR2, ADORA1, SSTR5, NPY, PENK, GAL, ADCY1, OPRM1, CHRM2, GNG2, DRD2, MCHR1, MTNR1A, ADRA2A, RGS4, PDYN, DRD3, NPY1R, DRD4, PNOC, HRH3, CHRM4, SSTR2, NMS, OPRD1, GPR55, ADRA2C, HTR1B |
| 118 | PDYN | 1.58E-19 | 2.13E-21 | 51 | 32 | POMC, KNG1, APP, GNAI2, ADCY2, CCR5, SST, ADCY8, ADORA1, ADCY6, SSTR5, NPY, PENK, CASR, GAL, OPRM1, CHRM2, ADCY5, DRD2, HTR1A, NMU, MCHR1, ADRA2A, RGS4, DRD3, DRD4, PNOC, HRH3, CHRM4, SSTR2, OPRD1, GPR55 |
| 119 | FASLG | 1.58E-19 | 2.13E-21 | 51 | 32 | JUN, AKT1, TNFRSF1A, CASP8, RIPK1, FADD, FOS, CD4, IL10, SIRT1, NFATC2, NFATC1, NFATC3, SRC, SYK, JAK2, GRB2, IL2, FYN, CASP3, IKBKG, IKBKB, BIRC2, CTLA4, IL12B, EGR2, EZR, FAS, IL18, CD247, EGR3, PTPN13 |
| 120 | CHRM3 | 1.93E-19 | 2.63E-21 | 65 | 36 | KNG1, GNA15, APP, PIK3CA, F2R, EDN1, PLCB2, GCG, PLCB3, GCGR, GNRH1, CASR, EDNRA, GAST, EDNRB, BDKRB2, PLCB1, MCHR1, TBXA2R, PLCB4, CCK, TRH, RGS2, GRP, NTS, ADRBK1, PTAFR, P2RY2, TAC1, GNRHR, LTB4R, HCRT, GRK5, TACR1, CCKAR, OXTR |
| 121 | HRH3 | 2.06E-19 | 2.85E-21 | 42 | 29 | POMC, KNG1, GNAI2, ADCY2, SST, ADCY8, ADORA1, NPY, PENK, CASR, GAL, ADCY4, OPRM1, CHRM2, DRD2, HTR1A, MCHR1, MTNR1A, ADRA2A, RGS4, PDYN, DRD3, NPY1R, DRD4, PNOC, NPY2R, CHRM4, HTR1B, HCAR2 |
| 122 | GSTM3 | 2.06E-19 | 2.85E-21 | 42 | 29 | GSTM1, CYP2E1, CYP1A2, CYP1A1, GSTA2, GSTA4, GSTA1, GSTA3, GSTP1, ALDH1A3, CYP2B6, CYP3A4, CYP3A43, CYP3A5, CYP2C19, CYP2C9, CYP2C18, CYP2C8, ADH1B, ADH1A, CYP1B1, GSTM2, GSTT2B, GSTO1, GSTZ1, GSTO2, GSS, GPX1, GGT1 |
| 123 | MET | 2.75E-19 | 3.83E-21 | 55 | 33 | MAPK1, MAPK3, TP53, UBC, VEGFA, EGFR, EGF, SRC, HRAS, CTNNB1, RB1, GRB2, RAC1, PTPN11, SOS1, PIK3CA, SHC1, CDC42, PIK3R1, PTPN1, PLCG1, CBL, SDC1, SH3GL2, CRK, CDH2, BCAR1, ITGA6, INPP5D, ITGB4, CTNNA1, CTNND1, RAB5A |
| 124 | GAST | 2.86E-19 | 4.02E-21 | 62 | 35 | INS-IGF2, FOS, CCND1, EGF, KNG1, GNA15, APP, PIK3CA, PIK3R1, F2R, EDN1, SST, GCG, PIK3R2, GCGR, GNRH1, CASR, EDNRA, EDNRB, BDKRB2, NMU, CCK, TRH, GRP, NTS, TF, PTAFR, P2RY2, TAC1, GNRHR, HCRT, TACR1, CCKAR, CHRM3, OXTR |
| 125 | FN1 | 4.53E-19 | 6.47E-21 | 107 | 46 | IL6, AKT1, YWHAZ, IL1B, VEGFA, EGFR, EGF, KNG1, FGF2, TGFBI, SRC, IFNG, SYK, IGF1, TGFB1, SMAD2, RAC1, IRS1, SDC2, SOS1, APP, ITGAV, ALB, ICAM1, PTPN1, EDN1, SDC4, SDC1, CD44, SERPINE1, BCAR1, GAPDH, ITGB1, DCN, CRP, ACTN2, ITGA6, GSN, PLG, PTK2, ALDOA, A2M, CLU, THBS1, ITGA2B, SERPING1 |
| 126 | ADCY8 | 4.53E-19 | 6.42E-21 | 70 | 37 | POMC, CREB1, PTH, KNG1, APP, GNAI2, PDE4A, CALM1, ADCY2, CRH, ADRB2, CALM2, SST, CALM3, CALCA, ADCY9, PKM, GCG, ADORA1, ADCY6, NPY, GNAS, GCGR, ADRB1, ADCY7, ADCY1, HTR7, ADCY4, ADCY5, HTR1A, HTR4, PDYN, NPY1R, NPY2R, HRH3, PRKACA, PDE8A |
| 127 | NFATC1 | 5.36E-19 | 7.78E-21 | 40 | 28 | PTGS2, MAPK3, JUN, FASLG, MAPK14, FOS, PPARG, CSF2, NFATC2, NFATC3, IFNG, GSK3B, IL2, IL2RA, IL8, IL4, YWHAQ, CREM, PRKCZ, EGR2, IL3, PPP3CA, EGR3, PPP3R1, PPP3CB, PPIA, KPNB1, PPP3CC |
| 128 | MTOR | 5.36E-19 | 7.73E-21 | 78 | 39 | INS-IGF2, PLD2, AKT1, YWHAZ, STAT3, VEGFA, BCL2, CCND1, EGFR, EGF, RELA, CTNNB1, HIF1A, NFKB1, LEP, GRB2, FOXO1, IL2, PRKCA, IRS1, YWHAG, SOS1, PIK3CA, PTEN, YWHAB, CDC42, PIK3R1, PPARGC1A, YWHAE, YWHAQ, YWHAH, SREBF1, IRS2, BCL2L1, PIK3CB, PIK3CG, PIK3CD, YY1, AKT3 |
| 129 | OXTR | 5.84E-19 | 8.54E-21 | 56 | 33 | KNG1, GNA15, APP, F2R, EDN1, GCG, PLCB3, GCGR, GNRH1, CASR, EDNRA, GAST, EDNRB, BDKRB2, NMU, TBXA2R, PLCB4, CCK, TRH, RGS2, GRP, NTS, ADRBK1, NMS, TAC1, NMUR2, GNRHR, LTB4R, HCRT, GRK5, TACR1, CCKAR, CHRM3 |
| 130 | F2RL1 | 7.96E-19 | 1.17E-20 | 53 | 32 | ANXA1, KNG1, F3, GNA15, PIK3CA, F2R, EDN1, LPAR2, GCG, PLCB3, GNRH1, CASR, EDNRA, GAST, EDNRB, BDKRB2, PLCB1, TBXA2R, CCK, TRH, GRP, NTS, ADRBK1, PTAFR, P2RY2, TAC1, GNRHR, LTB4R, GRK5, TACR1, CCKAR, CHRM3 |
| 131 | IL4 | 1.24E-18 | 1.86E-20 | 57 | 33 | IL6, JUN, IL1B, MAPK14, FOS, IL10, PPARG, CSF2, NFATC2, NFATC1, NFATC3, IFNG, JAK2, GRB2, IL2, NOS2, JAK1, NOTCH1, IRS1, IL2RA, PIK3CA, JAK3, PIK3R1, ICAM1, IL12B, IRS2, CBL, CCR5, VCAM1, IL3, PTPN6, INPP5D, CD209 |
| 132 | BCAR1 | 1.24E-18 | 1.86E-20 | 57 | 33 | ESR1, CSF1R, SRC, SYK, IGF1, JAK2, GRB2, RAC1, FYN, PRKCA, IRS1, LCK, PIK3CA, CDC42, PIK3R1, GNAI2, ITGAV, PTPN1, MET, YES1, CBL, FN1, CXCR4, CRK, HCK, PDGFRB, PTK2, PDGFB, GDNF, PTK2B, RET, VAV3, DCC |
| 133 | BDNF | 1.38E-18 | 2.09E-20 | 38 | 27 | IL6, GAD2, TP53, AKT1, FOS, VEGFA, NOS3, CREB1, FGF2, CTNNB1, IGF1, CDC42, RELN, MAPK9, CRH, CDH2, MMP3, CASP6, PLG, NGFR, CTNND1, MECP2, SHC2, TPM1, NTRK1, NTF3, NTRK2 |
| 134 | CYP2B6 | 1.47E-18 | 2.26E-20 | 88 | 41 | GSTM1, CYP2E1, UGT1A6, CYP1A2, CYP1A1, ADH7, GSTA2, GSTA4, GSTA1, GSTA3, GSTP1, ALDH3A1, ALDH1A3, CYP3A4, CYP3A43, CYP3A5, CYP2C19, CYP2C9, CYP2C18, CYP2C8, ADH1B, ADH4, ADH1A, GSTM2, GSTM3, GSTT2B, GSTO1, GSTZ1, GSTO2, ADH6, ALDH1A1, UGT1A1, ADH5, PLA2G1B, PTGS2, PTGS1, PLA2G2A, ALDH1A2, PLA2G4A, PLA2G6, ALOX12 |
| 135 | RGS4 | 1.47E-18 | 2.25E-20 | 41 | 28 | POMC, KNG1, GNAI2, CALM1, ERBB3, CCR5, SST, LPAR2, ADORA1, NPY, PENK, CASR, OPRM1, CHRM2, BDKRB2, GNG2, DRD2, HTR1A, ADRA2A, PDYN, DRD3, DRD4, PNOC, NPY2R, HRH3, CHRM4, SSTR2, ADRA2C |
| 136 | ADRBK1 | 1.60E-18 | 2.46E-20 | 76 | 38 | CAV1, KNG1, GNA15, PRKCA, APP, IL8, F2R, EDN1, PRKCD, ADRB2, CCR5, CXCR4, LPAR2, GCG, CXCL12, GCGR, GNRH1, CASR, EDNRA, CHRM2, EDNRB, BDKRB2, GNG2, TBXA2R, CCK, TRH, GRP, NTS, PTAFR, P2RY2, TAC1, GNRHR, LTB4R, HCRT, TACR1, CCKAR, CHRM3, OXTR |
| 137 | PTAFR | 2.14E-18 | 3.32E-20 | 65 | 35 | ANXA1, KNG1, GNA15, PIK3CA, ICAM1, F2R, EDN1, CD44, VCAM1, LPAR2, PLCB2, GCG, PLCB3, GCGR, GNRH1, CASR, EDNRA, GAST, EDNRB, BDKRB2, PLCB1, TBXA2R, PLCB4, CCK, TRH, GRP, ADRBK1, P2RY2, NCAM1, TAC1, GNRHR, LTB4R, GRK5, TACR1, CHRM3 |
| 138 | ICAM1 | 2.49E-18 | 3.90E-20 | 69 | 36 | IL6, JUN, INS-IGF2, AKT1, IL1B, STAT3, VEGFA, NOS3, IL10, CSF2, RELA, IL1A, F3, IFNG, TGFB1, NFKB1, RAC1, IL2, PRKCA, NFKBIA, IL4, RAC3, PRKCD, CCL2, CD44, FN1, VCAM1, EZR, IL18, CRP, B2M, CD86, ITGB2, PTAFR, HLA-DRB1, NCAM1 |
| 139 | SP1 | 2.80E-18 | 4.41E-20 | 81 | 39 | MAPK1, MAPK3, CREBBP, ESR1, RXRA, PPARA, NCOA2, TP53, JUN, UBC, MYC, EP300, STAT3, CEBPB, VEGFA, E2F1, PPARG, ABCB1, RELA, APC, ARNT, SMARCA4, CARM1, SMAD3, HIF1A, RB1, AR, SMAD4, NFKB1, SMAD2, CDK2, HDAC1, CEBPD, DNMT1, SREBF1, SREBF2, HDAC2, YY1, MMP14 |
| 140 | MCHR1 | 2.81E-18 | 4.45E-20 | 77 | 38 | POMC, GNA15, LEP, ADCY2, SST, PLCB2, GCG, ADORA1, SSTR5, NPY, GNRH1, CASR, GAL, CHRM2, DRD2, HTR1A, NMU, TBXA2R, CCK, MTNR1A, TRH, PDYN, DRD3, NPY1R, PNOC, GRP, NPY2R, NTS, HRH3, SSTR2, OPRD1, TAC1, GNRHR, HCAR2, HCRT, TACR1, CCKAR, CHRM3 |
| 141 | FGR | 3.61E-18 | 5.77E-20 | 55 | 32 | PLA2G4A, FASLG, MAPK14, STAT3, VEGFA, MAPK11, EGFR, EGF, SRC, SYK, FYN, LCK, IL8, GNAI2, PRKCD, PLCG1, YES1, SNCA, CBL, HCK, BCAR1, ABL1, RASA1, PDGFRB, PLCG2, PTK2, PDGFB, PTK2B, VAV2, PTPN13, EPHB2, WAS |
| 142 | ADRA1D | 3.83E-18 | 6.17E-20 | 42 | 28 | ANXA1, KNG1, GNA15, PIK3CA, EDN1, GCG, PLCB3, GCGR, GNRH1, CASR, EDNRA, GAST, EDNRB, PLCB1, GNG2, TBXA2R, CCK, TRH, RGS2, GRP, NTS, ADRBK1, P2RY2, TAC1, HCRT, TACR1, CCKAR, CHRM3 |
| 143 | CYP2E1 | 4.54E-18 | 7.35E-20 | 70 | 36 | GSTM1, UGT1A6, ADH7, GSTA2, GSTA4, GSTA1, GSTA3, GSTP1, ALDH3A1, CYP2B6, CYP3A4, CYP3A43, CYP3A5, CYP2C19, CYP2C9, CYP2C18, CYP2C8, ADH1B, ADH4, ADH1A, GSTM2, GSTM3, GSTT2B, GSTO1, GSTZ1, GSTO2, ADH6, UGT1A1, ADH5, PLA2G1B, ALDH3A2, ALDH2, PTGS2, PTGS1, PLA2G2A, AKR1B1 |
| 144 | CHRM2 | 5.02E-18 | 8.19E-20 | 59 | 33 | POMC, KNG1, GNA15, ADCY2, CCR5, SST, CXCR2, ADORA1, ADCY6, SSTR5, NPY, CASR, GAL, OPRM1, BDKRB2, GNG2, DRD2, HTR1A, MCHR1, ADRA2A, RGS4, PDYN, DRD3, NPY1R, DRD4, PNOC, NPY2R, ADRBK1, HRH3, CHRM4, OPRD1, ADRA2C, HTR1B |
| 145 | GSTA5 | 5.81E-18 | 9.54E-20 | 31 | 24 | GSTM1, CYP2E1, CYP1A2, CYP1A1, GSTA2, GSTA4, GSTA1, GSTA3, GSTP1, ALDH3A1, CYP2B6, CYP3A4, CYP3A43, CYP3A5, CYP2C19, CYP2C18, ADH1B, ADH1A, CYP1B1, GSTM2, GSTM3, GSTO1, GSTZ1, GSS |
| 146 | HDAC3 | 6.44E-18 | 1.07E-19 | 91 | 41 | CREBBP, RXRA, PPARA, NCOA2, TNFRSF1A, PAFAH1B1, MYC, EP300, STAT3, CEBPB, CCND1, SIN3A, E2F1, PPARG, BIRC5, RELA, CARM1, HIF1A, RB1, TGFB1, NFKB1, HDAC1, NFKBIA, RORA, PPARGC1A, CALM1, LPL, CLOCK, NPAS2, RBBP4, CALM2, CALM3, HDAC4, HDAC2, CSNK2A1, RBBP7, ARNTL, CHD4, YY1, HDAC6, GATAD2A |
| 147 | TAC1 | 6.44E-18 | 1.07E-19 | 91 | 41 | FOS, KNG1, GNA15, NGF, APP, PIK3CA, F2R, EDN1, SST, CALCA, GCG, PLCB3, GCGR, GNRH1, CASR, EDNRA, GAST, EDNRB, BDKRB2, PLCB1, NMU, MCHR1, TBXA2R, CCK, TRH, RGS2, GRP, NTS, ADRBK1, PTAFR, NMS, P2RY2, NMUR2, GNRHR, LTB4R, HCRT, GRK5, TACR1, CCKAR, CHRM3, OXTR |
| 148 | ABL1 | 7.39E-18 | 1.24E-19 | 67 | 35 | TP53, JUN, MYC, MAPK14, HSP90AA1, SRC, CTNNB1, RB1, GRB2, CDK2, RAC1, FYN, NFKBIA, MDM2, SOS1, LCK, ATM, SHC1, CDKN1B, CDC42, PIK3R1, PLCG1, YES1, CBL, CRK, CDH2, HCK, PDGFRB, CTNNA1, PDGFB, CTNND1, EPHB2, CDK5, NTRK1, SLIT2 |
| 149 | PRKCE | 8.26E-18 | 1.40E-19 | 34 | 25 | YWHAZ, SRC, HRAS, LCK, IKBKG, RAF1, CDC42, IKBKB, ICAM1, IRS2, ADCY2, PRKCD, PLCG1, CRK, VCAM1, ADCY9, ADCY8, BCAR1, ADCY6, GNB2L1, ADCY4, ADCY5, CABIN1, PTK2B, TIAM1 |
| 150 | OPRK1 | 8.26E-18 | 1.40E-19 | 34 | 25 | POMC, KNG1, APP, GNAI2, ADCY2, SST, ADORA1, NPY, PENK, GAL, OPRM1, CHRM2, DRD2, HTR1A, NMU, ADRA2A, RGS4, PDYN, DRD3, NPY1R, DRD4, PNOC, NPY2R, OPRD1, ADRA2C |
| 151 | PTK2 | 1.44E-17 | 2.48E-19 | 50 | 30 | TP53, NRAS, EGFR, ERBB2, SRC, HRAS, GRB2, RAC1, FYN, IRS1, PTPN11, SOS1, PIK3CA, PTEN, SHC1, CDC42, PIK3R1, ITGAV, FN1, CRK, BCAR1, ITGB1, PIK3R2, PDGFRB, ITGB4, ITGB2, NCAM1, ITGA2B, DCC, PTPRA |
| 152 | CASP8 | 1.44E-17 | 2.48E-19 | 50 | 30 | MAPK1, MAPK3, TP53, TNFRSF1A, TRADD, RIPK1, FADD, TRAF1, FASLG, TRAF2, MAP2K7, BCL2, RELA, SRC, STAT1, NFKB1, VIM, APP, CASP3, IKBKG, IKBKB, BIRC2, ITGAV, BCL2L1, MAVS, EZR, FAS, XIAP, CASP6, PLEC |
| 153 | ADH7 | 2.02E-17 | 3.50E-19 | 32 | 24 | GSTM1, CYP2E1, UGT1A6, GSTT1, GSTA2, GSTA4, GSTA1, GSTA3, GSTP1, ALDH3A1, ALDH1A3, CYP2B6, GSTM2, GSTT2B, ALDH1A1, COMT, UGT1A1, ALDH3A2, ALDH2, ALDH1A2, MAOA, ALDH9A1, ALDH7A1, ALDH1B1 |
| 154 | ADRA2A | 2.20E-17 | 3.84E-19 | 54 | 31 | POMC, KNG1, GNAI2, ADCY2, CCR5, SST, ADORA1, NPY, ADCY7, GAL, ADCY1, ADCY4, OPRM1, CHRM2, BDKRB2, ADCY5, DRD2, HTR1A, RGS4, PDYN, DRD3, NPY1R, DRD4, PNOC, NPY2R, HRH3, CHRM4, OPRD1, ADRA2C, HTR1B, HCAR2 |
| 155 | PIK3CD | 2.24E-17 | 3.94E-19 | 65 | 34 | AKT1, VEGFA, NOS3, EGFR, ERBB2, SRC, HRAS, SYK, IGF1, JAK2, RAC1, IRS1, SOS1, PTEN, PIK3R1, VAV1, RAC3, IRS2, PLCG1, MTOR, CBL, RAC2, PLCB2, PLCB3, PIK3R2, INPP5D, PLCG2, PLCB1, ERBB4, PLCB4, VAV2, PLCE1, PLCD3, AKT3 |
| 156 | ARRB2 | 2.29E-17 | 4.08E-19 | 44 | 28 | MAPK1, MAPK3, UBC, UBA52, MAPK14, SRC, GNA15, RAC1, FYN, UBB, NOTCH1, NFKBIA, MDM2, IL8, F2R, ADRB2, CXCR4, LHCGR, HCK, CXCR2, CXCL12, ADRB1, DVL2, RAB5A, TBXA2R, GLI2, WNT5A, SLC9A3R1 |
| 157 | MAPK9 | 2.29E-17 | 4.08E-19 | 44 | 28 | TP53, JUN, MAP2K7, FOS, NFATC2, NFATC3, IFNG, CTNNB1, GRB2, RAC1, IL2, JAK1, IRS1, IL2RA, NGF, LCK, APP, SHC1, BDNF, CDC42, JAK3, BAD, MAPK8IP1, PRKCZ, TRAF3, IL18, NGFR, WNT5A |
| 158 | GRP | 2.35E-17 | 4.22E-19 | 81 | 38 | FOS, ANXA1, KNG1, GNA15, APP, PIK3CA, F2R, EDN1, LPAR2, GCG, PLCB3, GCGR, GNRH1, CASR, EDNRA, GAST, EDNRB, BDKRB2, NMU, MCHR1, TBXA2R, CCK, TRH, RGS2, NTS, ADRBK1, PTAFR, NMS, P2RY2, TAC1, NMUR2, GNRHR, HCRT, GRK5, TACR1, CCKAR, CHRM3, OXTR |
| 159 | GSTO1 | 2.60E-17 | 4.69E-19 | 41 | 27 | GSTM1, CYP2E1, CYP1A2, CYP1A1, GSTA2, GSTA4, GSTA1, GSTA3, GSTP1, ALDH3A1, CYP2B6, CYP3A4, CYP3A43, CYP3A5, CYP2C19, GGT7, ADH1B, ADH1A, CYP1B1, GSTM2, GSTM3, GSTT2B, GSTZ1, GSTO2, GSS, GPX1, AS3MT |
| 160 | PENK | 2.68E-17 | 4.89E-19 | 38 | 26 | POMC, JUN, FOS, KNG1, APP, GNAI2, ADCY2, SST, ADORA1, NPY, GAL, OPRM1, DRD2, HTR1A, NMU, RGS4, PDYN, DRD3, NPY1R, DRD4, PNOC, NPY2R, HRH3, OPRD1, ADRA2C, HTR1B |
| 161 | GSTM4 | 2.68E-17 | 4.89E-19 | 38 | 26 | GSTM1, CYP2E1, CYP1A2, CYP1A1, ADH7, GSTA2, GSTA4, GSTA1, GSTA3, GSTP1, ALDH1A3, CYP2B6, CYP3A4, CYP3A43, CYP3A5, CYP2C19, GGT7, CYP1B1, GSTM2, GSTM3, GSTT2B, GSTO1, GSTZ1, GSTO2, GSS, GPX1 |
| 162 | GSTZ1 | 5.38E-17 | 9.87E-19 | 45 | 28 | GSTM1, CYP2E1, CYP1A2, CYP1A1, GSTA2, GSTA4, GSTA1, GSTA3, GSTP1, CYP2B6, CYP3A4, CYP3A43, CYP3A5, CYP2C19, CYP2C9, CYP2C18, CYP2C8, ADH1B, ADH1A, CYP1B1, GSTM2, GSTM3, GSTT2B, GSTO1, GSTO2, GSS, GPX1, ADH5 |
| 163 | SOCS3 | 5.68E-17 | 1.05E-18 | 59 | 32 | IL6, INS-IGF2, MAPK14, STAT3, MAPK11, NRAS, SRC, HRAS, IFNG, IL23A, STAT1, IGF1, JAK2, LEP, IL2, JAK1, IRS1, PTPN11, IL2RA, LCK, JAK3, INSR, IL12B, IRS2, TNF, IL6R, IL6ST, PTPN6, EPOR, RASA1, PRLR, LEPR |
| 164 | GNRHR | 6.72E-17 | 1.25E-18 | 63 | 33 | ANXA1, KNG1, GNA15, F2R, PLCB2, GCG, GCGR, GNRH1, CASR, EDNRA, GAST, EDNRB, BDKRB2, NMU, MCHR1, TBXA2R, CCK, TRH, RGS2, GRP, NTS, ADRBK1, PTAFR, NMS, P2RY2, TAC1, LTB4R, HCRT, GRK5, TACR1, CCKAR, CHRM3, OXTR |
| 165 | CHRM4 | 7.21E-17 | 1.35E-18 | 36 | 25 | POMC, KNG1, ADCY2, SST, ADORA1, SSTR5, NPY, ADCY7, OPRM1, CHRM2, BDKRB2, DRD2, HTR1A, ADRA2A, RGS4, PDYN, DRD3, NPY1R, DRD4, NPY2R, HRH3, SSTR2, OPRD1, ADRA2C, HCAR2 |
| 166 | PTPN6 | 7.53E-17 | 1.42E-18 | 75 | 36 | CD4, KIT, EGFR, EGF, IFNG, CTNNB1, SYK, JAK2, GRB2, FYN, JAK1, PTPN11, LCK, PIK3CA, IL4, JAK3, PIK3R1, INSR, VAV1, SOCS3, YES1, CXCR4, IL6ST, EPOR, B2M, CXCL12, CD247, INPP5D, PLCG2, CTNND1, CSF2RB, SSTR2, HLA-DRB1, SIRPA, NTRK1, LIFR |
| 167 | GNRH1 | 9.27E-17 | 1.76E-18 | 88 | 39 | INS-IGF2, FOS, ANXA1, KNG1, IGF1, GNA15, APP, PIK3CA, F2R, EDN1, PRL, GCG, NPY, GCGR, CASR, EDNRA, GAST, EDNRB, NMU, MCHR1, CCK, TRH, RGS2, GRP, NTS, ADRBK1, PTAFR, NMS, P2RY2, TAC1, NMUR2, GNRHR, LTB4R, HCRT, GRK5, TACR1, CCKAR, CHRM3, OXTR |
| 168 | GNAO1 | 1.55E-16 | 2.97E-18 | 31 | 23 | MAPK1, MAPK3, ESR1, AKT1, MAPK14, SRC, HRAS, GNA15, RAC1, CDC42, GNAI2, EDN1, ADCY2, CCR5, CXCR4, ADCY9, ADCY8, PLCB2, CXCL12, ADCY4, OPRM1, PLCB1, GNG2 |
| 169 | KRAS | 1.55E-16 | 2.97E-18 | 31 | 23 | TP53, NRAS, KIT, EGFR, EGF, HBEGF, HRAS, MAP2K1, GRB2, FYN, IRS1, PTPN11, SOS1, LCK, YWHAB, SHC1, RAF1, IRS2, CALM1, YES1, PTK2, SHC2, NCAM1 |
| 170 | ANXA1 | 1.70E-16 | 3.31E-18 | 57 | 31 | PLA2G1B, POMC, KNG1, GNA15, APP, PIK3CA, GNAI2, F2R, EDN1, ADCY2, CCR5, SST, ADCY9, CXCR2, PLCB2, NPY, GCGR, GNRH1, CASR, EDNRA, EDNRB, PTGER3, CCK, MTNR1A, TRH, RGS2, GRP, PTAFR, P2RY2, GNRHR, LTB4R |
| 171 | SERPINE1 | 1.70E-16 | 3.31E-18 | 57 | 31 | IL6, TP53, INS-IGF2, VEGFA, PPARG, EGF, KNG1, ARNT, HIF1A, SMAD4, TGFB1, HDAC1, APP, ITGAV, ALB, MAX, VLDLR, NR3C1, CLOCK, NPAS2, FN1, ARNTL, EPAS1, CRP, ACE, PLG, ALDOA, A2M, CLU, PLAT, SERPING1 |
| 172 | TNFRSF1A | 1.70E-16 | 3.31E-18 | 57 | 31 | IL6, CASP8, TRADD, RIPK1, FADD, TRAF1, FASLG, TRAF2, MAP2K7, TXN, PPARG, CAV1, RELA, SYK, STAT1, JAK2, NFKB1, MAP3K7, JAK1, NFKBIA, HDAC3, CASP3, IKBKG, IKBKB, BIRC2, TNF, TNFRSF1B, PRKCZ, TRAF3, IL1R1, GNB2L1 |
| 173 | GSTT2B | 1.79E-16 | 3.51E-18 | 40 | 26 | GSTM1, CYP2E1, CYP1A2, CYP1A1, ADH7, GSTA2, GSTA4, GSTA1, GSTA3, GSTP1, CYP2B6, CYP3A4, CYP3A43, CYP3A5, CYP2C19, CYP2C9, ADH1B, ADH1A, CYP1B1, GSTM2, GSTM3, GSTO1, GSTZ1, GSTO2, GSS, GPX1 |
| 174 | INPP5D | 1.87E-16 | 3.71E-18 | 50 | 29 | UBC, INS-IGF2, CSF2, HRAS, JAK2, GRB2, FYN, JAK1, IRS1, PTPN11, LCK, PIK3CA, PTEN, IL4, SHC1, JAK3, PIK3R1, INSR, IRS2, MET, PIK3CB, PIK3CG, PIK3CD, IL3, PTPN6, PIK3R2, RASA1, CSF2RB, CSF2RA |
| 175 | PNOC | 1.87E-16 | 3.71E-18 | 50 | 29 | POMC, KNG1, ADCY2, CCR5, SST, ADORA1, NPY, PENK, CASR, GAL, OPRM1, CHRM2, BDKRB2, DRD2, HTR1A, NMU, MCHR1, ADRA2A, RGS4, PDYN, DRD3, NPY1R, NPY2R, HRH3, SSTR2, OPRD1, GPR55, ADRA2C, NMUR2 |
| 176 | GALR3 | 1.90E-16 | 3.81E-18 | 37 | 25 | POMC, KNG1, GNAI2, ADCY2, SST, ADCY9, NPY, ADCY7, GAL, ADCY1, ADCY4, OPRM1, CHRM2, ADCY5, DRD2, HTR1A, NMU, ADRA2A, PDYN, NPY1R, DRD4, NPY2R, HRH3, CHRM4, NMS |
| 177 | GSTO2 | 1.90E-16 | 3.81E-18 | 37 | 25 | GSTM1, CYP2E1, CYP1A2, CYP1A1, GSTA2, GSTA4, GSTA1, GSTA3, GSTP1, ALDH3A1, CYP2B6, CYP3A4, CYP3A43, CYP3A5, CYP2C19, ADH1B, ADH1A, CYP1B1, GSTM2, GSTM3, GSTT2B, GSTO1, GSTZ1, GSS, GPX1 |
| 178 | ADRB2 | 1.95E-16 | 3.96E-18 | 61 | 32 | POMC, UBC, IL1B, PTH, SRC, ADCY2, CRH, LHCGR, CALCA, ADCY9, ADCY8, GCG, ADM, ADCY6, ADORA2A, GNAS, GCGR, DRD5, ADRB1, ADCY7, AVPR2, ADCY1, ADCY4, ADCY5, MC4R, GNG2, VIPR2, HTR4, ADRB3, SLC9A3R1, ADRBK1, GRK5 |
| 179 | TRHR | 1.95E-16 | 3.96E-18 | 61 | 32 | KNG1, GNA15, F2R, EDN1, GCG, GCGR, GNRH1, CASR, EDNRA, GAST, EDNRB, BDKRB2, NMU, MCHR1, TBXA2R, CCK, TRH, GRP, NTS, ADRBK1, PTAFR, P2RY2, TAC1, NMUR2, GNRHR, LTB4R, HCRT, GRK5, TACR1, CCKAR, CHRM3, OXTR |
| 180 | RXRA | 2.77E-16 | 5.65E-18 | 104 | 42 | CYP1A1, MAPK1, MAPK3, CREBBP, ESR1, PPARA, NCOA2, AKT1, EP300, CEBPB, BCL2, PPARG, RELA, SRC, CARM1, ABCA1, SP1, HIF1A, TGFB1, NFKB1, LEP, HDAC1, CEBPD, ESR2, PRKCA, HDAC3, RORA, PPARGC1A, APOA1, SREBF1, LPL, SREBF2, NR4A2, NR2F1, CLOCK, NPAS2, YAP1, FADS1, APOA2, ARNTL, KLF5, ALAS1 |
| 181 | CALCA | 3.64E-16 | 7.50E-18 | 62 | 32 | POMC, PTH, TGFBI, APP, APOA1, ADCY2, NPPA, CRH, PRL, ADRB2, LHCGR, ADCY9, ADCY8, GCG, ADM, IAPP, ADCY6, TTR, B2M, ADORA2A, GNAS, GCGR, ADCY7, GSN, AVPR2, ADCY1, ADCY4, ADCY5, MC4R, VIPR2, HTR4, TAC1 |
| 182 | BTK | 3.64E-16 | 7.50E-18 | 62 | 32 | JUN, FADD, FASLG, FOS, SRC, HRAS, SYK, JAK2, RAC1, FYN, SOS1, LCK, PIK3CA, PIK3R1, VAV1, MYD88, PLCG1, YES1, CBL, PIK3CB, PIK3CG, PIK3CD, HCK, PTPN6, EPOR, PIK3R2, INPP5D, PLCG2, TYROBP, VAV2, VAV3, WAS |
| 183 | IL3 | 3.99E-16 | 8.27E-18 | 24 | 20 | JUN, AKT1, FOS, CSF1R, CSF2, NFATC2, NFATC1, NFATC3, IFNG, JAK2, GRB2, IL2, PTPN11, IL2RA, PIK3CA, IL4, SHC1, PIK3R1, INPP5D, CSF2RB |
| 184 | PYY | 4.22E-16 | 8.79E-18 | 41 | 26 | POMC, KNG1, APP, GNAI2, ADCY2, SST, ADORA1, SSTR5, NPY, PENK, GAL, OPRM1, DRD2, HTR1A, NMU, MCHR1, CCK, ADRA2A, PDYN, NPY1R, DRD4, PNOC, NPY2R, HRH3, SSTR2, ADRA2C |
| 185 | OPRD1 | 4.86E-16 | 1.02E-17 | 38 | 25 | POMC, APP, GNAI2, ADCY2, SST, NPY, PENK, CASR, GAL, OPRM1, CHRM2, DRD2, HTR1A, NMU, MCHR1, ADRA2A, PDYN, DRD3, NPY1R, DRD4, PNOC, CHRM4, NMS, ADRA2C, HTR1B |
| 186 | EPHX1 | 5.05E-16 | 1.06E-17 | 35 | 24 | GSTM1, CYP2E1, CYP1A2, CYP1A1, GSTA2, GSTA4, GSTA1, GSTA3, GSTP1, CYP2B6, CYP3A4, CYP3A43, CYP3A5, CYP2C19, CYP2C9, CYP2C18, CYP2C8, CYP1B1, GSTM2, GSTM3, GSTT2B, GSTO1, GSTZ1, GSTO2 |
| 187 | NTS | 6.70E-16 | 1.43E-17 | 71 | 34 | JUN, FOS, KNG1, GNA15, APP, F2R, EDN1, SST, LPAR2, GCG, GCGR, GNRH1, CASR, EDNRA, GAST, EDNRB, BDKRB2, NMU, MCHR1, TBXA2R, CCK, TRH, GRP, ADRBK1, NMS, TAC1, NMUR2, GNRHR, LTB4R, HCRT, TACR1, CCKAR, CHRM3, OXTR |
| 188 | TACR1 | 6.70E-16 | 1.43E-17 | 71 | 34 | KNG1, GNA15, APP, F2R, EDN1, GCG, PLCB3, GCGR, GNRH1, CASR, EDNRA, GAST, EDNRB, BDKRB2, NMU, MCHR1, TBXA2R, CCK, TRH, RGS2, GRP, NTS, ADRBK1, PTAFR, P2RY2, TAC1, NMUR2, GNRHR, LTB4R, HCRT, GRK5, CCKAR, CHRM3, OXTR |
| 189 | DRD4 | 7.48E-16 | 1.60E-17 | 45 | 27 | POMC, KNG1, ADCY2, CCR5, SST, ADORA1, SSTR5, NPY, PENK, GAL, ADCY1, OPRM1, CHRM2, GNG2, DRD2, HTR1A, ADRA2A, RGS4, PDYN, DRD3, NPY1R, NPY2R, HRH3, CHRM4, OPRD1, ADRA2C, HTR1B |
| 190 | GALR1 | 1.16E-15 | 2.50E-17 | 30 | 22 | POMC, KNG1, GNAI2, ADCY2, SST, ADCY9, NPY, GAL, OPRM1, ADCY5, HTR1A, NMU, MCHR1, MTNR1A, PDYN, DRD3, NPY1R, PNOC, NPY2R, HRH3, SSTR2, NMS |
| 191 | TYK2 | 1.18E-15 | 2.55E-17 | 64 | 32 | IL6, IL1B, FASLG, FOS, STAT3, CD4, RELA, IFNG, IL23A, STAT1, JAK2, NFKB1, IL2, JAK1, NFKBIA, PTPN11, LCK, PIK3CA, IL4, PIK3R1, IL12B, PTPN1, SOCS3, CBL, CCL2, CCR5, IL6R, IL6ST, PTPN6, IL1R1, GNB2L1, PTAFR |
| 192 | PMCH | 1.19E-15 | 2.60E-17 | 39 | 25 | POMC, KNG1, GNA15, APP, PIK3CA, EDN1, ADCY2, CCR5, SST, GCG, NPY, PENK, GNRH1, GAL, MCHR1, CCK, TRH, PDYN, PNOC, NTS, TAC1, NMUR2, HCRT, GRK5, CHRM3 |
| 193 | GSTM5 | 1.34E-15 | 2.92E-17 | 36 | 24 | GSTM1, CYP2E1, CYP1A1, GSTA2, GSTA4, GSTA1, GSTA3, GSTP1, ALDH1A3, CYP2B6, CYP3A4, CYP3A43, CYP3A5, CYP2C19, GGT7, CYP1B1, GSTM2, GSTM3, GSTT2B, GSTO1, GSTZ1, GSTO2, GSS, GPX1 |
| 194 | PRL | 1.50E-15 | 3.29E-17 | 53 | 29 | ESR1, IL6, INS-IGF2, STAT3, EGF, TGFBI, HBEGF, SRC, STAT1, IGF1, JAK2, LEP, IL2, IRS1, PTPN11, APP, APOA1, IRS2, NPPA, SKP1, CALCA, IAPP, HSPG2, TTR, B2M, GNRH1, GSN, TRH, PRLR |
| 195 | CCKBR | 1.61E-15 | 3.55E-17 | 46 | 27 | KNG1, GNA15, F2R, EDN1, GCG, GCGR, GNRH1, CASR, EDNRA, GAST, EDNRB, BDKRB2, TBXA2R, CCK, TRH, RGS2, GRP, NTS, PTAFR, P2RY2, TAC1, GNRHR, HCRT, TACR1, CCKAR, CHRM3, OXTR |
| 196 | AR | 1.79E-15 | 3.98E-17 | 57 | 30 | MAPK1, CREBBP, NCOA2, IL6, TP53, JUN, UBC, AKT1, STUB1, EP300, BRCA1, STAT3, CCND1, EGFR, CAV1, HSP90AA1, SRC, SMARCA4, CTNNB1, SMAD3, SP1, RB1, HDAC1, FOXO1, MDM2, GAPDH, PRDX1, GNB2L1, PRKACA, PARK7 |
| 197 | SDC2 | 2.06E-15 | 4.60E-17 | 65 | 32 | MAPK1, MAPK3, JUN, FOS, STAT3, GPC1, CSF2, KNG1, FGF2, MMP2, SRC, HRAS, STAT1, TGFB1, GRB2, PTPN11, CASP3, IL8, SDC4, SDC1, FN1, EZR, ITGB1, DCN, HSPG2, RASA1, GNB2L1, SDC3, NCAN, CSF2RA, EPHB2, GPC6 |
| 198 | HTR1D | 2.79E-15 | 6.25E-17 | 28 | 21 | POMC, GNAI2, ADCY2, ADCY9, ADORA1, SSTR5, NPY, GAL, CHRM2, DRD2, HTR1A, ADRA2A, PDYN, DRD3, NPY1R, DRD4, CHRM4, NMS, OPRD1, ADRA2C, HTR1B |
| 199 | PTK2B | 2.81E-15 | 6.34E-17 | 40 | 25 | VEGFA, SRC, SYK, GSK3B, GRB2, IL2, FYN, PRKCA, JAK1, PTPN11, IL2RA, LCK, JAK3, VAV1, PRKCD, YES1, CBL, CRK, VCAM1, HCK, BCAR1, ITGB1, RASA1, GSN, ITGB2 |
| 200 | HDAC1 | 2.84E-15 | 6.43E-17 | 156 | 51 | CREBBP, ESR1, RXRA, TP53, UBC, MYC, EP300, STAT3, CCND1, SIN3A, LEF1, E2F1, TRIM28, CREB1, RELA, SMARCA4, CTNNB1, SP1, HIF1A, RB1, AR, SMAD4, NFKB1, SMAD2, NOTCH1, NFKBIA, HDAC3, PCNA, MDM2, DNMT1, DNMT3B, MAX, TCF7L2, KAT2B, NR3C1, RBBP4, CTBP1, SERPINE1, HDAC2, CSNK2A1, RBBP7, CHD4, YY1, RAN, PPP1CC, TAL1, MECP2, HDAC6, PHB, EZH2, GATAD2A |
| 201 | ADCY5 | 3.31E-15 | 7.55E-17 | 58 | 30 | POMC, PTH, APP, GNAI2, PDE4A, EDN1, ADCY2, CRH, ADRB2, CALCA, ADCY9, ADCY8, GCG, ADCY6, ADORA2A, GNAS, GCGR, ADRB1, ADCY7, CASR, ADCY1, HTR7, ADCY4, MC4R, DRD2, ADRA2A, PDYN, DRD3, PRKACA, PDE8A |
| 202 | AVPR1A | 3.34E-15 | 7.64E-17 | 47 | 27 | KNG1, EDN1, GCG, GCGR, GNRH1, EDNRA, EDNRB, BDKRB2, PLCB1, MCHR1, TBXA2R, CCK, TRH, RGS2, GRP, ADRBK1, PTAFR, P2RY2, TAC1, GNRHR, LTB4R, HCRT, GRK5, TACR1, CCKAR, CHRM3, OXTR |
| 203 | ADH5 | 3.38E-15 | 7.78E-17 | 23 | 19 | CYP2E1, GSTA2, GSTA4, GSTA1, GSTA3, GSTP1, ALDH3A1, ALDH1A3, CYP2B6, GSTM2, GSTZ1, ALDH1A1, COMT, ALDH3A2, ALDH2, ALDH1A2, ALDH9A1, ALDH7A1, ALDH1B1 |
| 204 | RET | 3.53E-15 | 8.28E-17 | 34 | 23 | STAT3, VEGFA, CREB1, SRC, HRAS, CTNNB1, GRB2, PRKCA, IRS1, PTPN11, SOS1, PIK3CA, SHC1, PIK3R1, PLCG1, CBL, CRK, BCAR1, RASA1, GRB10, CTNNA1, GDNF, CTNND1 |
| 205 | PTH | 3.53E-15 | 8.18E-17 | 66 | 32 | PTGS2, POMC, IL6, FOS, CCND1, IGF1, GJA1, ADCY2, CRH, ADRB2, LHCGR, CALCA, ADCY9, ADCY8, GCG, ADM, IAPP, ADCY6, ADORA2A, GNAS, GCGR, ADRB1, ADCY7, CASR, AVPR2, ADCY1, ADCY4, ADCY5, MC4R, VIPR2, HTR4, ADRB3 |
| 206 | CSK | 3.53E-15 | 8.28E-17 | 34 | 23 | AKT1, CD4, EGFR, EGF, CAV1, SRC, FYN, PTPN11, LCK, SHC1, PTPN1, YES1, CBL, FN1, HCK, PTPN6, RASA1, CD247, PTK2, HNRNPK, CTTN, ITGA2B, PTPRC |
| 207 | CD40LG | 3.53E-15 | 8.28E-17 | 34 | 23 | JUN, AKT1, TRAF1, MAPK14, FOS, CD4, NFATC2, NFATC1, NFATC3, RELA, F3, NFKB1, IL2, NFKBIA, BIRC2, JAK3, ICAM1, MAPK9, TRAF3, VCAM1, CD86, ITGB2, IGHG1 |
| 208 | GRK5 | 4.64E-15 | 1.09E-16 | 44 | 26 | KNG1, GNA15, CALM1, F2R, EDN1, SNCA, ADRB2, GCG, GCGR, GNRH1, CASR, EDNRA, EDNRB, BDKRB2, TBXA2R, CCK, TRH, GRP, PTAFR, P2RY2, TAC1, GNRHR, LTB4R, TACR1, CHRM3, OXTR |
| 209 | YWHAE | 5.91E-15 | 1.41E-16 | 59 | 30 | MAPK1, MAPK3, UBC, AKT1, YWHAZ, PAFAH1B1, NRAS, HRAS, CTNNB1, MAP2K1, FOXO1, YWHAG, NGF, CASP3, YWHAB, RAF1, CDKN1B, YWHAQ, YWHAH, BAD, KSR1, MTOR, PLK1, HDAC4, PPP2R1A, RAN, CSNK1D, NDE1, PCNT, NDEL1 |
| 210 | YWHAB | 5.91E-15 | 1.41E-16 | 59 | 30 | MAPK1, MAPK3, AKT1, YWHAZ, NRAS, HRAS, CTNNB1, MAP2K1, FOXO1, PRKCA, YWHAG, NGF, RAF1, CDKN1B, YWHAE, YWHAQ, YWHAH, BAD, KSR1, MTOR, CBL, HDAC4, YAP1, SPTAN1, ITGA6, ITGB4, PPP2R1A, RAN, CSNK1E, PPP3CC |
| 211 | MAOB | 5.99E-15 | 1.44E-16 | 21 | 18 | CYP3A4, CYP3A43, CYP3A5, CYP2C19, CYP2C9, CYP2C8, ADH1B, ADH1A, COMT, CYP2D6, ALDH3A2, ALDH2, ALDH9A1, ALDH1B1, AOX1, DDC, DBH, AANAT |
| 212 | TBXA2R | 5.99E-15 | 1.43E-16 | 67 | 32 | KNG1, SYK, GNA15, RAC1, PIK3CA, F2R, EDN1, GCG, PLCB3, GCGR, CASR, EDNRA, EDNRB, BDKRB2, NMU, MCHR1, CCK, RGS2, SLC9A3R1, GRP, NTS, ADRBK1, PTAFR, P2RY2, TAC1, GNRHR, LTB4R, GRK5, TACR1, CCKAR, CHRM3, OXTR |
| 213 | GHSR | 6.16E-15 | 1.49E-16 | 41 | 25 | KNG1, IGF1, LEP, PIK3CA, EDN1, GCG, GCGR, GNRH1, GAST, NMU, MCHR1, CCK, TRH, GRP, NTS, NMS, TAC1, NMUR2, GNRHR, HCRT, TACR1, GHRL, CCKAR, CHRM3, OXTR |
| 214 | NMU | 6.99E-15 | 1.70E-16 | 80 | 35 | POMC, KNG1, GNA15, F2R, ADCY2, SST, GCG, NPY, PENK, GCGR, GNRH1, EDNRA, GAL, GAST, OPRM1, EDNRB, MCHR1, TBXA2R, CCK, MTNR1A, TRH, PDYN, NPY1R, PNOC, GRP, NTS, NMS, OPRD1, ADRA2C, TAC1, NMUR2, GNRHR, HCRT, TACR1, OXTR |
| 215 | FRS2 | 8.46E-15 | 2.08E-16 | 29 | 21 | MAPK1, MAPK3, UBA52, NRAS, SRC, HRAS, GRB2, SDC2, PTPN11, NGF, SOS1, PIK3CA, SHC1, PIK3R1, PLCG1, FGFR2, CBL, CRK, GDNF, RET, NTRK1 |
| 216 | NFATC3 | 8.46E-15 | 2.08E-16 | 29 | 21 | PTGS2, JUN, FASLG, FOS, PPARG, CSF2, NFATC2, NFATC1, IFNG, IL2, IL2RA, IL4, MAPK9, CREM, EGR2, IL3, PPP3CA, EGR3, PPP3R1, PPP3CB, PPIA |
| 217 | CCR5 | 8.46E-15 | 2.08E-16 | 118 | 43 | POMC, ANXA1, STAT3, CD4, IL10, CSF2, KNG1, IFNG, JAK2, IL2, APP, IL8, IL4, GNAI2, IL12B, ADCY2, CCL2, CXCR4, SST, IL18, CXCR2, ADORA1, SSTR5, NPY, CXCL12, TLR2, CASR, GAL, ADCY4, OPRM1, CHRM2, BDKRB2, DRD2, ADRA2A, RGS4, PDYN, DRD3, NPY1R, DRD4, PNOC, NPY2R, ADRBK1, HCAR2 |
| 218 | MGST2 | 8.86E-15 | 2.21E-16 | 35 | 23 | GSTM1, CYP1A1, ADH7, GSTA2, GSTA4, GSTA1, GSTA3, GSTP1, ALDH3A1, CYP2B6, CYP3A4, CYP3A43, CYP3A5, CYP2C19, CYP1B1, GSTM2, GSTM3, GSTT2B, GSTO1, GSTZ1, GSTO2, GSS, GPX1 |
| 219 | NR3C1 | 8.86E-15 | 2.21E-16 | 35 | 23 | CREBBP, NCOA2, IL6, TP53, JUN, UBC, FOS, STAT3, CREB1, RELA, HSP90AA1, SRC, SMARCA4, SMAD4, NFKB1, HDAC1, TAT, CRH, SERPINE1, SMARCA2, HDAC6, GRIP1, PER2 |
| 220 | HTR1F | 8.86E-15 | 2.21E-16 | 35 | 23 | POMC, KNG1, GNAI2, ADCY2, SST, ADCY9, ADCY8, ADORA1, ADCY6, NPY, GAL, CHRM2, ADCY5, DRD2, HTR1A, PDYN, DRD3, DRD4, PNOC, NPY2R, HRH3, CHRM4, HTR1B |
| 221 | HTR5A | 9.23E-15 | 2.31E-16 | 32 | 22 | POMC, KNG1, GNAI2, ADCY2, SST, CXCR2, NPY, GAL, OPRM1, CHRM2, DRD2, HTR1A, ADRA2A, RGS4, PDYN, DRD3, NPY1R, DRD4, CHRM4, OPRD1, ADRA2C, HTR1B |
| 222 | CHRM1 | 9.43E-15 | 2.37E-16 | 45 | 26 | KNG1, GNA15, APP, F2R, LPAR2, PLCB2, GCG, PLCB3, GNRH1, GAST, BDKRB2, PLCB1, NMU, MCHR1, TBXA2R, CCK, TRH, RGS2, GRP, ADRBK1, TAC1, GNRHR, HCRT, GRK5, TACR1, CHRM3 |
| 223 | OPRM1 | 9.81E-15 | 2.48E-16 | 68 | 32 | POMC, KNG1, APP, GNAI2, ADCY2, CCR5, SST, CXCR2, ADORA1, NPY, PENK, CASR, GAL, CHRM2, BDKRB2, DRD2, HTR1A, NMU, PTGER3, ADRA2A, RGS4, PDYN, DRD3, DRD4, PNOC, HRH3, CHRM4, NMS, OPRD1, GPR55, ADRA2C, HTR1B |
| 224 | NPY1R | 1.28E-14 | 3.26E-16 | 49 | 27 | POMC, KNG1, ADCY2, CCR5, SST, ADCY8, CXCR2, NPY, PENK, ADCY7, GAL, CHRM2, DRD2, HTR1A, NMU, MCHR1, PTGER3, MTNR1A, ADRA2A, DRD3, DRD4, PNOC, NPY2R, HRH3, CHRM4, OPRD1, ADRA2C |
| 225 | HGF | 1.34E-14 | 3.42E-16 | 24 | 19 | IL6, JUN, INS-IGF2, STAT3, VEGFA, EGFR, ERBB2, TGFBI, CDH1, SRC, TGFB1, RAC1, IL8, ALB, RAC3, MET, MMP9, SDC1, CD44 |
| 226 | MTNR1A | 1.34E-14 | 3.42E-16 | 24 | 19 | POMC, ANXA1, GNAI2, ADCY2, SST, ADORA1, NPY, GAL, BDKRB2, DRD2, HTR1A, NMU, MCHR1, PTGER3, NPY1R, NPY2R, HRH3, SSTR2, ADRA2C |
| 227 | RASA1 | 1.55E-14 | 3.99E-16 | 53 | 28 | AKT1, NRAS, CSF1R, EGFR, EGF, BIRC5, SRC, HRAS, GRB2, FYN, SDC2, LCK, SHC1, RAF1, SOCS3, YES1, HCK, PDGFRB, GNB2L1, INPP5D, AURKB, PDGFB, ERBB4, GDNF, PTK2B, RET, EPHB2, EFNB2 |
| 228 | ADCY6 | 1.64E-14 | 4.23E-16 | 69 | 32 | POMC, PTH, KNG1, PDE4A, ADCY2, CRH, ADRB2, SST, CALCA, ADCY9, ADCY8, PKM, GCG, ADORA1, ADORA2A, PKLR, GNAS, GCGR, ADRB1, ADCY7, CASR, AVPR2, ADCY1, ADCY4, CHRM2, BDKRB2, ADCY5, DRD2, PDYN, DRD3, PRKACA, PDE8A |
| 229 | GNAS | 1.74E-14 | 4.54E-16 | 65 | 31 | POMC, UBC, PTH, GNA15, GNAI2, PDE4A, ADCY2, CRH, ADRB2, LHCGR, CALCA, ADCY9, ADCY8, GCG, ADM, IAPP, ADCY6, GCGR, ADRB1, ADCY7, AVPR2, ADCY1, HTR7, ADCY5, MC4R, GNG2, VIPR2, HTR4, ADRB3, HTR6, PDE8A |
| 230 | NCOA2 | 1.74E-14 | 4.54E-16 | 65 | 31 | CYP1A1, CREBBP, ESR1, RXRA, PPARA, EP300, BRCA1, FOS, CEBPB, SIN3A, PPARG, SIRT1, CARM1, CTNNB1, ABCA1, SP1, HIF1A, AR, TGFB1, LEP, ESR2, HDAC3, RORA, PPARGC1A, APOA1, LPL, SREBF2, KAT2B, NR3C1, CLOCK, NPAS2 |
| 231 | CRH | 1.77E-14 | 4.63E-16 | 61 | 30 | POMC, IL6, FOS, PTH, LEP, BDNF, NR3C1, ADCY2, ADRB2, LHCGR, CALCA, ADCY9, ADCY8, GCG, ADM, IAPP, ADCY6, NPY, ADORA2A, GNAS, GCGR, DRD5, ADRB1, AVPR2, ADCY5, MC4R, VIPR2, HTR4, ADRB3, GHRL |
| 232 | CAV1 | 1.89E-14 | 5.00E-16 | 46 | 26 | PTGS2, ESR1, TP53, INS-IGF2, AKT1, TNFRSF1A, TRADD, RIPK1, NOS3, EGFR, EGF, ABCB1, SRC, AR, TGFB1, SMAD2, RAC1, FYN, IRS1, INSR, PTPN1, LRP6, TGFBR2, EDNRB, ADRBK1, DNM2 |
| 233 | KIT | 1.89E-14 | 5.00E-16 | 46 | 26 | CREBBP, UBC, CHEK1, STAT3, NRAS, HRAS, STAT1, JAK2, GRB2, RAC1, FYN, PRKCA, PTPN11, SOS1, LCK, PIK3CA, PIK3R1, VAV1, PLCG1, YES1, CBL, PIK3CG, PTPN6, EPOR, PIK3R2, GRB10 |
| 234 | PPY | 2.39E-14 | 6.35E-16 | 33 | 22 | POMC, KNG1, APP, GNAI2, ADCY2, SST, SSTR5, NPY, PENK, CASR, GAL, OPRM1, HTR1A, NMU, MCHR1, PDYN, NPY1R, PNOC, NPY2R, HRH3, SSTR2, NMS |
| 235 | TACR2 | 2.43E-14 | 6.49E-16 | 50 | 27 | KNG1, GNA15, F2R, EDN1, GCG, GCGR, GNRH1, CASR, EDNRA, GAST, EDNRB, BDKRB2, PLCB1, CCK, TRH, GRP, NTS, ADRBK1, PTAFR, P2RY2, TAC1, NMUR2, GNRHR, HCRT, TACR1, CCKAR, CHRM3 |
| 236 | BDKRB1 | 2.75E-14 | 7.36E-16 | 43 | 25 | KNG1, GNAI2, F2R, EDN1, ADCY2, NPY, ADCY1, CHRM2, EDNRB, BDKRB2, PLCB1, DRD2, TBXA2R, PTGER3, PLCB4, CCK, PDYN, DRD3, PTAFR, P2RY2, HTR1B, TAC1, GRK5, TACR1, CHRM3 |
| 237 | CEBPB | 2.81E-14 | 7.58E-16 | 54 | 28 | MAPK1, MAPK3, CREBBP, ESR1, RXRA, NCOA2, JUN, PTGES2, EP300, MAPK14, MAPK11, RELA, SMARCA4, SP1, RB1, SMAD4, TGFB1, NFKB1, SMAD2, CEBPD, FOXO1, HDAC3, PPARGC1A, SREBF1, SRF, TTR, KLF5, SMARCA2 |
| 238 | TERT | 2.81E-14 | 7.58E-16 | 54 | 28 | CREBBP, JUN, UBC, AKT1, MYC, EP300, FOS, CCND1, SIN3A, E2F1, EGFR, EGF, HSP90AA1, IFNG, ARNT, SMARCA4, CTNNB1, SP1, HIF1A, NFKB1, HDAC1, IL2, CDKN1B, MAX, MTOR, NCL, RBBP4, ABL1 |
| 239 | ADCY1 | 3.01E-14 | 8.16E-16 | 58 | 29 | POMC, CREB1, PTH, APP, GNAI2, PDE4A, ADCY2, ADRB2, SST, CALCA, ADCY9, ADCY8, GCG, ADORA1, ADCY6, GNAS, GCGR, ADCY7, ADCY4, ADCY5, GNG2, DRD2, HTR1A, VIPR2, HTR4, ADRA2A, DRD4, PRKACA, PDE8A |
| 240 | CXCR4 | 3.02E-14 | 8.22E-16 | 62 | 30 | IL6, UBC, AKT1, STAT3, VEGFA, CD4, FGF2, IFNG, ARNT, HIF1A, STAT1, JAK2, RAC1, PTPN11, LCK, IL8, CDC42, JAK3, GNAI2, VAV1, SDC4, CCL2, CD44, CCR5, BCAR1, PTPN6, CXCL12, ADRBK1, DNM1, PTPRC |
| 241 | CYP2C19 | 3.12E-14 | 8.51E-16 | 79 | 34 | GSTM1, CYP2E1, UGT1A6, CYP1A2, CYP1A1, GSTA2, GSTA4, GSTA1, GSTA3, GSTP1, CYP2B6, CYP3A4, CYP3A43, CYP3A5, GSTM2, GSTM3, GSTT2B, GSTO1, GSTZ1, GSTO2, ALDH1A1, UGT1A1, CYP2D6, GPX1, PLA2G1B, PTGS2, PTGS1, PLA2G2A, ALDH1A2, MAOA, MAOB, PLA2G4A, PLA2G6, ALOX12 |
| 242 | YWHAQ | 3.73E-14 | 1.02E-15 | 47 | 26 | MAPK1, MAPK3, AKT1, YWHAZ, NRAS, NFATC2, NFATC1, HRAS, CTNNB1, FOXO1, PRKCA, YWHAG, YWHAB, RAF1, CDKN1B, YWHAE, YWHAH, BAD, KSR1, MTOR, CBL, HDAC4, ITGB4, PPP2R1A, RAN, CABIN1 |
| 243 | CRK | 4.85E-14 | 1.33E-15 | 67 | 31 | MAPK8, EGFR, SRC, IGF1, GRB2, RAC1, FYN, PRKCA, IRS1, PTPN11, SOS1, PIK3CA, PIK3R1, ITGAV, RAC3, IRS2, PTPN1, MET, PRKCD, YES1, CBL, BCAR1, PIK3R2, ABL1, PDGFRB, PTK2, PDGFB, GDNF, PTK2B, RET, IRS4 |
| 244 | IL2RB | 4.90E-14 | 1.36E-15 | 37 | 23 | AKT1, MYC, STAT3, HRAS, SYK, STAT1, JAK2, GRB2, IL2, FYN, JAK1, PTPN11, IL2RA, LCK, PIK3CA, SHC1, JAK3, PIK3R1, BCL2L1, MAPK9, SOCS3, PTPN6, CD247 |
| 245 | VCAM1 | 5.09E-14 | 1.41E-15 | 55 | 28 | IL6, INS-IGF2, IL1B, VEGFA, IL1A, SRC, IFNG, RAC1, PRKCA, IL4, APOA1, ALB, RAC3, ICAM1, PRKCD, CD44, EZR, IL18, ITGB1, CRP, B2M, CXCL12, ITGB2, PTK2B, PTAFR, HLA-DRB1, NCAM1, CYBB |
| 246 | FOXO3 | 5.88E-14 | 1.65E-15 | 34 | 22 | SOD2, UBC, AKT1, YWHAZ, FASLG, EP300, CEBPB, SIRT1, CTNNB1, SMAD4, SMAD2, FOXO1, YWHAG, ATM, YWHAB, CDKN1B, IKBKB, YWHAE, YWHAQ, YWHAH, KAT2B, PLK1 |
| 247 | ANGPT1 | 5.88E-14 | 1.65E-15 | 34 | 22 | AKT1, MAPK14, VEGFA, NOS3, NRAS, MMP2, RELA, HBEGF, HRAS, NFKB1, GRB2, PTPN11, PIK3CA, PIK3R1, FN1, PIK3CB, CRK, ITGB1, PIK3R2, RASA1, PLG, PDGFB |
| 248 | EDN2 | 6.03E-14 | 1.70E-15 | 28 | 20 | TP53, ANXA1, KNG1, GNA15, APP, EDN1, GCG, GNRH1, CASR, EDNRA, GAST, EDNRB, BDKRB2, MCHR1, CCK, TRH, GRP, NTS, TAC1, TACR1 |
| 249 | BAD | 6.03E-14 | 1.70E-15 | 28 | 20 | MAPK1, MAPK3, AKT1, YWHAZ, BCL2, YWHAG, NGF, CASP3, YWHAB, RAF1, YWHAE, YWHAQ, YWHAH, BCL2L1, MAPK9, SNCA, PPP3CA, NGFR, PPP3R1, PRKACA |
| 250 | NPY2R | 7.22E-14 | 2.04E-15 | 48 | 26 | POMC, KNG1, ADCY2, CCR5, SST, ADCY8, CXCR2, ADORA1, NPY, PENK, GAL, CHRM2, DRD2, MCHR1, MTNR1A, ADRA2A, RGS4, DRD3, NPY1R, DRD4, PNOC, HRH3, CHRM4, SSTR2, ADRA2C, NMUR2 |
| 251 | SCT | 8.00E-14 | 2.28E-15 | 41 | 24 | POMC, PTH, ADCY2, CRH, ADRB2, SST, LHCGR, CALCA, ADCY9, ADCY8, GCG, ADM, IAPP, ADCY6, ADORA2A, GNAS, GCGR, GAST, AVPR2, ADCY1, ADCY4, ADCY5, MC4R, VIPR2 |
| 252 | PPARG | 9.08E-14 | 2.60E-15 | 86 | 35 | PTGS2, MAPK8, ESR1, RXRA, PPARA, NCOA2, IL6, JUN, INS-IGF2, TNFRSF1A, EP300, VEGFA, CCND1, NFATC2, NFATC1, NFATC3, SP1, RB1, LEP, APOE, IL2, ESR2, NOS2, HDAC3, IL4, RORA, PPARGC1A, LPL, NR4A2, NR2F1, CCL2, SERPINE1, HMGA1, CDK5, UCP2 |
| 253 | CYP2C9 | 9.08E-14 | 2.60E-15 | 60 | 29 | GSTM1, CYP2E1, UGT1A6, CYP1A2, CYP1A1, GSTA2, GSTA4, GSTA1, GSTA3, GSTP1, CYP2B6, CYP3A4, CYP3A43, CYP3A5, GSTM2, GSTM3, GSTT2B, GSTZ1, ALDH1A1, UGT1A1, CYP2D6, GPX1, PLA2G1B, PTGS2, PTGS1, MAOA, MAOB, PLA2G4A, ALOX12 |
| 254 | CSF2RA | 1.02E-13 | 2.94E-15 | 16 | 15 | STAT3, NRAS, CSF2, HRAS, SYK, STAT1, JAK2, GRB2, SDC2, PTPN11, PIK3CA, IKBKB, PIK3R1, INPP5D, CSF2RB |
| 255 | CAMK2B | 1.06E-13 | 3.05E-15 | 23 | 18 | CREB1, HRAS, IFNG, STAT1, JAK2, MDM2, CALM1, CALM2, CALM3, PLCB3, GRIN2A, ACTN2, CAMK2G, CAMK2A, GRIN2D, GRIN2C, CACNG4, EPB41L1 |
| 256 | MC4R | 1.08E-13 | 3.15E-15 | 38 | 23 | POMC, PTH, LEP, ADCY2, CRH, ADRB2, LHCGR, CALCA, GCG, ADM, IAPP, NPY, GNAS, GCGR, ADRB1, SDC3, AVPR2, ADCY5, HTR4, TRH, ADRB3, AGRP, HTR6 |
| 257 | TSC2 | 1.08E-13 | 3.15E-15 | 38 | 23 | MAPK1, MAPK3, TP53, UBC, INS-IGF2, AKT1, YWHAZ, MYC, MAPK14, CCND1, MAPK11, SIRT1, GSK3B, FOXO1, YWHAG, YWHAB, IKBKB, YWHAE, YWHAQ, YWHAH, MAX, MTOR, AKT3 |
| 258 | MGST1 | 1.08E-13 | 3.15E-15 | 38 | 23 | GSTM1, CYP2E1, CYP1A2, CYP1A1, GSTA2, GSTA4, GSTA1, GSTA3, GSTP1, CYP2B6, CYP3A4, CYP3A43, CYP3A5, CYP2C19, CYP1B1, GSTM2, GSTM3, GSTT2B, GSTO1, GSTZ1, GSTO2, GSS, GPX1 |
| 259 | NGFR | 1.36E-13 | 3.99E-15 | 49 | 26 | TP53, UBC, UBA52, E2F1, RELA, RAC1, UBB, NFKBIA, NGF, APP, CASP3, PIK3CA, IKBKG, SHC1, BDNF, IKBKB, MYD88, BAD, MAPK9, PRKCZ, CASP6, ARHGDIA, NTRK1, NTF3, RHOB, NTRK2 |
| 260 | NMBR | 1.37E-13 | 4.06E-15 | 35 | 22 | KNG1, GNA15, PLCB2, GCG, GNRH1, EDNRA, GAST, EDNRB, BDKRB2, NMU, TBXA2R, CCK, TRH, GRP, NTS, ADRBK1, TAC1, GNRHR, HCRT, TACR1, CCKAR, CHRM3 |
| 261 | EZR | 1.37E-13 | 4.06E-15 | 35 | 22 | AKT1, CASP8, FADD, FASLG, EGFR, SRC, PRKCA, SDC2, LCK, ICAM1, CD44, CFTR, L1CAM, VCAM1, FAS, AP2M1, ACTB, ARHGDIA, SLC9A3R1, CDK5, DCC, SLC9A1 |
| 262 | GPX1 | 1.37E-13 | 4.06E-15 | 35 | 22 | GSTM1, GSTA2, GSTA4, GSTA1, GSTA3, GSTP1, CYP2C19, CYP2C9, CYP2C18, CYP2C8, GGT7, GSTM2, GSTM3, GSTT2B, GSTO1, GSTZ1, GSTO2, GSS, GGT1, SOD2, IL6, TP53 |
| 263 | HTR1B | 1.49E-13 | 4.44E-15 | 26 | 19 | POMC, GNAI2, ADCY2, ADCY9, ADORA1, SSTR5, NPY, PENK, GAL, OPRM1, CHRM2, DRD2, HTR1A, ADRA2A, DRD3, DRD4, HRH3, OPRD1, ADRA2C |
| 264 | SIRT1 | 1.52E-13 | 4.54E-15 | 61 | 29 | MAPK8, SOD2, NCOA2, TP53, JUN, FASLG, MYC, EP300, STAT3, NOS3, E2F1, TRIM28, RELA, ARNT, RB1, FOXO1, CDKN1B, PPARGC1A, SREBF1, IRS2, KAT2B, HDAC4, CSNK2A1, EPAS1, DVL1, MECP2, EZH2, UCP2, CTTN |
| 265 | ADRA2C | 1.59E-13 | 4.79E-15 | 32 | 21 | POMC, ADCY2, NPY, PENK, ADCY7, OPRM1, CHRM2, DRD2, HTR1A, NMU, MTNR1A, ADRA2A, RGS4, DRD3, NPY1R, DRD4, PNOC, NPY2R, CHRM4, OPRD1, HTR1B |
| 266 | GSN | 1.59E-13 | 4.79E-15 | 32 | 21 | EGFR, EGF, TGFBI, SRC, APP, CASP3, PIK3CA, APOA1, NPPA, PLCG1, SNCA, FN1, PRL, CALCA, IAPP, HSPG2, TTR, B2M, PTK2B, TNIK, TPM1 |
| 267 | TGFBI | 1.60E-13 | 4.86E-15 | 42 | 24 | PTGS2, VEGFA, EGF, SMAD3, IGF1, TGFB1, SMAD2, APP, APOA1, ITGAV, MMP9, NPPA, CCL2, FN1, PRL, CALCA, IAPP, ITGB1, DCN, HSPG2, TTR, B2M, GSN, ITGB2 |
| 268 | UTS2 | 1.60E-13 | 4.86E-15 | 42 | 24 | KNG1, F2R, EDN1, GCG, GCGR, GNRH1, CASR, EDNRA, GAST, EDNRB, NMU, MCHR1, CCK, TRH, RGS2, GRP, NTS, NMS, P2RY2, TAC1, GNRHR, HCRT, TACR1, OXTR |
| 269 | SSTR5 | 1.64E-13 | 5.02E-15 | 29 | 20 | POMC, PDZK1, KNG1, GNAI2, ADCY2, CCR5, SST, ADORA1, NPY, CASR, GAL, CHRM2, DRD2, HTR1A, MCHR1, PDYN, DRD4, CHRM4, SSTR2, HTR1B |
| 270 | FOXO4 | 1.64E-13 | 5.02E-15 | 29 | 20 | CREBBP, SOD2, UBC, AKT1, YWHAZ, EP300, CEBPB, SIRT1, CTNNB1, SMAD4, SMAD2, FOXO1, YWHAG, MDM2, YWHAB, CDKN1B, YWHAE, YWHAQ, YWHAH, MAPK9 |
| 271 | CYP1A1 | 1.68E-13 | 5.15E-15 | 74 | 32 | GSTM1, UGT1A6, GSTT1, GSTA2, GSTA4, GSTA1, GSTA3, GSTP1, CYP2B6, CYP3A4, CYP3A43, CYP3A5, CYP2C19, CYP2C9, CYP2C18, CYP2C8, CYP1B1, GSTM2, GSTM3, GSTT2B, GSTO1, GSTZ1, GSTO2, ALDH1A1, COMT, UGT1A1, ALDH1A2, CREBBP, ESR1, RXRA, PPARA, NCOA2 |
| 272 | VAV2 | 2.53E-13 | 7.81E-15 | 62 | 29 | AKT1, EGFR, SRC, SYK, JAK2, GRB2, RAC1, FYN, PTPN11, LCK, PIK3CA, CDC42, PIK3R1, VAV1, RAC3, PLCG1, CBL, PIK3CB, RHOA, PIK3CG, PIK3CD, HCK, RAC2, EPOR, PIK3R2, TYROBP, VAV3, SHC2, RHOB |
| 273 | CD44 | 3.22E-13 | 9.96E-15 | 71 | 31 | IL6, TP53, JUN, INS-IGF2, AKT1, MYC, STAT3, VEGFA, CD4, EGFR, EGF, ERBB2, SRC, IGF1, VIM, FYN, LCK, ALB, ICAM1, MMP9, FN1, CXCR4, VCAM1, EZR, B2M, ACTB, PTAFR, HLA-DRB1, NCAM1, PTPRC, SLC9A1 |
| 274 | PLAU | 3.24E-13 | 1.01E-14 | 43 | 24 | MAPK1, MAPK3, JUN, FOS, VEGFA, EGFR, EGF, FGF2, RELA, SRC, HRAS, NFKB1, PIK3CA, PIK3R1, ITGAV, MMP9, VLDLR, FN1, NCL, SERPINE1, ITGB1, PDGFRB, PLG, ITGB2 |
| 275 | ALDH3A1 | 3.58E-13 | 1.12E-14 | 24 | 18 | GSTM1, CYP2E1, GSTT1, ADH7, GSTA2, GSTA4, GSTA1, GSTA3, GSTP1, CYP2B6, ADH1B, ADH4, ADH1A, GSTM2, GSTO1, GSTO2, ADH6, ADH5 |
| 276 | KLF4 | 3.58E-13 | 1.12E-14 | 24 | 18 | CREBBP, RXRA, NCOA2, TP53, EP300, CEBPB, CCND1, RELA, CTNNB1, SP1, TGFB1, NFKB1, CEBPD, MED12, SREBF1, TCF7L2, HDAC2, KLF5 |
| 277 | ADRB1 | 3.83E-13 | 1.21E-14 | 33 | 21 | POMC, PTH, ADCY2, CRH, ADRB2, LHCGR, ADCY9, ADCY8, GCG, ADM, ADCY6, ADORA2A, GNAS, GCGR, DRD5, AVPR2, ADCY5, MC4R, VIPR2, HTR4, ADRB3 |
| 278 | CD86 | 3.83E-13 | 1.21E-14 | 33 | 21 | UBC, AKT1, CD4, IL10, IFNG, RAC1, FYN, PTPN11, IL2RA, LCK, PIK3CA, CDC42, PIK3R1, CTLA4, VAV1, ICAM1, B2M, TLR2, CD247, HLA-DRB1, LMNA |
| 279 | GRPR | 3.98E-13 | 1.26E-14 | 47 | 25 | KNG1, GNA15, PIK3CA, F2R, GCG, PLCB3, GCGR, GNRH1, EDNRA, GAST, EDNRB, BDKRB2, TBXA2R, CCK, TRH, GRP, NTS, ADRBK1, TAC1, GNRHR, HCRT, TACR1, CCKAR, CHRM3, OXTR |
| 280 | ERBB3 | 4.32E-13 | 1.37E-14 | 27 | 19 | UBC, AKT1, NRAS, EGFR, EGF, ERBB2, HRAS, MAP2K1, JAK2, GRB2, PTPN11, SOS1, SHC1, PIK3R1, ITGA6, ITGB4, ERBB4, NRG1, RGS4 |
| 281 | CCKAR | 4.46E-13 | 1.42E-14 | 51 | 26 | FOS, KNG1, GNA15, F2R, GCG, GCGR, GNRH1, CASR, EDNRA, GAST, EDNRB, BDKRB2, MCHR1, TBXA2R, CCK, TRH, GRP, NTS, ADRBK1, P2RY2, TAC1, GNRHR, HCRT, TACR1, CHRM3, OXTR |
| 282 | MAPK8 | 4.60E-13 | 1.48E-14 | 55 | 27 | GSTP1, TP53, JUN, INS-IGF2, FADD, MAP2K7, MYC, FOS, STAT3, BCL2, PPARG, SIRT1, NFATC2, HRAS, SMAD3, SMAD4, MAP3K7, IRS1, NGF, RAC3, IRS2, BCL2L1, MAPK8IP1, RHOA, CRK, RAC2, WWOX |
| 283 | PPARGC1A | 4.60E-13 | 1.48E-14 | 55 | 27 | CREBBP, RXRA, PPARA, NCOA2, UBC, EP300, MAPK14, CEBPB, MAPK11, PPARG, CREB1, SIRT1, CARM1, HIF1A, TGFB1, LEP, CEBPD, FOXO1, HDAC3, RORA, MED12, SREBF1, LPL, MTOR, CLOCK, ARNTL, YY1 |
| 284 | OXT | 6.24E-13 | 2.03E-14 | 44 | 24 | APP, EDN1, GCG, PLCB3, GNRH1, EDNRA, GAST, EDNRB, NMU, MCHR1, TBXA2R, CCK, TRH, RGS2, GRP, NTS, ADRBK1, P2RY2, TAC1, GNRHR, HCRT, TACR1, CCKAR, OXTR |
| 285 | YWHAG | 6.24E-13 | 2.03E-14 | 44 | 24 | MAPK1, MAPK3, AKT1, YWHAZ, AANAT, PAFAH1B1, NRAS, HRAS, CTNNB1, FOXO1, PRKCA, YWHAB, RAF1, CDKN1B, YWHAE, YWHAQ, YWHAH, BAD, KSR1, MTOR, PPP2R1A, RAN, NDE1, PCNT |
| 286 | CDH1 | 6.24E-13 | 2.03E-14 | 44 | 24 | PTGS2, UBC, AKT1, VEGFA, CCND1, EGFR, EGF, ERBB2, SRC, CTNNB1, TGFB1, SMAD2, RAC1, FYN, NOTCH1, CDC42, RAC3, CTNNA1, CTNND1, VCL, JUP, PSEN1, TJP1, ZEB2 |
| 287 | TLR4 | 6.24E-13 | 2.03E-14 | 44 | 24 | PTGS2, MAPK1, AKT1, RIPK1, IL1B, STAT3, IL10, EGFR, RELA, SRC, SYK, RAC1, NOS2, IL8, IKBKG, IKBKB, RAC3, ICAM1, MYD88, CCR5, HSPD1, CD86, PLCG2, HMGB1 |
| 288 | NTSR1 | 6.61E-13 | 2.16E-14 | 37 | 22 | KNG1, APP, F2R, GCG, GCGR, GNRH1, CASR, EDNRA, GAST, EDNRB, NMU, CCK, TRH, GRP, NTS, NMS, TAC1, NMUR2, HCRT, TACR1, CHRM3, OXTR |
| 289 | TAC3 | 7.35E-13 | 2.41E-14 | 48 | 25 | KNG1, F2R, EDN1, GCG, GCGR, GNRH1, EDNRA, GAST, EDNRB, BDKRB2, NMU, CCK, TRH, GRP, NTS, NMS, P2RY2, TAC1, NMUR2, GNRHR, HCRT, TACR1, CCKAR, CHRM3, OXTR |
| 290 | MLN | 8.20E-13 | 2.69E-14 | 22 | 17 | KNG1, GNA15, APP, GCG, GNRH1, EDNRA, GAST, EDNRB, NMU, CCK, TRH, GRP, NTS, TAC1, NMUR2, CCKAR, OXTR |
| 291 | RB1 | 9.12E-13 | 3.01E-14 | 92 | 35 | CREBBP, ESR1, TP53, JUN, UBC, UBA52, MYC, EP300, BRCA1, CEBPB, CCND1, E2F1, PPARG, SIRT1, SMARCA4, SP1, AR, CDK2, HDAC1, UBB, HDAC3, MDM2, DNMT1, RAF1, CDKN1B, MET, RBBP4, CTBP1, HDAC2, RBBP7, TBP, ABL1, GNB2L1, SMARCA2, PHB |
| 292 | SSTR2 | 1.08E-12 | 3.56E-14 | 31 | 20 | POMC, KNG1, GNAI2, ADCY2, SST, ADORA1, PTPN6, SSTR5, NPY, CASR, GAL, DRD2, HTR1A, MCHR1, MTNR1A, RGS4, PDYN, PNOC, NPY2R, CHRM4 |
| 293 | CCND3 | 1.20E-12 | 3.98E-14 | 45 | 24 | CREBBP, NCOA2, MYC, EP300, CCND1, E2F1, TRIM28, RB1, GRB2, CDK2, HDAC1, IL2, JAK1, HDAC3, PTPN11, IL2RA, PCNA, SOS1, LCK, PIK3CA, CDKN1B, JAK3, PIK3R1, MTOR |
| 294 | PTEN | 1.20E-12 | 4.01E-14 | 74 | 31 | SOD2, TP53, JUN, UBC, INS-IGF2, AKT1, FOS, VEGFA, CCND1, EGFR, SRC, SMAD4, NOTCH1, PIK3CA, CDKN1B, PIK3R1, PLCG1, MTOR, PIK3CB, PIK3CG, PIK3CD, PLCB2, XIAP, PIK3R2, PDGFRB, INPP5D, PTK2, PDGFB, SLC9A3R1, PLCE1, AKT3 |
| 295 | CSNK2A1 | 1.40E-12 | 4.69E-14 | 38 | 22 | TP53, JUN, SIN3A, SIRT1, RELA, HSP90AA1, CTNNB1, HDAC1, NFKBIA, HDAC3, CALM1, PTPN1, NCL, SRF, SLC18A2, HDAC2, DVL2, DVL3, DVL1, XRCC1, XRCC4, PTPRC |
| 296 | NMUR1 | 1.40E-12 | 4.69E-14 | 38 | 22 | POMC, KNG1, ADCY2, SST, NPY, GNRH1, GAL, DRD2, NMU, CCK, TRH, DRD3, PNOC, GRP, NTS, NMS, GPR55, TAC1, NMUR2, HCRT, CHRM3, OXTR |
| 297 | HCRT | 1.86E-12 | 6.26E-14 | 75 | 31 | FOS, KNG1, GNA15, APP, PIK3CA, PIK3R1, GCG, GCGR, GNRH1, CASR, GAST, EDNRB, NMU, MCHR1, CCK, TRH, RGS2, GRP, NTS, ADRBK1, NMS, P2RY2, TAC1, NMUR2, GNRHR, LTB4R, TACR1, GHRL, CCKAR, CHRM3, OXTR |
| 298 | CD40 | 1.94E-12 | 6.62E-14 | 35 | 21 | IL6, AKT1, TRAF1, TRAF2, MAPK14, MAPK11, CSF2, CAV1, RELA, STAT1, NFKB1, IL2, NFKBIA, BIRC2, JAK3, ICAM1, HSPA4, MAPK9, TRAF3, IL18, CD86 |
| 299 | GDNF | 1.94E-12 | 6.62E-14 | 35 | 21 | AKT1, FOS, CREB1, FGF2, SRC, HRAS, CTNNB1, GRB2, PRKCA, IRS1, PTPN11, PIK3CA, SHC1, PIK3R1, CRK, BCAR1, RASA1, GRB10, EDNRB, RET, NCAM1 |
| 300 | P2RY1 | 1.94E-12 | 6.62E-14 | 35 | 21 | MAPK14, KNG1, PIK3R1, F2R, EDN1, LPAR2, GCG, PLCB3, GNRH1, CASR, TBXA2R, CCK, TRH, RGS2, GRP, ADRBK1, PTAFR, P2RY2, LTB4R, GRK5, TACR1 |
| 301 | NPSR1 | 1.94E-12 | 6.62E-14 | 35 | 21 | POMC, PTH, KNG1, PIK3CA, EDN1, ADCY2, CRH, ADRB2, LHCGR, CALCA, CASR, GAST, NMU, TBXA2R, CCK, NTS, TAC1, NMUR2, GNRHR, HCRT, TACR1 |
| 302 | RAF1 | 2.23E-12 | 7.62E-14 | 46 | 24 | MAPK1, MAPK3, AKT1, YWHAZ, STUB1, BCL2, NRAS, HSP90AA1, HRAS, MAP2K1, RB1, RAC1, PRKCA, YWHAG, YWHAB, YWHAE, YWHAQ, YWHAH, BAD, KSR1, PRKCD, PRKCZ, RASA1, GRB10 |
| 303 | IKBKB | 2.30E-12 | 7.88E-14 | 71 | 30 | TP53, UBC, AKT1, TNFRSF1A, CASP8, TRADD, RIPK1, FADD, FASLG, TRAF2, UBA52, CSF2, RELA, NFKB1, MAP3K7, UBB, PRKCA, NFKBIA, IRS1, NGF, APP, IKBKG, MYD88, MAVS, PRKCZ, SKP1, TLR2, BTRC, NGFR, CSF2RA |
| 304 | KDR | 2.39E-12 | 8.24E-14 | 50 | 25 | IL6, UBC, VEGFA, FGF2, HSP90AA1, SRC, ARNT, CTNNB1, GRB2, FYN, PTPN11, SHC1, CDC42, ITGAV, PLCG1, CBL, PTPN6, ITGB1, EPAS1, PLCG2, PDGFB, PTK2B, SHC2, DNM2, NRP1 |
| 305 | IQGAP1 | 2.51E-12 | 8.69E-14 | 32 | 20 | MAPK1, MAPK3, UBC, PAFAH1B1, EGFR, ERBB2, CDH1, SRC, CTNNB1, MAP2K1, RAC1, CDC42, RAC3, CALM1, CALM2, CALM3, EZR, CTNNA1, CTNND1, S100B |
| 306 | SSTR1 | 3.00E-12 | 1.04E-13 | 29 | 19 | POMC, KNG1, ADCY2, CCR5, SST, LPAR2, ADORA1, SSTR5, NPY, PENK, CASR, GAL, DRD2, HTR1A, PDYN, DRD3, DRD4, CHRM4, SSTR2 |
| 307 | HTR4 | 3.12E-12 | 1.09E-13 | 26 | 18 | POMC, PTH, ADCY2, CRH, ADRB2, LHCGR, CALCA, ADCY8, GCG, GNAS, DRD5, ADRB1, AVPR2, ADCY1, HTR7, MC4R, VIPR2, HTR6 |
| 308 | CEBPD | 3.12E-12 | 1.09E-13 | 26 | 18 | CREBBP, RXRA, MYC, EP300, STAT3, CEBPB, CREB1, RELA, SP1, TGFB1, NFKB1, PPARGC1A, SREBF1, EGR2, TTR, TBP, KLF5, SMARCA2 |
| 309 | GHRH | 3.59E-12 | 1.26E-13 | 43 | 23 | POMC, FOS, PTH, LEP, ADCY2, CRH, ADRB2, SST, LHCGR, CALCA, GCG, ADM, IAPP, NPY, GNAS, GCGR, ADRB1, AVPR2, ADCY1, MC4R, VIPR2, TRH, GHRL |
| 310 | BAX | 5.75E-12 | 2.02E-13 | 33 | 20 | GPX1, CREBBP, TP53, JUN, AKT1, MYC, EP300, VEGFA, BCL2, SIRT1, RELA, SP1, GSK3B, PIK3CA, MAX, BCL2L1, PIK3CB, PIK3CG, PIK3CD, HSPD1 |
| 311 | CYP3A5 | 6.37E-12 | 2.25E-13 | 78 | 31 | GSTM1, CYP2E1, UGT1A6, CYP1A2, CYP1A1, GSTA2, GSTA4, GSTA1, GSTA3, GSTP1, CYP2B6, CYP2C19, CYP2C9, CYP2C18, CYP2C8, GSTM2, GSTM3, GSTT2B, GSTO1, GSTZ1, GSTO2, ALDH1A1, UGT1A1, CYP2D6, PLA2G1B, PLA2G2A, ALDH1A2, MAOA, MAOB, PLA2G4A, PLA2G6 |
| 312 | PLCB3 | 6.37E-12 | 2.25E-13 | 78 | 31 | CAMK2B, KNG1, GNA15, APP, PIK3CA, CALM1, DGKG, F2R, EDN1, PLCG1, PIK3CB, PIK3CG, PIK3CD, LPAR2, PLCB2, CAMK2G, EDNRA, PLCG2, BDKRB2, PLCB1, TBXA2R, PLCB4, CCK, GRP, PTAFR, PLCE1, P2RY2, TAC1, TACR1, CHRM3, OXTR |
| 313 | PLA2G1B | 6.77E-12 | 2.41E-13 | 44 | 23 | CYP2E1, CYP1A2, CYP2B6, CYP3A4, CYP3A43, CYP3A5, CYP2C19, CYP2C9, CYP2C18, CYP2C8, PTGS2, PTGS1, MAPK1, ALOX12, MAPK3, PLA2G7, FADS2, PEMT, IL6, PLD2, IL1B, PAFAH1B1, ANXA1 |
| 314 | CCL2 | 6.77E-12 | 2.41E-13 | 44 | 23 | IL6, JUN, INS-IGF2, IL1B, VEGFA, IL10, PPARG, CSF2, TGFBI, F3, IL23A, JAK2, IL8, ALB, ICAM1, IL12B, MMP9, CCR5, CXCR4, IL18, CXCR2, ATF4, CRP |
| 315 | CYP2C8 | 6.86E-12 | 2.45E-13 | 56 | 26 | GSTM1, CYP2E1, UGT1A6, CYP1A2, CYP1A1, GSTA2, GSTA4, GSTA1, GSTA3, GSTP1, CYP2B6, CYP3A4, CYP3A43, CYP3A5, GSTM2, GSTM3, GSTZ1, ALDH1A1, UGT1A1, CYP2D6, GPX1, PLA2G1B, PTGS2, PTGS1, MAOA, MAOB |
| 316 | FLT1 | 7.20E-12 | 2.59E-13 | 30 | 19 | GPC1, VEGFA, EGF, ARNT, HIF1A, PTPN11, PIK3CA, SHC1, PIK3R1, PLCG1, CBL, EPAS1, RASA1, PLG, PTK2, PDGFB, SHC2, AKT3, NRP1 |
| 317 | MLNR | 7.20E-12 | 2.59E-13 | 30 | 19 | GNA15, GCG, GCGR, GAST, EDNRB, NMU, MCHR1, TBXA2R, CCK, TRH, SLC9A3R1, GRP, NTS, NMS, TAC1, NMUR2, HCRT, TACR1, CCKAR |
| 318 | EPOR | 7.20E-12 | 2.59E-13 | 30 | 19 | CREBBP, UBC, AKT1, MAPK14, KIT, STAT1, JAK2, NFKB1, GRB2, JAK1, SOS1, SHC1, PIK3R1, IRS2, SOCS3, PLCG1, CBL, PTPN6, VAV2 |
| 319 | PTGIR | 8.22E-12 | 2.97E-13 | 24 | 17 | POMC, PTH, ADCY2, CRH, ADRB2, LHCGR, CALCA, ADCY9, ADCY8, GCG, ADM, ADCY6, GNAS, ADRB1, AVPR2, ADCY5, HTR4 |
| 320 | DNM1 | 8.23E-12 | 3.00E-13 | 27 | 18 | EGFR, EGF, SRC, GRB2, NOTCH1, NGF, IL8, PIK3R1, F2R, SH3GL2, CXCR4, CXCR2, AP2M1, CXCL12, GNB2L1, CDK5, CTTN, DNM2 |
| 321 | CBLB | 8.23E-12 | 3.00E-13 | 27 | 18 | UBC, KIT, EGFR, SYK, GRB2, FYN, JAK3, PIK3R1, VAV1, YES1, CBL, CRK, PIK3CD, ABL1, PLCG2, ERBB4, VAV2, RET |
| 322 | AVPR1B | 8.39E-12 | 3.08E-13 | 37 | 21 | KNG1, GNA15, PIK3CA, PIK3R1, GCG, PIK3R2, GCGR, GNRH1, MCHR1, TBXA2R, CCK, TRH, GRP, NTS, TAC1, GNRHR, HCRT, GRK5, CCKAR, CHRM3, OXTR |
| 323 | ADRA1B | 8.39E-12 | 3.08E-13 | 37 | 21 | KNG1, GNA15, APP, EDN1, PLCB3, GNRH1, EDNRA, GAST, TBXA2R, CCK, TRH, RGS2, ADRBK1, PTAFR, TAC1, GNRHR, HCRT, GRK5, TACR1, CHRM3, OXTR |
| 324 | YWHAH | 8.39E-12 | 3.08E-13 | 37 | 21 | MAPK1, MAPK3, AKT1, YWHAZ, NRAS, HRAS, CTNNB1, MAP2K1, FOXO1, YWHAG, YWHAB, RAF1, CDKN1B, YWHAE, YWHAQ, BAD, KSR1, MTOR, PPP2R1A, RAN, CSNK1E |
| 325 | CALM1 | 1.01E-11 | 3.72E-13 | 104 | 36 | INS-IGF2, AKT1, CAMK2B, PAFAH1B1, NOS3, EGFR, CREB1, BIRC5, HSP90AA1, SRC, HRAS, PYGB, NOS2, HDAC3, CDC42, INSR, ADCY2, CALM2, CALM3, DLG3, ADCY8, CSNK2A1, PLCB3, CAMK2G, PPP3CA, CAMK2A, PPP3R1, PPP3CB, CSNK1D, RGS4, CLASP2, NOS1, TPM1, MYO5A, GRK5, PPP3CC |
| 326 | CASP3 | 1.03E-11 | 3.81E-13 | 70 | 29 | MAPK1, AKT1, TNFRSF1A, CASP8, FADD, IL1B, FASLG, MAPK14, BCL2, NFATC2, BIRC5, SRC, VIM, SDC2, NGF, MDM2, BIRC2, YWHAE, SREBF1, BAD, PRKCD, HSPD1, XIAP, SPTAN1, CASP6, GSN, NGFR, KPNB1, DCC |
| 327 | ADCY9 | 1.03E-11 | 3.81E-13 | 70 | 29 | POMC, ANXA1, PTH, GNAI2, PDE4A, ADCY2, CRH, ADRB2, SST, CALCA, ADCY8, GCG, ADORA1, ADCY6, NPY, GNAS, GCGR, ADRB1, ADCY7, ADCY1, HTR7, ADCY4, ADCY5, DRD2, HTR1A, PTGER3, HTR1B, PRKACA, PDE8A |
| 328 | IL12B | 1.07E-11 | 3.96E-13 | 41 | 22 | IL6, IL1B, FASLG, FOS, STAT3, CD4, IL10, RELA, IFNG, IL23A, STAT1, JAK2, NFKB1, NFKBIA, LCK, IL4, PIK3R1, SOCS3, CCL2, CCR5, IL1R1, IGHG1 |
| 329 | EPHB2 | 1.20E-11 | 4.49E-13 | 34 | 20 | NRAS, SRC, HRAS, MAP3K7, RAC1, FYN, SDC2, LCK, PIK3CA, CDC42, PIK3R1, YES1, L1CAM, HCK, ABL1, RASA1, PTPN13, EFNB2, TIAM1, EPHB1 |
| 330 | CXCL12 | 1.20E-11 | 4.51E-13 | 45 | 23 | AKT1, STAT3, VEGFA, ARNT, HIF1A, STAT1, JAK2, RAC1, PTPN11, LCK, CDC42, GNAI2, VAV1, SDC4, MMP9, CCR5, CXCR4, VCAM1, CXCR2, PTPN6, ADRBK1, DNM1, PTPRC |
| 331 | TIMP1 | 1.20E-11 | 4.51E-13 | 45 | 23 | IL6, JUN, IL1B, FOS, STAT3, EGF, KNG1, MMP2, TGFBI, TGFB1, APP, ALB, MMP9, CD44, FN1, SERPINE1, MMP3, PLG, ALDOA, A2M, CLU, MMP14, SERPING1 |
| 332 | BCR | 1.20E-11 | 4.49E-13 | 34 | 20 | HSP90AA1, GRB2, RAC1, PTPN11, SOS1, PIK3CA, SHC1, PIK3R1, VAV1, CBL, PIK3CG, CRK, PIK3CD, HCK, RAC2, ABL1, ARHGDIA, VAV2, TIAM1, RHOB |
| 333 | UGT1A6 | 1.20E-11 | 4.49E-13 | 34 | 20 | CYP2E1, CYP1A2, CYP1A1, ADH7, CYP2B6, CYP3A4, CYP3A43, CYP3A5, CYP2C19, CYP2C9, CYP2C18, CYP2C8, ADH1B, ADH4, ADH1A, CYP1B1, ADH6, ALDH1A1, COMT, CYP2D6 |
| 334 | ITGAV | 1.26E-11 | 4.77E-13 | 66 | 28 | MAPK1, MAPK3, CASP8, VEGFA, CSF1R, MMP2, TGFBI, SRC, SYK, IGF1, FYN, PIK3CA, CDKN1B, PIK3R1, SDC4, SDC1, FN1, L1CAM, CRK, SERPINE1, BCAR1, ITGB1, PDGFRB, TGFBR2, PTK2, PDGFB, ITGB2, VAV3 |
| 335 | CYP3A43 | 1.34E-11 | 5.07E-13 | 80 | 31 | GSTM1, CYP2E1, UGT1A6, CYP1A2, CYP1A1, GSTA2, GSTA4, GSTA1, GSTA3, GSTP1, CYP2B6, CYP2C19, CYP2C9, CYP2C18, CYP2C8, GSTM2, GSTM3, GSTT2B, GSTO1, GSTZ1, GSTO2, ALDH1A1, UGT1A1, CYP2D6, PLA2G1B, PLA2G2A, ALDH1A2, MAOA, MAOB, PLA2G4A, PLA2G6 |
| 336 | ADRA2B | 1.60E-11 | 6.14E-13 | 31 | 19 | GNAI2, ADCY2, NPY, ADCY7, OPRM1, CHRM2, DRD2, HTR1A, MCHR1, ADRA2A, DRD3, NPY1R, DRD4, PNOC, NPY2R, CHRM4, OPRD1, ADRA2C, HTR1B |
| 337 | APC | 1.60E-11 | 6.14E-13 | 31 | 19 | PTGS2, TP53, CTNNB1, SP1, GSK3B, RAC1, NOTCH1, CDC42, CTBP1, SKP1, LRP6, BTRC, DVL2, CTNNA1, DVL3, CSNK1D, DVL1, JUP, CSNK1E |
| 338 | JUNB | 1.60E-11 | 6.14E-13 | 31 | 19 | MAPK8, IL6, JUN, EP300, MAPK14, FOS, STAT3, IL10, CSF2, NFATC2, NFATC1, NFATC3, MMP2, SP1, SMAD4, IL2, IL4, MMP9, MAPK9 |
| 339 | SNCA | 1.60E-11 | 6.14E-13 | 31 | 19 | UBC, PLD2, STUB1, SYK, FYN, LCK, APP, APOA1, BAD, PRKCD, IAPP, TTR, MAPT, PARK2, B2M, GSN, PARK7, GRK5, UCHL1 |
| 340 | NPBWR1 | 1.64E-11 | 6.30E-13 | 38 | 21 | POMC, KNG1, GNAI2, ADCY2, SST, NPY, PENK, CASR, GAL, OPRM1, NMU, MCHR1, PDYN, DRD3, NPY1R, PNOC, HRH3, CHRM4, NMS, OPRD1, HCAR2 |
| 341 | FOSL1 | 1.98E-11 | 7.68E-13 | 28 | 18 | HMOX1, IL6, JUN, MYC, EP300, FOS, CCND1, NFATC2, NFATC1, NFATC3, MMP2, SP1, IL2, IL8, MAX, MMP9, CCL2, ITGB4 |
| 342 | IL23A | 1.98E-11 | 7.68E-13 | 28 | 18 | IL6, JUN, IL1B, STAT3, CD4, IL10, RELA, IFNG, STAT1, JAK2, NFKB1, NFKBIA, PIK3CA, IL12B, SOCS3, CCL2, IL17A, IGHG1 |
| 343 | HSP90AA1 | 1.98E-11 | 7.71E-13 | 54 | 25 | ESR1, TP53, UBC, AKT1, STUB1, CHEK1, NOS3, HSPA8, EGFR, ERBB2, BIRC5, MMP2, SRC, HIF1A, AR, MAP3K7, RAF1, HSPA4, CALM1, NR3C1, CFTR, HSPD1, CSNK2A1, ABL1, HDAC6 |
| 344 | CD4 | 2.04E-11 | 7.94E-13 | 86 | 32 | UBC, FASLG, FOS, IL10, IFNG, IL23A, SYK, JAK2, FYN, PTPN11, IL2RA, LCK, SHC1, GNAI2, VAV1, IL12B, PLCG1, CBL, CD44, CCR5, CXCR4, SKP1, PTPN6, AP2M1, CD86, CD247, BTRC, GNG2, PPIA, IGHG1, HLA-DRB1, PTPRC |
| 345 | NCOA3 | 2.10E-11 | 8.27E-13 | 46 | 23 | MAPK1, CREBBP, ESR1, RXRA, PPARA, NCOA2, UBC, MYC, EP300, MAPK14, CCND1, PPARG, CARM1, AR, TGFB1, LEP, CEBPD, ESR2, HDAC3, PPARGC1A, KAT2B, NR3C1, EGR2 |
| 346 | GSK3B | 2.10E-11 | 8.25E-13 | 50 | 24 | NFE2L2, TP53, JUN, AKT1, MYC, CCND1, CREB1, NFATC2, NFATC1, APC, CTNNB1, MAX, PRKCZ, LRP6, MAPT, DPYSL2, DVL2, PPP1CC, DVL3, GLI2, PTK2B, DVL1, PSEN1, PRKACA |
| 347 | GNG2 | 2.10E-11 | 8.25E-13 | 50 | 24 | AKT1, CD4, SRC, GNA15, RAC1, PTPN11, PIK3CA, CDC42, GNAI2, ADCY2, ADRB2, PIK3CG, PLCB2, GNAS, ADCY1, CHRM2, EDNRB, PLCB1, DRD2, HTR1A, RGS4, RGS2, DRD4, ADRBK1 |
| 348 | PTGER4 | 2.16E-11 | 8.51E-13 | 25 | 17 | PTGS2, POMC, PTH, ADCY2, CRH, ADRB2, LHCGR, CALCA, ADCY9, ADCY8, ADM, ADCY6, ADORA2A, GNAS, DRD5, AVPR2, GNG2 |
| 349 | CARM1 | 2.82E-11 | 1.12E-12 | 59 | 26 | CREBBP, RXRA, PPARA, NCOA2, TP53, EP300, SIN3A, RELA, SRC, SMARCA4, ABCA1, SP1, HIF1A, HDAC3, RORA, PPARGC1A, MED12, SREBF2, CLOCK, NPAS2, ARNTL, MED15, ALAS1, HNRNPK, HIST1H3A, GRIP1 |
| 350 | RAC3 | 2.82E-11 | 1.12E-12 | 59 | 26 | MAPK1, MAPK8, AKT1, MAPK14, STAT3, VEGFA, CDH1, SRC, JAK2, NFKB1, PIK3CA, VAV1, ICAM1, MMP9, PIK3CB, PIK3CG, CRK, PIK3CD, VCAM1, PLCB2, ARHGDIA, VAV2, CDK5, CTTN, TIAM1, DNM2 |
| 351 | CYP3A4 | 2.89E-11 | 1.15E-12 | 87 | 32 | GSTM1, CYP2E1, UGT1A6, CYP1A2, CYP1A1, GSTA2, GSTA4, GSTA1, GSTA3, GSTP1, CYP2B6, CYP2C19, CYP2C9, CYP2C18, CYP2C8, GSTM2, GSTM3, GSTT2B, GSTO1, GSTZ1, GSTO2, ALDH1A1, UGT1A1, CYP2D6, PLA2G1B, PLA2G2A, ALDH1A2, MAOA, MAOB, PLA2G4A, PLA2G6, INS-IGF2 |
| 352 | CSF2RB | 2.95E-11 | 1.18E-12 | 17 | 14 | YWHAZ, STAT3, CSF2, SYK, STAT1, JAK2, JAK1, PTPN11, SHC1, PIK3R1, IL3, PTPN6, INPP5D, CSF2RA |
| 353 | EDN3 | 3.13E-11 | 1.25E-12 | 39 | 21 | KNG1, GNA15, APP, F2R, EDN1, GCG, GNRH1, EDNRA, GAST, EDNRB, BDKRB2, GNG2, NMU, TBXA2R, CCK, TRH, GRP, NTS, TAC1, TACR1, OXTR |
| 354 | BCL2 | 3.55E-11 | 1.43E-12 | 43 | 22 | MAPK1, MAPK3, CREBBP, MAPK8, RXRA, TP53, UBC, AKT1, CASP8, MYC, CREB1, SOD1, STAT1, CASP3, RAF1, MAX, BAD, BCL2L1, MTOR, PSEN1, BECN1, BIK |
| 355 | CD3E | 3.55E-11 | 1.43E-12 | 43 | 22 | JUN, FOS, CD4, IFNG, IL23A, SYK, JAK2, IL2, FYN, PTPN11, IL2RA, LCK, SHC1, PIK3R1, VAV1, IL12B, PLCG1, CBL, B2M, CD86, CD247, PTPRC |
| 356 | NCK1 | 3.65E-11 | 1.47E-12 | 64 | 27 | FASLG, VEGFA, EGFR, EGF, SRC, HRAS, SYK, RAC1, FYN, SOS1, CDC42, PIK3R1, VAV1, PLCG1, CBL, PIK3CD, BCAR1, ABL1, RASA1, PDGFRB, PLCG2, PTK2, PDGFB, EPHB2, DCC, EPHB1, WAS |
| 357 | NCOA1 | 4.47E-11 | 1.81E-12 | 60 | 26 | CREBBP, ESR1, RXRA, PPARA, NCOA2, JUN, EP300, FOS, CEBPB, PPARG, ARNT, CARM1, HIF1A, AR, SMAD4, TGFB1, LEP, CEBPD, ESR2, HDAC3, PPARGC1A, LPL, KAT2B, NR3C1, PRL, SRF |
| 358 | GAPDH | 4.57E-11 | 1.86E-12 | 29 | 18 | TP53, UBC, INS-IGF2, PLD2, AKT1, MYC, AR, IL2, APP, MAX, FN1, GPI, ACTB, PLG, ALDOA, TF, TKT, ALDOC |
| 359 | ADM | 4.85E-11 | 1.98E-12 | 36 | 20 | POMC, VEGFA, PTH, ARNT, HIF1A, EDN1, ADCY2, NPPA, CRH, ADRB2, LHCGR, CALCA, GCG, IAPP, ADORA2A, GNAS, GCGR, ADRB1, AVPR2, MC4R |
| 360 | ARRB1 | 4.85E-11 | 1.98E-12 | 36 | 20 | MAPK1, MAPK3, UBC, UBA52, UBB, NOTCH1, MDM2, IL8, F2R, SH3GL2, ADRB2, CCR5, HCK, CXCR2, ADRB1, RAB5A, ADRBK1, DNM1, GRK5, DNAJC6 |
| 361 | SHC2 | 4.88E-11 | 2.01E-12 | 20 | 15 | VEGFA, NRAS, EGFR, HRAS, GRB2, NGF, SOS1, SHC1, BDNF, INSR, PDGFRB, ERBB4, VAV2, NTRK1, NTRK2 |
| 362 | HCRTR1 | 4.88E-11 | 2.01E-12 | 20 | 15 | APP, PIK3CA, GCG, GNRH1, GAST, NMU, MCHR1, CCK, TRH, NTS, NMS, TAC1, HCRT, CCKAR, OXTR |
| 363 | STAT5A | 4.88E-11 | 2.01E-12 | 20 | 15 | CREBBP, ESR1, EP300, KIT, EGFR, SRC, JAK2, JAK1, PTPN11, JAK3, SOCS3, PRL, EPOR, ERBB4, PRLR |
| 364 | CDH2 | 5.19E-11 | 2.14E-12 | 56 | 25 | CREBBP, INS-IGF2, MAPK14, MAPK11, SRC, CTNNB1, VIM, RAC1, PTPN11, PIK3CA, BDNF, CDC42, PIK3R1, PTPN1, MET, MMP9, GJA1, ABL1, GNB2L1, CTNNA1, CTNND1, VCL, JUP, CTTN, NTRK2 |
| 365 | STUB1 | 5.45E-11 | 2.25E-12 | 26 | 17 | ESR1, TP53, UBC, UBA52, HSPA8, ERBB2, HSP90AA1, AR, UBB, RAF1, HSPA4, SNCA, CFTR, MAPT, PARK2, DNAJB1, UBE2N |
| 366 | IL5 | 5.72E-11 | 2.37E-12 | 23 | 16 | JUN, FOS, CSF2, NFATC2, NFATC1, NFATC3, IFNG, JAK2, GRB2, HDAC1, IL2, PTPN11, PIK3CA, IL4, IL3, CSF2RB |
| 367 | NMS | 6.26E-11 | 2.61E-12 | 48 | 23 | POMC, KNG1, F2R, SST, NPY, GNRH1, CASR, EDNRA, GAL, OPRM1, DRD2, HTR1A, NMU, CCK, TRH, GRP, NTS, OPRD1, TAC1, NMUR2, GNRHR, HCRT, OXTR |
| 368 | IAPP | 6.28E-11 | 2.62E-12 | 44 | 22 | POMC, INS-IGF2, PTH, TGFBI, FOXO1, APP, APOA1, ADCY2, NPPA, SNCA, CRH, PRL, CALCA, GCG, ADM, TTR, B2M, GNAS, GCGR, GSN, MC4R, ADRB3 |
| 369 | MAPK11 | 6.56E-11 | 2.75E-12 | 70 | 28 | PTGS2, PLA2G4A, ESR1, TP53, JUN, YWHAZ, MAPK14, FOS, CEBPB, CREB1, SRC, SYK, MAP3K7, RAC1, IL2, ESR2, FYN, JAK1, LCK, CDC42, TH, JAK3, PPARGC1A, SOCS3, SRF, CDH2, HCK, GDI1 |
| 370 | VIM | 7.13E-11 | 2.99E-12 | 33 | 19 | PLA2G4A, AKT1, CASP8, EGF, SRC, CASP3, CD44, CDH2, ACTN2, CASP6, AURKB, TF, TPM1, TPM2, DMD, DES, TNNI3, TNNT1, CRYAB |
| 371 | GRAP2 | 9.30E-11 | 3.94E-12 | 37 | 20 | CD4, KIT, HRAS, SYK, RAC1, FYN, LCK, SHC1, CDC42, VAV1, PLCG1, YES1, CBL, CRK, CD86, GRB10, CD247, VAV2, VAV3, WAS |
| 372 | NMUR2 | 9.30E-11 | 3.94E-12 | 37 | 20 | POMC, ADCY2, SST, NPY, GNRH1, GAL, DRD2, NMU, CCK, TRH, DRD3, PNOC, GRP, NPY2R, NTS, NMS, TAC1, HCRT, TACR1, OXTR |
| 373 | ADH4 | 9.30E-11 | 3.94E-12 | 37 | 20 | GSTM1, CYP2E1, UGT1A6, GSTA2, GSTA4, GSTA1, GSTA3, GSTP1, ALDH3A1, ALDH1A3, CYP2B6, ALDH1A1, COMT, UGT1A1, ALDH3A2, ALDH2, ALDH1A2, MAOA, ALDH9A1, ALDH7A1 |
| 374 | APLNR | 9.30E-11 | 3.94E-12 | 37 | 20 | POMC, VEGFA, KNG1, CCR5, SST, LPAR2, CXCR2, NPY, GAL, ADCY4, BDKRB2, DRD2, NMU, PTGER3, ADRA2A, PDYN, DRD4, PNOC, ADRA2C, HCAR2 |
| 375 | PTGFR | 1.00E-10 | 4.25E-12 | 30 | 18 | KNG1, GNA15, EDN1, PLCB2, PLCB3, GNRH1, EDNRB, PLCB1, MCHR1, TBXA2R, PLCB4, RGS2, GRP, TAC1, GNRHR, LTB4R, CHRM3, OXTR |
| 376 | TH | 1.14E-10 | 4.86E-12 | 18 | 14 | DDC, JUN, YWHAZ, MAPK14, FOS, MAPK11, IL4I1, GOT2, GOT1, NGF, TAT, TYR, CAMK2G, GAL |
| 377 | STAT5B | 1.14E-10 | 4.86E-12 | 18 | 14 | CREBBP, EP300, KIT, EGFR, SRC, IGF1, JAK2, JAK1, JAK3, INSR, NR3C1, SOCS3, PRL, PRLR |
| 378 | HBEGF | 1.30E-10 | 5.57E-12 | 27 | 17 | PTGS2, NRAS, EGFR, EGF, ERBB2, MMP2, HRAS, JAK2, PRKCA, SOS1, IL8, MMP9, PRL, MMP3, ERBB4, NRG1, PRLR |
| 379 | CSF1R | 1.50E-10 | 6.45E-12 | 21 | 15 | STAT3, CSF2, GRB2, PIK3R1, ITGAV, PTPN1, CBL, IL3, BCAR1, PIK3R2, RASA1, TYROBP, VAV3, C1QA, C1QB |
| 380 | DDC | 1.50E-10 | 6.45E-12 | 21 | 15 | COMT, MAOA, MAOB, DBH, IDO1, AANAT, IL4I1, GOT2, GOT1, TPH1, TAT, TH, TYR, PAH, TPH2 |
| 381 | OPRL1 | 1.51E-10 | 6.52E-12 | 24 | 16 | POMC, KNG1, ADCY2, SST, NPY, PENK, CASR, GAL, OPRM1, DRD2, NMU, MCHR1, PDYN, PNOC, SSTR2, OPRD1 |
| 382 | NPAS2 | 1.51E-10 | 6.52E-12 | 24 | 16 | CREBBP, RXRA, PPARA, NCOA2, EP300, CARM1, HIF1A, HDAC3, RORA, CLOCK, SERPINE1, ARNTL, BTRC, CSNK1E, PER2, CRY2 |
| 383 | SMAD2 | 1.54E-10 | 6.69E-12 | 77 | 29 | MAPK1, MAPK3, CREBBP, TP53, JUN, UBC, MYC, EP300, CEBPB, GPC1, SIN3A, CAV1, TGFBI, CDH1, SMARCA4, SMAD3, SP1, SMAD4, TGFB1, CDK2, HDAC1, FOXO1, MAX, KAT2B, FN1, CTBP1, MED15, CAMK2A, TGFBR2 |
| 384 | PLCB1 | 1.54E-10 | 6.69E-12 | 77 | 29 | KNG1, GNA15, PRKCA, APP, PIK3CA, DGKG, F2R, EDN1, PRKCD, PLCG1, PIK3CB, PIK3CG, PIK3CD, PLCB2, GCG, PLCB3, PIK3R2, PLCG2, EDNRB, BDKRB2, GNG2, PLCB4, CCK, PTAFR, PLCE1, P2RY2, TAC1, PLCD3, CHRM3 |
| 385 | CHUK | 1.62E-10 | 7.09E-12 | 63 | 26 | AKT1, TNFRSF1A, CASP8, TRADD, RIPK1, FADD, FASLG, TRAF2, MYC, CCND1, RELA, HSP90AA1, NFKB1, MAP3K7, PRKCA, NFKBIA, IRS1, NGF, APP, IKBKG, IKBKB, MYD88, MAVS, PRKCZ, SKP1, TLR2 |
| 386 | SMAD4 | 1.64E-10 | 7.16E-12 | 119 | 37 | MAPK1, CREBBP, MAPK8, ESR1, JUN, UBC, UBA52, MYC, EP300, FOS, CEBPB, SIN3A, LEF1, IL10, ARNT, SMAD3, SP1, HIF1A, TGFB1, SMAD2, CDK2, HDAC1, FOXO1, UBB, PTEN, MAX, KAT2B, NR3C1, RBBP4, CTBP1, SERPINE1, MED15, YY1, TGFBR2, BTRC, SHH, KPNB1 |
| 387 | ITGA6 | 1.74E-10 | 7.63E-12 | 38 | 20 | AKT1, YWHAZ, MYC, EGFR, EGF, IL1A, HRAS, GRB2, PRKCA, PIK3CA, YWHAB, PIK3R1, MET, ERBB3, FN1, ITGB1, ITGB4, ITGB2, PLEC, THBS1 |
| 388 | MMP2 | 1.89E-10 | 8.29E-12 | 42 | 21 | PTGS2, TP53, JUN, AKT1, IL1B, FOS, VEGFA, LEF1, HBEGF, HSP90AA1, SRC, SMARCA4, CTNNB1, IGF1, TGFB1, SDC2, ITGAV, DCN, MMP3, ITGB2, MMP14 |
| 389 | CYP1A2 | 2.02E-10 | 8.92E-12 | 59 | 25 | GSTM1, UGT1A6, GSTA2, GSTA4, GSTA1, GSTA3, GSTP1, CYP2B6, CYP3A4, CYP3A43, CYP3A5, CYP2C19, CYP2C9, CYP2C18, CYP2C8, GSTM2, GSTM3, GSTT2B, GSTO1, GSTZ1, GSTO2, ALDH1A1, UGT1A1, PLA2G1B, PLA2G6 |
| 390 | ALB | 2.08E-10 | 9.23E-12 | 73 | 28 | INS-IGF2, TXN, VEGFA, EGF, KNG1, TGFB1, LEP, APOE, SLC27A5, APP, IL8, APOA1, EDN1, CCL2, CD44, FN1, VCAM1, SERPINE1, TTR, ACTN2, PLG, CCK, ALDOA, A2M, TF, CLU, SERPING1, GHRL |
| 391 | PLCG2 | 2.08E-10 | 9.23E-12 | 73 | 28 | SRC, SYK, FYN, PRKCA, SOS1, LCK, PIK3CA, PIK3R1, VAV1, DGKG, PRKCD, PLCG1, CBL, PIK3CB, PIK3CG, PIK3CD, HCK, RAC2, PLCB2, PTPN6, PLCB3, PPP3CA, PLCB1, PPP3CB, PLCB4, PLCE1, PLCD3, PPP3CC |
| 392 | ITGB4 | 2.08E-10 | 9.29E-12 | 31 | 18 | AKT1, YWHAZ, MYC, EGFR, EGF, HRAS, GRB2, PRKCA, PIK3CA, YWHAB, SHC1, YWHAQ, MET, ERBB3, ITGB1, ITGA6, PTK2, PLEC |
| 393 | IL12RB1 | 2.08E-10 | 9.29E-12 | 31 | 18 | IL6, IL1B, FASLG, FOS, STAT3, CD4, IFNG, IL23A, STAT1, JAK2, NFKB1, LCK, IL4, IL12B, SOCS3, CCL2, CCR5, IL1R1 |
| 394 | CDKN1B | 2.18E-10 | 9.73E-12 | 88 | 31 | JUN, UBC, AKT1, YWHAZ, UBA52, MYC, BRCA1, FOS, CCND1, E2F1, SIRT1, SRC, IFNG, CTNNB1, RB1, GRB2, CDK2, FOXO1, UBB, YWHAG, PTEN, YWHAB, YWHAE, YWHAQ, ITGAV, YWHAH, MAX, SKP1, ABL1, CDK5, KPNA1 |
| 395 | PXN | 2.77E-10 | 1.24E-11 | 35 | 19 | MAPK8, INS-IGF2, SRC, SYK, PTPN11, LCK, MAPK9, CBL, CRK, BCAR1, ITGB1, ABL1, RASA1, PKLR, ITGA6, PTK2, VCL, PTK2B, CTTN |
| 396 | BIRC3 | 2.77E-10 | 1.24E-11 | 35 | 19 | UBC, TNFRSF1A, CASP8, TRADD, RIPK1, FADD, TRAF1, FASLG, TRAF2, BIRC5, RELA, NFKB1, NFKBIA, CASP3, IKBKG, BIRC2, TNFRSF1B, TRAF3, XIAP |
| 397 | KITLG | 2.88E-10 | 1.30E-11 | 28 | 17 | AKT1, STAT3, KIT, CSF2, IGF1, JAK2, GRB2, PTPN11, SOS1, PIK3CA, SHC1, PIK3R1, VAV1, PTPN6, EPOR, PARK2, GRB10 |
| 398 | CLOCK | 2.88E-10 | 1.30E-11 | 28 | 17 | CREBBP, RXRA, PPARA, NCOA2, EP300, CARM1, HDAC3, RORA, PPARGC1A, KAT2B, NPAS2, SERPINE1, ARNTL, BTRC, CSNK1E, PER2, CRY2 |
| 399 | ADCY7 | 3.13E-10 | 1.42E-11 | 47 | 22 | POMC, PTH, PDE4A, ADCY2, ADRB2, CALCA, ADCY9, ADCY8, GCG, ADCY6, GNAS, GCGR, ADCY1, ADCY4, ADCY5, VIPR2, ADRA2A, NPY1R, CHRM4, ADRA2C, PRKACA, PDE8A |
| 400 | RGS2 | 3.13E-10 | 1.42E-11 | 47 | 22 | ANXA1, KNG1, GNA15, F2R, EDN1, LPAR2, GCG, GNRH1, EDNRA, EDNRB, BDKRB2, GNG2, TBXA2R, CCK, GRP, P2RY2, TAC1, GNRHR, HCRT, TACR1, CHRM3, OXTR |
| 401 | NPFF | 3.15E-10 | 1.43E-11 | 39 | 20 | KNG1, GNRH1, CASR, GAST, NMU, MCHR1, CCK, TRH, GRP, NTS, ADRBK1, NMS, TAC1, NMUR2, GNRHR, LTB4R, HCRT, TACR1, CCKAR, OXTR |
| 402 | SOCS1 | 3.26E-10 | 1.48E-11 | 43 | 21 | IL6, STAT3, KIT, RELA, HRAS, IFNG, STAT1, JAK2, GRB2, IL2, JAK1, IRS1, IL2RA, LCK, IL4, JAK3, INSR, VAV1, IL12B, IRS2, SOCS3 |
| 403 | NOS2 | 3.62E-10 | 1.66E-11 | 25 | 16 | HMOX1, UBC, IL1B, NOS3, IL10, PPARG, IFNG, TGFB1, APOE, RAC1, IL4, MYD88, CALM1, EDN1, CALM2, NOS1 |
| 404 | CSNK1E | 3.62E-10 | 1.66E-11 | 25 | 16 | TP53, APC, CTNNB1, YWHAB, YWHAH, CLOCK, NPAS2, PLK1, ARNTL, BTRC, PPP2R1A, CSNK1D, GLI2, AKAP9, PER2, CRY2 |
| 405 | ADCYAP1R1 | 3.62E-10 | 1.66E-11 | 25 | 16 | POMC, PTH, ADCY2, CRH, ADRB2, LHCGR, CALCA, GCG, GNAS, GCGR, ADCY7, ADCY1, HTR7, ADCY4, MC4R, VIPR2 |
| 406 | CYP2C18 | 3.78E-10 | 1.74E-11 | 56 | 24 | GSTM1, CYP2E1, UGT1A6, CYP1A2, CYP1A1, GSTA2, GSTA4, GSTA1, GSTA3, GSTP1, CYP2B6, CYP3A4, CYP3A43, CYP3A5, GSTM2, GSTM3, GSTZ1, ALDH1A1, UGT1A1, CYP2D6, GPX1, PLA2G1B, PTGS2, PLA2G4A |
| 407 | SSTR3 | 4.01E-10 | 1.86E-11 | 22 | 15 | POMC, KNG1, ADCY2, CCR5, SST, ADCY6, SSTR5, NPY, PENK, GAL, DRD2, HTR1A, NMU, MCHR1, SSTR2 |
| 408 | MMP3 | 4.01E-10 | 1.86E-11 | 22 | 15 | IL1B, VEGFA, EGFR, FGF2, MMP2, IL1A, HBEGF, CTNNB1, PRKCA, BDNF, SDC4, PLG, CTNNA1, CTNND1, JUP |
| 409 | CRP | 4.01E-10 | 1.86E-11 | 22 | 15 | IL6, STAT3, VEGFA, NOS3, F3, LEP, IL8, ICAM1, CCL2, FN1, VCAM1, SERPINE1, C1QA, C1QB, PLAT |
| 410 | AXIN1 | 4.49E-10 | 2.09E-11 | 52 | 23 | TP53, UBC, UBA52, MYC, APC, CTNNB1, GSK3B, UBB, NOTCH1, PPP2CA, SKP1, CDH2, LRP6, TGFBR2, BTRC, DVL2, CTNNA1, CTNND1, DVL3, CSNK1D, DVL1, JUP, CSNK1E |
| 411 | SCTR | 5.15E-10 | 2.41E-11 | 36 | 19 | POMC, PTH, ADCY2, CRH, ADRB2, LHCGR, CALCA, ADCY9, ADCY8, GCG, ADM, IAPP, ADCY6, GCGR, DRD5, AVPR2, ADCY4, MC4R, VIPR2 |
| 412 | SMAD3 | 5.15E-10 | 2.41E-11 | 36 | 19 | MAPK1, CREBBP, MAPK8, JUN, UBC, EP300, MAPK14, TGFBI, CTNNB1, SP1, AR, SMAD4, TGFB1, SMAD2, HSPA4, SREBF2, KAT2B, YBX1, ZEB2 |
| 413 | GIPR | 5.15E-10 | 2.41E-11 | 36 | 19 | POMC, PTH, ADCY2, CRH, ADRB2, LHCGR, CALCA, ADCY8, GCG, ADM, IAPP, GNAS, GCGR, ADRB1, AVPR2, ADCY5, MC4R, VIPR2, HTR4 |
| 414 | P2RY6 | 5.54E-10 | 2.60E-11 | 44 | 21 | KNG1, GNA15, PIK3CA, F2R, EDN1, GCG, CASR, EDNRB, BDKRB2, CCK, RGS2, ADRBK1, PTAFR, P2RY2, TAC1, LTB4R, HCRT, GRK5, TACR1, CCKAR, CHRM3 |
| 415 | TEK | 6.11E-10 | 2.88E-11 | 29 | 17 | MAPK14, NRAS, MMP2, HRAS, GRB2, FYN, PTPN11, SOS1, PIK3CA, SHC1, PIK3R1, FN1, PIK3CB, CRK, ITGB1, RASA1, PLG |
| 416 | GPX2 | 6.11E-10 | 2.88E-11 | 29 | 17 | GSTM1, GSTA2, GSTA4, GSTA1, GSTA3, GSTP1, CYP2C19, CYP2C18, GGT7, GSTM2, GSTM3, GSTT2B, GSTO1, GSTZ1, GSTO2, GSS, GGT1 |
| 417 | AHR | 7.35E-10 | 3.47E-11 | 12 | 11 | CYP1A1, CYP1B1, ESR1, FOS, RELA, HSP90AA1, SRC, ARNT, SMARCA4, RB1, CCNT1 |
| 418 | MC2R | 8.27E-10 | 3.96E-11 | 26 | 16 | POMC, PTH, ADCY2, CRH, LHCGR, CALCA, ADCY9, GCG, ADCY6, GNAS, ADCY1, HTR7, ADCY4, ADCY5, MC4R, HTR4 |
| 419 | UGT1A9 | 8.27E-10 | 3.92E-11 | 33 | 18 | CYP2E1, CYP1A2, CYP1A1, CYP2B6, CYP3A4, CYP3A43, CYP3A5, CYP2C19, CYP2C9, CYP2C18, CYP2C8, ADH1B, ADH4, CYP1B1, ADH6, ALDH1A1, COMT, CYP2D6 |
| 420 | GPX4 | 8.27E-10 | 3.96E-11 | 26 | 16 | GSTM1, GSTA2, GSTA4, GSTA1, GSTA3, GSTP1, GGT7, GSTM2, GSTM3, GSTT2B, GSTO1, GSTZ1, GSTO2, GSS, GGT1, SOD2 |
| 421 | ADH6 | 8.27E-10 | 3.96E-11 | 26 | 16 | CYP2E1, UGT1A6, GSTA4, ALDH3A1, ALDH1A3, CYP2B6, ALDH1A1, COMT, UGT1A1, ALDH3A2, ALDH2, ALDH1A2, MAOA, ALDH9A1, ALDH7A1, ALDH1B1 |
| 422 | MGST3 | 8.27E-10 | 3.96E-11 | 26 | 16 | GSTM1, GSTA2, GSTA4, GSTA1, GSTA3, GSTP1, CYP2B6, CYP1B1, GSTM2, GSTM3, GSTT2B, GSTO1, GSTZ1, GSTO2, GSS, GPX1 |
| 423 | IL6R | 8.83E-10 | 4.24E-11 | 17 | 13 | IL6, STAT3, STAT1, JAK2, GRB2, FOXO1, JAK1, PTPN11, VAV1, SOCS3, HCK, IL6ST, CNTF |
| 424 | ADRB3 | 8.83E-10 | 4.24E-11 | 17 | 13 | POMC, PTH, LEP, ADCY2, CRH, ADRB2, GCG, IAPP, GNAS, GCGR, ADRB1, MC4R, VIPR2 |
| 425 | PLD2 | 9.27E-10 | 4.46E-11 | 45 | 21 | PLA2G1B, PLA2G2A, PLA2G4A, PLA2G6, MAPK1, MAPK3, PEMT, MBOAT1, MBOAT2, EGFR, SYK, FYN, PRKCA, DGKG, PTPN1, PRKCD, MTOR, SNCA, LPAR2, GAPDH, DPYSL2 |
| 426 | HSPA4 | 9.38E-10 | 4.54E-11 | 37 | 19 | ESR1, TP53, UBC, AKT1, STUB1, MYC, NOS3, ERBB2, HSP90AA1, SOD1, SMAD3, HIF1A, NQO1, MAX, CFTR, HSPD1, MAPT, PARK2, DNAJB1 |
| 427 | EPHA2 | 9.38E-10 | 4.54E-11 | 37 | 19 | TP53, SRC, CTNNB1, GRB2, RAC1, FYN, PTPN11, LCK, PIK3CA, PIK3R1, YES1, CBL, HCK, BCAR1, RASA1, CTNND1, VAV2, VAV3, TIAM1 |
| 428 | CYP4A22 | 9.66E-10 | 4.70E-11 | 41 | 20 | CYP2E1, CYP1A2, CYP1A1, CYP2B6, CYP3A4, CYP3A43, CYP3A5, CYP2C19, CYP2C9, CYP2C18, CYP2C8, ALDH1A1, UGT1A1, ALDH3A2, PTGS2, PTGS1, PLA2G2A, ALDH1A2, ALDH9A1, PLA2G4A |
| 429 | RPS6KB1 | 9.66E-10 | 4.70E-11 | 41 | 20 | INS-IGF2, AKT1, KIT, IGF1, TGFB1, GRB2, RAC1, IL2, IRS1, PIK3CA, PTEN, CDC42, PIK3R1, INSR, PPP2CA, MTOR, PIK3CB, PIK3CG, CRK, PIK3CD |
| 430 | IL23R | 1.06E-09 | 5.18E-11 | 20 | 14 | IL6, IL1B, STAT3, CD4, IFNG, IL23A, STAT1, JAK2, NFKB1, NFKBIA, PIK3CA, IL12B, SOCS3, CCL2 |
| 431 | MAP3K5 | 1.24E-09 | 6.09E-11 | 30 | 17 | GSTM1, JUN, UBC, AKT1, TNFRSF1A, CASP8, TRADD, RIPK1, FADD, YWHAZ, TRAF1, FASLG, TRAF2, MAP2K7, CAMK2B, STUB1, TXN |
| 432 | ERBB4 | 1.24E-09 | 6.09E-11 | 30 | 17 | UBC, EGF, ERBB2, HBEGF, PIK3CA, PIK3R1, ERBB3, PLCG1, PIK3CB, PIK3CG, YAP1, PIK3CD, PIK3R2, WWOX, RASA1, NRG1, SHC2 |
| 433 | ETS1 | 1.24E-09 | 6.09E-11 | 30 | 17 | MAPK1, MAPK3, CREBBP, TP53, JUN, EP300, FOS, VEGFA, CCND1, CSF1R, HRAS, ARNT, HIF1A, JAK1, IL4, JAK3, TAL1 |
| 434 | GLP1R | 1.54E-09 | 7.63E-11 | 34 | 18 | POMC, INS-IGF2, PTH, ADCY2, CRH, ADRB2, LHCGR, CALCA, ADCY8, GCG, ADM, IAPP, GCGR, ADCY7, ADCY5, MC4R, VIPR2, HTR4 |
| 435 | CTTN | 1.54E-09 | 7.63E-11 | 34 | 18 | SIRT1, SRC, CTNNB1, SYK, GRB2, RAC1, FYN, RAC3, PTPN1, CDH2, SDC3, CTNNA1, CTNND1, HDAC6, DNM1, TJP1, DNM2, WAS |
| 436 | UGT1A7 | 1.54E-09 | 7.63E-11 | 34 | 18 | CYP2E1, CYP1A2, CYP1A1, CYP2B6, CYP3A4, CYP3A43, CYP3A5, CYP2C19, CYP2C9, CYP2C18, CYP2C8, ADH1B, ADH4, CYP1B1, ADH6, ALDH1A1, COMT, CYP2D6 |
| 437 | BCL2L1 | 1.54E-09 | 7.63E-11 | 34 | 18 | MAPK8, TP53, AKT1, CASP8, STAT3, BCL2, STAT1, IL2, JAK1, LCK, PIK3CA, JAK3, PIK3R1, BAD, MTOR, PIK3CB, BECN1, BIK |
| 438 | MMP1 | 1.54E-09 | 7.63E-11 | 34 | 18 | IL6, JUN, IL1B, EP300, FOS, VEGFA, FGF2, MMP2, IL1A, IGF1, TGFB1, EDN1, MMP9, CCL2, SDC1, MMP3, CRP, EDNRA |
| 439 | CTNND1 | 1.65E-09 | 8.21E-11 | 69 | 26 | EGFR, EGF, CDH1, SRC, CTNNB1, IGF1, RAC1, FYN, PTPN11, PIK3CA, BDNF, CDC42, MET, YES1, CDH2, PTPN6, MMP3, ABL1, CTNNA1, VCL, JUP, RET, PSEN1, CTTN, TJP1, DNM2 |
| 440 | ALDH1A1 | 1.65E-09 | 8.21E-11 | 42 | 20 | UGT1A6, CYP1A2, CYP1A1, ADH7, CYP2B6, CYP3A4, CYP3A43, CYP3A5, CYP2C19, CYP2C9, CYP2C18, CYP2C8, ADH1B, ADH4, ADH1A, ADH6, UGT1A1, ADH5, RDH10, RDH5 |
| 441 | HTR2A | 1.66E-09 | 8.32E-11 | 38 | 19 | GNA15, APP, F2R, PLCB2, GNRH1, EDNRB, PLCB1, MCHR1, TBXA2R, CCK, TRH, RGS2, ADRBK1, PTAFR, GNRHR, HCRT, GRK5, TACR1, CHRM3 |
| 442 | LCP2 | 1.66E-09 | 8.32E-11 | 38 | 19 | CD4, SYK, GRB2, RAC1, FYN, LCK, CDC42, VAV1, PLCG1, CBL, CRK, PTPN6, CD247, PLCG2, TYROBP, VAV2, VAV3, PTPRC, WAS |
| 443 | CTLA4 | 1.97E-09 | 9.92E-11 | 15 | 12 | IDO1, AKT1, FASLG, IL10, NFATC2, IFNG, FYN, PTPN11, LCK, PIK3R1, AP2M1, CD86 |
| 444 | SELE | 1.97E-09 | 9.92E-11 | 15 | 12 | JUN, IL1B, VEGFA, CD4, RELA, IL1A, IFNG, NFKB1, PRKCA, ICAM1, CD44, VCAM1 |
| 445 | NCOR2 | 2.08E-09 | 1.05E-10 | 51 | 22 | CREBBP, ESR1, RXRA, PPARA, NCOA2, AKT1, EP300, CEBPB, SIN3A, PPARG, RELA, CARM1, AR, TGFB1, HDAC1, NOTCH1, HDAC3, PPARGC1A, LPL, HDAC4, HDAC2, MED15 |
| 446 | GRB10 | 2.36E-09 | 1.20E-10 | 24 | 15 | INS-IGF2, AKT1, KIT, EGFR, IGF1, PRKCA, SHC1, RAF1, INSR, MYD88, PDGFRB, PDGFB, GDNF, RET, EPHB1 |
| 447 | NTRK1 | 2.36E-09 | 1.20E-10 | 24 | 15 | UBC, GRB2, IRS1, NGF, SHC1, BDNF, PIK3R1, IRS2, PLCG1, CBL, PTPN6, ABL1, NGFR, SHC2, NTF3 |
| 448 | INPPL1 | 2.43E-09 | 1.24E-10 | 31 | 17 | AKT1, PTEN, SHC1, INSR, MET, PLCG1, CBL, PLCB2, PTPN6, PLCB3, ABL1, INPP5D, PLCG2, PLCB1, PLCB4, PLCE1, PLCD3 |
| 449 | VIPR2 | 2.43E-09 | 1.24E-10 | 31 | 17 | POMC, PTH, ADCY2, CRH, ADRB2, LHCGR, CALCA, GCG, GNAS, GCGR, DRD5, ADRB1, ADCY7, ADCY1, ADCY4, HTR4, ADRB3 |
| 450 | PLA2G4A | 2.44E-09 | 1.25E-10 | 47 | 21 | CYP2B6, CYP3A4, CYP3A43, CYP3A5, CYP2C19, CYP2C9, CYP2C18, PTGS2, PTGS1, PLA2G6, MAPK1, ALOX12, PLA2G7, FADS2, PLD2, MAPK14, MAPK11, MAP2K1, SYK, GNA15, VIM |
| 451 | PLG | 2.58E-09 | 1.32E-10 | 56 | 23 | VEGFA, EGF, KNG1, F3, IGF1, TGFB1, NGF, APP, BDNF, ALB, SDC4, MMP9, FN1, SERPINE1, GAPDH, MMP3, ITGB2, ALDOA, A2M, CLU, THBS1, PLAT, SERPING1 |
| 452 | GRIN2A | 2.74E-09 | 1.40E-10 | 18 | 13 | CAMK2B, SRC, HRAS, FYN, PRKCA, RELN, DLG3, ACTN2, CAMK2A, GRIN2D, GRIN2C, RASGRF1, AKAP9 |
| 453 | MAP3K1 | 2.74E-09 | 1.41E-10 | 43 | 20 | MAPK8, TRADD, RIPK1, FADD, TRAF2, MAP2K7, MAPK14, IFNG, MAP2K1, JAK2, SMAD2, MAP3K7, RAC1, JAK1, CASP3, IKBKG, CDC42, IKBKB, MYD88, CRK |
| 454 | OPN4 | 2.75E-09 | 1.42E-10 | 21 | 14 | GNA15, PLCB2, GCG, PLCB3, GNRH1, EDNRA, PLCB1, NMU, PLCB4, CCK, GRP, NTS, TAC1, HCRT |
| 455 | PLA2G12B | 2.75E-09 | 1.42E-10 | 21 | 14 | CYP2E1, CYP1A2, CYP3A4, CYP3A43, CYP3A5, CYP2C19, CYP2C9, CYP2C18, CYP2C8, PTGS2, PTGS1, PLA2G7, FADS2, PEMT |
| 456 | VAV3 | 2.75E-09 | 1.42E-10 | 21 | 14 | CSF1R, SYK, GRB2, RAC1, FYN, LCK, CDC42, ITGAV, VAV1, PLCG1, CBL, BCAR1, TYROBP, VAV2 |
| 457 | MTNR1B | 2.75E-09 | 1.42E-10 | 21 | 14 | POMC, ANXA1, GNAI2, ADCY2, NPY, ADCY5, HTR1A, MCHR1, MTNR1A, ADRA2A, NPY1R, DRD4, NPY2R, HRH3 |
| 458 | CRKL | 2.76E-09 | 1.44E-10 | 35 | 18 | MAPK8, IGF1, JAK2, GRB2, FYN, IRS1, PTPN11, PIK3R1, MET, VLDLR, RELN, CBL, CRK, BCAR1, ITGB1, EPOR, PIK3R2, ABL1 |
| 459 | UGT1A5 | 2.76E-09 | 1.44E-10 | 35 | 18 | CYP2E1, CYP1A2, CYP1A1, CYP2B6, CYP3A4, CYP3A43, CYP3A5, CYP2C19, CYP2C9, CYP2C18, CYP2C8, ADH1B, ADH4, CYP1B1, ADH6, ALDH1A1, COMT, CYP2D6 |
| 460 | UGT2B10 | 2.76E-09 | 1.44E-10 | 35 | 18 | CYP2E1, CYP1A2, CYP1A1, ADH7, CYP2B6, CYP3A4, CYP3A43, CYP3A5, CYP2C19, CYP2C9, CYP2C18, CYP2C8, ADH4, CYP1B1, ADH6, ALDH1A1, COMT, CYP2D6 |
| 461 | HTR2C | 2.84E-09 | 1.49E-10 | 39 | 19 | KNG1, GNA15, F2R, PLCB2, GCG, GNRH1, MCHR1, CCK, TRH, RGS2, GRP, ADRBK1, NMS, TAC1, HCRT, GRK5, TACR1, CCKAR, CHRM3 |
| 462 | PLCB2 | 3.16E-09 | 1.65E-10 | 81 | 28 | MAPK14, ANXA1, KNG1, GNA15, RAC1, APP, PIK3CA, PTEN, RAC3, DGKG, F2R, PLCG1, PIK3CB, PIK3CG, PIK3CD, GCG, PLCB3, PLCG2, PLCB1, GNG2, MCHR1, PLCB4, CCK, PTAFR, PLCE1, P2RY2, GNRHR, CHRM3 |
| 463 | CYP4A11 | 3.16E-09 | 1.66E-10 | 52 | 22 | CYP2E1, UGT1A6, CYP1A2, CYP1A1, CYP2B6, CYP3A4, CYP3A43, CYP3A5, CYP2C19, CYP2C9, CYP2C18, CYP2C8, ALDH1A1, UGT1A1, PLA2G1B, ALDH3A2, PTGS2, PTGS1, PLA2G2A, ALDH1A2, ALDH9A1, PLA2G4A |
| 464 | ARNT | 3.16E-09 | 1.66E-10 | 52 | 22 | HMOX1, CREBBP, AKT1, EP300, VEGFA, SIRT1, ABCB1, SP1, HIF1A, SMAD4, LEP, EDN1, CXCR4, SERPINE1, PKM, ADM, EPAS1, CXCL12, SLC2A1, CITED2, ALDOA, TF |
| 465 | APOA1 | 3.16E-09 | 1.66E-10 | 52 | 22 | CREBBP, RXRA, PPARA, NCOA2, TGFBI, ABCA1, APOE, APP, ALB, LPL, NPPA, SNCA, PRL, VCAM1, CALCA, IAPP, TTR, APOA2, B2M, GSN, A2M, TF |
| 466 | GPX7 | 3.57E-09 | 1.90E-10 | 28 | 16 | GSTM1, GSTT1, GSTA2, GSTA4, GSTA1, GSTA3, GSTP1, GGT7, GSTM2, GSTM3, GSTT2B, GSTO1, GSTZ1, GSTO2, GSS, GGT1 |
| 467 | PLA2G6 | 3.57E-09 | 1.90E-10 | 28 | 16 | CYP1A2, CYP2B6, CYP3A4, CYP3A43, CYP3A5, CYP2C19, PTGS2, PTGS1, PLA2G4A, ALOX12, PLA2G7, FADS2, PEMT, PLD2, MBOAT1, MBOAT2 |
| 468 | FFAR1 | 3.57E-09 | 1.90E-10 | 28 | 16 | PLCB2, GCG, GCGR, CASR, GAST, NMU, CCK, TRH, GRP, NTS, PTAFR, TAC1, LTB4R, HCRT, CCKAR, CHRM3 |
| 469 | GPX3 | 3.57E-09 | 1.90E-10 | 28 | 16 | GSTM1, GSTA2, GSTA4, GSTA1, GSTA3, GSTP1, CYP2C19, GGT7, GSTM2, GSTM3, GSTT2B, GSTO1, GSTZ1, GSTO2, GSS, GGT1 |
| 470 | UGT2B4 | 3.57E-09 | 1.90E-10 | 28 | 16 | CYP2E1, CYP1A2, CYP1A1, CYP2B6, CYP3A4, CYP3A43, CYP3A5, CYP2C19, CYP2C9, CYP2C18, CYP2C8, ADH4, CYP1B1, ALDH1A1, COMT, CYP2D6 |
| 471 | CALM3 | 3.80E-09 | 2.03E-10 | 48 | 21 | INS-IGF2, CAMK2B, PAFAH1B1, NOS3, EGFR, CREB1, BIRC5, SRC, HDAC3, CDC42, INSR, CALM1, ADCY2, CALM2, ADCY8, CAMK2G, CSNK1D, CLASP2, NOS1, TPM1, MYO5A |
| 472 | GSTT1 | 3.87E-09 | 2.07E-10 | 13 | 11 | GSTM1, CYP1A1, ADH7, GSTA2, GSTA4, GSTA1, GSTA3, GSTP1, ALDH3A1, ALDH1A3, GGT7 |
| 473 | IDO1 | 3.87E-09 | 2.07E-10 | 13 | 11 | CYP1B1, MAOA, DDC, AANAT, IFNG, KYNU, IL4I1, TPH1, CTLA4, AFMID, WARS |
| 474 | NOTCH1 | 4.07E-09 | 2.19E-10 | 109 | 33 | CREBBP, TP53, JUN, UBC, AKT1, UBA52, MYC, EP300, FOS, STAT3, VEGFA, CCND1, E2F1, EGFR, CDH1, APC, CTNNB1, HIF1A, NFKB1, HDAC1, UBB, LCK, PTEN, IL4, KAT2B, CTBP1, SKP1, YY1, DVL1, PSEN1, EFNB2, DNM1, FURIN |
| 475 | SMARCA4 | 4.24E-09 | 2.28E-10 | 62 | 24 | CREBBP, NFE2L2, ESR1, TP53, MYC, BRCA1, CEBPB, SIN3A, MMP2, CARM1, CTNNB1, SP1, RB1, STAT1, AR, SMAD2, HDAC1, NR3C1, SRF, HDAC2, CHD4, ACTB, SMARCA2, PHB |
| 476 | COMT | 4.45E-09 | 2.41E-10 | 32 | 17 | UGT1A6, CYP1A1, ADH7, ADH1B, ADH4, ADH1A, CYP1B1, ADH6, UGT1A1, ADH5, ALDH3A2, ALDH2, MAOA, MAOB, DDC, DBH, AHCY |
| 477 | UGT1A8 | 4.45E-09 | 2.41E-10 | 32 | 17 | CYP2E1, CYP1A2, CYP1A1, CYP2B6, CYP3A4, CYP3A43, CYP3A5, CYP2C19, CYP2C9, CYP2C18, CYP2C8, ADH1B, ADH4, CYP1B1, ADH6, COMT, CYP2D6 |
| 478 | UGT1A10 | 4.45E-09 | 2.41E-10 | 32 | 17 | CYP2E1, CYP1A2, CYP1A1, CYP2B6, CYP3A4, CYP3A43, CYP3A5, CYP2C19, CYP2C9, CYP2C18, CYP2C8, ADH1B, ADH4, CYP1B1, ADH6, COMT, CYP2D6 |
| 479 | EFNB1 | 4.45E-09 | 2.41E-10 | 32 | 17 | NRAS, ERBB2, SRC, HRAS, GRB2, RAC1, FYN, LCK, PIK3CA, CDC42, HCK, RASA1, PTPN13, EPHB2, EFNB2, TIAM1, EPHB1 |
| 480 | UGT1A1 | 4.77E-09 | 2.59E-10 | 40 | 19 | CYP2E1, CYP1A2, CYP1A1, ADH7, CYP2B6, CYP3A4, CYP3A43, CYP3A5, CYP2C19, CYP2C9, CYP2C18, CYP2C8, ADH1B, ADH4, CYP1B1, ADH6, ALDH1A1, COMT, CYP2D6 |
| 481 | LTB4R | 4.77E-09 | 2.61E-10 | 53 | 22 | ANXA1, KNG1, GNA15, PIK3CA, F2R, GCG, GNRH1, CASR, TBXA2R, CCK, TRH, NTS, ADRBK1, PTAFR, P2RY2, TAC1, GNRHR, HCRT, GRK5, TACR1, CHRM3, OXTR |
| 482 | SDC4 | 4.77E-09 | 2.61E-10 | 53 | 22 | MAPK1, MAPK3, GPC1, FGF2, RAC1, PRKCA, SDC2, ITGAV, MMP9, PRKCD, SDC1, FN1, CXCR4, DCN, HSPG2, MMP3, CXCL12, SDC3, PLG, GPC6, THBS1, DNM2 |
| 483 | NPY5R | 5.00E-09 | 2.74E-10 | 25 | 15 | POMC, ADCY2, SST, NPY, PENK, GAL, OPRM1, HTR1A, NMU, MCHR1, DRD3, NPY1R, NPY2R, ADRA2C, HTR1B |
| 484 | FCER1G | 5.00E-09 | 2.74E-10 | 25 | 15 | PLA2G4A, SYK, GRB2, FYN, PTPN11, LCK, PIK3CA, SHC1, VAV1, PLCG1, CBL, PIK3CB, PIK3CG, INPP5D, TYROBP |
| 485 | S1PR1 | 5.91E-09 | 3.25E-10 | 49 | 21 | MAPK1, MAPK3, AKT1, VEGFA, KNG1, SRC, RAC1, FYN, LCK, GNAI2, ADCY2, YES1, CCR5, HCK, LPAR2, PDGFRB, ADCY4, PDGFB, HTR1A, ADRA2A, GPR55 |
| 486 | IKBKG | 6.40E-09 | 3.52E-10 | 68 | 25 | UBC, AKT1, TNFRSF1A, CASP8, TRADD, RIPK1, FADD, FASLG, TRAF2, RELA, NFKB1, MAP3K7, PRKCA, NFKBIA, NGF, APP, ATM, IKBKB, BIRC2, MYD88, MAVS, SKP1, TLR2, BTRC, NGFR |
| 487 | LEPR | 6.44E-09 | 3.56E-10 | 11 | 10 | INS-IGF2, STAT3, IGF1, JAK2, LEP, IRS1, PTPN11, JAK3, PTPN1, SOCS3 |
| 488 | PDE8B | 6.44E-09 | 3.56E-10 | 11 | 10 | GNAI2, ADCY2, ADCY9, ADCY8, ADCY6, GNAS, ADCY7, ADCY1, ADCY4, ADCY5 |
| 489 | BLK | 6.47E-09 | 3.59E-10 | 22 | 14 | MAPK14, EGFR, SRC, SYK, FYN, LCK, GNAI2, PLCG1, CBL, HCK, RASA1, PDGFRB, PLCG2, PTK2B |
| 490 | PPP3CB | 6.47E-09 | 3.59E-10 | 22 | 14 | NFATC2, NFATC1, NFATC3, PPP1CB, CALM1, PPP2CA, PLCG1, PPP3CA, PLCG2, PPP2R1A, PPP3R1, PPIA, PPP1CC, PPP3CC |
| 491 | PTHLH | 6.98E-09 | 3.89E-10 | 45 | 20 | POMC, IL6, PTH, ADCY2, CRH, ADRB2, LHCGR, CALCA, ADCY9, GCG, ADM, IAPP, ADCY6, GNAS, GCGR, ADRB1, AVPR2, ADCY1, VIPR2, ADRB3 |
| 492 | IL6ST | 6.98E-09 | 3.89E-10 | 29 | 16 | IL6, STAT3, IFNG, STAT1, JAK2, GRB2, IL2, JAK1, PTPN11, VAV1, SOCS3, IL6R, HCK, PTPN6, CNTF, LIFR |
| 493 | PTGES2 | 7.04E-09 | 3.93E-10 | 9 | 9 | PTGS2, PTGS1, PTGDS, HPGDS, PTGES, PTGIS, TBXAS1, CEBPB, CBR1 |
| 494 | EPHB1 | 7.26E-09 | 4.07E-10 | 19 | 13 | SRC, HRAS, MAP2K1, GRB2, RAC1, FYN, LCK, HCK, GRB10, PTPN13, EPHB2, EFNB2, TIAM1 |
| 495 | CYP26B1 | 7.26E-09 | 4.07E-10 | 19 | 13 | CYP1A2, CYP1A1, CYP2B6, CYP3A4, CYP3A43, CYP3A5, CYP2C19, CYP2C9, CYP2C18, CYP2C8, ALDH1A1, UGT1A1, ALDH1A2 |
| 496 | CTBP1 | 7.88E-09 | 4.43E-10 | 41 | 19 | CREBBP, MYC, BRCA1, CCND1, SIN3A, APC, CTNNB1, RB1, SMAD4, SMAD2, HDAC1, NOTCH1, ATM, TCF7L2, KAT2B, RBBP4, HDAC2, RBBP7, ZEB2 |
| 497 | UGT1A4 | 8.09E-09 | 4.56E-10 | 33 | 17 | CYP2E1, CYP1A2, CYP1A1, CYP2B6, CYP3A4, CYP3A43, CYP3A5, CYP2C19, CYP2C9, CYP2C18, CYP2C8, ADH1B, ADH4, CYP1B1, ADH6, COMT, CYP2D6 |
| 498 | SREBF1 | 8.09E-09 | 4.56E-10 | 33 | 17 | CREBBP, RXRA, UBC, INS-IGF2, EP300, CEBPB, SIRT1, SP1, LEP, CEBPD, CASP3, RORA, PPARGC1A, MED12, MTOR, MED15, KLF5 |
| 499 | ALDOA | 8.32E-09 | 4.71E-10 | 37 | 18 | UBC, EGF, KNG1, ARNT, HIF1A, TGFB1, ALB, FN1, SERPINE1, PKM, GAPDH, GPI, ACTN2, PLG, A2M, CLU, TKT, PFKM |
| 500 | B2M | 8.76E-09 | 4.97E-10 | 74 | 26 | JUN, TGFBI, IL2, IL2RA, LCK, APP, APOA1, ICAM1, NPPA, SNCA, CBL, CD44, PRL, VCAM1, CALCA, IAPP, PTPN6, HSPG2, TTR, CD86, CD247, GSN, TYROBP, IGHG1, HLA-DRB1, NCAM1 |

| **Supplementary Table S5** Potential candidate genes among 500 potential ones required to test their association with ATDH in future | | |
| --- | --- | --- |
| Common genes for SzGene database and 500 candidate gene set | Genes with significant effects on SCZ from SzGene study in 2008 | Genes with significant effects on SCZ from meta-analysis of GWAS data in 2014 |
| AKT1 | COMT | ALDOA |
| ADH1B | DRD2 | CHRM4 |
| ADRA2A | DRD4 | CTNND1 |
| ALDH1A1 | IL1B | CYP26B1 |
| APC | TP53 | DRD2 |
| APP |  | EP300 |
| AR |  | GRIN2A |
| ARRB2 |  | MAPK3 |
| BDNF |  | MCHR1 |
| CALCA |  | NCK1 |
| CALM1 |  | NFATC3 |
| CCK |  | PLCB2 |
| CCKAR |  | SREBF1 |
| CCKBR |  | TAC3 |
| CCL2 |  |  |
| CCR5 |  |  |
| CD4 |  |  |
| CHRM1 |  |  |
| CLOCK |  |  |
| COMT |  |  |
| CRP |  |  |
| CSF2RA |  |  |
| CSF2RB |  |  |
| CSNK1E |  |  |
| CSNK2A1 |  |  |
| CTLA4 |  |  |
| CYP1A2 |  |  |
| CYP26B1 |  |  |
| CYP2C18 |  |  |
| CYP2E1 |  |  |
| CYP3A4 |  |  |
| CYP3A5 |  |  |
| DDC |  |  |
| DRD2 |  |  |
| DRD3 |  |  |
| DRD4 |  |  |
| EGF |  |  |
| EGFR |  |  |
| ERBB2 |  |  |
| ERBB3 |  |  |
| ERBB4 |  |  |
| ESR1 |  |  |
| FGF2 |  |  |
| FN1 |  |  |
| FYN |  |  |
| GAPDH |  |  |
| GDNF |  |  |
| GNAO1 |  |  |
| GNAS |  |  |
| GNRH1 |  |  |
| GPX1 |  |  |
| GRB2 |  |  |
| GRIN2A |  |  |
| GSK3B |  |  |
| GSN |  |  |
| GSTM1 |  |  |
| GSTO1 |  |  |
| GSTP1 |  |  |
| GSTT1 |  |  |
| HDAC3 |  |  |
| HTR1A |  |  |
| HTR1B |  |  |
| HTR1D |  |  |
| HTR2A |  |  |
| HTR2C |  |  |
| HTR4 |  |  |
| HTR5A |  |  |
| ICAM1 |  |  |
| IFNG |  |  |
| IGF1 |  |  |
| IGF1R |  |  |
| IL10 |  |  |
| IL12B |  |  |
| IL1B |  |  |
| IL2 |  |  |
| IL2RB |  |  |
| IL3 |  |  |
| IL4 |  |  |
| IL6 |  |  |
| IL6R |  |  |
| INPP5D |  |  |
| INPPL1 |  |  |
| IRS1 |  |  |
| MAOA |  |  |
| MAOB |  |  |
| MAPK14 |  |  |
| MCHR1 |  |  |
| MET |  |  |
| MMP3 |  |  |
| MMP9 |  |  |
| NGFR |  |  |
| NOS3 |  |  |
| NOTCH1 |  |  |
| NPAS2 |  |  |
| NPSR1 |  |  |
| NPY |  |  |
| NR3C1 |  |  |
| NTS |  |  |
| NTSR1 |  |  |
| OPRM1 |  |  |
| OXT |  |  |
| OXTR |  |  |
| PDGFB |  |  |
| PDGFRB |  |  |
| PDYN |  |  |
| PENK |  |  |
| PIK3CB |  |  |
| PIK3CD |  |  |
| PIK3CG |  |  |
| PIK3R1 |  |  |
| PIK3R2 |  |  |
| PIK3R3 |  |  |
| PLA2G1B |  |  |
| PLA2G4A |  |  |
| PLA2G6 |  |  |
| PLCB1 |  |  |
| PLCB3 |  |  |
| PLCG1 |  |  |
| PMCH |  |  |
| PNOC |  |  |
| PPARG |  |  |
| PPP3CB |  |  |
| PRKCA |  |  |
| PTEN |  |  |
| PTGS2 |  |  |
| RELA |  |  |
| RET |  |  |
| RGS4 |  |  |
| SIRT1 |  |  |
| SREBF1 |  |  |
| SST |  |  |
| TH |  |  |
| TP53 |  |  |
| TSC2 |  |  |
| UGT1A1 |  |  |
| VIPR2 |  |  |
| YWHAB |  |  |
| YWHAE |  |  |
| YWHAG |  |  |
| YWHAH |  |  |
| YWHAZ |  |  |

**Supplementary Table S6** Characteristics of studies included in the meta-analysis

| studies (year) | Ethnicity | Characteristics of cases | Characteristics of controls | Genotyping method | Target gene |
| --- | --- | --- | --- | --- | --- |
| ATDH |  |  |  |  |  |
| An et al.(2012)^1^ | Chinese | an increase of over 2 ULN (upper limit of the normal range) in ALT or conjugated bilirubin (CBIL) levels or a concurrent increase in AST levels, according to the criteria of drug-induced liver injury developed at an international consensus meeting.19 In the present study, patients with increases between 2 and 5 ULN for both ALT and CBIL all had symptoms related to poor liver function. | The inclusion criteria were as follows: (i) daily treatment with isoniazid, rifampicin, pyrazinamide and ethambutol for 2 months, followed by 4 months treatment with isoniazid and rifampicin, with drug dosages calculated according to bodyweight; (ii) normal serum alanine aminotransferase (ALT), aspartate aminotransferase (AST) and bilirubin levels, no symptoms related to abnormal liver function (i.e. jaundice) prior to anti-TB drug treatment and close monitoring of changes in liver function within 6 months of treatment; and (iii) patients with and without hepatotoxicity during drug treatment. Patients with any of the following conditions were excluded from the study: (i) malnutrition; (ii) human immunodeficiency virus type 1 (HIV) infection; (iii) alcoholic liver disease or habitual drinking; (iv) hepatitis B or C infection, liver disease, systemic diseases and/ or treatment with drugs other than the anti-TB drugs that can induce hepatotoxicity; (v) severe TB or cardiac dysfunction that may cause liver dysfunction; and (vi) transient increases in ALT. The control group consisted of randomly selected patients from the same cohort as the hepatotoxicity patients who fulfilled both the inclusion and exclusion criteria. | Sequencing | CYP2E1 |
| Bose et al.(2010)2 | Indian | Liver biochemical parameters more than two times the upper limit of the normal value was considered as hepatotoxicity. | up to 40 U/L aspartate aminotransferase (AST), up to 35 U/L alanine aminotransferase (ALT), 0.2– 1 mg/dL bilirubin, and 3.5–5 g/dL albumin. | PCR-RFLP | CYP2E1 |
| Chatterjee et al. (2009)3 | Indian | 51 pulmonary TB patients meeting the criteria of DILI: ALT >3 ULN; or TBIL>1.0 mg/dl; or ALT <3 ULN but associated with severe anorexia, nausea, vomiting. Patients without pre-existing documented liver diseases: cirrhosis / acute or chronic hepatitis / alcoholic liver disease; F:M =26:25; mean age= 37.16 ± 14.44 | 100 pulmonary TB patients without hepatotoxicity; with matched age, sex, disease severity and drug dosage; F:M =37:63; mean age= 33.08 ± 15.06 | multiplex PCR | GSTM1,GSTT1 |
| Gupta et al. (2013)4 | Indian | 50 TB patients monitored for 6-9 months meeting ICC for DIH: ALT>2ULN; or increased AST and bilirubin, one of them > 2UNL; or increased ALT and/or AST > the baseline levels. F:M =26:24; mean age=37(24-49) | 246 TB patients with evident lesion of TB, positive sputum smear and/or culture for acid-fast bacilli, and normal ALT/AST/TBIL; without chronic liver diseases, positive serological testing for hepatitis B and/or C viruses, alcoholic liver disease or habitual alcohol drinking, using anti-TB drugs prior to enrollment in the study and/or other potentially hepatotoxic drugs, ALT/AST/TBIL>2ULN before the treatment, and refusal to pparticipate in the study. F:M =107:139; mean age=36.5(26-50) | multiplex PCR | GSTM1,GSTT1 |
| Huang et al.(2003)5 | Chinese Taiwanese | 49 ADIH patients with DILI: ALT>2ULN;negative serum hepatitis B virus surface antigen, IgM antibody to hepatitis A virus, and antibody to hepatitis C virus when ALT or AST is elevated; patients withoutwithout any other major hepatic or systemic diseases that may induce elevation of liver biochemical tests, such as alcoholic liver disease, autoimmune hepatitis, congestive heart failure, hypoxia, and bacteremia /Causality assessment score(CAS)>5. F:M = 40:9; mean age= 70(37-83) | 269 patients without hepatotoxicity, with matched sex, age, drug(s), duration of therapy of cases; F:M=229:40; mean age= 59 (24-88) | PCR-RFLP | CYP2E1 |
| Huang et al. (2007) 6 | Chinese | 115 DILI patients with DILI: ALT/AST>5ULN/BV or ALP>2ULN/BV, CAS>5, etc; patients without acute or chronic viral hepatitis/chronic alcoholism or fatty liver/autoimmune liver diseases/any other major hepatic or systemic diseases /CAS>5. F:M = 42:73; mean age= 60.3 ± 12.1 | 115 in- and out-patients without hepatotoxicity, with matched sex, age, drug(s), duration of therapy of cases; F:M=42:73; mean age= 59.1 ± 11.3 | multiplex PCR | GSTM1,GSTT1 |
| Kim et al. (2010)7 | Korean | 57 pulmonary TB and/or TB pleuritis patients without abnormal liver function test result at baseline; or active or chronic hepatitis; or carriers of the hepatitis B or C virus; or heavy alcohol intake; or decreased renal function; or other chronic medical conditions requiring medication; or skin diseases before treatment before treatment, who are dianosed with ATDIH according to the American Thoracic Society guidelines: ALT or AST or TBIL >3ULN. F:M=23:34; mean age=47.3 ± 17.7 | 190 pulmonary TB and/or TB pleuritis patients without adverse reactions to ATD during the treatment period,and without abnormal liver function test result at baseline; or active or chronic hepatitis; or carriers of the hepatitis B or C virus; or heavy alcohol intake; or decreased renal function; or other chronic medical conditions requiring medication; or skin diseases before treatment before treatment, with matched demographic parameters, such as age, sex, height and weight, and baseline levels of AST, ALT or total bilirubin. F:M=61:129; mean age=42.4 ± 17.1 | multiplex PCR | GSTM1,GSTT1 |
| Leiro et al. (2008)8 | Caucasian | 35 ADIH patients: ALT/AST>3ULN; F:M =21:14; age= 34 (24–50) | 60 active TB patients showing no evidence of ADIH; F:M =35:25; age= 31 (25–42) | multiplex PCR | GSTM1,GSTT1 |
| Liu et al. (2014)9 | Chinese | 20 ATDH patients meeting ICC: serum ALT>2ULN or DBil>2ULN or increases of serum AST, ALP and TBil or any index mentioned above>1ULN and associated with liver damage symptoms, such as skin or sclera yellow dye, severe anorexia,nausea, vomiting, fever, rash, itching. F:M=8:12; age=3.59±4.24 | 143 active TB patients meeting (i) Chinese Han children aged between 0 and 16 years; (ii) standard anti-tuberculosis treatment for at least two weeks; (iii) serum transaminases were normal before treatment. Patients matched to cases on age, sex and anti-tuberculosis therapy without pre-existing liver disease, viral hepatitis, chronic alcoholism, or history of intake of other hepatotoxic drugs; and any liver dysfunction during the anti-tuberculosis therapy . F:M=58:85; age=6.12±4.61 | multiplex PCR | GSTM1,GSTT1 |
| Monteiro et al. (2012)10 | Brazilian | 59 TB patients with ATDILI; serum ALT levels > 2ULN; F:M= 14:45 | 118 TB patients without any ATDILI; F:M= 46:72 | multiplex PCR | GSTM1,GSTT1 |
| Rana et al. (2014)11 | North Indian | 55 TB pationts with ATDIH meeting ICC: serum AST or ALT >5ULN, irrespective of symptoms and serum bilirubin levels, or serum AST or ALT >2ULN with hyperbilirubinaemia, and an absence of infection with hepatitis viruses (A, B, C and E) ; no viral hepatitis; F:M=22:33; mean age= 43.6 ± 18.7 | 245 TB pationts without ATDIH; F:M=93:152; mean age= 42.3 ± 16.7 | PCR-RFLP | GSTM1,GSTT1,CYP2E1 |
| Roy et al (2001)12 | Indian | 33 pulmonary TB patients with ADIH meeting ICC: serum bilirubin >3.0 mg/dl and ALT >2 ULN; patients without pre-existing liver disease, evidence of viral hepatitis, chronic alcoholism, history of intake of other hepatotoxic drugs, concurrent medical illness and pregnancy; age : 18 - 75 | 33 age- and sex-matched pulmonary TB patients; patients without any clinical symptoms suggestive of hepatotoxicity and/or biochemical evidence of liver dysfunction during stringent follow up while on ATD treatment; age: 18 - 75 | multiplex PCR | GSTM1,GSTT1 |
| Santos et al.(2013)^13^ | Brazilian | an increase in serum alanine aminotransferase (ALT) level in excess of three times the upper limit of normal after INH treatment.12 ALT was measured prior to anti-tuberculosis treatment, 30 and 60 days after the beginning of treatment or when the physician suspected hepatotoxicity. | Exclusion criteria included patients aged <18 years, those with mental disabilities, chronic liver disease confi rmed by clinical and laboratory data, users of anti-tuberculosis drugs before enrolment in the study, and those with liver function results higher than two times the upper limit of normal before beginning treatment. | TaqMan | CYP2E1 |
| Singla et al. (2014)14 | Indian | 17 TB patients as controls monitored after appearance of symptoms like loss of appetite, nausea, vomiting, fever, and jaundice during the treatment course meeting the International consensus criteria defining ATDH, such as: ALT and AST> 2ULN. F:M= 6:11; mean age= 48.17±17 | 391 newly diagnosed TB patients settled in DOTS centre from a minimum of three generations with the standard respiratory symptoms (cough >3 weeks or longer, pain in the hest,coughing up blood or sputum, fever, sweating at night etc.), sputum smear or culture or typical changes on chest adiographs,and histopathological evidence; without history of heavy use of alcohol or chronic liver diseases or liver cirrhosis or infection of HIV.  F:M= 150:241; mean age= 32.66±15 | multiplex PCR for GSTM1/T1,PCR-RFLP for CYP2E1 | GSTM1,GSTT1,CYP2E1 |
| Sotsuka et al. (2011)15 | Japanese | 20 active TB inpatients with hepatotoxicity; ALT/AST >3 ULN;  F:M= 2:18; mean age= 59.9±20.2 | 92 active TB inpatients without hepatotoxicity; F:M= 24:68; mean age= 50.4 ± 17.3 | multiplex PCR for GSTM1/T1,PCR-RFLP for CYP2E1 | GSTM1,GSTT1,CYP2E1 |
| Tang et al. (2012)16 | Chinese | 89 anti-TB treatment patients with ADIH; ALT >2 ULN or a combined increase in AST/ TBIL >2 ULN;causality was assessed as certain, probable or possible based on the WHO Uppsala Monitoring Centre criteria; F:M =24:65; mean age= 43.7 ± 16.4 (20.0–80.0) | 359 anti-TB treatment patients without ADIH, with matched age, sex, treatment history, disease severity and drug dosage; F:M =96:260; mean age= 43.6 ± 16.4 (17.0–84.0) | multiplex PCR for GSTM1/T1,PCR-RFLP for CYP2E1 | GSTM1,GSTT1,CYP2E1 |
| Teixeira et al. (2011)17 | Brazilian | 26 active TB patients with a history of acute ATDH; transaminase values >3 ULN; F:M =10:16; mean age= 47.58 ± 20.07 | 141 active TB patients with no evidence of anti-TB hepatic side effects with matched age, sex and basal liver function; F:M =67:74; mean age= 42.99 ± 14.94 | multiplex PCR for GSTM1/T1,PCR-RFLP for CYP2E1 | GSTM1,GSTT1,CYP2E1 |
| Wang et al. (2010)18 | Chinese | 104 TB patients with ADIH; ALT/bilirubin/AST/alkaline phosphatase/total bilirubin ≥2 ULN; without hepatitis A antibody, hepatitis B surface antigen and hepatitis C marker; or other factors influencing the levels of AST/ALT/serum proteins, such as alcohol-induced liver disease, hypoxia, auto - immune disease, congestive heart failure and bacteraemia; causality assessment score >5; F:M =34:70; mean age= 48.6 ± 11.98 | 111 TB patients without ADIH; patients without chronic HBV infection, alcohol consumption, obesity, senility and poor nutritional;  F:M = 36:75; mean age: 44.68 ± 11.24 | multiplex PCR for GSTM1/T1,PCR-RFLP for CYP2E1 | GSTM1,GSTT1,CYP2E1 |
| Zaverucha-Do-Valle et al. (2014)19 | Brazilian | hepatotoxicity was defined as a 2-fold increase in the normal upper limit (ALT≥42 [IU]/L) or at least a 2-fold increase in ALT initial levels for those patients with a baseline ALT of >84 IU/L, during the treatment period. | Possible predictors of anti-TB DIH analyzed were age (years), gender, ethnicity, TB clinical form, defined as pulmonary, extra-pulmonary and disseminated, HIV and HCV infection based on positive serology, HBV infection defined by serological HBV surface antigen (HBsAg) detection; HAART use; tobacco use and alcoholism. Concerning ethnicity, patients were classified as White or Non-White according to personal report. Tobacco use defined as current use reported by the patient; and alcoholism defined by a positive CAGE questionnaire. Alanine transferase (ALT) values were obtained at baseline, days 15 and 30, and monthly during of TB treatment. | DNA sequencing | CYP2E1 |
| Gupta et al.(2013)20 | Indian | The patients were monitored for ALT, AST, and total bilirubin levels weekly for 1 month and then monthly until the completion of treatment to detect asymptomatic hepatotoxicity. Increase in ALT over two times of ULN or a combined increase in AST and bilirubin levels, provided one of them is above two times of ULN | The patients with following criteria were included (i) age above 18 years; (ii) positive smear and/ or culture for detection of mycobacteria in clinical samples; and (iii) normal alanine aminotransferase (ALT), aspartate aminotransferase (AST) and total bilirubin levels. Exclusion criteria were (i) patients presenting clinically and laboratory confirmed chronic liver disease such as jaundice; (ii) acute and chronic hepatitis B and/or C or HIV; (iii) alcoholic liver diseases; (iv) a rise of two times the upper limit of normal (ULN) of ALT, AST and total bilirubin levels; (v) medication with anti-TB drugs prior to beginning of the treatment and/or other potentially hepatotoxic drugs; and (vi) Refusal to provide blood sample or signed informed consent form. | PCR-RFLP | CYP2E1 |
| Lee et al.(2010)29 | Chinese Taiwanese | serum ALT level of >2 × ULN after anti-tuberculosis treatment | Inclusion criteria were as follows: adult patients newly diagnosed with active TB, having evident lesions of TB by simple X-ray, computed tomography, positive results of sputum smears and cultures for detection of mycobacteria. Patients with any of the following conditions were excluded from the study: 1) positive serum hepatitis B virus surface antigen, antibody to hepatitis C virus; 2) alcoholic liver disease or habitual alcohol drinking; 3) any other hepatic or systemic diseases that may cause liver dysfunction; 4) abnormal serum ALT, aspartate aminotransferase (AST) or bilirubin levels before anti-tuberculosis treatment. | TaqMan | CYP2E1 |
| Kim et al.(2009)30 | Korean | serum ALT >2 × ULN after anti-tuberculosis treatment | patients with active or chronic hepatitis including alcoholic hepatitis, fatty liver disease, liver cirrhosis, carriers of the hepatitis B or C virus, heavy alcohol intake, decreased renal function and severe cardiac diseases. Age- and sex-matched controls who did not show any adverse reaction during treatment. | SNP-IT™ | CYP2E1 |
| Cho et al.(2007)31 | Korea | serum ALT >2 × ULN after anti-tuberculosis treatment | Patients with any of the following conditions were excluded from the study: (1) abnormal serum alanine transferase (ALT), aspartate transaminase (AST), or bilirubin levels or symptoms related to abnormal liver function such as jaundice before antituberculosis treatment; (2) alcoholic liver disease or habitual alcohol drinking; (3) any other hepatic or systemic diseases that may cause liver dysfunction. | Sequencing | CYP2E1 |
| SCZ |  |  |  |  |  |
| Gravina et al. （2011)21 | Italian | 138 schizophrenic outpatients diagnosed on DSM-IV criteria; patients without a history of alcohol /drug dependence / traumatic head injury / any past or present major medical / neurological illness / any additional psychiatric disorder / any brain pathology identified on magnetic resonance imaging / mental retardation; F:M = 56: 82; mean age=47.33 ± 5.18 | 133 healthy individuals matched with age and gender; F:M =71:62; mean age=47.84± 3.80 | multiplex PCR | GSTM1,GSTT1 |
| Harada et al. (2001)22 | Japanese | 87 schizophrenic patients diagnosed on DSM-IV criteria, without other psychiatric disorders; F:M=34:53; mean age=46.8 ± 14.5 | 117 healthy individuals without history of psychiatric disorders; F:M =30:87; mean age=48.58± 10.2 | multiplex PCR | GSTM1,GSTT1 |
| Matsuzawa et al. (2008)23 | Japanese | 214 schizophrenic patients meeting the DSM-IV criteria without other psychiatric disorders, and with the average age of onset of 24.9 ± 8.3 years.They were subdivided into a disorganized-type(n=19), catatonic-type(n=19), paranoid-type (n=75), residual-type (n=83), and undifferentiated-type (n=18) . F:M=108:106; mean age=51.8± 14.8 | 220 healthy controls with matched gender and geographical origin,without matched age and history of psychotic disorders or drug dependence  F:M=118:102.  mean age=32.9 ±16.7 | multiplex PCR | GSTM1,GSTT1 |
| Pae et al. (2004)24 | Korean | 111 schizophrenic inpatients diagnosed on DSM-IV criteria, without neurological illnesses and medico-surgical illnesses; F:M =67:44; mean age=46.8± 8.7 | 130 healthy individuals without current psychiatric problems or a history of psychiatric illnesses; F:M =74:56; mean age=45.9± 7.4 | multiplex PCR | GSTM1,GSTT1 |
| Raffa et al. (2013)25 | Tunisian | 138 schizophrenic patients diagnosed on DSM-IV-TR criteria; patients without other psychiatric disorders including major depression, schizoaffective disorder, substance abuse (except tobacco consumption ), or mental retardation; F:M =18:120; mean age=32.67± 7.44 | 123 healthy individuals without personal or family history of major psychiatric disorders including schizophrenia, bipolar disorder or major depression; F:M =24:99; mean age=31.28± 5.28 | multiplex PCR | GSTM1,GSTT1 |
| Saadat et al. (2007)26 | Iranian | 292 schizophrenia in-patients diagnosed on DSM-IV criteria; patients without other psychiatric disorders: schizoaffective disorder/ major depressive /episode with psychotic features /substance abuse / bipolar disorder / mental retardation; F:M =86:206; mean age= 41.6± 14.0 | 292 healthy blood donors matched to the patients according to age (±5 years) and gender; subjects without history of psychiatric disorders, cancers, asthma, cataracts, or psychotic disorder including schizophrenia, bipolar disorder and major depression; F:M =86:206; mean age=43.5 ± 15.0 | multiplex PCR | GSTM1,GSTT1 |
| Saruwatari et al. (2013)27 | Japanese | 154 patients diagnosed with schizophrenia based on DSM-IV-TR criteria. They have been treated with antipsychotic medication for at least 3 months prior to enrollment in the study without psychosis due to general medical conditions, substance-related psychosis, and mood disorders with psychotic features. F:M =74:80; mean age=50.2±15.3 | 203 Age-matched and sex-matched healthy controls recruited from participants in a 2-day health screening program. F:M =86:117; mean age=52.4±9.0 | multiplex PCR | GSTM1,GSTT1 |
| Watanabe et al. (2010)28 | Japanese | 627 schizophrenic patients diagnosed on DSM-IV criteria; F:M = 293：334; mean age= 39.9± 13.9 | 620 mentally healthy individuals without personal or family history of psychiatric disorders; F:M=303:317; mean age= 38.2± 10.6 | TaqMan real-time PCR assay | GSTM1,GSTT1 |
| Huo et al. (2012)32 | Chinese | Meeting the Diagnostic and Statistical Manual of Mental Disorders-IV (DSM-IV) criteria for schizophrenia; No physical complications or other psychiatric diseases such as alcoholism or other substance abuse; No history suggesting resistance to antipsychotics treatment; Had received no medication for 4 weeks; Had not previously been treated with atypical antipsychotics | mentally healthy individuals without personal or family history of psychiatric disorders; | DNA sequencing | CYP2E1 |

References:

1 An, H. R., Wu, X. Q., Wang, Z. Y., Zhang, J. X. & Liang, Y. NAT2 and CYP2E1 polymorphisms associated with antituberculosis drug-induced hepatotoxicity in Chinese patients. Clinical and experimental pharmacology & physiology 39, 535-543, doi:10.1111/j.1440-1681.2012.05713.x (2012).

2 Bose, P. D. et al. Role of polymorphic N-acetyl transferase2 and cytochrome P4502E1 gene in antituberculosis treatment-induced hepatitis. Journal of gastroenterology and hepatology 26, 312-318, doi:10.1111/j.1440-1746.2010.06355.x (2011).
3 Chatterjee, S., Lyle, N., Mandal, A. & Kundu, S. GSTT1 and GSTM1 gene deletions are not associated with hepatotoxicity caused by antitubercular drugs. Journal of clinical pharmacy and therapeutics 35, 465-470 (2010).
4 Gupta, V. H. et al. Association of GST null genotypes with anti-tuberculosis drug induced hepatotoxicity in Western Indian population. Annals of hepatology 12, 959-965 (2013).
5 Huang, Y. S. et al. Cytochrome P450 2E1 genotype and the susceptibility to antituberculosis drug-induced hepatitis. Hepatology 37, 924-930, doi:10.1053/jhep.2003.50144 (2003)

6Huang, Y. S. Genetic polymorphisms of drug-metabolizing enzymes and the susceptibility to antituberculosis drug-induced liver injury. Expert opinion on drug metabolism & toxicology 3, 1-8 (2007).
7 Kim, S. H. et al. GSTT1 and GSTM1 null mutations and adverse reactions induced by antituberculosis drugs in Koreans. Tuberculosis (Edinburgh, Scotland) 90, 39-43 (2010).
8 Leiro, V. et al. Influence of glutathione S-transferase M1 and T1 homozygous null mutations on the risk of antituberculosis drug-induced hepatotoxicity in a Caucasian population. Liver international : official journal of the International Association for the Study of the Liver 28, 835-839, doi:10.1111/j.1478-3231.2008.01700.x (2008).
9 Liu, F. et al. Impact of glutathione S-transferase M1 and T1 on anti-tuberculosis drug-induced hepatotoxicity in Chinese pediatric patients. PloS one 9, e115410, doi:10.1371/journal.pone.0115410 (2014).
10 Monteiro, T. P. et al. The roles of GSTM1 and GSTT1 null genotypes and other predictors in anti-tuberculosis drug-induced liver injury. Journal of clinical pharmacy and therapeutics 37, 712-718, doi:10.1111/j.1365-2710.2012.01368.x (2012).
11 Rana, S. V. et al. N-acetyltransferase 2, cytochrome P4502E1 and glutathione S-transferase genotypes in antitubercular treatment-induced hepatotoxicity in North Indians. Journal of clinical pharmacy and therapeutics 39, 91-96, doi:10.1111/jcpt.12105 (2014).
12 Roy, B. et al. Increased risk of antituberculosis drug-induced hepatotoxicity in individuals with glutathione S-transferase M1 'null' mutation. Journal of gastroenterology and hepatology 16, 1033-1037 (2001).
13 Santos, N. P. et al. N-acetyl transferase 2 and cytochrome P450 2E1 genes and isoniazid-induced hepatotoxicity in Brazilian patients. The international journal of tuberculosis and lung disease : the official journal of the International Union against Tuberculosis and Lung Disease 17, 499-504, doi:10.5588/ijtld.12.0645 (2013).
14 Singla, N., Gupta, D., Birbian, N. & Singh, J. Association of NAT2, GST and CYP2E1 polymorphisms and anti-tuberculosis drug-induced hepatotoxicity. Tuberculosis (Edinburgh, Scotland) 94, 293-298, doi:10.1016/j.tube.2014.02.003 (2014).
15 Sotsuka, T., Sasaki, Y., Hirai, S., Yamagishi, F. & Ueno, K. Association of isoniazid-metabolizing enzyme genotypes and isoniazid-induced hepatotoxicity in tuberculosis patients. In vivo 25, 803-812 (2011).
16 Tang, S. W. et al. CYP2E1, GSTM1 and GSTT1 genetic polymorphisms and susceptibility to antituberculosis drug-induced hepatotoxicity: a nested case-control study. Journal of clinical pharmacy and therapeutics 37, 588-593 (2012).
17 Teixeira, R. L. et al. Genetic polymorphisms of NAT2, CYP2E1 and GST enzymes and the occurrence of antituberculosis drug-induced hepatitis in Brazilian TB patients. Memorias do Instituto Oswaldo Cruz 106, 716-724 (2011).
18 Wang, T. et al. Genetic polymorphisms of cytochrome P450 and glutathione S-transferase associated with antituberculosis drug-induced hepatotoxicity in Chinese tuberculosis patients. The Journal of international medical research 38, 977-986 (2010).
19 Zaverucha-do-Valle, C. et al. The role of cigarette smoking and liver enzymes polymorphisms in anti-tuberculosis drug-induced hepatotoxicity in Brazilian patients. Tuberculosis (Edinburgh, Scotland) 94, 299-305, doi:10.1016/j.tube.2014.03.006 (2014).

20 Gupta, V. H. et al. Association of N-acetyltransferase 2 and cytochrome P450 2E1 gene polymorphisms with antituberculosis drug-induced hepatotoxicity in Western India. Journal of gastroenterology and hepatology 28, 1368-1374, doi:10.1111/jgh.12194 (2013).
21 Gravina, P. et al. Genetic polymorphisms of glutathione S-transferases GSTM1, GSTT1, GSTP1 and GSTA1 as risk factors for schizophrenia. Psychiatry research 187, 454-456 (2011).
22 Harada, S., Tachikawa, H. & Kawanishi, Y. Glutathione S-transferase M1 gene deletion may be associated with susceptibility to certain forms of schizophrenia. Biochemical and biophysical research communications 281, 267-271 (2001).
23 Matsuzawa, D. et al. Association study between the genetic polymorphisms of glutathione-related enzymes and schizophrenia in a Japanese population. Am J Med Genet B Neuropsychiatr Genet 150B, 86-94 (2009).
24 Pae, C. U. et al. Glutathione S-transferase M1 polymorphism may contribute to schizophrenia in the Korean population. Psychiatric genetics 14, 147-150 (2004).
25 Raffa, M. et al. Relationship between GSTM1 and GSTT1 polymorphisms and schizophrenia: a case-control study in a Tunisian population. Gene 512, 282-285 (2013).
26 Saadat, M., Mobayen, F. & Farrashbandi, H. Genetic polymorphism of glutathione S-transferase T1: a candidate genetic modifier of individual susceptibility to schizophrenia. Psychiatry research 153, 87-91 (2007).
27 Saruwatari, J. et al. Possible associations between antioxidant enzyme polymorphisms and metabolic abnormalities in patients with schizophrenia. Neuropsychiatric disease and treatment 9, 1683-1698, doi:10.2147/NDT.S52585 (2013).
28 Watanabe, Y., Nunokawa, A., Kaneko, N. & Someya, T. A case-control study and meta-analysis of association between a common copy number variation of the glutathione S-transferase mu 1 (GSTM1) gene and schizophrenia. Schizophrenia research 124, 236-237 (2010).

29 Lee, S. W. et al. NAT2 and CYP2E1 polymorphisms and susceptibility to first-line anti-tuberculosis drug-induced hepatitis. The international journal of tuberculosis and lung disease : the official journal of the International Union against Tuberculosis and Lung Disease 14, 622-626 (2010).

30 Kim, S. H. et al. Genetic polymorphisms of drug-metabolizing enzymes and anti-TB drug-induced hepatitis. Pharmacogenomics 10, 1767-1779, doi:10.2217/pgs.09.100 (2009).

31 Cho, H. J. et al. Genetic polymorphisms of NAT2 and CYP2E1 associated with antituberculosis drug-induced hepatotoxicity in Korean patients with pulmonary tuberculosis. Tuberculosis (Edinburgh, Scotland) 87, 551-556, doi:10.1016/j.tube.2007.05.012 (2007).

32 Huo, R. et al. Genetic polymorphisms in CYP2E1: association with schizophrenia susceptibility and risperidone response in the Chinese Han population. PloS one 7, e34809, doi:10.1371/journal.pone.0034809 (2012).

**Supplementary Table S7** FPRP values and power estimates for the assumed effect sizes of *GSTM1* genes on different diseases

| OR | Power | Prior | | | | | |
| --- | --- | --- | --- | --- | --- | --- | --- |
|  |  | 0.05 | 0.01 | 0.001 | 0.0001 | 0.00001 | 0.00001 |
| ATDH |  |  |  |  |  |  |  |
| 0.6 | 0.918 | 0.088 | 0.334 | 0.835 | 0.981 | 0.998 | 0.998 |
| 1.5 | 0.699 | 0.112 | 0.397 | 0.869 | 0.985 | 0.998 | 0.998 |
| 2 | 0.998 | 0.081 | 0.315 | 0.823 | 0.979 | 0.998 | 0.998 |
|  |  |  |  |  |  |  |  |
| SCZ |  |  |  |  |  |  |  |
| 0.7 | 0.901 | 0.063 | 0.259 | 0.779 | 0.972 | 0.997 | 0.997 |
| 1.5 | 0.969 | 0.059 | 0.245 | 0.766 | 0.970 | 0.997 | 0.997 |
| 2 | 1.000 | 0.057 | 0.239 | 0.760 | 0.969 | 0.997 | 0.997 |
| Green background implies noteworthiness at 0.2 level | | | | | | | |


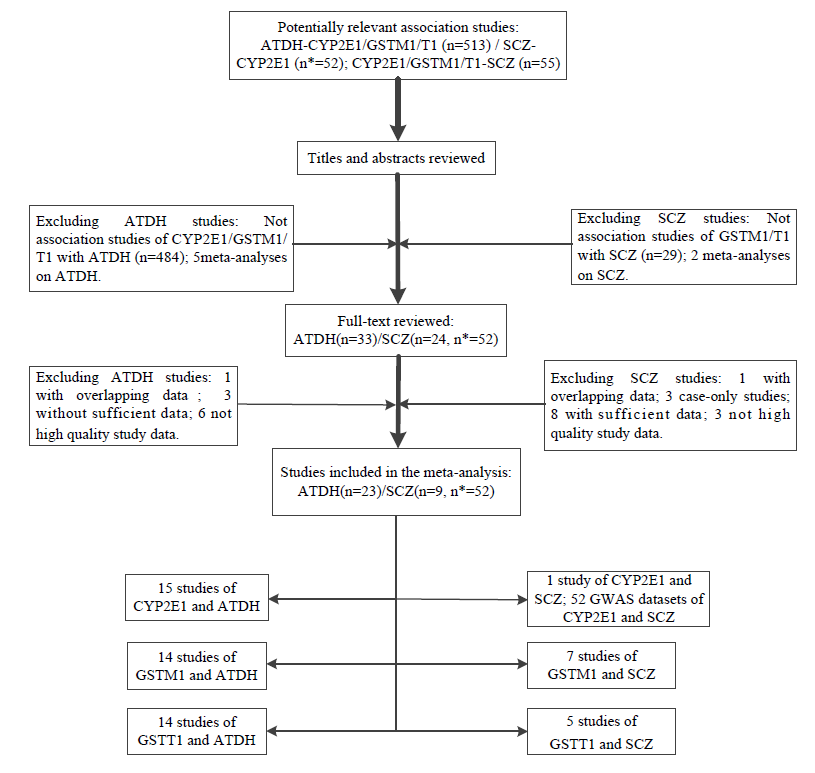


**Supplementary Figure 1**. A flow diagram of the study selection process.
